# Aryl Halide Carboxylation via Decarboxylative Metal–Halogen Exchange

**DOI:** 10.1021/jacsau.5c01545

**Published:** 2026-02-02

**Authors:** Daniel J. Ryder-Mahoney, Ken Yamazaki, Gregory J. P. Perry

**Affiliations:** † School of Chemistry and Chemical Engineering, 7423 University of Southampton, Southampton SO17 1BJ, U.K.; ‡ Division of Applied Chemistry, 12997 Okayama University, Tsushimanaka, Okayama 700-8530, Japan

**Keywords:** Carboxylation, Metal−Halogen Exchange, Carbon Isotope Labeling, Dual-Function Reagent, CO_2_ Transfer

## Abstract

A unique mode of metal–halogen exchange is reported for the carboxylation of aromatic halides. The process is mediated by the potassium salt of a commercially available carboxylic acid, which acts as the source of CO_2_ and metalating agent. The procedure demonstrates that readily available, bench-stable carboxylic acid salts can generate metalating agents *in situ* for metal–halogen exchange, thus avoiding sensitive and hazardous organometallics. The carboxylation proceeds under mild conditions, shows broad substrate scope and avoids specialized apparatus such as pressurized containers or strictly inert conditions. Application to the carbon isotope labeling of biologically relevant compounds is also reported, including a late-stage carbon isotope exchange. Experimental and computational studies support our proposed mechanism of decarboxylative metal–halogen exchange in which the metalating agent and CO_2_ are generated *in situ* from the carboxylate salt.

## Graphical abstract

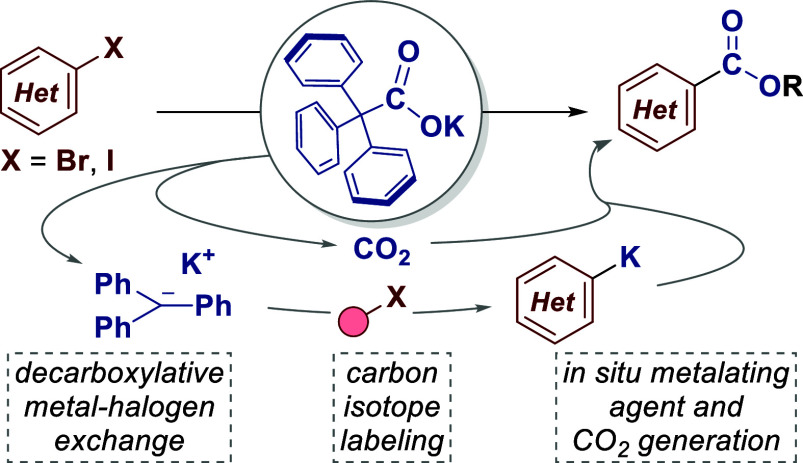

The carboxylic acid motif is a highly valuable functional group found in a variety of important molecules.^1^ They are also key synthetic building blocks as they can be transformed into other functionalities, such as esters, amides and alcohols.^2^ Carboxylation is also the method of choice for installing carbon isotope labels. However, efficient and practical methods are needed in this area due to the high costs associated with labeled compounds.^3^

The carboxylation of carbon–halogen (C–X) bonds is particularly useful for preparing carboxylic acids as the site of the C–X bond controls the position of carboxylation.^4^ Metal–halogen exchange is a fundamental process in organometallic chemistry and a widely adopted strategy in synthesis.^5^ Ever since the pioneering work by Wittig and Gilman, metal–halogen exchange has been closely entwined with the field of carboxylation.^6^ Indeed, Gilman’s first evidence of metal–halogen exchange involved carboxylation of aryl halide **I** to give benzoic acid **III** via metalated intermediate **II** (Scheme 1A).^6b^ Of the various methods for C–X carboxylation, metal–halogen exchange has established itself among the most powerful. Significant advancements have been made in this area, but the habitual use of hazardous and difficult-to-handle organometallics can create barriers toward application.^7^

**Scheme 1:**
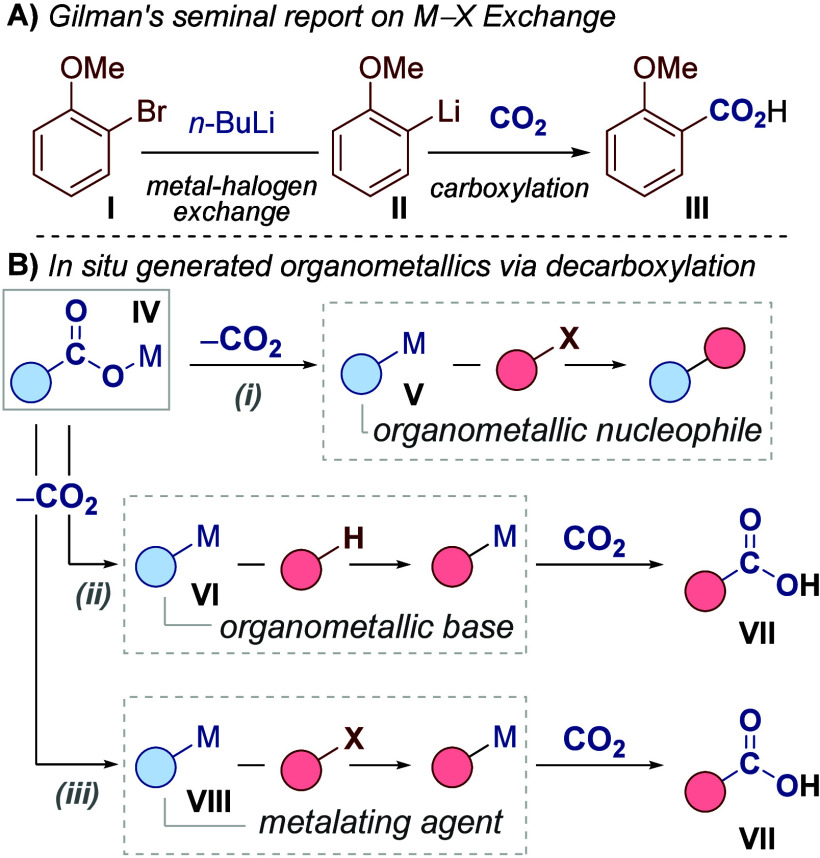
(A) Classical Metal–Halogen Exchange. (B) (i) Established Reactivity: Decarboxylative Functionalization, (ii) Our Previous Work: Decarboxylative Metalation via Deprotonation, (iii) This Work: Decarboxylative Metalation via Metal–Halogen Exchange

When considering reactions between carboxylic acids/carboxylates and organohalides, the simplest process involves ester formation via nucleophilic substitution. More recently, decarboxylative functionalization has emerged to enable cross-coupling between carboxylic acids and organohalides (Scheme 1B­(i)).^8^ These couplings have received considerable attention as they replace traditional nucleophilic organometallics, which can present hazards, are difficult-to-handle and are of limited availability, with stable and abundant carboxylic acid derivatives **IV**. Despite sustained investigation, this field limits the role of the *in situ* generated organometallic to a nucleophilic species **V** for coupling reactions. Though significant, this fails to tap into the wide-ranging functions that organometallics are capable of, most strikingly highly valuable metalation chemistry.^5^

We have recently established a program to expand the capabilities of *in situ* formed organometallics. Previously, we revealed a process for C–H carboxylation that proceeded via decarboxylative deprotonation (Scheme 1B, (ii)).^9–11^ In this process, carboxylate **IV** underwent decarboxylation to provide an *in situ* organometallic **VI** capable of deprotonating C–H bonds. Impressively, the CO_2_ generated during the initial decarboxylation step was captured by the deprotonated intermediate to give carboxylic acid products **VII**. We therefore described carboxylate **IV** as a dual-function reagent as it provided a combined source of base and CO_2_. We questioned whether an analogous process of decarboxylative metalation with aryl halides would be possible (Scheme 1B­(iii)). In this process, the *in situ* generated organometallic **VIII** would promote metal–halogen exchange. The resulting metalated substrate would then capture the *in situ* generated CO_2_ to give product **VII**. In this way, dual-function reagent **IV** would act as a source of metalating agent and CO_2_. This C–X carboxylation via decarboxylative metal–halogen exchange would augment existing CO_2_ transfer^9,10a,12^ and exchange reactions^13,14^ while delivering a novel concept in organometallic chemistry and a useful tool for carbon isotope labeling.^15^

We began our study by submitting aryl halide **1a-I** to our previously reported reaction conditions (Table 1).^9^ Potassium triphenylacetate **2-K**, which is easily prepared from commercially available triphenylacetic acid (CAS: 595–91–5), was used as the dual source of CO_2_ and metalating agent. This provided the carboxylated product (isolated as the corresponding methyl ester) **3a** in reasonable yield (Entry 1). The process was optimized by simply doubling the equivalents of dual-function reagent **2-K** (Entry 2). The reaction was similarly efficient with aryl bromide **1a-Br** (Entry 3). Poor reactivity was observed with aryl chloride **1a-Cl** (Entry 4), however, engaging these substrates in metal–halogen exchange is notoriously difficult.^7^ Interestingly, whereas our previous C–H carboxylation procedure required long reaction times, this C–X carboxylation was completed in as little as 5 min (Entries 5–7), indicating the greater efficiency of metal–halogen exchange over deprotonation.^9,16^ Following a solvent screen, we chose DMF as the solvent for this investigation, though DMSO was also effective (Entry 8).^16,17^ The cesium and rubidium salts **2-Rb** and **2-Cs** (Entries 11 and 12) were also effective carboxylating agents, but diminished yields were observed for the lithium and sodium salts **2-Li** and **2-Na** (Entries 9 and 10). We found that the lithium and sodium salts were hygroscopic, therefore, we believe this drop in yield is likely due to the presence of water, rather than any observable reactivity difference.^16^

**Table 1:**
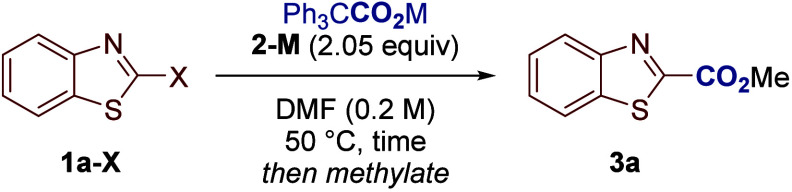
Reaction Optimization^a^

Entry	X	M	time	NMR yield **3a** (%)
1^b^	I	K	16 h	45
2	I	K	16 h	90
3	Br	K	16 h	96
4	Cl	K	16 h	0, 7^c^
5	Br	K	30 min	96 (91)^d^
6	Br	K	5 min	91
7	Br	K	1 min	trace
8^e^	Br	K	30 min	73
9	Br	Li	30 min	37
10	Br	Na	30 min	63
11	Br	Rb	30 min	93
12	Br	Cs	60 min	85

a Reaction conditions: **1a-X** (0.5 mmol), **2-M** (2.0 equiv), DMF (0.2 M), 50 °C. Then MeI (8.0 equiv), 50 °C, 2 h. DMSO = dimethyl sulfoxide. DMF = *N*,*N*-dimethylformamide.

b **2-K** (1.0 equiv).

c 140 °C.

d Isolated yield.

e DMSO instead of DMF.

We have been able to demonstrate our decarboxylative metal–halogen exchange process in the carboxylation of various heteroaromatic bromides (Scheme 2). The reaction was applicable to substrates bearing electron-donating (**3b**, **3c**) and electron-withdrawing groups (**3g**, **3h**), and with substitution around the heteroaromatic ring (**3e**, **3i**, **3j**). We were impressed that sensitive functional groups, such as esters (**3g**, **3h**) and aldehydes (**3k**) were tolerated. We also note that all reactions were carried out in DMF (*N*,*N*-dimethylformamide) but we have never observed competing formylation reactions, again highlighting the functional group tolerance and complementarity of our approach to traditional metal–halogen exchange.^18^ The compatibility of the reaction with various functional groups was further investigated by means of a robustness screen.^16,19^ This revealed that ketones, alkenes, aryl triflates, alkynes, nitriles, nitro groups and alkyl chlorides are all tolerated under the reaction conditions. This improved tolerance over traditional metal–halogen exchange may be due to the electrophile (CO_2_) being present in the reaction mixture while the metalated intermediate (see **5**, Scheme 3) is formed. Conversely, traditional metal–halogen exchange requires the metalated intermediate to be formed before the addition of the desired electrophile, resulting in a greater chance of side reactions occurring.^20^ Several instances of selective C–X carboxylation are also noteworthy, for example, C–Br bonds were preferentially carboxylated in the presence of C–Cl and C–F bonds (**3d**, **3e**). Moreover, dibrominated substrates could be selectively monocarboxylated (**3f**). Impressive examples of site-selective C–X carboxylation were observed, for example, 2- and 5-brominated thiazoles **1n-Br** and **1o-Br** gave 2- and 5-carboxylated products **3n** and **3o** in good yields. We also observed that carboxylation with less activated heteroaromatics, such as benzothiophenes and benzofurans, proceeded efficiently (**3t**, **3u**). The carboxylation of less reactive substrates (see **3r** and **3s**) is currently outside the limits of this methodology,^21^ however, some other 6-membered rings reacted well under the standard conditions (see **3v** and **3w**). Overall, we have carboxylated a variety of (hetero)­aromatics, including (benzo)­thiazoles, imidazoles, oxadiazoles, benzothiophenes and benzofurans, which are among the most used ring systems in drug discovery.^22^

**Scheme 2:**
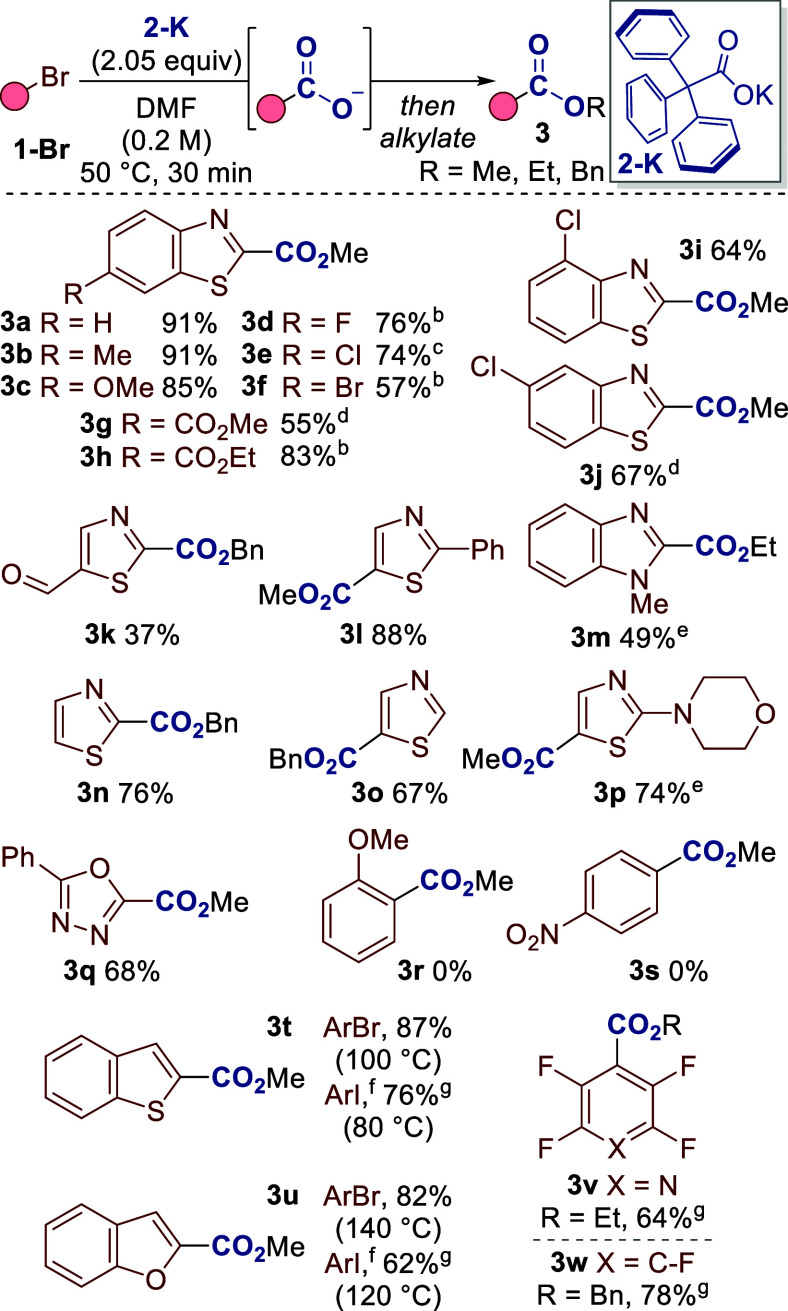
Scope of the C–X Carboxylation^a^

a Reaction conditions: **1-Br** (1.0 mmol), **2-K** (2.05 equiv), DMF (0.2 M), 50 °C, 30 min. See the Supporting Information for details on the alkylation.
b 0.1 M.
c 60 °C.
d 0.05 M.
e 100 °C.
f The aryl iodide (**1r-I**/**1s-I**) was used.
g NMR yield.

**Scheme 3:**
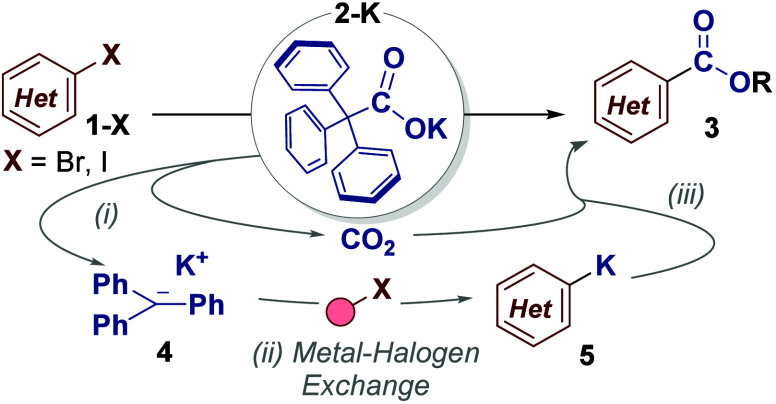
Proposed Mechanism for C–X Carboxylation

Our proposed mechanism for C–X carboxylation is provided in Scheme 3. Decarboxylative metalation consists of decarboxylation of **2-K** to give the metalating agent **4** (step (i)), followed by metal–halogen exchange with the substrate **1-X** to give metalated intermediate **5** (step (ii)). Carboxylation of **5** with the *in situ* formed CO_2_ and subsequent alkylation would then lead to the desired product **3** (step (iii)).

To gather evidence for our proposed decarboxylative metal–halogen exchange mechanism we first conducted the reaction in the presence of a radical scavenger, TEMPO (2,2,6,6-tetramethylpiperidine 1-oxyl). The reaction was largely unaffected, suggesting radical intermediates were not involved in this process (Scheme 4A). Next, we could isolate side product ester **6** from the reaction (Scheme 4B). The structure of **6** was unequivocally determined through X-ray crystallography (Scheme 4E).^23^ In a separate experiment, the potassium salt **2-K** and trityl bromide **7** reacted to form ester **6** in similarly high yield (Scheme 4C). Thus, to explain the formation of **6**, we believe **2-K** first mediates decarboxylative metal–halogen exchange to give trityl bromide **7** (Scheme 4D, steps (i) and (ii), cf. Scheme 3, steps (i) and (ii)). Trityl bromide **7** then reacts with another equivalent of carboxylate **2-K** to give ester **6**. This provides indirect evidence of decarboxylative metal–halogen exchange and explains the need for two equivalents of **2-K**. In this way, the process is reminiscent of metal–halogen exchange involving *t*-BuLi, which also requires two equivalents of the organometallic to destroy a reactive alkyl halide side product.^24^

**Scheme 4:**
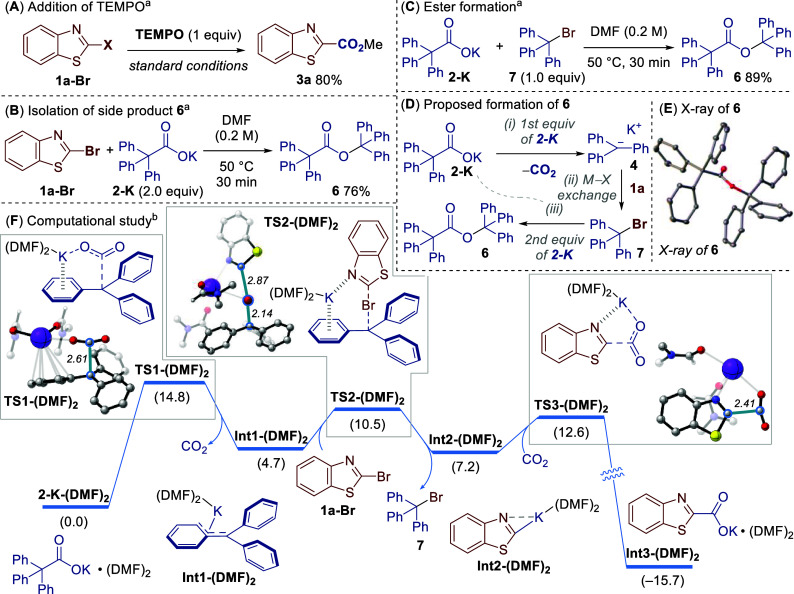
Mechanistic Studies

a See the Supporting Information for further details on the reaction conditions.
b Potential energy diagram for the C–X carboxylation computed at the SMD­(DMF)/B3LYP-D3/def2-TZVPP//SMD­(DMF)/B3LYP-D3/def2-SVP level of theory. Gibbs free energies (Δ*G* [kcal mol^–1^]) and bond lengths (Å) are provided in the inset.

To provide further evidence for our proposed decarboxylative metal–halogen exchange mechanism, a density functional theory (DFT) study was conducted (Scheme 4F). We systematically explored the coordination of the potassium cation with multiple DMF molecules (n = 1, 2, 3) in the calculations to account for solvation of the potassium cation.^16^ The initial decarboxylation of **2-K-(DMF)**_**2**_ proceeded via the transition state **TS1-(DMF)_2_** with an activation energy barrier of 14.8 kcal mol^–1^. A cation-π interaction was observed, which contributed to the stabilization of the transition state through charge delocalization. The metalating agent **Int1-(DMF)**_**2**_, formed after the release of CO_2_, then underwent the metal–halogen exchange with **1a-Br** via the transition state **TS2-(DMF)**_**2**_ (Δ*G* ^‡^ = 10.5 kcal mol^–1^). This process was facilitated by the interaction of the potassium cation with both the phenyl ring and the nitrogen atom of **1a-Br**. Finally, carboxylation of **Int2-(DMF)**_**2**_ furnished the carboxylate **Int3-(DMF)**_**2**_ (Δ*G* = −15.7 kcal mol^–1^). Overall, the activation energy barrier for the rate-determining decarboxylation step is consistent with the reaction conditions employed.

Labeled compounds are key to the development of new medicines and agrochemicals, and relied upon in mechanistic studies.^3,25,26^ Due to the price, hazards and scarcity of some isotopes, practical and efficient methods are of great relevance in this field, for example, ^14^CO_2_ is radioactive, costs >£1,500/mmol and only produced in select locations.^13a,14i^ Dual-function reagent **2-K** is a bench-stable and weighable solid, so offers a practical alternative for carbon isotope labeling. Our C–X carboxylation only requires 2.0 equiv of **2-K** and side product **6** can undergo routine hydrolysis to regenerate the precursor to **2-K** if desired.^27,28^ Scheme 5 reveals the formation of various labeled drug derivatives and precursors via C–X carboxylation. We have also demonstrated a formal carbon isotope exchange^13,14^ for late-stage isotope labeling of the anti-inflammatory drug Febuxostat (Scheme 6). Accordingly, Febuxostat **3aa-H** underwent decarboxylative bromination to provide aryl bromide intermediate **1aa-Br**.^29^ Exposure to our C–X carboxylation conditions with labeled reagent 2-K* then provided the labeled Febuxostat ester 3aa*.

**Scheme 5:**
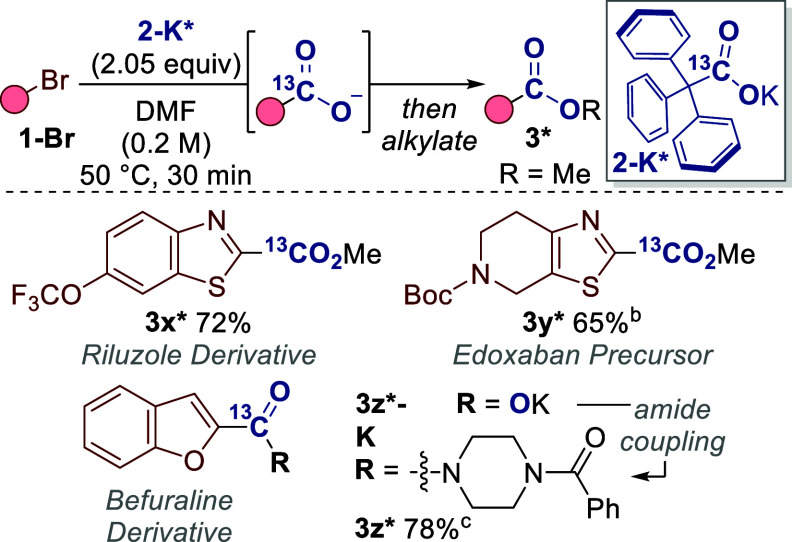
Carbon Isotope Labeling^a^

a Reaction conditions: **1-Br** (0.25 mmol), 2-K* (2.05 equiv), DMF (0.2 M), 50 °C, 30 min. See the Supporting Information for details on the alkylation and amide coupling steps.
b 1 h.
c Carboxylation performed at 140 °C.

**Scheme 6:**
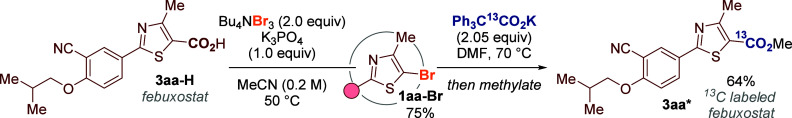
Late-Stage Carbon Isotope Exchange^a^

a Reaction conditions: Bromination – **3aa** (0.5 mmol), Bu_4_NBr_3_ (2.0 equiv), K_3_PO_4_ (1.0 equiv), MeCN (0.2 M), 50 °C, 16 h. Extraction/filtration. Carboxylation – **1aa-Br** (0.25 mmol), 2-K* (2.05 equiv), DMF (0.2 M), 70 °C, 30 min. See the Supporting Information for details on the methylation.

In conclusion, we have introduced decarboxylative metalation as a unique mode of reactivity for metal–halogen exchange. The procedure employs carboxylate **2-K** as a dual-function reagent to generate a metalating agent and CO_2_ *in situ*, thereby avoiding hazardous organometallics. This reactivity has been applied in the C–X carboxylation of various heteroaromatic substrates and shows notable functional group tolerance and selectivity in comparison to traditional metal–halogen exchange pathways. Preliminary mechanistic studies and computational analysis provide support for our proposed decarboxylative metal–halogen exchange in which the metalating agent is generated *in situ*. The application of this method in carbon isotope chemistry has also been demonstrated in the labeling of molecules with known bioactivity and a formal late-stage carbon isotope exchange.

## Supplementary Material



## Notes

Supporting Information is available free of charge via the Internet at . Small molecule crystal data is deposited with the Cambridge Crystallographic Data Centre (CCDC) with deposition numbers 2333766 (for **6**) and 2371571 (for **3y**). The Supporting Information is available free of charge at https://pubs.acs.org/doi/10.1021/jacsau.5c01545.

Experimental details, optimization studies, scope of the C-X carboxylation of aryl halides, robustness screen, unsuccessful examples, mechanistic studies, computational studies, isotope labeling, and NMR data (PDF)
