# Supplementary material for: Aryl Halide Carboxylation via Decarboxylative Metal–Halogen Exchange
Source: JACS Au. 2026 Feb 2;6(2):773–8. doi: 10.1021/jacsau.5c01545 (PMC12933346; doi:10.1021/jacsau.5c01545)
Supplement: Supplementary file 1 [file au5c01545_si_001.pdf]

SUPPORTING INFORMATION

Aryl Halide Carboxylation via Decarboxylative Metal-Halogen Exchange

Daniel J. Ryder-Mahoney,[a] Ken Yamazaki,[b]* and Gregory J. P. Perry[a]*

[a] School of Chemistry, University of Southampton, Southampton, SO17 1BJ (UK)

[b] Division of Applied Chemistry, Okayama University, Tsushimanaka, Okayama 700-8530, (Japan)

*Corresponding Authors

Gregory J. P. Perry: gregory.perry@soton.ac.uk

Ken Yamazaki: k-yamazaki@okayama-u.ac.jp

Contents

1. General Experimental 4
2. Experimental procedures for the preparation of starting materials 5
2.1. Preparation of 2,2,2-triphenylacetic Acid (2') 5
2.2. General procedure 1 for the preparation of triphenylacetate salts 6
Potassium 2,2,2-triphenylacetate (2-K) 6
Lithium 2,2,2-triphenyl acetate (2-Li) 7
Sodium 2,2,2-triphenyl acetate (2-Na) 7
Rubidium 2,2,2-triphenyl acetate (2-Rb) 8
Cesium 2,2,2-triphenyl acetate (2-Cs) 8
3. Optimization Studies 10
3.1. General procedure for optimisation studies 10
3.1.1 Reaction Duration 10
3.1.2. Solvent Screening 11
3.1.3. Introduction of H_2O/open to air reaction 11
3.1.4. Metal Screening 12
3.1.5. Comparing the reactivity of C–H carboxylation and C–X carboxylation 14
4. The scope of the C-X carboxylation of aryl halides 15
4.1. General procedure 2 for the carboxylation of aryl halides 15
Methyl benzo[*d*]thiazole-2-carboxylate (3a) 15
Methyl 6-Methylbenzo[*d*]thiazole-2-carboxylate (3b) 16
Methyl 6-Methoxybenzo[*d*]thiazole-2-carboxylate (3c) 16
Methyl 6-Fluorobenzo[*d*]thiazole-2-carboxylate (3d) 17
Methyl 6-Chlorobenzo[*d*]thiazole-2-carboxylate (3e) 18
Methyl 6-Bromobenzo[*d*]thiazole-2-carboxylate (3f) 18
Dimethyl benzothiazole-2,6-dicarboxylate (3g) 19
6-Ethyl 2-Methyl Benzothiazole-2,6-dicarboxylate (3h) 20
Methyl 4-Chlorobenzo[*d*]thiazole-2-carboxylate (3i) 20
Methyl 5-Chlorobenzo[*d*]thiazole-2-carboxylate (3j) 21
Benzyl 5-formylthiazole-2-carboxylate (3k) 21
Methyl 2-phenylthiazole-5-carboxylate (3l) 22
Ethyl 1-Methyl-1H-benzo[*d*]imidazole-2-carboxylate (3m) 23
Benzyl thiazole-2-carboxylate (3n) 23
Benzyl Thiazole-5-carboxylate (3o) 24
Methyl 2-morpholinothiazole-5-carboxylate (3p) 24
Methyl 5-phenyl-1,3,4-oxadiazole-2-carboxylate (3q) 25

Methyl Benzo[*b*]thiophene-2-carboxylate (3t) 25
Methyl Benzofuran-2-carboxylate (3u) 26
Ethyl tetrafluoroisonicotinate (3v) 27
Benzyl 2,3,4,5,6-pentafluorobenzoate (3w) 27
5. Robustness Screen 29
6. Unsuccessful Examples 30
7. Mechanistic Studies 31
7.1. Radical Trap Experiment 31
7.2. Synthesis of trityl 2,2,2-triphenylacetate (6) 32
7.3. Isolation of trityl 2,2,2-triphenylacetate (6) from the reaction mixture 34
7.4. Synthesis of Potassium benzothiazole-2-carboxylate 35
7.5. Carboxylate Reactivity 36
8. Computational Studies 37
8.1. Computational Methods 37
8.2. Computational Methods 38
8.3. Cartesian Coordinates 39
9. Isotope Labeling 73
9.1. Preparation of ^{13}C-labeled 2,2,2-triphenyl acetic acid (2'*) 74
9.2. Preparation of ^{13}C-labeled potassium 2,2,2-triphenyl acetic acid (2-K*) 76
9.3. Synthesis of isotope labeled compounds 77
Riluzole derivative (3x) 77
^{13}C-Labeled riluzole derivative (3x*) 77
Edoxaban precursor (3y) 78
^{13}C-Labeled edoxaban precursor (3y*) 79
General procedure 3 for the synthesis of befuraline derivative (3z) 80
^{13}C-labeled befuraline derivative (3z*) 81
9.4. Carbon Isotope Exchange 83
Preparation of 5-(5-Bromo-4-methylthiazol-2-yl)-2-isobutoxybenzonitrile (1aa-Br) 83
Febuxostat ester (3aa) 84
^{13}C-Labeled febuxostat ester (3aa*) 85
9.5. Recovery of 2,2,2-Triphenylacetic Acid 86
10. References 87
11. NMR Data 89

1. General Experimental

^1H (400 MHz), ^{13}C (100 MHz) and $^{19}F\{1H\}$ (376 MHz) NMR measurements were carried out using either a Bruker AVII400 FT-NMR or AVIII HD 400 FT-NMR spectrometer. For ^{13}C NMR spectra, all spectra were recorded with decoupling to ^1H unless otherwise noted. Chemical shifts in ^1H NMR spectra are reported in delta (δ) units, parts per million (ppm) relative to residual solvent peaks found at δ = 7.26 ppm ($CDCl_3$), δ = 2.50 ppm (DMSO-d_6) or δ = 4.79 ppm (D_2O). Chemical shifts in ^{13}C NMR spectra are reported in delta (δ) units, parts per million (ppm) relative to the residual solvent peaks found at δ = 77.16 ppm ($CDCl_3$) or δ = 39.52 ppm (DMSO-d_6). All J coupling constants were measured in Hertz. The following abbreviations are used for spin multiplicity: s = singlet, d = doublet, t = triplet, q = quartet, m = multiplet, br = broad.

High resolution electrospray ionisation (ESI) samples were analysed using a MaXis (Bruker Daltonics, Bremen, Germany) time of flight (TOF) mass spectrometer. Samples were introduced to the mass spectrometer via a Dionex Ultimate 3000 autosampler and uHPLC pump. High resolution electron ionisation (EI) mass spectrometry was carried out using a LECO HRT+ (LECO Corporation, St Joseph, Mi, USA) time-of-flight (TOF) mass spectrometer.

Analytical thin layer chromatography (TLC) was performed on Merck precoated analytical plates, 0.25-mm thick, silica gel 60 F254.

Flash chromatography was performed using Silica Gel high purity grade, pore size 60 Å, 230-400 mesh particle size, 40-60 μm particle size, purchased from Merck.

All reagents and solvents were bought from commercial suppliers and used without further purification unless otherwise noted. Anhydrous AcroSeal™ *N*,*N*-dimethylformamide (DMF) was purchased from Thermo Fisher Scientific Inc. and used without further purification. The concentration of *n*-BuLi in hexane was determined by titration prior to use.[1] The $^{13}CO_2$ used for the labeling experiments was purchased from Merck (364592-1L, packaged in a 450 mL carbon steel lecture bottle with brass CGA 110/180 valve and equipped with a stainless-steel control valve. Merck state that nominal gas pressures at 21 °C are 20 psig).

2. Experimental procedures for the preparation of starting materials

2.1. Preparation of 2,2,2-triphenylacetic Acid (2')

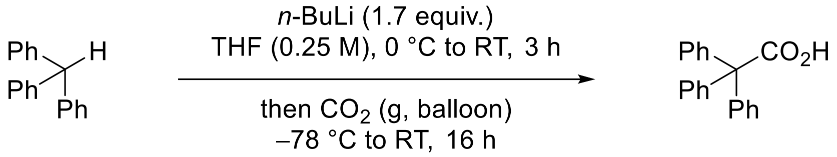

Adapted from the works of Perry et al.[2] A 500 mL 3-neck RBF was equipped with a stir bar and dried with a heat gun under *vacuo*. The flask was charged with triphenylmethane (12.2 g, 50.0 mmol, 1.0 equiv.) and placed under an inert atmosphere through cycles of N_2 gas and vacuum. THF (200 mL, 0.25 M) was added, and the solution was cooled to 0 °C. *n*-BuLi (42.1 mL, 85.0 mmol, 1.7 equiv., 2.02 M in hexane) was added dropwise to form a red solution. Upon complete addition of *n*-BuLi, the solution was warmed to room temperature and stirred for 3 h. The mixture was solidified in a liquid N_2 bath, then placed under vacuum for 5 min. The tap to the vacuum was closed and the mixture was warmed to –78 °C in a dry ice/acetone bath to give a red solution (*caution*: upon thawing, N_2 gas is released from the reaction mixture creating a pressure build up. To prevent a large pressure build-up, thaw the reaction slowly. If necessary, keep the vacuum tap open at the beginning of the process to avoid pressure build-up, then close to avoid evaporating too much solvent). The flask was filled with CO_2 gas *via* balloon attached with a needle and the mixture was warmed to room temperature. After stirring overnight, the product mixture was quenched with 100 mL H_2O and acidified to pH 1 with 2 M HCl. The organic phase was then extracted with EtOAc (3 × 150 mL), dried over Na_2SO_4 and concentrated under *vacuo* until ~10% solvent remained. The contents were then filtered under *vacuo* and washed with ice-cold EtOAc. The white solid obtained was then dried under *vacuo*.

Yield: 12.3 g (85%, 42.5 mmol)

^{1}H NMR (400 MHz, DMSO-d_6): δ 13.23 (br.s, 1H, CO_2*H*), 7.34 – 7.29 (m, 6H, Ar*H*), 7.28 – 7.23 (m, 3H, Ar*H*), 7.15 – 7.11 (m, 6H, Ar*H*).

^{13}C NMR (101 MHz, DMSO-d_6): δ 174.7 (*C*O_2H), 143.7 (C_{Ar}), 130.4 (CH_{Ar}), 128.1 (CH_{Ar}), 127.1 (CH_{Ar}), 67.3 (Ph_3C).

The ^{1}H and ^{13}C NMR resonances are consistent with those previously reported.[2]

2.2. General procedure 1 for the preparation of triphenylacetate salts

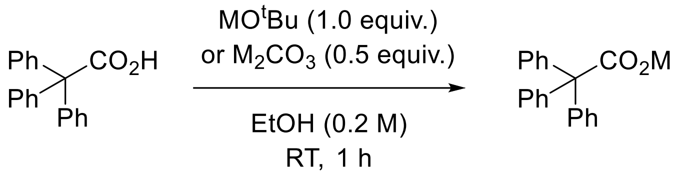

Adapted from the works of Perry et. al.[2] A 250 mL RBF equipped with a stir bar was charged with Ph_3CCO_2H (1.0 equiv.) and EtOH (0.4 M). In a separate flask, MO*t*-Bu (1.0 equiv.) or M_2CO_3 (0.5 equiv.) was dissolved in EtOH (0.4 M). The MO*t*-Bu/M_2CO_3 solution was slowly added to the solution containing Ph_3CCO_2H and the MO*t*-Bu/M_2CO_3 flask was rinsed with small portions of EtOH to ensure all the MO*t*-Bu/M_2CO_3 was transferred. The resulting mixture was stirred at room temperature for 1 h. The solution was then concentrated under *vacuo* until ~25% of the solvent remained. The remaining mixture was cooled in an ice bath then filtered under *vacuo* and washed with cold Et_2O. The white solid obtained was then dried under *vacuo* overnight at 40 °C.

Potassium 2,2,2-triphenylacetate (2-K)

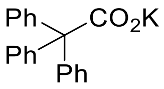

Following general procedure 1 with Ph_3CCO_2H (13.0 g, 45 mmol, 1.00 equiv.), KO *t*-Bu (5.15 g, 45 mmol, 1.00 equiv.) and EtOH (225 mL, 0.2 M). Product was isolated as a white solid.

Yield: 14.3 g (97%, 43.7 mmol)

^{1}H NMR (400 MHz, D_2O): δ 7.28 – 7.16 (m, 15H, Ar*H*).

^{13}C NMR (101 MHz, D_2O): δ 180.3 (*C*O_2K), 145.5 (C_{Ar}), 130.3 (CH_{Ar}) 127.7 (CH_{Ar}) 126.2 (CH_{Ar}), 70.3 (Ph_3*C*).

Elemental Analysis: Calcd. For $C_{20}H_{15}KO_2$: C, 73.59; H, 4.63. Found: C, 73.70; H, 4.68.

The ^{1}H and ^{13}C NMR resonances are consistent with those previously reported.[2]

Lithium 2,2,2-triphenyl acetate (2-Li)

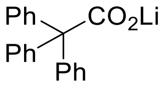

Following general procedure 1 with Ph_3CCO_2H (228 mg, 0.79 mmol, 1.00 equiv.), LiO*t*-Bu (63 mg, 0.79 mmol, 1.00 equiv.) and EtOH (3.0 mL, 0.2 M). The product was obtained as a white solid after drying under vacuum at 100 °C for 16 h.

Yield: 188 mg (81%, 0.64 mmol)

^{1}H NMR (400 MHz, D_2O): δ 7.35 – 7.25 (m, 15H, Ar*H*).

^{13}C NMR (101 MHz, D_2O): δ 180.3 (*C*O_2Li), 145.5 (C_{Ar}), 130.3 (CH_{Ar}), 127.7 (CH_{Ar}), 126.2 (CH_{Ar}), 70.3 (Ph_3C).

Elemental Analysis: Calcd. For $C_{20}H_{15}LiO_2$: C, 81.63; H, 5.14. Found C, 79.52; H, 5.12. Although these results are outside the range viewed as establishing analytical purity (±0.4%), they are provided to illustrate the best values obtained to date. A discussion on the implications of the purity is provided in section 3.1.4.

The ^{1}H and ^{13}C NMR resonances are consistent with those previously reported.[2]

Sodium 2,2,2-triphenyl acetate (2-Na)

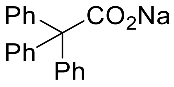

Following general procedure 1 with Ph_3CCO_2H (1.44 g, 5.0 mmol, 1.0 equiv.), NaO*t*-Bu (481 mg, 5.0 mmol, 1.0 equiv.) and EtOH (25 mL, 0.2 M). The product was obtained as a white solid after drying under vacuum at 100 °C for 16 h.

Yield: 1.49 g (96%, 4.8 mmol)

^{1}H NMR (400 MHz, D_2O): δ 7.35 – 7.26 (m, 15H, Ar*H*).

^{13}C NMR (101 MHz, D_2O): δ 180.3 (*C*O_2Na), 145.5 (C_{Ar}), 130.3 (CH_{Ar}), 127.7 (CH_{Ar}), 126.2 (CH_{Ar}), 70.3 (Ph_3C).

Elemental Analysis: Calcd. For $C_{20}H_{15}NaO_2$: C, 77.41; H, 4.87. Found: C, 76.11; H, 5.07. Although these results are outside the range viewed as establishing analytical

purity (±0.4%), they are provided to illustrate the best values obtained to date. A discussion on the implications of the purity is provided in section 3.1.4.

The ^{1}H and ^{13}C NMR resonances are consistent with those previously reported.[2]

Rubidium 2,2,2-triphenyl acetate (2-Rb)

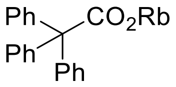

Following general procedure 1 with Ph_3CCO_2H (2.88 g, 10 mmol, 1.0 equiv.), Rb_2CO_3 (1.16 g, 5.0 mmol, 0.50 equiv.) and EtOH (50 mL, 0.2 M). Product was isolated as a white solid.

Yield: 2.77 g (74%, 7.4 mmol).

^{1}H NMR (400 MHz, D_2O): δ 7.36 – 7.26 (m, 15H, Ar*H*).

^{13}C NMR (101 MHz, D_2O): δ 180.3 (CO_2Rb), 145.5 (C_{Ar}), 130.3 (CH_{Ar}), 127.7 (CH_{Ar}), 126.2 (CH_{Ar}), 70.3 (Ph_3C).

Elemental Analysis: Calcd. For $C_{20}H_{15}RbO_2$: C, 64.44; H, 4.06. Found C, 64.07; H, 4.19.

Cesium 2,2,2-triphenyl acetate (2-Cs)

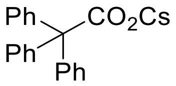

Following general procedure 1 with Ph_3CCO_2H (1.44 g, 5.0 mmol, 1.0 equiv.), Cs_2CO_3 (815 mg, 2.50 mmol, 0.50 equiv.) and MeOH (25 mL, 0.2 M). Product was isolated as a white solid.

Yield: 2.02 g (96%, 4.81 mmol).

^{1}H NMR (500 MHz, D_2O): 7.36 – 7.27 (m, 15H, Ar*H*).

^{13}C NMR (126 MHz, D_2O):δ 180.3 (CO_2Cs) 145.5 (C_{Ar}), 130.3 (CH_{Ar}), 127.7 (CH_{Ar}), 126.2 (CH_{Ar}), 70.3 (Ph_3C).

Elemental Analysis: Calcd. For $C_{20}H_{15}CsO_2$: C, 57.16; H, 3.60. Found C, 57.02; H, 3.57.

The ^1H and ^{13}C NMR resonances are consistent with those previously reported.[2]

3. Optimization Studies

3.1. General procedure for optimisation studies

A Biotage® microwave vial was equipped with a magnetic stir bar and septum and dried with a heat-gun under vacuum. Upon cooling, the vial was then charged with aryl halide (0.50 mmol, 1.00 equiv.) and the appropriate carboxylating agent (1.00 mmol, 2.00 equiv.) and placed under a nitrogen atmosphere through cycles of vacuum/nitrogen gas. Solvent (2.5 mL, 0.2 M) was then added to the vial. Under a flow of nitrogen gas, the septum was replaced with a crimp cap and firmly sealed using a Biotage® crimper. The reaction mixture was stirred at the given temperature (100, 50 or 30 °C) for the given time (between 1 minute and 16 h). After this time, methyl iodide (250 µL, 4.00 mmol, 8.00 equiv.) was added and the mixture was stirred at 50 °C for 2 h. The reaction was cooled to room temperature and quenched with 10 mL aqueous $NaHCO_3$ (sat.). The reaction mixture was extracted with Et_2O (3 × 10 mL), dried over Na_2SO_4, and then concentrated under *vacuo*. The reaction mixture was analysed using quantitative ^{1}H NMR, using 1,1,2,2-tetrachloroethane as an internal standard.

3.1.1 Reaction Duration

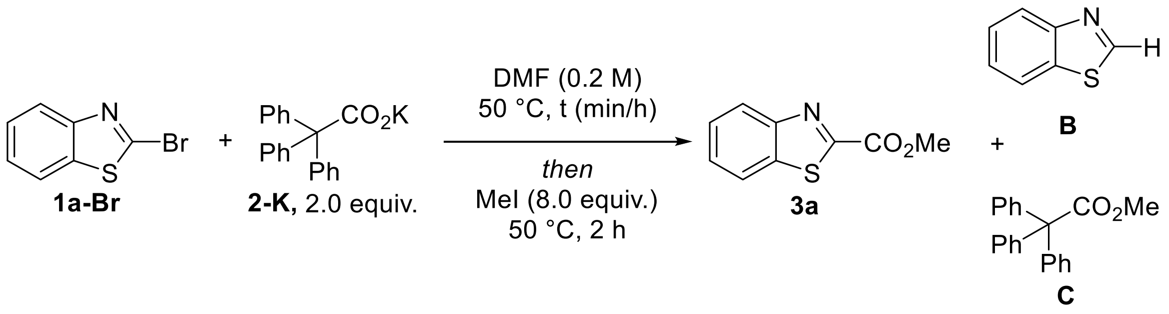

Entry	t (min/h)	3a (%)	B (%)	C (%)[a]	1a (%)
**1.**	16 h	96	4	2	0
**2.**	30 min	96	4	4	0
**3.**	5 min	91	6	17	0
**4.**	1 min	Trace	4	89	92

[a] Recovery w.r.t **2**.

3.1.2. Solvent Screening

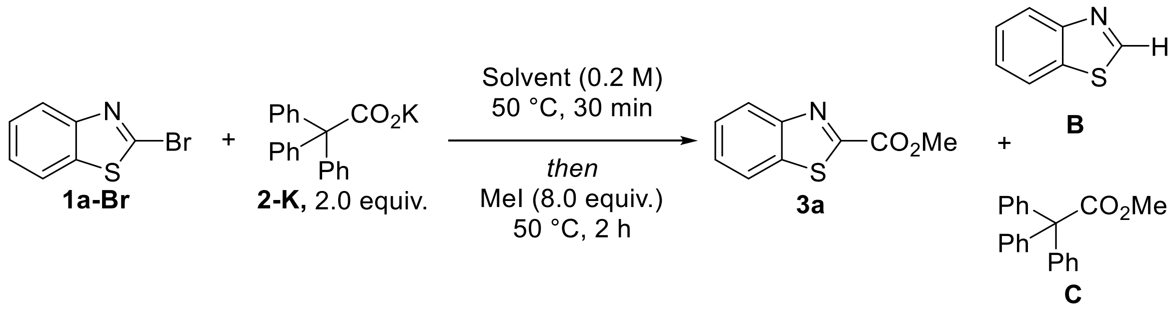

Entry	Solvent	3a (%)	B (%)	C (%)[a]	1a (%)
**1.**	DMF	96	4	4	0
**2.**	DMSO	73	7	2	0
**3.**	DMA	80	5	3	0
**4.**	THF	0	0	18	99
**5.**	PhMe	0	0	0	98

[a] Recovery w.r.t **2**.

3.1.3. Introduction of H_2O/open to air reaction

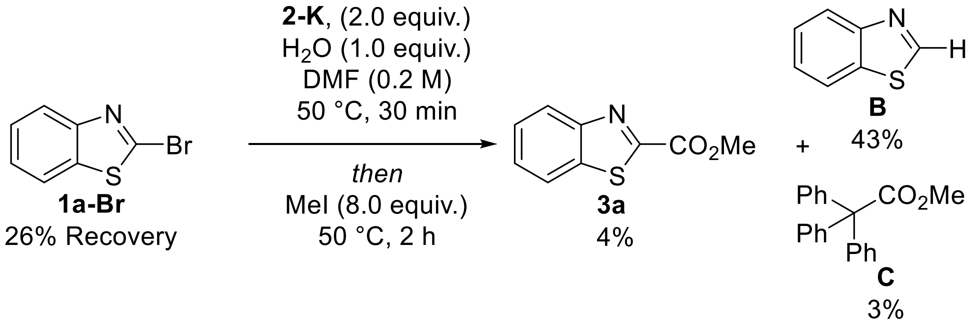

The addition of stoichiometric quantities of water resulted in almost complete shutdown of the optimised reaction. The major product observed was **B**.

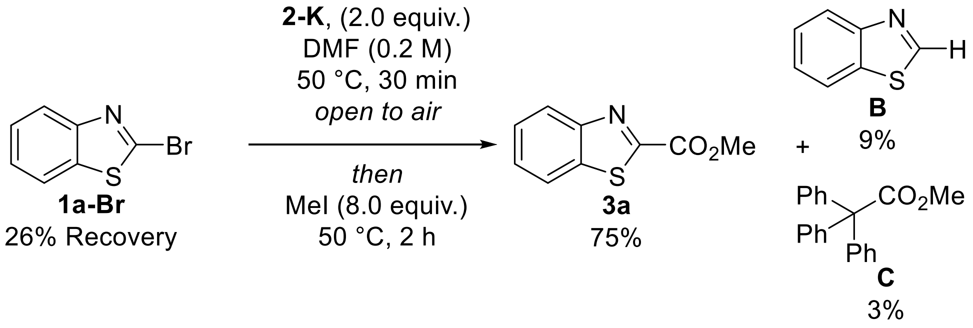

In this experiment, the vial was not capped allowing the mixture to be exposed to air during the reaction. The yield dropped slightly, but good reactivity was still observed.

3.1.4. Metal Screening

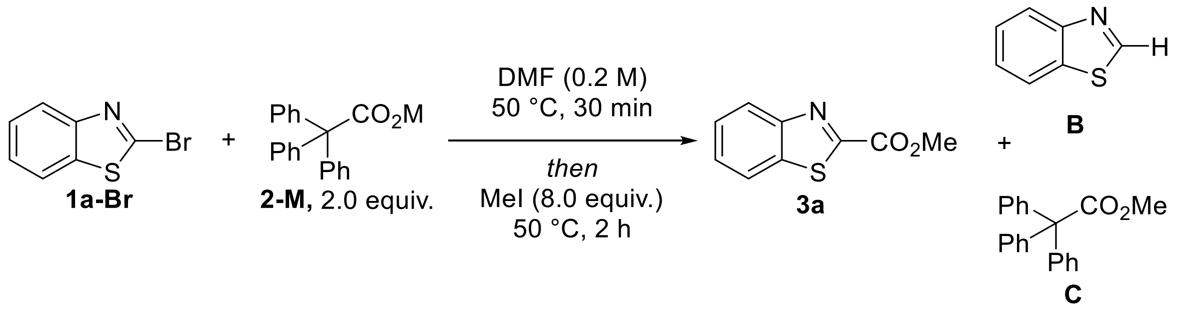

Entry	M	3a (%)	B (%)	C (%)[a]	1a (%)
**1.**	**Li**	37	11	37	30
**2.**	**Na**	63	12	18	10
**3.**	**K**	96	4	0	0
**4.**	**Rb**	93	6	2	2
**5.**	**Cs**	85[b]	8	0	2

[a] Recovery w.r.t **2.** [b] Stirred for 1 h.

The results above show that the potassium salt Ph_3CCO_2K was the best carboxylating agent for this process. The rubidium and cesium salts also showed good reactivity, however, the lithium and sodium salts were less effective. Elemental analysis revealed that the potassium, rubidium and caesium salts were of high purity (the found values were within 0.4% of the calculated values). However, the elemental analysis values for the lithium and sodium salts were outside the range viewed as establishing analytical purity (±0.4%). We believe that the lithium and sodium salts are more hygroscopic than the other salts. This causes water to be present in the reaction, which shuts down reactivity. We attempted to dry the salts by heating at 100 °C under vacuum for 16 h, but the elemental analysis values still suggested that some water remained. Overall, we opted to abandon the lithium and sodium salts and continue with the more practical potassium salt. We believe that the lithium and sodium salts can promote the carboxylation, but their hygroscopicity currently makes their use impractical.

For example, the difference in yields between the batches of sodium salts (Ph_3CCO_2Na, **2-Na**) supports our suggestion that hygroscopicity is a major factor for the lower reactivity. The elemental analysis for the 2 batches of the sodium salt that we prepared are provided below. Batch 1 was calculated to contain ~0.3 equivalents of water, whereas Batch 2 was calculated to contain ~0.6 equivalents of water (Table

2). Under the standard reaction conditions, Batch 1, which contained less water, gave a higher yield (63%) than Batch 2 (53%).

Elemental Analysis for Batch 1:

Calcd. for $C_{20}H_{15}NaO_2$: C, 77.41; H, 4.87.

Calcd. for $C_{20}H_{15}NaO_2$•0.3(H_2O): C, 76.08; H, 4.98

Found: C, 76.11; H, 5.07.

Elemental Analysis for Batch 2:

Calcd. for $C_{20}H_{15}NaO_2$: C, 77.41; H, 4.87.

Calcd. for $C_{20}H_{15}NaO_2$•0.6(H_2O): C, 74.80; H, 5.08

Found: C, 74.78; H, 5.00.

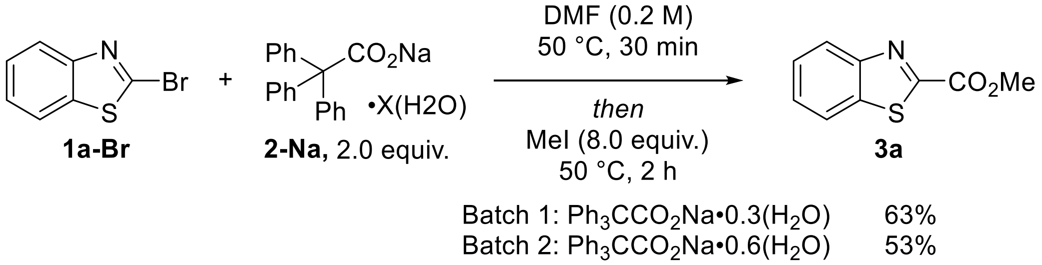

3.1.5. Comparing the reactivity of C–H carboxylation and C–X carboxylation

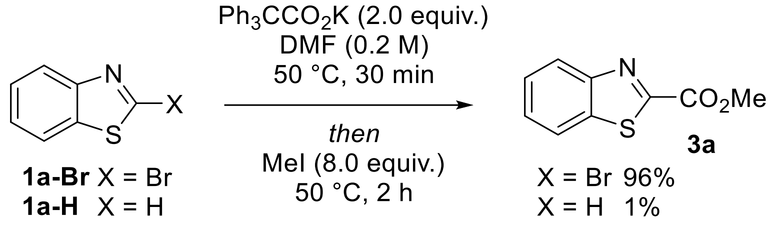

These results illustrate that the C–X carboxylation of **1a-Br** (X = Br) is much faster than the C–H carboxylation of **1a-H** (X = H).

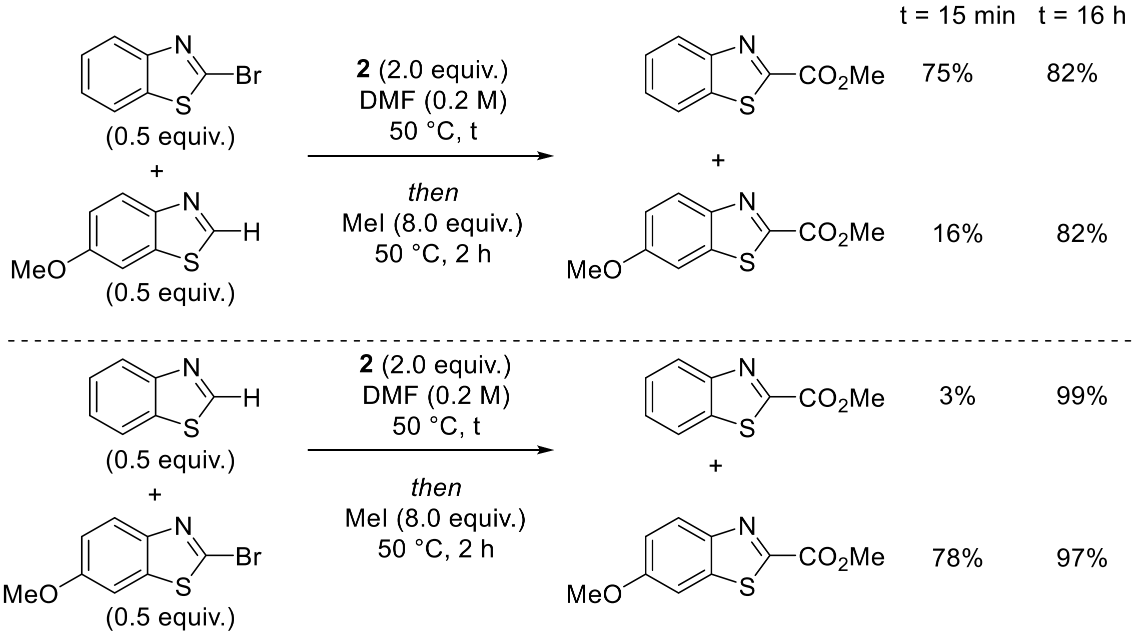

These results further illustrate that the C–X carboxylation is faster than the C–H carboxylation. In both experiments the 2-bromobenzothiazole **1-Br** reacted faster than the benzothiazole **1-H** when an excess of Ph_3CCO_2K was used.

4. The scope of the C-X carboxylation of aryl halides

4.1. General procedure 2 for the carboxylation of aryl halides

A Biotage® microwave vial was equipped with a magnetic stir bar and septum and dried with a heat-gun under *vacuo*. The vial was then charged with aryl halide (1.00 mmol, 1.00 equiv.) and Ph_3CCO_2K (669 mg, 2.05 mmol, 2.05 equiv.) and placed under a nitrogen atmosphere through cycles of vacuum/nitrogen gas. DMF (5.0 mL, 0.2 M) was then added to the vial. Under an N_2 gas flow, the septum was replaced with a Biotage® crimp cap. The reaction mixture was stirred at the given temperature (50 or 60 °C) for 30 mins. After this time, $NaHCO_3$ (252 mg, 3.00 mmol, 3.00 equiv) was added and stirred for a further 2 min. The reaction was cooled to room temperature and quenched with 20 mL H_2O and washed with either DCM or Et_2O (3 × 20 mL) to remove the majority of the $Ph_3CCO_2CPh_3$ side product. The aqueous phase, which contained the product as the carboxylate salt, was collected and concentrated under *vacuo* and dried overnight. The solid obtained was dispersed in DMF (5.0 mL, 0.2 M) and methyl iodide (500 µL, 8.00 mmol, 8.00 equiv) was added. The reaction was stirred at 50 °C for a further 2 h and then cooled to room temperature. The reaction was quenched with saturated $NaHCO_3$ (aq., 20 mL) and extracted with Et_2O (3 × 20 mL). The organic phase was dried over Na_2SO_4, purified under *vacuo* and purified using silica gel column chromatography.

Methyl benzo[*d*]thiazole-2-carboxylate (3a)

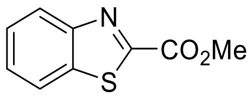

Following general procedure 2 with 2-bromobenzothiazole (214 mg, 1.00 mmol, 1.00 equiv.). Product obtained as an off-white solid. Column eluent: 0% to 30% Et_2O in hexane.

Yield: 176 mg (91%, 0.91 mmol).

R_f: 0.22 (4:1, Hex:Et_2O)

^{1}H NMR (400 MHz, $CDCl_3$):δ 8.25 – 8.23 (m, 1H, Ar*H*), 7.99 – 7.97 (m, 1H, Ar*H*), 7.61 – 7.52 (m, 2H, Ar*H*), 4.08 (s, 3H, CO_2CH_3).

^{13}C NMR (101 MHz, $CDCl_3$):δ 161.1 (CO_2Me), 158.0 (C_{Ar}), 153.2 (C_{Ar}), 136.8 (C_{Ar}), 127.6 (CH_{Ar}), 127.2 (CH_{Ar}), 125.5 (CH_{Ar}), 122.1 (CH_{Ar}), 53.7 (CO_2CH_3).

The ^{1}H and ^{13}C NMR resonances are consistent with those previously reported.[2]

Methyl 6-Methylbenzo[*d*]thiazole-2-carboxylate (3b)

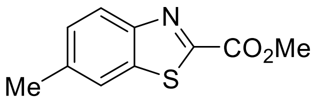

Following general procedure 2 with 2-bromo-6-methylbenzothiazole (228 mg, 1.00 mmol, 1.00 equiv.). Product obtained as an off-white solid. Column eluent: 0% to 50% Et_2O in hexane.

Yield: 189 mg (91%, 0.91 mmol).

R_f: 0.30 (3:2 Hex:Et_2O)

^{1}H NMR (400 MHz, $CDCl_3$):δ 8.08 (d, 1H, J = 8.4 Hz, ArH), 7.73 (d, 1H, J = 1.0 Hz, ArH), 7.37 (dd, 1H, J = 8.4, 1.0 Hz, ArH), 4.05 (s, 3H, CO_2CH_3), 2.50 (s, 3H, CH_3).

^{13}C NMR (101 MHz, $CDCl_3$):δ 161.2 (CO_2Me), 156.9 (C_{Ar}), 151.3 (C_{Ar}), 138.3 (C_{Ar}), 137.1 (C_{Ar}), 129.0 (CH_{Ar}), 125.0 (CH_{Ar}), 121.6 (CH_{Ar}), 53.5 (CO_2CH_3), 21.8 (CH_3).

The ^{1}H and ^{13}C NMR resonances are consistent with those previously reported.[2]

Methyl 6-Methoxybenzo[*d*]thiazole-2-carboxylate (3c)

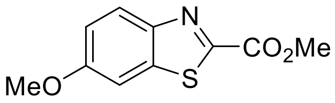

Following general procedure 2 with 2-bromo-6-methoxybenzothiazole (244 mg, 1.00 mmol, 1.00 equiv.). Product obtained as an off-white solid. Column eluent: 0% to 50% Et_2O in hexane.

Yield: 190 mg (85%, 0.85 mmol)

R_f: 0.22 (3:2 Hex:Et_2O)

^{1}H NMR (400 MHz, $CDCl_3$): δ 8.11 (d, 1H, *J* = 9.2 Hz, Ar*H*), 7.37 (d, 1H, *J* = 2.5 Hz, Ar*H*), 7.18 (dd, 1H, *J* = 9.2, 2.5 Hz, Ar*H*), 4.07 (s, 3H, CO_2CH_3), 3.91 (s, 3H, OCH_3).

^{13}C NMR (101 MHz, $CDCl_3$): δ 161.2 (CO_2Me), 159.7 (C_{Ar}), 155.2 (C_{Ar}), 147.8 (C_{Ar}), 138.7 (C_{Ar}), 126.2 (CH_{Ar}), 117.8 (CH_{Ar}), 103.4 (CH_{Ar}), 55.9 (OCH_3), 53.5 (CO_2CH_3).

The ^{1}H and ^{13}C NMR resonances are consistent with those previously reported.[2]

Methyl 6-Fluorobenzo[*d*]thiazole-2-carboxylate (3d)

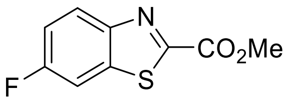

Following general procedure 2 with 2-bromo-6-fluorobenzothiazole (116 mg, 0.50 mmol, 1.00 equiv.), Ph_3CCO_2K (335 mg, 1.03 mmol, 2.05 equiv.) and DMF (5.0 mL, 0.1 M). The work-up was conducted using $NaHCO_3$ (126 mg, 1.50 mmol, 3.00 equiv.), methyl iodide (250 µL, 4.00 mmol, 4.00 equiv) and DMF (2.5 mL, 0.2 M). The product was isolated as a white solid. Column chromatography was not required.

Yield: 80 mg (76%, 0.38 mmol)

R_f: 0.19 (4:1 Hex:Et_2O)

^{1}H NMR (400 MHz, $CDCl_3$) δ: 8.20 (ddd, 1H, *J* = 9.1, 4.8, 0.4 Hz, Ar*H*), 7.65 (ddd, 1H, *J* = 8.0, 2.6, 0.4 Hz, Ar*H*), 7.33 (td, 1H, *J* = 8.9, 2.6 Hz, Ar*H*), 4.08 (s, 3H, CO_2CH_3).

^{13}C NMR (101 MHz, $CDCl_3$): δ 162.0 (d, *J* = 250 Hz, CF_{Ar}), 160.8 (CO_2Me), 157.8 (d, *J* = 4.0 Hz, C_{Ar}), 149.9 (s, C_{Ar}), 138.0 (d, *J* = 11.1 Hz, C_{Ar}), 126.9 (d, *J* = 9.1 Hz, CH_{Ar}), 116.6 (d, *J* = 25.3 Hz, CH_{Ar}), 108.1 (d, *J* = 27.3 Hz, CH_{Ar}), 53.7 (s, CO_2CH_3).

^{19}F{^{1}H} NMR (376 MHz, $CDCl_3$)δ: –111.34 (s, 1F, Ar*F*).

HR-MS (ESI$^+$): Found *m/z* 212.0173, $[M + H]^+$; calculated for $[C_9H_7FNO_2S]^+$: 212.0176.

Methyl 6-Chlorobenzo[*d*]thiazole-2-carboxylate (3e)

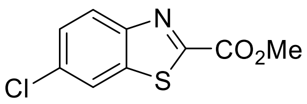

Following general procedure 2 with (procedure 2) 2-bromo-6-chlorobenzothiazole (249 mg, 1.00 mmol, 1.00 equiv.). After the reaction had completed, methyl iodide (500 µL, 8.00 mmol, 8.00 equiv) was added and the mixture was allowed to stir at 50 °C for a further 2h. Upon completion, the reaction mixture was cooled to room temperature, quenched with 20 mL $NaHCO_3$ (sat.) and extracted with Et_2O (3 × 20 mL). The organic phase was then dried over Na_2SO_4, concentrated under *vacuo* and purified using silica gel column chromatography. The product was obtained as an off white solid. Column eluent: 0% to 40% Et_2O in hexane.

Yield: 168 mg (74%, 0.74 mmol).

R_f: 0.20 (4:1 Hex:Et_2O)

^{1}H NMR (400 MHz, $CDCl_3$):δ 8.13 (dd, 1H, *J* = 8.7, 0.3 Hz, Ar*H*), 7.95 (dd, 1H, *J* = 2.1, 0.3 Hz, Ar*H*), 7.53 (dd, 1H, *J* = 8.7, 2.1 Hz, Ar*H*), 4.08 (s, 3H, CO_2CH_3).

^{13}C NMR (101 MHz, $CDCl_3$):δ 160.8 (CO_2Me), 158.5 (C_{Ar}), 151.6 (C_{Ar}), 137.9 (C_{Ar}), 134.0 (C_{Ar}), 128.3 (CH_{Ar}), 126.3 (CH_{Ar}), 121.7 (CH_{Ar}), 53.8 (CO_2CH_3).

The ^{1}H and ^{13}C NMR resonances are consistent with those previously reported.[3]

Methyl 6-Bromobenzo[*d*]thiazole-2-carboxylate (3f)

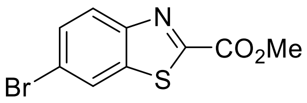

Following general procedure 2 with 2,6-bromobenzothiazole (146 mg, 0.50 mmol, 1.00 equiv.), Ph_3CCO_2K (335 mg, 1.03 mmol, 2.05 equiv.) and DMF (5.0 mL, 0.1 M). Ph_3CCO_2K (335 mg, 1.03 mmol, 2.05 equiv.) and DMF (5.0 mL, 0.1 M). The work-up was conducted using $NaHCO_3$ (126 mg, 1.50 mmol, 3.00 equiv.), methyl iodide (250 µL, 4.00 mmol, 4.00 equiv) and DMF (2.5 mL, 0.2 M). The product was isolated as a white solid. Column eluent: 0% to 30% Et_2O in hexane.

Yield: 78 mg (57%, 0.29 mmol)

R$_f$: 0.22 (4:1 Hex:Et_2O)

^{1}H NMR (400 MHz, $CDCl_3$)δ: 8.12 (dd, 1H, *J* = 2.0, 0.5 Hz, Ar*H*), 8.08 (dd, 1H, *J* = 8.8, 0.5 Hz, Ar*H*), 7.68 (dd, 1H, *J* = 8.8, 2.0 Hz, Ar*H*), 4.08 (s, 3H, CO_2CH_3)

^{13}C NMR (101 MHz, $CDCl_3$)δ: 160.7 (CO_2Me), 158.5 (C_{Ar}), 151.9 (C_{Ar}), 138.3 (C_{Ar}), 131.0 (CH_{Ar}), 126.6 (CH_{Ar}), 124.7 (CH_{Ar}), 121.9 (C_{Ar}), 53.8 (OCH_3).

The ^{1}H and ^{13}C NMR resonances are consistent with those previously reported.[4]

Dimethyl benzothiazole-2,6-dicarboxylate (3g)

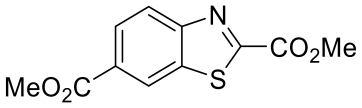

Following general procedure 2 with methyl 2-bromobenzo[*d*]thiazole-6-carboxylate (69 mg, 0.25 mmol, 1.00 equiv.), Ph_3CCO_2K (167 mg, 0.51 mmol, 2.05 equiv.) and DMF (5.0 mL, 0.05 M). The work-up was conducted using $NaHCO_3$ (63.0 mg, 0.75 mmol, 3.00 equiv.), methyl iodide (250 μL, 4.00 mmol, 4.00 equiv) and DMF (1.25 mL, 0.2 M). The product was obtained as an off-white solid. Column eluent: 0% to 20% EtOAc in hexane.

Yield: 35 mg (55%, 0.14 mmol)

R$_f$: 0.37 (Hex:EtOAc 3:1)

^{1}H NMR (400 MHz, $CDCl_3$):δ 8.71 (dd, 1H, *J* = 1.5, 0.7 Hz, Ar*H*), 8.28 – 8.22 (m, 2H, Ar*H*), 4.10 (s, 3H, CO_2CH_3), 3.98 (s, 3H, CO_2CH_3).

^{13}C NMR (101 MHz, $CDCl_3$):δ 166.1 (CO_2CH_3), 161.2 (CO_2CH_3), 160.7 (C_{Ar}), 155.7 (C_{Ar}), 136.6 (C_{Ar}), 129.3 (C_{Ar}), 128.0 (CH_{Ar}), 125.3 (CH_{Ar}), 124.5 (CH_{Ar}), 53.9 (CO_2CH_3), 52.6 (CO_2CH_3).

HR-MS (ESI$^+$): Found *m/z* 252.0321, $[M + H]^+$; calculated for $[C_{11}H_{10}NO_4S]^+$: 252.0325.

6-Ethyl 2-Methyl Benzothiazole-2,6-dicarboxylate (3h)

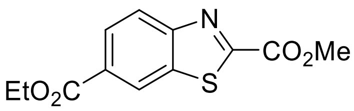

Following general procedure 2 with ethyl 2-bromobenzo[*d*]thiazole-6-carboxylate (143 mg, 0.50 mmol, 1.00 equiv.), Ph_3CCO_2K (335 mg, 1.03 mmol, 2.05 equiv.) and DMF (5.0 mL, 0.1 M). The work-up was conducted using $NaHCO_3$ (126 mg, 1.50 mmol, 3.00 equiv.), methyl iodide (250 µL, 4.00 mmol, 4.00 equiv) and DMF (2.5 mL, 0.2 M). Product was obtained as an off-white solid. Column chromatography was not required for this substrate.

Yield: 109 mg (83%, 0.41 mmol)

R_f: 0.19 (4:1 Hex:Et_2O)

^{1}H NMR (400 MHz, $CDCl_3$):δ 8.70 (dd, 1H, *J* = 1.5, 0.7 Hz, Ar*H*), 8.28 – 8.22 (m, 2H, Ar*H*), 4.44 (q, 2H, *J* = 7.1 Hz, CH_2CH_3), 4.09 (s, 3H, CO_2CH_3), 1.43 (t, 3H, *J* = 7.1 Hz, CH_2CH_3).

^{13}C NMR (101 MHz, $CDCl_3$):δ 165.6 (CO_2Et), 161.1 (C_{Ar}), 160.7 (CO_2Me), 155.7 (C_{Ar}), 136.6 (C_{Ar}), 129.6 (C_{Ar}), 128.1 (CH_{Ar}), 125.2 (CH_{Ar}), 124.4 (CH_{Ar}), 61.6 (CH_2CH_3), 53.9 (CO_2CH_3), 14.3 (CH_2CH_3).

The ^{1}H and ^{13}C NMR resonances are consistent with those previously reported.[2]

Methyl 4-Chlorobenzo[*d*]thiazole-2-carboxylate (3i)

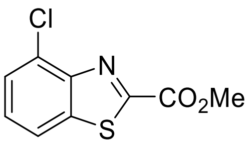

Following general procedure 2 with 2-bromo-4-chlorobenzothiazole (249 mg, 1.00 mmol, 1.00 equiv). Product obtained as a white solid. Column eluent: 0% to 30% Et_2O in hexane.

Yield: 146 mg (64%, 0.64 mmol)

R_f: 0.27 (4:1 Hex:Et_2O)

^{1}H NMR (400 MHz, $CDCl_3$):δ 7.87 (dd, 1H, *J* = 8.0 Hz, 1.0 Hz, Ar*H*), 7.61 (dd, 1H, *J* = 7.8 Hz, 1.0 Hz, Ar*H*), 7.47 (dd, 1H, *J* = 8.0, 7.8 Hz, Ar*H*), 4.08 (s, 1H, CO_2CH_3).

^{13}C NMR (101 MHz, $CDCl_3$):δ 160.9 (CO_2Me), 159.0 (C_{Ar}), 150.5 (C_{Ar}), 138.2 (C_{Ar}), 130.4 (C_{Ar}), 128.1 (CH_{Ar}), 127.3 (CH_{Ar}), 120.6 (CH_{Ar}), 53.8 (CO_2CH_3).

The ^{1}H and ^{13}C NMR resonances are consistent with those previously reported.[4]

Methyl 5-Chlorobenzo[*d*]thiazole-2-carboxylate (3j)

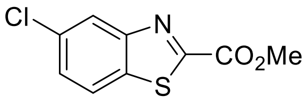

Following general procedure 2 with 2-bromo-5-chlorobenzothiazole (62 mg, 0.25 mmol, 1.00 equiv.), Ph_3CCO_2K (167 mg, 0.51 mmol, 2.05 equiv.) and DMF (5.0 mL, 0.05 M). The work-up was conducted using $NaHCO_3$ (63.0 mg, 0.75 mmol, 3.00 equiv.), methyl iodide (250 μL, 4.00 mmol, 4.00 equiv) and DMF (1.25 mL, 0.2 M). Product was isolated as a pale-yellow solid. Column eluent 0% to 30% Et_2O in hexane.

Yield: 38 mg (67%, 0.17 mmol)

R_f: 0.30 (4:1 Hex:Et_2O)

^{1}H NMR (400 MHz, $CDCl_3$):δ 8.22 (dd, 1H, *J* = 2.1, 0.5 Hz, Ar*H*), 7.91 (dd, 1H, *J* = 8.7, 0.5 Hz, Ar*H*), 7.53 (dd, 1H, *J* = 8.7, 2.1 Hz, Ar*H*), 4.09 (s, 3H, OCH_3)

^{13}C NMR (101 MHz, $CDCl_3$):δ 160.8 (CO_2Me), 159.8 (C_{Ar}), 153.9 (C_{Ar}), 135.0 (C_{Ar}), 133.4 (C_{Ar}), 128.4 (CH_{Ar}), 125.1 (CH_{Ar}), 122.9 (CH_{Ar}), 53.8 (OCH_3).

The ^{1}H and ^{13}C NMR resonances are consistent with those previously reported.[2]

Benzyl 5-formylthiazole-2-carboxylate (3k)

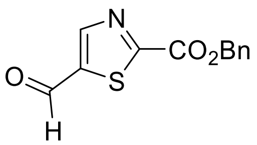

Following general procedure 2 with 2-bromo-5-formylthiazole (96 mg, 0.50 mmol, 1.00 equiv.), Ph_3CCO_2K (335 mg, 1.03 mmol, 2.05 equiv.) and DMF (2.5 mL, 0.2 M).

The work-up was conducted using $NaHCO_3$ (126 mg, 1.50 mmol, 3.00 equiv.), benzyl bromide (70 µL, 0.60 mmol, 1.20 equiv.) and DMF (2.5 mL, 0.2 M). The product was obtained as an orange oil. Column eluent: 0% to 80% Et_2O in hexane.

Yield: 46 mg (37%, 0.19 mmol)

R_f: 0.17 (Hex:Et_2O 1:1)

^{1}H NMR (500 MHz, $CDCl_3$):δ 10.12 (s, 1H, C*H*O), 8.55 (s, 1H, Ar*H*), 7.48 – 7.46 (m, 2H, Ar*H*), 7.42 – 7.35 (m, 3H, Ar*H*), 5.47 (s, 1H, PhCH_2).

^{13}C NMR (125 MHz, $CDCl_3$):δ (1 x C_{Ar} missing), 182.1 (*C*HO), 163.7 (*C*O_2Bn), 159.1 (C_{Ar}), 151.2 (CH_{Ar}), 143.2 (CH_{Ar}), 134.4 (C_{Ar}), 129.0 (CH_{Ar}), 128.8 (CH_{Ar}), 68.8 (Ph*C*H_2).

HR-MS (ESI$^+$): Found *m/z* 270.0201, [M + Na]$^+$; calculated [$C_{12}H_9NO_3SNa$]$^+$: 270.0195.

Methyl 2-phenylthiazole-5-carboxylate (3l)

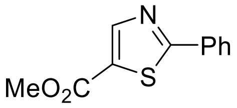

Following general procedure 2 with 5-bromo-2-phenylthiazole (240 mg, 1.00 mmol, 1.00 equiv.). The product was isolated as a white crystalline solid. Column eluent: 0% to 20% Et_2O in hexane.

Yield: 193 mg (88%, 0.88 mmol).

R_f: 0.43 (4:1 Hex:Et_2O).

^{1}H NMR (400 MHz, $CDCl_3$):δ 8.43 (s, 1H, Ar*H*), 8.00 – 7.98 (m, 2H, Ar*H*), 7.49 – 7.48 (m, 3H, Ar*H*), 3.93 (s, 3H, OCH_3).

^{13}C NMR (101 MHz, $CDCl_3$): δ 173.5 (*C*O_2Me), 161.9 (C_{Ar}), 149.4 (CH_{Ar}), 132.9 (C_{Ar}), 131.3 (CH_{Ar}), 129.2 (CH_{Ar}), 128.6 (C_{Ar}), 126.9 (CH_{Ar}), 52.5 (O*C*H_3).

The ^{1}H and ^{13}C NMR resonances are consistent with those previously reported.[5]

Ethyl 1-Methyl-1H-benzo[*d*]imidazole-2-carboxylate (3m)

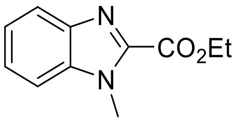

Following general procedure 2 with 2-iodo-1-methyl-1H-benzo[*d*]imidazole (129 mg, 0.50 mmol, 1.00 equiv.), Ph_3CCO_2K (335 mg, 1.03 mmol, 2.05 equiv.) and DMF (2.5 mL, 0.2 M). The work-up was conducted using $NaHCO_3$ (126 mg, 1.50 mmol, 3.00 equiv.), ethyl iodide (45 µL, 0.55 mmol, 1.10 equiv) and DMF (2.5 mL, 0.2 M). The product was isolated as an orange oil. Column eluent: 0% to 100% Et_2O in hexane.

Yield: 50 mg (49%, 0.25 mmol)

R_f: 0.64 (100% Et_2O)

^{1}H NMR (400 MHz, $CDCl_3$) δ: 7.92 – 7.90 (m, 1H, Ar*H*), 7.46 – 7.40 (m, 2H, Ar*H*), 7.38 – 7.34 (m, 1H, Ar*H*), 4.52 (q, 2H, *J* = 7.1 Hz, CH_2CH_3), 4.17 (s, 3H, NCH_3), 1.49 (t, 3H, *J* = 7.1 Hz, CH_2CH_3).

^{13}C NMR (101 MHz, $CDCl_3$) δ: 160.2 (CO_2Me), 141.7 (C_{Ar}), 141.0 (C_{Ar}), 136.7 (C_{Ar}), 125.4 (CH_{Ar}), 123.7 (CH_{Ar}), 122.0 (CH_{Ar}), 110.4 (CH_{Ar}), 62.2 (CH_2CH_3), 32.1 (NCH_3), 14.3 (CH_2CH_3).

The ^{1}H and ^{13}C NMR resonances are consistent with those previously reported.[6]

Benzyl thiazole-2-carboxylate (3n)

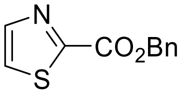

Following general procedure 2 with 2-bromothiazole (164 mg, 1.00 mmol,1.00 equiv.) and BnBr (143 µL, 1.20 mmol, 1.20 equiv.). The product was obtained as a yellow solid. Column eluent: 0% to 80% Et_2O in hexane.

Yield: 167 mg (76%, 0.76 mmol)

R_f: 0.26 (3:2 Hex:Et_2O)

^{1}H NMR (500 MHz, $CDCl_3$):δ 8.03 (d, 1H, *J* = 3.1 Hz, Ar*H*), 7.63 (d, 1H, *J* = 3.1 Hz, Ar*H*), 7.49 – 7.47 (m, 2H, Ar*H*), 7.41 – 7.33 (m, 3H, Ar*H*), 5.45 (s, 2H, PhCH_2).

^{13}C NMR (101 MHz, $CDCl_3$)δ: 159.9 (CO_2Bn), 158.2 (C_{Ar}), 145.1 (CH_{Ar}), 135.0 (C_{Ar}), 128.7 (CH_{Ar}), 128.7 (CH_{Ar}), 128.7 (CH_{Ar}), 125.4 (CH_{Ar}), 68.1 (PhCH_2).

The ^{1}H and ^{13}C NMR resonances are consistent with those previously reported.[7]

Benzyl Thiazole-5-carboxylate (3o)

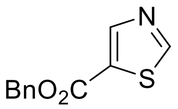

Following general procedure 2 with 5-bromothiazole (164 mg, 1.00 mmol,1.00 equiv.) and BnBr (143 µL, 1.20 mmol, 1.20 equiv.). The product was obtained as a yellow oil. Column eluent: 0% to 100% Et_2O in hexane.

Yield: 147 mg (67%, 0.67 mmol)

R_f: 0.29 (Hex:Et_2O 3:2)

^{1}H NMR (400 MHz, $CDCl_3$):δ 8.94 (s, 1H, Ar*H*), 8.53 (d, 1H, *J* = 0.6 Hz, Ar*H*), 7.45-7.33 (m, 5H, Ar*H*), 5.36 (s, 2H, PhCH_2).

^{13}C NMR (101 MHz, $CDCl_3$): δ 161.1 (CO_2Bn), 158.2 (CH_{Ar}), 149.1 (CH_{Ar}), 135.3 (C_{Ar}), 129.6 (C_{Ar}), 128.7 (CH_{Ar}), 128.6 (CH_{Ar}), 128.4 (CH_{Ar}), 67.4 (PhCH_2).

HR-MS (ESI^+): Found *m/z* 220.0426, $[M + H]^+$; calculated for $[C_{11}H_{10}NO_2S]^+$: 220.0427.

Methyl 2-morpholinothiazole-5-carboxylate (3p)

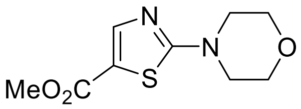

Following general procedure 2 with 2-bromo-5-morpholinothiazole (249 mg, 1.00 mmol, 1.00 equiv.) at 100 °C. The product was isolated as a yellow solid. Column eluent: 0% to 100% Et_2O in hexane.

Yield: 169 mg (74%, 0.74 mmol).

R_f: 0.18 (1:1 Hex:Et_2O)

^{1}H NMR (400 MHz, $CDCl_3$):δ 7.88 (s, 1H, Ar*H*), 3.83 – 3.80 (m, 7H, CO_2CH_3 + OCH_2), 3.56 – 3.54 (m, 4H, NCH_2).

^{13}C NMR (101 MHz, $CDCl_3$):δ 174.9 (CO_2Me), 162.4 (C_{Ar}), 148.0 (CH_{Ar}), 116.8 (C_{Ar}), 66.0 (OCH_2), 51.9 (OCH_3), 48.2 (NCH_2).

HR-MS (ESI$^+$): Found *m/z* 229.0636, $[M + H]^+$; calculated for $[C_9H_{13}N_2O_3S]^+$: 229.0641.

Methyl 5-phenyl-1,3,4-oxadiazole-2-carboxylate (3q)

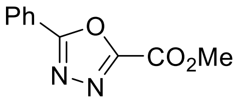

Following general procedure 2 with 2-bromo-5-phenyl-1,3,4-oxadiazole (225 mg, 1.00 mmol, 1.00 equiv.). The product was obtained as a white solid. Column eluent 0% to 50% Et_2O in hexane.

Yield: 139 mg (68%, 0.68 mmol)

R_f: 0.33 (Hex:Et_2O 3:2)

^{1}H NMR (400 MHz, $CDCl_3$): δ 8.19 – 8.16 (m, 2H, Ar*H*), 7.64 – 7.59 (m, 1H, Ar*H*), 7.58 – 7.53 (m, 2H, Ar*H*), 4.09 (s, 3H, CO_2CH_3).

^{13}C NMR (101 MHz, $CDCl_3$): δ 166.6 (C_{Ar}), 156.3 (C_{Ar}), 154.9 (CO_2Me), 132.9 (CH_{Ar}), 129.3 (CH_{Ar}), 127.7 (CH_{Ar}), 122.7 (C_{Ar}), 53.8 (CO_2Me).

The ^{1}H and ^{13}C NMR resonances are consistent with those previously reported.[2]

Methyl Benzo[*b*]thiophene-2-carboxylate (3t)

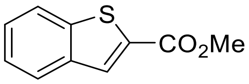

Following general procedure 2 with 2-bromobenzothiopene (213 mg, 1.0 mmol, 1.00 equiv.) at 100 °C. The product was isolated as an orange solid. Column eluent: 0% to 30% Et_2O in hexane.

Yield: 167 mg (87%, 0.87 mmol)

The reaction was also performed following general procedure 2 with 2-iodobenzothiophene at 80 °C. A yield of 76% was observed by quantitative ^{1}H NMR using 1,1,2,2-tetrachloroethane as an internal standard.

R_f: 0.56 (4:1 Hex:Et_2O)

^{1}H NMR (400 MHz, $CDCl_3$): δ 8.07 (s, 1H, Ar*H*), 7.89 – 7.86 (m, 2H, Ar*H*), 7.48 – 7.39 (m, 2H, Ar*H*), 3.95 (s, 3H, OCH_3).

^{13}C NMR (101 MHz, $CDCl_3$): δ 163.3 (*C*O_2Me), 142.2 (C_{Ar}), 138.7 (C_{Ar}), 133.3 (C_{Ar}), 130.6 (CH_{Ar}), 127.0 (CH_{Ar}), 125.6 (CH_{Ar}), 124.9 (CH_{Ar}), 122.8 (CH_{Ar}), 52.5 (OCH_3).

The ^{1}H and ^{13}C NMR resonances are consistent with those previously reported.[2]

Methyl Benzofuran-2-carboxylate (3u)

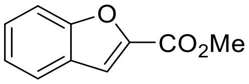

Following general procedure 2 with 2-iodobenzofuran (62.0 mg, 0.25 mmol, 1.00 equiv.), Ph_3CCO_2K (170 mg, 0.52 mmol, 2.05 equiv.) and DMF (1.25 mL, 0.2 M) at 120 °C. The work-up was conducted using $NaHCO_3$ (63.0 mg, 0.75 mmol, 3.00 equiv.), methyl iodide (250 µL, 4.00 mmol, 4.00 equiv) and DMF (1.25 mL, 0.2 M). The product was isolated as a yellow solid. Column eluent: 0% to 20% Et_2O in hexane.

Yield: 27 mg (62%, 0.16 mmol)

The reaction was also performed following general procedure 2 with 2-bromobenzofuran at 140 °C. A yield of 82% was observed by quantitative ^{1}H NMR using 1,1,2,2-tetrachloroethane as an internal standard.

R_f: 0.52 (Hex:Et_2O 4:1)

^{1}H NMR (400 MHz, $CDCl_3$):δ 7.69 (ddd, 1H, *J* = 7.9, 1.2, 0.7 Hz, Ar*H*), 7.59 (ddd, 1H, *J* = 8.4, 1.8, 0.9 Hz, Ar*H*), 7.54 (d, 1H, *J* = 1.0 Hz, Ar*H*), 7.46 (ddd, 1H, *J* = 8.4, 7.2, 1.3 Hz, Ar*H*), 7.31 (ddd, 1H, *J* = 7.9, 7.2, 0.9 Hz, Ar*H*), 3.98 (s, 3H, CO_2CH_3).

^{13}C NMR (101 MHz, $CDCl_3$):δ 159.0 (CO_2Me), 154.7 (C_{Ar}), 144.4 (C_{Ar}), 126.7 (CH_{Ar}), 125.9 (C_{Ar}), 122.8 (CH_{Ar}), 121.8 (CH_{Ar}), 113.0 (CH_{Ar}), 111.4 (CH_{Ar}), 51.4 (CO_2CH_3).

The ^{1}H and ^{13}C NMR resonances are consistent with those previously reported.[2]

Ethyl tetrafluoroisonicotinate (3v)

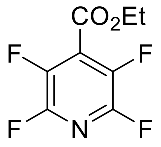

Following general procedure 2 with 4-bromo-2,3,5,6-tetrafluoropyridine (60 µL, 0.50 mmol, 1.00 equiv.), Ph_3CCO_2K (335 mg, 1.03 mmol, 2.05 equiv.). Due to the instability of the pentafluorobenzoate product towards our standard isolation procedure, the work-up procedure described in our optimization studies (section 3.1) was performed using EtI (40 µL, 0.50 mmol, 1.00 equiv.). The product was formed in 64% NMR yield, analyzed from the crude reaction mixture by quantitative ^{1}H NMR, using 1,1,2,2-tetrachloroethane as an internal standard. Crude NMR data is provided as evidence for the formation of this compound.

Yield: 64% (NMR yield)

^{1}H NMR (400 MHz, $(CD_3)_2CO$) δ: 4.52 (q, 2H, J = 7.1 Hz, $CO_2CH_2CH_3$), 1.40 (t, 3H, J = 7.1 Hz, $CO_2CH_2CH_3$).

^{19}F{^{1}H} NMR (376 MHz, $(CD_3)_2CO$) δ: –91.51 - –99.86 (m, 2F, F_{Ar}), –142.69 - –142.87 (m, 2F, *FAr*).

The ^{1}H and ^{19}F NMR resonances are consistent with those previously reported.[8]

Benzyl 2,3,4,5,6-pentafluorobenzoate (3w)

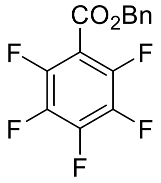

Following general procedure 2 with bromopentafluorobenzene (62 µL, 0.50 mmol, 1.00 equiv.), Ph_3CCO_2K (335 mg, 1.03 mmol, 2.05 equiv.). Due to the instability of the pentafluorobenzoate product towards our standard isolation procedure, the work-up

procedure described in our optimization studies (section 3.1) was performed using BnBr (178 µL, 1.50 mmol, 3.00 equiv.). The product was formed in 78% NMR yield, analyzed from the crude reaction mixture by quantitative ^{1}H NMR, using 1,1,2,2-tetrachloroethane as an internal standard. A sample of the product was isolated for characterization purposes via column chromatography (Column eluent: 0% to 5% Et_2O in hexane).

Yield: 78% (NMR yield)

^{1}H NMR (400 MHz, $CDCl_3$) δ: 7.45 – 7.35 (m, 5H, H_{Ar}), 5.41 (s, 2H, CO_2CH_2).

^{13}C NMR (101 MHz, $CDCl_3$) δ: 158.9 (CO_2CH_2), 145.5 (dm, J = 258 Hz, CF_{Ar}), 143.3 (dm, J = 260 Hz, CF_{Ar}), 137.7 (dm, J = 251 Hz, CF_{Ar}), 134.5 (C_{Ar}), 128.8 (CH_{Ar}), 128.7 (CH_{Ar}), 128.4 (CH_{Ar}), 108.5 - 108.0 (m, CF_{Ar}), 68.5 (CO_2CH_2).

^{19}F{^{1}H} NMR (376 MHz, $CDCl_3$) δ: –137.8 - –137.9 (m, 2F, F_{Ar}), –148.3 - –148.4 (m, 1F, F_{Ar}), –160.2 - –160.4 (m, 2F, F_{Ar}).

The ^{1}H, ^{13}C and ^{19}F NMR resonances are consistent with those previously reported.[2]

5. Robustness Screen

A Biotage® microwave vial was equipped with a magnetic stir bar and septum and dried with a heat-gun under vacuum. Upon cooling, the vial was then charged 2-bromobenzothiazole (107 mg, 0.50 mmol, 1.00 equiv.), potassium 2,2,2-triphenyl acetate (326 mg, 1.00 mmol, 2.00 equiv.) and an additive (if a solid, 0.50 mmol, 1.00 equiv.), and placed under a nitrogen atmosphere through cycles of vacuum/nitrogen gas. DMF (2.5 mL, 0.2 M) was then added to the vial (Note: liquid additives were introduced at this stage). Under a flow of nitrogen gas, the septum was replaced with a crimp cap and firmly sealed using a Biotage® crimper. The reaction mixture was stirred at 50 °C for 30 minutes. After this time, methyl iodide (250 µL, 4.00 mmol, 8.00 equiv.) was added and the mixture was stirred at 50 °C for 2 h. The reaction was cooled to room temperature and quenched with 10 mL aqueous $NaHCO_3$ (sat.). The reaction mixture was extracted with Et_2O (3 × 10 mL), dried over Na_2SO_4, and then concentrated under *vacuo*. The reaction mixture was analysed using quantitative ^1H NMR, using 1,1,2,2-tetrachloroethane or dibromomethane as an internal standard.

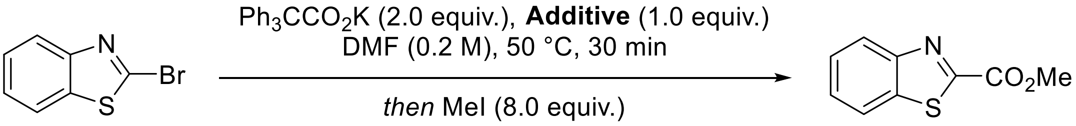

1a-Br → 3a

Additive	Yield 3a (%)	Additive Recovery (%)	Additive	Yield 3a (%)	Additive Recovery (%)
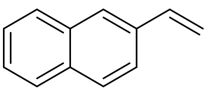	80% ✓	93% ✓	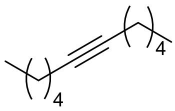	77% ✓	99% ✓
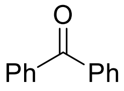	83% ✓	>99% ✓	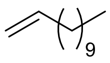	83% ✓	80% ✓
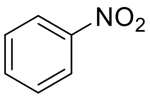	85% ✓	79% ✓	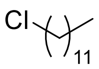	78% ✓	87% ✓
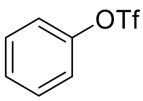	83% ✓	67% ✓	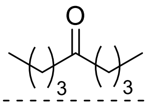	80% ✓	90% ✓
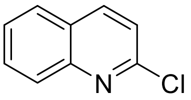	79% ✓	>99% ✓	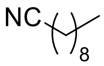	75% ✓	98% ✓

Table S2. Robustness Screen. In accordance with the ranges proposed by Glorius and co-workers[9]: Green = 66% – 100%, Yellow = 33% - 66%, Red = 0% - 33%.

6. Unsuccessful Examples

Unsuccessful examples are provided below. In all cases low mass recovery was observed. In general, few side products were observed suggesting that mass loss was largely due to decomposition.

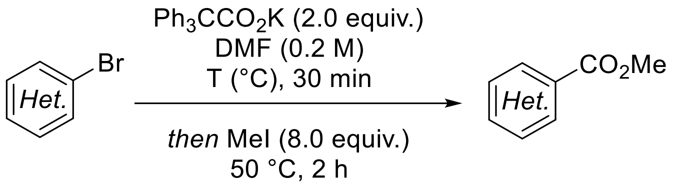

Substrate	T °C	Yield (%)[a]	Recovered SM (%)[a]	Other (%)[a]
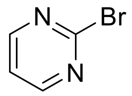	100	0	6	Ph_3CH = 32<br>Ph_3CCO_2Me = 30
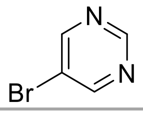	140	11[b]	0	/
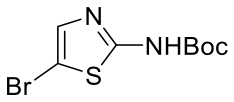	50	0	0	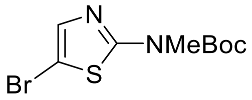<br>65<br>Ph_3CH = 49<br>Ph_3CCO_2Me = 42
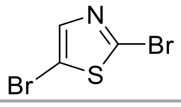	50	22[b,c,d]	0	/
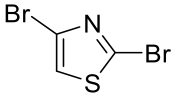	50	22[b,c]	0	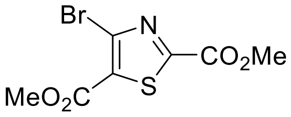<br>22[b]
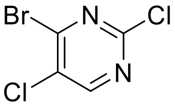	100	N/A	10	/

[a] NMR Yield using 1,1,2,2-tetrachloroethane internal standard. [b] We were unable to isolate these compounds, so these are only tentative assignments based on the appearance of the correct molecular ion peak by GCMS and relevant peaks in the ^{1}H NMR. [c] GCMS and ^{1}H NMR suggest mono-carboxylation occurred, but as we were unable to isolate these compounds this is only a tentative assignment. [d] 1.2 equiv. BnBr.

7. Mechanistic Studies

7.1. Radical Trap Experiment

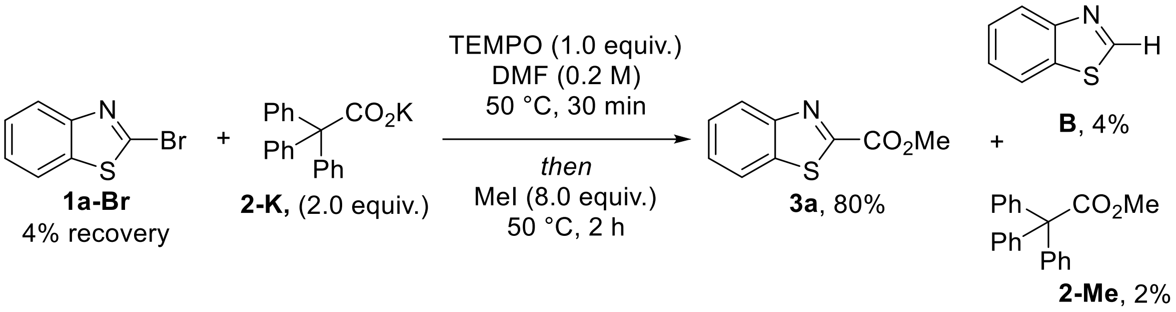

Adapted from the works of Perry et al.[2] A Biotage® microwave vial was equipped with a magnetic stir bar and septum and dried with a heat-gun under vacuum. The vial was then charged with 2-bromobenzothiazole (107 mg, 0.50 mmol, 1.00 equiv.), Ph_3CCO_2K (326 mg, 1.00 mmol, 2.00 equiv.) and TEMPO (78 mg, 0.50 mmol, 1.00 equiv.). The vessel was placed under a nitrogen atmosphere through cycles of vacuum/nitrogen gas and DMF (2.5 mL, 0.2 M) was then added to the vial. Under a flow of nitrogen gas, the septum was replaced with a crimp cap and firmly sealed using a Biotage® crimper. The reaction mixture was then stirred at 50 °C for 30 mins. After this time, methyl iodide (250 µL, 4.00 mmol, 8.00 equiv.) was added and the mixture was stirred at 50 °C for 2 h. The reaction was cooled to room temperature and the reaction quenched with 10 mL $NaHCO_3$ (aq. sat.) and extracted with Et_2O (3 × 10 mL). The organic phase was dried over Na_2SO_4 and then concentrated under *vacuo*. The reaction mixture was analysed using quantitative ^1H NMR, using 1,1,2,2-tetrachloroethane as an internal standard.

7.2. Synthesis of trityl 2,2,2-triphenylacetate (6)

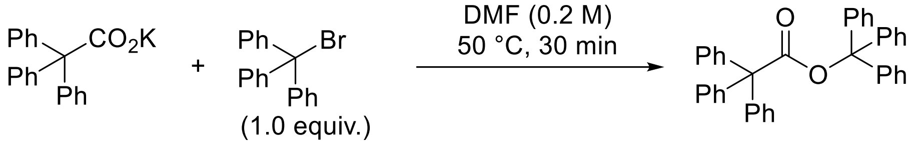

A Biotage® microwave 2 – 5 mL vial equipped with stir bar was dried under *vacuo* with a heat gun. Upon cooling to room temperature, the vial was charged with Ph_3CCO_2K (163 mg, 0.50 mmol, 1.00 equiv.) and bromotriphenylmethane (162 mg, 0.50 mmol, 1.00 equiv.). The vial was then placed under a nitrogen atmosphere and cycled through vacuum and N_2 gas. DMF (2.5 mL, 0.2 M) was then added. Under an N_2 gas flow, the septum was replaced by a Biotage® crimp cap. The mixture was stirred at 50 °C for 30 mins. After this time, $NaHCO_3$ (46 mg, 0.55 mmol, 1.10 equiv.) was added and the mixture was stirred for a further 2 minutes. The reaction mixture was then quenched with 10 mL $NaHCO_3$ (sat) and extracted with Et_2O (3 × 10 mL). The organic phases were dried over Na_2SO_4 and passed through a Al_2O_3 plug. The mixture was then concentrated under vacuum to give the product as a pale-yellow powder.

Yield: 236 mg (89%, 0.45 mmol)

^{1}H NMR (400 MHz, $CDCl_3$)δ: 7.24 - 7.16 (m, 30H, Ar*H*)

^{13}C NMR (101 MHz, $CDCl_3$)δ: 170.0 (Ph_3CCO), 143.0 (C_{Ar}), 142.7 (C_{Ar}), 130.4 (CH_{Ar}), 128.6 (CH_{Ar}), 127.7 (CH_{Ar}), 127.6 (CH_{Ar}), 127.2 (CH_{Ar}), 126.6 (CH_{Ar}), 91.4 (Ph_3CO), 67.9 (Ph_3CCO).

HR-MS (ESI$^+$): Found *m/z* 569.1866, $[M + K]^+$; calculated for $[C_{39}H_{30}O_2K]^+$: 569.1877.

X-Ray Crystallography (6, CCDC: 2333766):

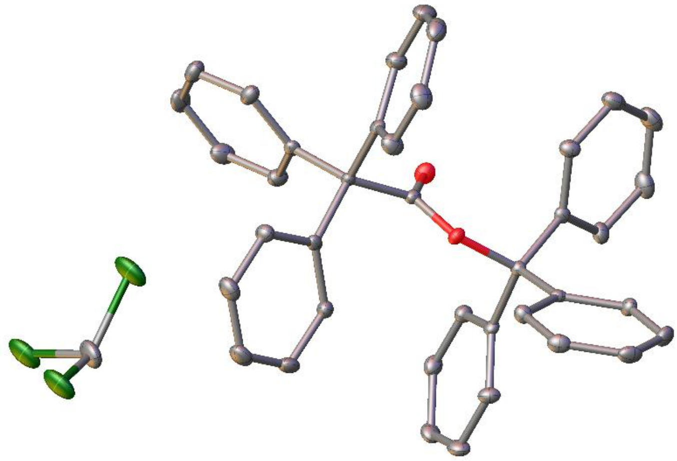

Experimental. Single clear colourless prism-shaped crystals of **6** recrystallised from chloroform by slow evaporation. A suitable crystal with dimensions $0.13 \times 0.06 \times 0.04$ mm^3 was selected and mounted on a MITIGEN holder with silicon oil on a ROD, Synergy Custom system, HyPix diffractometer. The crystal was kept at a steady T = 100(2) K during data collection. The structure was solved with the ShelXT 2014/5 (Sheldrick, 2014) solution program using dual methods and by using Olex2 1.5-alpha (Dolomanov et al., 2009) as the graphical interface. The model was refined with ShelXL 2016/6 (Sheldrick, 2015) using full matrix least squares minimisation on $\boldsymbol{F}^2$.

Crystal Data. $C_{39.5}H_{30.5}Cl_{1.5}O_2$, M_r = 590.31, monoclinic, $I2/a$ (No. 15), a = 15.5030(3) Å, b = 8.9838(2) Å, c = 43.3801(12) Å, b = 99.915(2)°, a = g = 90°, V = 5951.6(2) Å^3, T = 100(2) K, Z = 8, Z' = 1, m(Mo K_a) = 0.209, 158841 reflections measured, 9964 unique (R_{int} = 0.0933) which were used in all calculations. The final wR_2 was 0.2833 (all data) and R_1 was 0.1371 (I≥2 s(I)).

7.3. Isolation of trityl 2,2,2-triphenylacetate (6) from the reaction mixture

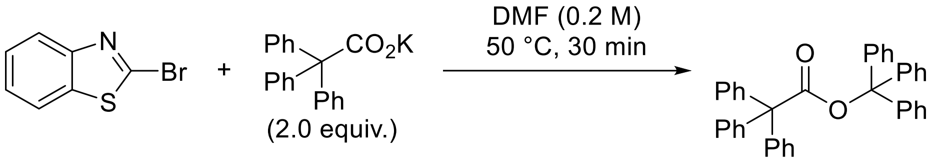

A Biotage® microwave vial, equipped with stir bar and septum was dried with a heat gun under *vacuo*. Upon cooling to room temperature, the vial was charged with 2-bromobenzothiazole (214 mg, 1.00 mmol, 1.00 equiv.) and Ph_3CCO_2K (653 mg, 2.00 mmol, 2.00 equiv.). The vial was placed under a nitrogen atmosphere through cycling with vacuum/nitrogen gas. DMF (5.0 mL, 0.2 M) was then added to the vial. Under an N_2 gas flow, the septum was replaced by a Biotage® crimp cap, and the reaction was stirred at 50 °C for 30 min. The mixture was then allowed to cool to room temperature and then diluted with sat. $NaHCO_3$ (aq., 20 mL). The organic phase was recovered with Et_2O (3 x 20 mL), dried over Na_2SO_4 and concentrated under *vacuo* until ~10% solvent remained. The mixture was then filtered under vacuum and washed with hexane (3 x 5 mL). The eluent was concentrated under vacuum to give the product as a pale-yellow solid.

Yield: 399 mg (76%, 0.75 mmol).

The spectral data matched that reported in section 7.2 above.

7.4. Synthesis of Potassium benzothiazole-2-carboxylate

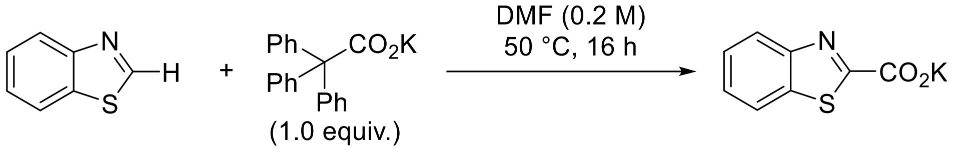

From the works of Perry et al.[2] A Biotage® microwave vial was equipped with a magnetic stir bar and septum and dried with a heat-gun under *vacuo*. The vial was then charged with Ph_3CCO_2K (326 mg, 1.00 mmol, 1.00 equiv.) and placed under a nitrogen atmosphere through cycles of vacuum/nitrogen gas. DMF (5.0 mL, 0.2 M) and benzothiazole (109 µL, 1.00 mmol, 1.00 equiv.) were added to the vial. Under an N_2 gas flow, the septum was replaced with a Biotage® crimp cap. The reaction mixture was stirred at 50 °C for 16 hours. After this time, the reaction mixture was cooled to room temperature and then diluted with 20 mL hexane with rapid stirring. The precipitate was then filtered under vacuum and washed with hexane (3 x 5 mL) and 5 mL Et_2O. The obtained white solid was then dried under vacuum.

Yield: 207 mg (95%, 0.95 mmol).

^{1}H NMR (400 MHz, D_2O): δ 8.08 – 8.02 (m, 2H, Ar*H*), 7.60 – 7.54 (m, 2H, Ar*H*).

^{13}C NMR (101 MHz, D_2O): δ 167.4 (*C*O_2K), 166.1 (C_{Ar}), 152.3 (C_{Ar}), 136.2 (C_{Ar}), 126.8 (CH_{Ar}), 126.7 (CH_{Ar}), 123.5 (CH_{Ar}), 122.5 (CH_{Ar}).

The ^{1}H and ^{13}C NMR resonances are consistent with those previously reported.[2]

7.5. Carboxylate Reactivity

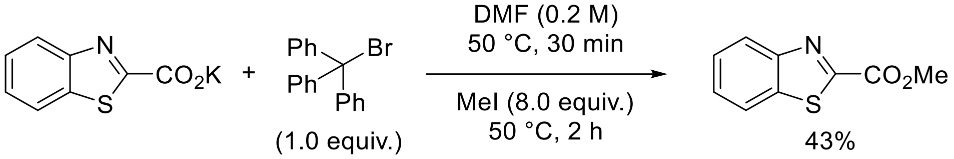

A Biotage® microwave vial was equipped with a magnetic stirrer bar and septum and dried with a heat-gun under *vacuo*. The vial was then charged with potassium benzothiazole-2-carboxylate (109 mg, 0.50 mmol, 1.00 equiv.) and trityl bromide (162 mg, 0.50 mmol, 1.00 equiv.) and placed under a nitrogen atmosphere through cycles of vacuum/nitrogen gas. DMF (2.5 mL, 0.2 M) was then added to the vial. Under an N_2 gas flow, the septum was replaced with a Biotage® crimp cap. The reaction mixture was stirred at 50 °C for 30 minutes. After this time, methyl iodide (250 µL, 4.00 mmol, 8.00 equiv.) was added and the mixture was allowed to stir at 50 °C for a further 2h. Upon completion, the reaction mixture was cooled to room temperature, quenched with $NaHCO_3$ (sat., 10 mL) and extracted with Et_2O (3 × 10 mL). The organic phase was then dried over Na_2SO_4, concentrated under *vacuo* and analysed using quantitative ^1H NMR, using a 1,1,2,2-tetrachloroethane internal standard.

This experiment was run to assess the reactivity of potassium benzothiazole-2-carboxylate, the product of the C–X carboxylation, in comparison to potassium 2,2,2-triphenylacetate (**2-K**). In this reaction, an unknown species was detected in 24% which may correspond to trityl benzothiazole-2-carboxylate, however, we were unable to isolate or characterize this species. Nonetheless, as we observed 43% of methyl benzothiazole-2-carboxylate, the maximum yield of the reaction between potassium benzothiazole-2-carboxylate and trityl bromide is 57%. This reactivity is much lower than the potassium 2,2,2-triphenylacetate (**2-K**) which shows complete conversion within 30 minutes (see section 5.2). This somewhat explains why under the standard reaction conditions trityl 2,2,2-triphenylacetate (**6**) is the only side product and trityl benzothiazole-2-carboxylate is not observed.

8. Computational Studies

8.1. Computational Methods

All geometry optimizations and frequency calculations for reported structures were performed using the B3LYP functional[10] with the def2-SVP basis set using the Gaussian 16 (G16) program.[11] Dispersion interactions were included using Grimme's DFT-D3 correction.[12] The SMD solvent effects were incorporated into all calculations with DMF as the solvent.[13] This level is referred to as SMD(DMF)/B3LYP-D3/def2-SVP. All stationary points have been verified, through vibrational analysis, to be minima (zero imaginary frequencies) or transition structures (one imaginary frequency). The character of the normal mode associated with the imaginary frequency has been analyzed to ensure it resembles the reaction under consideration. Energy changes were shown by the use of Gibbs free energies (T = 298.15 K and P = 1 atm). Optimized structures were illustrated using CYLview20.[14] Potential energies were refined by means of single point calculations using the B3LYP functional with the def2-TZVPP basis set.[10] This level is denoted SMD(DMF)/B3LYP-D3/def2-TZVPP//SMD(DMF)/B3LYP-D3/def2-SVP.

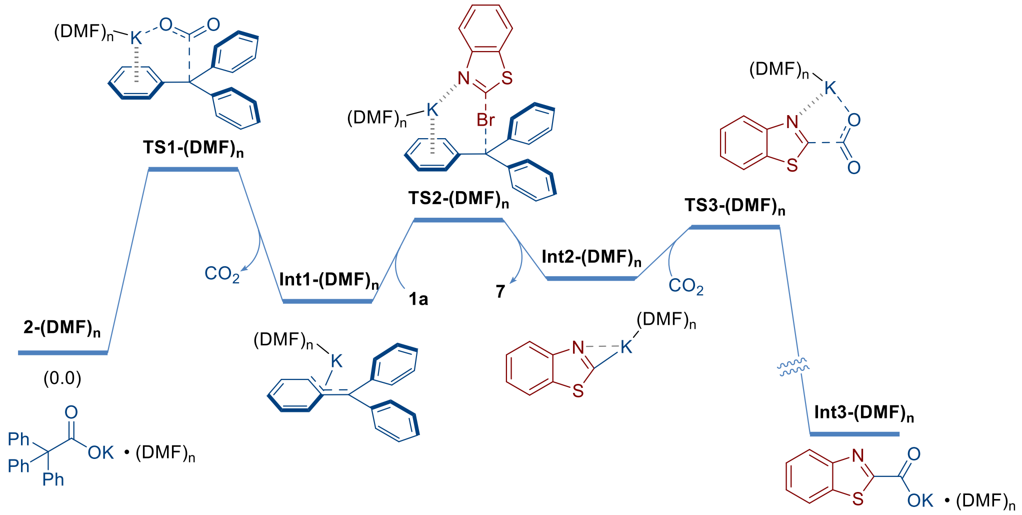

n	2-(DMF)$_n$	TS1-(DMF)$_n$	Int1-(DMF)$_n$	TS2-(DMF)$_n$	Int2-(DMF)$_n$	TS3-(DMF)$_n$	Int3-(DMF)$_n$
0	0.0	20.7	12.4	17.5	12.0	16.3	−10.3
1	0.0	16.6	7.3	11.8	7.0	11.6	−14.9
2	0.0	14.8	4.7	10.5	7.2	12.6	−15.7
3	0.0	16.8	4.4	15.2	7.2	11.8	−13.4

Scheme S1. Potential energy diagram for the C–X carboxylation computed with DMF molecules at the SMD(DMF)/B3LYP-D3/def2-TZVPP//SMD(DMF)/B3LYP-D3/def2-SVP level of theory. Gibbs free energies (ΔG [kcal mol^{-1}]) are provided in the insert.

8.2. Computational Methods

structure	*E*	*H*	*G*	E^{high}	*Imag. Freq.*
CO_2	-188.445146	-188.429877	-188.454176	-188.671992	-
**1a-Br**	-3295.644018	-3295.542675	-3295.585560	-3296.4505	-
**7**	-3306.457252	-3306.159612	-3306.222919	-3307.55935	-
**2-K-(DMF)$_0$**	-1520.958694	-1520.645694	-1520.714353	-1522.04329	-
**2-K-(DMF)$_1$**	-1769.335013	-1768.909745	-1768.998261	-1770.7012	-
**2-K-(DMF)$_2$**	-2017.707137	-2017.170677	-2017.277795	-2019.35543	-
**2-K-(DMF)$_3$**	-2266.076781	-2265.428976	-2265.557395	-2268.0071	-
**TS1-(DMF)$_0$**	-1522.011718	-1520.614592	-1520.686366	-1520.9254	258.8i
**TS1-(DMF)$_1$**	-1769.297605	-1768.875609	-1768.968610	-1770.66706	237.4i
**TS1-(DMF)$_2$**	-2017.676941	-2017.143357	-2017.251633	-2019.32774	239.4i
**TS1-(DMF)$_3$**	-2266.046911	-2265.401981	-2265.528539	-2267.97934	252.0i
**Int1-(DMF)$_0$**	-1332.468800	-1332.173655	-1332.238753	-1333.335	-
**Int1-(DMF)$_1$**	-1580.845868	-1580.440487	-1580.523570	-1581.99413	-
**Int1-(DMF)$_2$**	-1829.224128	-1828.706317	-1828.807767	-1830.65393	-
**Int1-(DMF)$_3$**	-2077.595502	-2076.96653	-2077.088005	-2079.30717	-
**TS2-(DMF)$_0$**	-4628.128667	-4627.730483	-4627.820519	-4629.79701	39.5i
**TS2-(DMF)$_1$**	-4876.505366	-4875.995954	-4876.106129	-4878.45589	30.0i
**TS2-(DMF)$_2$**	-5124.879455	-5124.259598	-5124.388317	-5127.11153	19.8i
**TS2-(DMF)$_3$**	-5373.249888	-5372.51771	-5372.660437	-5375.76395	11.7i
**Int2-(DMF)$_0$**	-1321.657089	-1321.557349	-1321.601943	-1322.22777	-
**Int2-(DMF)$_1$**	-1570.026473	-1569.815572	-1569.885186	-1570.88059	-
**Int2-(DMF)$_2$**	-1818.397278	-1818.075069	-1818.16312	-1819.53484	-
**Int2-(DMF)$_3$**	-2066.771396	-2066.338037	-2066.442968	-2068.19059	-
**TS3-(DMF)$_0$**	-1510.115803	-1510.000699	-1510.054150	-1510.90841	188.9i
**TS3-(DMF)$_1$**	-1758.485389	-1758.259089	-1758.336881	-1759.56146	185.5i
**TS3-(DMF)$_2$**	-2006.864034	-2006.526315	-2006.61621	-2008.22087	192.4i
**TS3-(DMF)$_3$**	-2255.232879	-2254.784035	-2254.895966	-2256.87285	184.3i
**Int3-(DMF)$_0$**	-1510.161253	-1510.043406	-1510.095213	-1510.95511	-
**Int3-(DMF)$_1$**	-1758.530211	-1758.301209	-1758.377745	-1759.60776	-
**Int3-(DMF)$_2$**	-2255.271917	-2254.820215	-2254.932789	-2256.91518	-
**Int3-(DMF)$_3$**	-2503.644979	-2503.081854	-2503.206457	-2505.56652	-

Table S2. Energies (in Hartrees), and number of imaginary frequencies of all stationary points, computed at SMD(DMF)/B3LYP-D3/def2-TZVPP//SMD(DMF)/B3LYP-D3/def2-SVP level of theory. Energies (in Hartrees) at SMD(DMF)/B3LYP-D3/def2-SVP level of theory are also provided.

8.3. Cartesian Coordinates

CO_2

O	0.00000000	0.00000000	1.16313200
C	0.00000000	0.00000000	0.00000000
O	0.00000000	0.00000000	-1.16313200

1a-Br

C	-3.80230000	-0.34852300	-0.00018500
N	0.10833900	1.11748000	0.00020600
C	0.88384900	0.09653600	0.00027700
S	0.12181200	-1.49710000	0.00024800
C	-3.60778800	1.04702300	0.00001000
C	-2.32676100	1.59213800	0.00005000
C	-1.22200700	0.72416200	0.00005700
C	-1.43286400	-0.68045500	-0.00011700
C	-2.72023600	-1.22923600	-0.00022500
Br	2.77124000	0.21716800	-0.00012000
H	-2.16178600	2.67164200	0.00021100
H	-4.81825800	-0.75119000	-0.00033500
H	-4.47635500	1.71013200	0.00005900
H	-2.87574200	-2.31008500	-0.00037100

7

C	0.00052700	0.00057200	0.20869300
Br	0.00080900	-0.00090600	2.32179500
H	1.77207700	1.91161300	1.09401900
H	-1.96444400	-0.57755900	-1.64625800
H	0.47729700	1.98595900	-1.65243200
C	-0.90707700	1.15772500	-0.20403900
C	-2.20480400	1.27915400	0.32963100
C	-3.06807300	2.28406500	-0.10346400
C	-2.65732000	3.19102400	-1.08952800
C	-1.37895800	3.07375200	-1.63645900
C	-0.51050900	2.06545500	-1.19853700
H	-2.53922800	0.57689900	1.09528700
H	-4.06847100	2.36050200	0.33061000
H	-3.33301200	3.98082900	-1.42792400
H	-1.04608000	3.76842900	-2.41211400
C	1.45609900	0.20804300	-0.20548900
C	2.04325900	-0.58967400	-1.20032900
C	3.35121600	-0.34380500	-1.63764800
C	4.09340000	0.70339400	-1.09037900
C	3.51406800	1.51339600	-0.10457100

C	2.21175100	1.26999800	0.32836700
H	1.47961500	-1.40379700	-1.65593600
H	3.78527300	-0.98023400	-2.41331700
H	5.11590900	0.89177100	-1.42803000
H	4.08156200	2.34049100	0.33009400
C	-0.54836400	-1.36366500	-0.20482300
C	-0.00186000	-2.54899700	0.32446300
C	-0.44251900	-3.79846100	-0.10837200
C	-1.43832300	-3.89507600	-1.08957000
C	-1.97877100	-2.72873900	-1.63223500
C	-1.53742300	-1.47308600	-1.19494200
H	0.77714900	-2.48857000	1.08661400
H	-0.00651000	-4.70356000	0.32271700
H	-1.78638700	-4.87494100	-1.42688800
H	-2.75100500	-2.78657500	-2.40387600

2-K-(DMF)$_0$

C	-0.08942700	0.00294000	0.03876100
C	1.28113100	0.09646800	0.84908300
H	2.11832200	0.68184900	-1.46825600
H	-2.68400300	0.35229300	1.06302100
H	-1.27749100	1.34619200	-2.11072100
C	-0.73397500	1.40038200	-0.01655700
C	-0.81557200	2.17661200	1.15581300
C	-1.44571700	3.42104700	1.16006500
C	-2.01390800	3.92905600	-0.01634400
C	-1.94527600	3.17073900	-1.18599000
C	-1.31260700	1.91915700	-1.18299200
H	-0.38249700	1.79425300	2.08209500
H	-1.49303000	4.00083300	2.08632700
H	-2.50489600	4.90608100	-0.01642300
H	-2.38520700	3.54909400	-2.11309400
C	0.28773700	-0.46192700	-1.39128200
C	-0.56200100	-1.29470600	-2.13940400
C	-0.24748800	-1.66159200	-3.45280700
C	0.92840900	-1.20207500	-4.05243900
C	1.77817000	-0.36192100	-3.32519700
C	1.45832100	0.00716000	-2.01534200
H	-1.49083600	-1.65963200	-1.69872600
H	-0.93042200	-2.31044300	-4.00845000
H	1.17814600	-1.49150700	-5.07680700
H	2.69868100	0.01437000	-3.78063900
C	-1.04065500	-1.01425200	0.70279000

C	-0.64565300	-2.36249900	0.83025400
C	-1.48390000	-3.31346600	1.41248100
C	-2.75255900	-2.94878000	1.88304800
C	-3.16696700	-1.62332500	1.75018000
C	-2.32147800	-0.67067700	1.16283100
H	0.33484100	-2.66161200	0.46466200
H	-1.14616100	-4.35058200	1.49638400
H	-3.41031300	-3.69384700	2.33920300
H	-4.15788900	-1.31838400	2.09854800
O	1.89672200	1.19180400	0.78498600
O	1.69081400	-0.93117900	1.43992800
K	3.99436600	0.18452700	1.99167800

2-K-(DMF)$_1$

O	3.38586100	1.24568000	0.91984400
C	3.20150800	0.05596100	0.63937000
H	2.38676300	-0.52677300	1.11477100
N	3.92377300	-0.65683600	-0.24297300
C	5.01606200	-0.05727200	-0.98789400
H	5.05453500	1.01696100	-0.76997500
H	4.86296100	-0.20522300	-2.06940500
H	5.97800500	-0.51988300	-0.70679800
C	3.67737700	-2.07251200	-0.45065600
H	2.80141700	-2.38959100	0.13193900
H	4.55079300	-2.66911200	-0.13513300
H	3.48185800	-2.27726000	-1.51523500
C	-1.05908300	0.02420800	0.10381400
C	-0.40955800	0.88855300	1.28297400
H	0.48050500	2.09113000	-0.83125300
H	-3.51653700	-1.33235100	0.16367900
H	-1.97845000	0.36438800	-2.50698200
C	-2.33034200	0.72911500	-0.40342300
C	-3.25012900	1.26974600	0.51520500
C	-4.44682400	1.84689400	0.08846000
C	-4.75869700	1.90028200	-1.27677300
C	-3.85989300	1.36357100	-2.20042400
C	-2.66036300	0.78319200	-1.76509900
H	-3.02584300	1.23293600	1.58261200
H	-5.14170600	2.25949000	0.82556600
H	-5.69416500	2.35569600	-1.61320900
H	-4.08761900	1.39209300	-3.26981900
C	0.00568800	-0.00493800	-1.02472600
C	0.26583600	-1.16065400	-1.77957800

C	1.19085800	-1.14940700	-2.83066100
C	1.88184500	0.02131000	-3.15235300
C	1.62023100	1.18560300	-2.42189900
C	0.69047100	1.17294400	-1.38002600
H	-0.26276400	-2.08807600	-1.55741300
H	1.37100700	-2.06715600	-3.39723700
H	2.61188100	0.02856200	-3.96610400
H	2.14673800	2.11364600	-2.66171200
C	-1.39303100	-1.39894100	0.59460800
C	-0.36985800	-2.23682500	1.08597300
C	-0.63808400	-3.53092300	1.53269300
C	-1.94452400	-4.03643000	1.50087200
C	-2.96822800	-3.22841200	1.00545500
C	-2.69280900	-1.92915300	0.55487000
H	0.65215500	-1.86607000	1.10892200
H	0.18141700	-4.15220500	1.90563100
H	-2.15623600	-5.05043000	1.85130400
H	-3.99411600	-3.60490200	0.96027100
O	-0.56851100	2.13314200	1.21115700
O	0.27365300	0.28976800	2.14620200
K	1.71011800	2.55409300	2.49758300

2-K-$(DMF)_2$

O	2.51904100	0.10028300	-1.46231200
C	2.29328200	-0.60506800	-0.47469300
H	1.71369800	-0.21894200	0.38824300
N	2.69609000	-1.88161500	-0.31954400
C	3.44931200	-2.56747400	-1.35347200
H	3.49037500	-1.93545900	-2.24892400
H	2.96189200	-3.52319200	-1.60559600
H	4.47695800	-2.78124400	-1.01187300
C	2.42097300	-2.61917600	0.89973600
H	3.36087100	-2.91620700	1.39527200
H	1.84126700	-3.52991300	0.67792000
H	1.83864200	-1.99610000	1.59250100
C	-1.85725500	0.16044700	0.07184700
C	-0.83675000	1.37372000	-0.16827900
H	-0.73261200	0.06236500	-2.41734200
H	-4.19039900	0.16361600	1.63212200
H	-3.58579500	-1.48039200	-1.37319500
C	-3.21086800	0.49687700	-0.57919300
C	-3.76295700	1.78364000	-0.43355600
C	-5.02271100	2.09454400	-0.94729400

C	-5.76994800	1.12158300	-1.62474200
C	-5.23984200	-0.16164200	-1.77175100
C	-3.97452700	-0.46787200	-1.25182300
H	-3.19707700	2.55279800	0.09476800
H	-5.42501800	3.10368400	-0.81982600
H	-6.75546300	1.36468700	-2.03143800
H	-5.81010100	-0.93583500	-2.29305600
C	-1.22469200	-1.06140300	-0.64438000
C	-1.21022300	-2.34530700	-0.07462400
C	-0.67853500	-3.44119900	-0.76571000
C	-0.14847300	-3.27889800	-2.04799100
C	-0.17354500	-2.00984800	-2.63671900
C	-0.70926700	-0.92066400	-1.94697300
H	-1.62419000	-2.50585500	0.92112500
H	-0.68048100	-4.42704200	-0.29250000
H	0.27516100	-4.13183200	-2.58487100
H	0.23398500	-1.86286100	-3.64081000
C	-2.04007800	-0.10595500	1.57938200
C	-0.92685600	-0.43403100	2.38218200
C	-1.06115700	-0.68716800	3.74749900
C	-2.31918900	-0.62399000	4.36139600
C	-3.43441800	-0.31460900	3.58242500
C	-3.29451000	-0.06381100	2.20959600
H	0.05652400	-0.49478000	1.92238700
H	-0.17531200	-0.94005800	4.33752600
H	-2.42548000	-0.82146400	5.43171800
H	-4.42834100	-0.26971800	4.03673900
O	-0.98140200	2.01823400	-1.23605900
O	0.07025100	1.55031900	0.67702200
K	1.64238600	2.69063200	-1.22787700
O	3.63495300	2.83948900	0.54955400
C	3.99485600	1.83730500	1.17176300
H	3.58896100	1.59794400	2.17900600
N	4.88914600	0.92966100	0.73869600
C	5.52198600	1.06225700	-0.56249300
H	5.39079600	2.08778600	-0.93012100
H	5.06602200	0.36505800	-1.28477700
H	6.59785000	0.84185100	-0.47708000
C	5.21457000	-0.25383100	1.51474900
H	4.59106600	-0.29639900	2.41877000
H	6.27672400	-0.24656100	1.81346700
H	5.02967500	-1.16141500	0.91850900

2-K-(DMF)$_3$

O	-2.55484000	0.96217600	0.95065200
C	-2.54485000	-0.26731500	0.84539700
H	-2.01906900	-0.77060100	0.00877100
N	-3.13870200	-1.12575600	1.70000000
C	-3.85309600	-0.65330300	2.87119400
H	-3.72111700	0.43202100	2.95951000
H	-3.46100200	-1.14190800	3.77839500
H	-4.92983500	-0.88324400	2.79183100
C	-3.09589000	-2.55956500	1.48304700
H	-4.11446900	-2.96674200	1.36337500
H	-2.61684900	-3.06618500	2.33695900
H	-2.51692700	-2.78426800	0.57694300
C	1.55665400	-1.14658900	-0.23678800
C	0.75890000	-0.02484100	-1.06359900
H	0.82165300	0.99560600	1.29563300
H	3.56468300	-2.92583400	-1.04971900
H	3.23909500	-1.17787600	1.99638200
C	3.03410600	-0.73515400	-0.11163700
C	3.73800600	-0.30214400	-1.25236600
C	5.09165200	0.02802400	-1.18795400
C	5.78535800	-0.06545200	0.02684300
C	5.10516900	-0.50077800	1.16499900
C	3.74461600	-0.83400300	1.09271500
H	3.21258300	-0.22368700	-2.20540100
H	5.61069900	0.36317800	-2.09059300
H	6.84543900	0.19743200	0.08034800
H	5.63034900	-0.58604500	2.12063900
C	0.89542400	-1.15763700	1.16433500
C	0.63084200	-2.34458000	1.86610000
C	0.08227200	-2.31830200	3.15462700
C	-0.21392300	-1.10090000	3.77309500
C	0.05918500	0.09062700	3.09242000
C	0.60939500	0.05950800	1.81015600
H	0.85806400	-3.30954000	1.41164300
H	-0.11482500	-3.26026900	3.67394700
H	-0.65018800	-1.08012100	4.77529400
H	-0.16671300	1.05419600	3.55713300
C	1.43744800	-2.52739800	-0.91606100
C	0.16954200	-3.08960800	-1.17665500
C	0.03662500	-4.34642900	-1.76888800
C	1.16985300	-5.09291700	-2.11603200
C	2.43273200	-4.56236900	-1.85006900

C	2.56172100	-3.29980500	-1.25426000
H	-0.72028200	-2.52453000	-0.91128700
H	-0.96325500	-4.74889900	-1.95625600
H	1.06619200	-6.07777000	-2.57986500
H	3.33288200	-5.13097800	-2.10057800
O	1.25905700	1.12587900	-1.07427100
O	-0.34303800	-0.34654900	-1.56200200
K	-1.23630400	2.28980800	-1.09931900
O	-3.43270400	1.57604800	-2.52330100
C	-3.93212800	0.48479800	-2.24403700
H	-3.60721200	-0.45257300	-2.74659300
N	-4.89827600	0.27900900	-1.32792300
C	-5.44421300	1.37993100	-0.55366200
H	-5.13421900	2.33057100	-1.00514800
H	-5.07240100	1.34188200	0.48360800
H	-6.54468000	1.32232900	-0.54471500
C	-5.40362100	-1.04951200	-1.03286500
H	-4.83215700	-1.80581200	-1.58948200
H	-6.46924200	-1.13463500	-1.30730200
H	-5.30722300	-1.26151600	0.04377200
O	-0.30106500	4.67720700	-0.42077600
C	0.79272000	5.24877900	-0.43015100
H	0.95643000	6.18388800	-1.01025800
N	1.88509400	4.84213400	0.23808300
C	1.85041000	3.62295200	1.03645900
H	0.85115100	3.49576800	1.47560500
H	2.58826800	3.70057000	1.84819000
H	2.07331900	2.73828200	0.41774500
C	3.16616400	5.50919700	0.08455200
H	3.05958600	6.38419000	-0.57221600
H	3.90857200	4.82472200	-0.36067800
H	3.54995900	5.84497600	1.06239600

TS1-(DMF)$_0$

C	0.48454200	-0.03435000	-0.18358800
C	-0.16470500	0.02568300	2.19086200
H	2.78900500	0.29074200	1.22904900
H	-1.25562600	1.21139300	-1.95543900
H	2.67784800	1.16279300	-1.37230100
C	0.78557200	1.42183200	-0.34446500
C	-0.11026300	2.41906700	0.11468500
C	0.14881300	3.78044200	-0.04384000
C	1.32705700	4.21264800	-0.66810000

C	2.22860800	3.25081100	-1.13533800
C	1.95955000	1.88682800	-0.98198000
H	-1.02922800	2.11276300	0.61843100
H	-0.57110500	4.51243000	0.33458100
H	1.53658700	5.27912200	-0.78656400
H	3.15050300	3.56250600	-1.63591700
C	1.64377500	-0.95730100	-0.12380700
C	1.71248600	-2.17508000	-0.84797900
C	2.82035100	-3.02379000	-0.77037100
C	3.92350800	-2.69094000	0.02364100
C	3.89347100	-1.48142300	0.73192000
C	2.78513200	-0.63827500	0.65982600
H	0.88725900	-2.45947100	-1.50335400
H	2.82476400	-3.95048300	-1.35290700
H	4.79146700	-3.35319800	0.08280200
H	4.74584100	-1.19263500	1.35504900
C	-0.79173500	-0.52573100	-0.72954400
C	-1.31694400	-1.79664400	-0.35231500
C	-2.53387700	-2.27551100	-0.83743700
C	-3.31586200	-1.49888600	-1.71279300
C	-2.83171100	-0.23880800	-2.09333700
C	-1.60186000	0.23314000	-1.61797600
H	-0.75758900	-2.40664800	0.36020100
H	-2.88879700	-3.25972200	-0.51664400
H	-4.27057200	-1.87193200	-2.09192100
H	-3.41197300	0.38336600	-2.78148300
O	0.85336200	0.09302000	2.78938400
O	-1.36458600	0.00770300	2.17619000
K	-3.75810800	-0.00995100	1.19070800

TS1-(DMF)$_1$

C	1.65668700	0.19395800	0.33761800
C	0.83429000	-0.22255200	-2.13151600
H	3.56370100	1.21192500	-1.35428100
H	0.99428800	-1.80633900	2.14927500
H	4.36933200	0.30888700	0.89787100
C	2.66652700	-0.88813400	0.27199800
C	2.32139200	-2.21853100	-0.09637100
C	3.26210700	-3.24484900	-0.16763700
C	4.61191900	-3.00250100	0.12503500
C	4.98428800	-1.70647700	0.50154900
C	4.03822600	-0.68154000	0.57940500
H	1.28439600	-2.44590300	-0.34720600

H	2.93815600	-4.24674400	-0.46665500
H	5.35214700	-3.80459800	0.06360900
H	6.02747500	-1.48930600	0.75259900
C	2.10834900	1.59331900	0.21168400
C	1.59050700	2.65022900	1.01180400
C	2.02274700	3.97128300	0.87816300
C	3.01176000	4.31416900	-0.05235200
C	3.55967900	3.29189600	-0.84036700
C	3.12235600	1.97405100	-0.71281500
H	0.83895300	2.42720800	1.77156800
H	1.59009900	4.74136700	1.52511800
H	3.35286100	5.34776500	-0.15570800
H	4.33739600	3.52648300	-1.57446600
C	0.36508700	-0.08091600	0.97833800
C	-0.78718600	0.72058400	0.71185800
C	-2.02083800	0.48616200	1.31653300
C	-2.19349700	-0.58495300	2.21255900
C	-1.08594400	-1.40258500	2.48505600
C	0.15597600	-1.15789000	1.88777100
H	-0.70339900	1.53608200	-0.01035400
H	-2.87015000	1.12740400	1.06423100
H	-3.16268400	-0.77425500	2.67966100
H	-1.18412000	-2.23754400	3.18618100
O	1.66304800	0.35298400	-2.72484000
O	-0.11701000	-0.89018700	-1.91512400
K	-2.05688400	-2.27930900	-0.69945000
H	-6.75996900	-0.15329100	0.92668900
H	-5.30473500	0.42862900	1.78599900
C	-5.81803500	2.11945100	-0.31974500
H	-5.45560100	2.68203400	0.55684100
H	-6.92076200	2.11769000	-0.29771400
H	-5.48286200	2.63231700	-1.23202800
O	-4.06861500	-0.86363800	-1.33840900
C	-4.54465900	0.27603100	-1.28638700
H	-4.36424100	1.01431900	-2.09619700
N	-5.30748200	0.75905400	-0.29329100
C	-5.66390600	-0.04999100	0.86038100
H	-5.21347100	-1.04518800	0.76915700

TS1-(DMF)$_2$

C	2.31111900	-0.01079100	-0.31436600
C	1.88973200	-0.17521500	2.25954900
H	4.30486500	1.35246100	0.97481500

H	0.07196100	0.30354300	-1.91056700
H	3.65304200	2.12526100	-1.46131900
C	1.90166900	1.41514500	-0.39303500
C	0.66579300	1.86196400	0.14396400
C	0.26080400	3.19442200	0.07388900
C	1.07696200	4.15882700	-0.53509500
C	2.30074900	3.74907700	-1.07689400
C	2.69905900	2.41041300	-1.01206700
H	0.00532600	1.14353700	0.63349100
H	-0.70017600	3.48548600	0.50837000
H	0.76470700	5.20545200	-0.58401400
H	2.95215700	4.47837300	-1.56888400
C	3.75243800	-0.32398000	-0.29021500
C	4.32726100	-1.42352700	-0.98852200
C	5.69453400	-1.70441500	-0.94702600
C	6.57555900	-0.89517300	-0.21877600
C	6.04474000	0.21206700	0.45947000
C	4.67944900	0.49039900	0.42357400
H	3.68733100	-2.06622900	-1.59571500
H	6.07827500	-2.56272600	-1.50827600
H	7.64616000	-1.11442200	-0.18709900
H	6.70693100	0.86835300	1.03372300
C	1.35006200	-1.02649800	-0.75456800
C	1.46790700	-2.39860600	-0.37335500
C	0.55467600	-3.37113200	-0.78249600
C	-0.55179800	-3.03449300	-1.58316200
C	-0.70242800	-1.69327000	-1.96757300
C	0.22083700	-0.72214800	-1.57054800
H	2.28881500	-2.69508800	0.28160300
H	0.69682400	-4.40681400	-0.45730400
H	-1.27003800	-3.79520300	-1.89927300
H	-1.54861000	-1.39845200	-2.59543200
O	2.59340600	0.68585700	2.63187000
O	1.10168400	-1.05551400	2.30031500
K	-1.40429500	-1.83350400	1.52117200
H	-4.29118400	-0.79725100	-2.33577300
H	-5.52487300	-2.06634000	-2.08899500
C	-6.06484600	0.22486000	-0.54990200
H	-7.00995000	-0.13669700	-0.99053900
H	-5.65328600	1.00429400	-1.21052000
H	-6.27973900	0.67430400	0.42963600
O	-3.95417700	-2.23733100	1.02242200
C	-4.72342700	-1.29573500	0.81021800

H	-5.16229600	-0.70401500	1.64176300
N	-5.11637800	-0.86548000	-0.40235700
C	-4.68065100	-1.53420800	-1.61699700
H	-3.89198400	-2.25649300	-1.37458300
H	-1.82153900	2.01691200	-1.64998000
H	-3.48750500	1.44048200	-1.94811500
C	-3.77964400	3.43376700	-0.17866500
H	-4.68578000	3.32842800	-0.79765000
H	-3.09732400	4.14079400	-0.68012900
H	-4.06326600	3.84989900	0.79802100
O	-2.30201500	0.58272400	1.45411200
C	-2.89653900	1.63879700	1.21641200
H	-3.29966400	2.27143600	2.03535400
N	-3.13275600	2.14526100	-0.00446800
C	-2.67337100	1.47421700	-1.20876300
H	-2.35870200	0.45435500	-0.96715600

TS1-(DMF)$_3$

C	-2.91749200	-0.01593600	0.08705300
C	-1.77671200	-1.55176100	-1.46310900
H	-4.07381000	0.44610900	-2.32897900
H	-1.58352000	1.43654600	2.00464200
H	-4.23066700	2.38418100	-0.31592800
C	-2.36502500	1.28561800	-0.40464600
C	-0.98996500	1.43402900	-0.70116000
C	-0.44141500	2.65739000	-1.08851200
C	-1.25439100	3.79191100	-1.21283700
C	-2.62209600	3.67212400	-0.93902600
C	-3.16256800	2.44474000	-0.53977900
H	-0.32967800	0.56857000	-0.64783100
H	0.62987100	2.71964900	-1.30168300
H	-0.82942500	4.75105200	-1.52106600
H	-3.27542900	4.54601200	-1.02428300
C	-4.28699300	-0.36873200	-0.32870500
C	-5.23257900	-1.01612800	0.51427600
C	-6.51816800	-1.34526100	0.07736900
C	-6.94120400	-1.03548800	-1.22080700
C	-6.03888200	-0.37503200	-2.06872800
C	-4.75454700	-0.05025100	-1.63725200
H	-4.96210500	-1.25225100	1.54461800
H	-7.20491900	-1.83992300	0.77211300
H	-7.94769500	-1.29296000	-1.56139500
H	-6.34082700	-0.11360600	-3.08829100

C	-2.38952200	-0.48174200	1.38016300
C	-2.51028500	-1.83450700	1.81347600
C	-1.94012800	-2.28661100	3.00416100
C	-1.21401800	-1.41647300	3.83510200
C	-1.09859000	-0.07400000	3.44928000
C	-1.67871100	0.38039500	2.26164600
H	-3.02747100	-2.54972200	1.17432800
H	-2.04952200	-3.33965700	3.28230500
H	-0.76119400	-1.77569400	4.76293000
H	-0.55319500	0.63322500	4.08171500
O	-2.03643400	-1.08206900	-2.51610800
O	-1.28432100	-2.36396500	-0.74711000
K	0.96961200	-1.61873400	0.88910900
H	5.77819800	0.21801500	1.22555500
H	5.55416800	-1.22828500	2.24925400
C	6.65231700	-1.47874800	-0.52232800
H	7.46890700	-1.76444400	0.16286500
H	6.85185600	-0.45970500	-0.89174400
H	6.65453300	-2.17072000	-1.37584500
O	3.35160900	-2.58618900	0.38714200
C	4.41807100	-2.41048200	-0.20598900
H	4.67881100	-2.98471600	-1.12127500
N	5.37011200	-1.53382700	0.15833700
C	5.19603900	-0.70674500	1.34365600
H	4.13893100	-0.44332400	1.47554500
H	3.41526900	1.85588100	-2.00189800
H	4.61551100	0.86302300	-1.12590800
C	4.41190300	-0.38223100	-3.48157500
H	5.39655000	-0.56656100	-3.02281800
H	4.48820700	0.52106800	-4.11129600
H	4.15593700	-1.23720500	-4.12317800
O	1.35236600	-0.83393500	-1.63676100
C	2.28247800	-0.96013300	-2.43358700
H	2.27133500	-1.74371400	-3.22281100
N	3.39879600	-0.20833500	-2.45651800
C	3.58604200	0.88079600	-1.51259000
H	2.88750200	0.77062400	-0.67606500
H	4.18176000	3.69609500	1.83800600
H	3.84190100	3.37552500	0.11312700
C	1.57178100	4.34058900	1.42873700
H	1.68313500	4.82805800	0.44567300
H	2.01808600	4.99875800	2.19338600
H	0.50036200	4.22334600	1.64090100

O	1.99753800	0.76941600	1.65121600
C	1.53527600	1.91492700	1.66514300
H	0.46627400	2.10357100	1.88345700
N	2.22599800	3.04370000	1.42837400
C	3.64996100	3.02679700	1.14153300
H	4.03587300	2.00798400	1.25461900

Int1-(DMF)$_0$

C	-0.07063400	-0.03004600	-0.43076200
K	0.64680500	0.12066400	2.46517200
H	0.20622700	-2.54430800	0.75203400
H	0.89441100	2.19065000	-1.75719200
H	1.21950500	-2.26540000	-1.41553400
C	1.36150300	-0.29031900	-0.50328100
C	2.34823200	0.64075600	-0.03235100
C	3.71509000	0.35303000	-0.03012700
C	4.19861700	-0.87293100	-0.50332400
C	3.26411400	-1.79901000	-0.99506900
C	1.90041100	-1.52041800	-0.99941900
H	2.03708500	1.62697600	0.32498500
H	4.41208000	1.10667600	0.35030900
H	5.26818600	-1.09694600	-0.50121600
H	3.60921400	-2.75718500	-1.39725700
C	-1.04139800	-1.13355900	-0.35966500
C	-2.34563200	-1.01862500	-0.92337300
C	-3.29788400	-2.03126200	-0.81871900
C	-3.00657400	-3.23046300	-0.15167000
C	-1.73037200	-3.38368700	0.40511300
C	-0.77628800	-2.36875900	0.30405000
H	-2.60721700	-0.10543500	-1.46310900
H	-4.28150900	-1.88784500	-1.27738800
H	-3.75278900	-4.02524600	-0.07278000
H	-1.47148800	-4.30618800	0.93441900
C	-0.57358700	1.35350800	-0.38998600
C	-1.70132000	1.72699600	0.40119400
C	-2.15812600	3.04467700	0.47384400
C	-1.51662000	4.06759200	-0.23698900
C	-0.41092100	3.73193000	-1.03167900
C	0.04377100	2.41586600	-1.10965400
H	-2.23758200	0.95784800	0.96562400
H	-3.02496900	3.27505600	1.10120300
H	-1.87265300	5.09937100	-0.17846000
H	0.09790600	4.50770100	-1.61269800

Int1-(DMF)$_1$

O	2.41920700	0.46006100	-2.17795900
C	3.62392300	0.55015100	-1.91143900
H	4.30693200	1.16315900	-2.53688300
N	4.24316300	-0.05071300	-0.88628900
C	3.52624500	-0.89970800	0.05253400
H	2.48966300	-1.03307700	-0.27489200
H	3.53181600	-0.44896000	1.05785200
H	4.01319200	-1.88584600	0.11380400
C	5.66337600	0.13790400	-0.64356600
H	6.09315800	0.80238300	-1.40600200
H	6.19105500	-0.82996300	-0.67614000
H	5.82625200	0.58643600	0.35088500
C	-1.26531200	-0.10448200	0.66863000
K	-0.06712700	0.81285400	-1.98608200
H	-2.82058700	1.57097400	-0.94387700
H	0.35723800	-0.45968900	2.85782400
H	-2.82918000	2.08489100	1.34257400
C	-0.84362800	1.25455000	1.00104400
C	0.53625900	1.64160500	1.01439300
C	0.94638200	2.95138200	1.26491000
C	0.01317600	3.96484100	1.52461700
C	-1.34622500	3.61713800	1.54010400
C	-1.76190300	2.31010600	1.29229400
H	1.30424400	0.89011700	0.81756100
H	2.01638900	3.18268000	1.24874400
H	0.33441400	4.99160900	1.71707200
H	-2.09978800	4.37946600	1.76356200
C	-2.55303300	-0.37435900	0.03036500
C	-3.21221500	-1.63678800	0.16066300
C	-4.40840500	-1.93061600	-0.48920900
C	-5.04113600	-0.98540600	-1.31212000
C	-4.43431900	0.26934200	-1.45335100
C	-3.23529300	0.56892000	-0.80375700
H	-2.76773300	-2.39939300	0.80374800
H	-4.86476900	-2.91488600	-0.34093100
H	-5.98067500	-1.21806100	-1.81973000
H	-4.89879700	1.03366900	-2.08482200
C	-0.34944200	-1.22618000	0.95319400
C	-0.18495100	-2.32379800	0.06091300
C	0.70178300	-3.36995500	0.32174500
C	1.48055800	-3.37628200	1.48672200

C	1.34178700	-2.30961600	2.38597800
C	0.44946000	-1.26865400	2.12877500
H	-0.77160300	-2.35473000	-0.86200200
H	0.79544400	-4.18495300	-0.40282900
H	2.17922900	-4.19223700	1.68893800
H	1.93120800	-2.29388400	3.30817900

Int1-(DMF)$_2$

O	-2.19049800	1.46071700	-1.75677900
C	-3.26271400	1.27870600	-1.17056400
H	-3.99031800	0.51649500	-1.51863600
N	-3.68180800	1.94285900	-0.08090100
C	-2.89285300	3.00895300	0.51392900
H	-1.96199800	3.13729300	-0.04918100
H	-2.64955500	2.76509100	1.56033000
H	-3.46128900	3.95420800	0.50035800
C	-4.95437100	1.63831900	0.54985400
H	-5.62070200	2.51710900	0.52342900
H	-4.79968300	1.35414000	1.60399900
H	-5.44524000	0.80405500	0.02949300
C	1.60741400	0.29566600	0.76101900
K	0.19232600	0.48022500	-2.06745500
H	1.80056700	-2.15892500	-0.46591000
H	0.18608600	2.22490100	2.16070500
H	1.70484700	-2.06337700	2.18205200
C	0.49916200	-0.44024900	1.36779500
C	-0.86301000	-0.01197400	1.28320100
C	-1.91709400	-0.73151500	1.84247500
C	-1.68865500	-1.93312700	2.52951700
C	-0.36558800	-2.39181900	2.62298900
C	0.69152700	-1.67246100	2.06633800
H	-1.10178900	0.90128200	0.73909200
H	-2.93630400	-0.34999900	1.72405600
H	-2.51381100	-2.49634300	2.97205400
H	-0.15043600	-3.32591300	3.15245400
C	2.82608100	-0.43324200	0.35778900
C	4.12734100	0.10477000	0.55397800
C	5.28059600	-0.57390200	0.15718500
C	5.19803900	-1.83490800	-0.45028500
C	3.92890100	-2.39412200	-0.65551500
C	2.77540900	-1.71003600	-0.26733600
H	4.22721800	1.07931500	1.03921400
H	6.25958200	-0.11818300	0.33715500

H	6.10156900	-2.36867700	-0.75689600
H	3.83414000	-3.37168200	-1.13915400
C	1.53929000	1.73306900	0.52479400
C	2.25934900	2.36553900	-0.54296500
C	2.14330000	3.72706100	-0.82504300
C	1.31259000	4.55808400	-0.05955000
C	0.61766200	3.97880900	1.01386900
C	0.72987100	2.62047300	1.30089600
H	2.92808200	1.76468900	-1.16626200
H	2.71056500	4.14432900	-1.66351100
H	1.21819600	5.62344900	-0.28422900
H	-0.02036200	4.60250400	1.64865800
O	-0.40883400	-2.02467000	-1.87581700
C	-0.79952100	-2.96109100	-1.17244300
H	-0.08885500	-3.69953400	-0.74428100
N	-2.07718700	-3.21066000	-0.84173800
C	-3.15854800	-2.34775100	-1.28396200
H	-2.75974000	-1.56776300	-1.94240500
H	-3.64758800	-1.87388100	-0.41713000
H	-3.91347100	-2.93520800	-1.83252700
C	-2.44133900	-4.34911900	-0.01722900
H	-1.53940800	-4.89401900	0.29407100
H	-3.09888800	-5.03656300	-0.57621400
H	-2.97503000	-4.01163200	0.88570600

Int1-(DMF)$_3$

O	1.65501600	-2.89968200	-1.05860900
C	2.87637600	-2.99293900	-1.18963700
H	3.33617400	-3.32815300	-2.14548500
N	3.79235000	-2.71579100	-0.24233500
C	3.39926900	-2.25757700	1.07844400
H	2.30735800	-2.20588800	1.14424800
H	3.81996900	-1.25999500	1.27892600
H	3.77200100	-2.95109000	1.85107900
C	5.21457100	-2.86412700	-0.49073300
H	5.65073000	-3.62306900	0.18124400
H	5.73585800	-1.90923900	-0.31581800
H	5.38643100	-3.17287700	-1.53152100
C	-1.32261300	1.53105500	0.75315500
K	0.15801200	-0.69015900	-1.09767300
H	-2.71277400	1.71760000	-1.60104600
H	0.85844300	2.42742500	2.20320900
H	-2.72213300	3.81693900	0.15783200

C	-0.82958700	2.74771100	0.08943700
C	0.51552300	2.89571200	-0.35880900
C	0.96580300	4.04879700	-1.00322700
C	0.10078400	5.12555500	-1.24496900
C	-1.23115500	5.01080900	-0.82013900
C	-1.68068700	3.86117200	-0.17044100
H	1.22199900	2.07560500	-0.22286100
H	2.00758900	4.09758700	-1.33708000
H	0.45327400	6.02769400	-1.75197200
H	-1.92949100	5.83760700	-0.98638400
C	-2.70232300	1.10099300	0.48419500
C	-3.53301300	0.49153300	1.46767600
C	-4.84267000	0.09325000	1.19617100
C	-5.40730500	0.28782700	-0.07219600
C	-4.61624600	0.88591600	-1.06403500
C	-3.30446400	1.27399600	-0.79610000
H	-3.14103400	0.34727200	2.47721700
H	-5.43752900	-0.36397400	1.99345800
H	-6.43273200	-0.02481500	-0.28415100
H	-5.02316600	1.03554300	-2.06931500
C	-0.47499100	0.78470700	1.66880900
C	-0.67553200	-0.60882700	1.94956800
C	0.15766500	-1.33454300	2.79943700
C	1.25124000	-0.73029400	3.43707900
C	1.47508600	0.63383600	3.19177500
C	0.64723600	1.36558100	2.34360100
H	-1.50580700	-1.13330100	1.47369200
H	-0.04643600	-2.39919900	2.95462900
H	1.90846500	-1.30204300	4.09684800
H	2.31372800	1.14275900	3.67840600
O	2.57142400	0.28303300	-1.43957100
C	3.80396600	0.31163400	-1.47139900
H	4.37429600	-0.12562700	-2.31860000
N	4.59281900	0.84978400	-0.52510200
C	4.01955400	1.47600600	0.65561800
H	3.00672600	1.09472700	0.82493100
H	3.97390000	2.57306800	0.54014800
H	4.64080200	1.24335600	1.53355900
C	6.03214800	0.94520600	-0.69719300
H	6.33499700	0.44549200	-1.62781400
H	6.55418800	0.46758300	0.14799300
H	6.34689400	2.00184500	-0.74531000
O	-2.02100900	-1.89789900	-1.82667800

C	-3.21675900	-2.17364400	-1.71051500
H	-3.96346500	-1.84979900	-2.46574600
N	-3.76260200	-2.88413200	-0.70674500
C	-2.96055800	-3.39409700	0.39150400
H	-1.89892100	-3.20425600	0.19561600
H	-3.11780000	-4.47991100	0.50179200
H	-3.24712300	-2.90432600	1.33702300
C	-5.18560800	-3.16703800	-0.65995600
H	-5.69206600	-2.70427400	-1.51777600
H	-5.62719100	-2.76341400	0.26576200
H	-5.36406100	-4.25574000	-0.68552700

TS2-(DMF)$_0$

C	2.16903500	-0.04727000	0.07150100
H	2.08836700	4.49558200	1.37576000
H	2.07694000	-2.71586900	-0.54726900
H	2.37296000	2.09873100	1.85696400
H	4.43292400	-1.10749500	1.21008400
C	2.40295800	-0.41725400	1.53401600
C	1.42660000	-0.19594400	2.52563200
C	1.67851200	-0.48667800	3.86729700
C	2.91839000	-1.00585200	4.25812700
C	3.90049400	-1.22402300	3.28957000
C	3.64555200	-0.93364700	1.94434100
H	0.45270900	0.20715100	2.24464300
H	0.89794100	-0.30864200	4.61190700
H	3.11443800	-1.23806900	5.30825200
H	4.87636400	-1.62534200	3.57617600
C	3.08528000	-0.82695400	-0.86162300
C	4.18966500	-0.21646600	-1.48002800
C	5.08137400	-0.96189100	-2.26105900
C	4.89056300	-2.33429600	-2.43486500
C	3.80295300	-2.95849700	-1.81091500
C	2.91654800	-2.21411500	-1.03226300
H	4.37277600	0.85013900	-1.34297200
H	5.93189800	-0.46086100	-2.73127600
H	5.58392600	-2.91599500	-3.04807100
H	3.64331400	-4.03337800	-1.93187600
C	2.11824000	1.45128800	-0.19727400
C	1.90817800	1.91609200	-1.51660400
C	1.75505400	3.27534600	-1.79103100
C	1.80575500	4.21719800	-0.75087300
C	2.03206900	3.77590900	0.55480600

C	2.19299100	2.40905400	0.82798700
H	1.83862900	1.19563800	-2.33369900
H	1.58767900	3.60240000	-2.82072900
H	1.67695000	5.28168500	-0.96243500
C	-7.36212000	-0.67911700	0.46356600
N	-3.38949800	0.40110000	-0.31722400
C	-2.69396400	-0.61441000	-0.78312000
S	-3.74635300	-2.08732400	-0.88698200
C	-6.96842400	0.65032400	0.71155000
C	-5.65828200	1.06296300	0.46900600
C	-4.72533100	0.13615300	-0.02872200
C	-5.13147500	-1.20285800	-0.27672300
C	-6.44949600	-1.61406400	-0.03095500
Br	0.13480700	-0.60365700	-0.41395900
H	-5.34691100	2.09378800	0.65971200
H	-8.39363900	-0.98438000	0.65986800
H	-7.69889700	1.36594700	1.09880500
H	-6.75862200	-2.64480700	-0.22214900
K	-1.44140000	2.25238500	-0.38573300

TS2-(DMF)$_1$

C	0.33459800	3.65571200	1.69882300
H	-0.38757000	2.88289700	1.41125900
H	-0.03373100	4.18106100	2.59576600
H	1.29487200	3.17660400	1.94592100
C	1.44740500	5.68153500	0.82153300
H	2.44722700	5.28348800	1.06350600
H	1.11624400	6.31445900	1.66201000
H	1.52418300	6.30295400	-0.08148900
O	-0.93292000	3.53944800	-0.83202000
C	-0.13223700	4.44129800	-0.56472900
H	0.11916500	5.22856400	-1.30671000
N	0.51046800	4.59392800	0.60387100
C	2.10112000	-0.81131200	0.02405800
H	3.04854700	3.74698500	-0.77762500
H	1.80766900	-3.44745400	0.70704000
H	2.93193100	1.76959700	0.68360100
H	4.21851100	-1.56139600	1.62686900
C	2.31921000	-0.52352000	1.50561800
C	1.40800100	0.26291300	2.23757900
C	1.64198500	0.58700100	3.57403500
C	2.79894600	0.13244300	4.21924200
C	3.71628900	-0.64245400	3.50629000

C	3.47982800	-0.96546300	2.16432400
H	0.50462800	0.63099000	1.75089900
H	0.91481600	1.19967500	4.11390000
H	2.98149100	0.38173300	5.26791600
H	4.62767500	-1.00144900	3.99218700
C	2.91560800	-2.00185200	-0.46262200
C	4.01319900	-1.83868000	-1.32427000
C	4.80203200	-2.93257300	-1.70279600
C	4.51259900	-4.21153400	-1.22351600
C	3.42946700	-4.38675100	-0.35210300
C	2.64585500	-3.29616100	0.02396100
H	4.27188400	-0.84731800	-1.69828700
H	5.64990100	-2.77601800	-2.37529200
H	5.12544400	-5.06633200	-1.52171200
H	3.19313100	-5.38101900	0.03667100
C	2.22284200	0.42443700	-0.86007100
C	1.90216400	0.34096100	-2.23408000
C	1.96622900	1.46198400	-3.06218500
C	2.36703800	2.70193200	-2.54229700
C	2.71963100	2.79288300	-1.19579900
C	2.64750900	1.66711900	-0.36338300
H	1.58781000	-0.61762900	-2.65261000
H	1.70265400	1.36808300	-4.11935400
H	2.40891800	3.58358400	-3.18695100
C	-7.42374300	-0.78741400	0.74138200
N	-3.48790600	-0.31611300	-0.61705700
C	-2.79625000	-1.42736600	-0.49523200
S	-3.83686100	-2.72553900	0.23582700
C	-7.03548400	0.46809000	0.23462200
C	-5.73717900	0.67885400	-0.22985300
C	-4.81060600	-0.37803600	-0.18935400
C	-5.21099900	-1.64162900	0.32437400
C	-6.51730500	-1.84936400	0.79050500
Br	0.02138400	-1.35167000	-0.19473200
H	-5.42935500	1.65122700	-0.62430400
H	-8.44604200	-0.93422000	1.10079000
H	-7.76076200	1.28592600	0.20539400
H	-6.82245500	-2.82212000	1.18459400
K	-1.47455500	1.18450500	-1.66302500

TS2-(DMF)$_2$

C	-1.00207400	5.01429000	0.39407100
H	0.07242800	5.04076100	0.15930200

H	-1.16233700	4.34816300	1.25981400
H	-1.32564000	6.02745400	0.66773200
C	-3.03573000	5.20969100	-1.04062100
H	-2.88358100	6.27346500	-1.28819200
H	-3.70202400	5.14812000	-0.16488100
H	-3.52918500	4.71672900	-1.88965500
O	-0.28122800	2.90037500	-1.25231300
C	-1.32950900	3.51386700	-1.47532600
H	-1.99883600	3.23225900	-2.31460500
N	-1.76510700	4.56270700	-0.76069800
C	-1.97245600	-1.08398300	-0.53485600
H	-4.61713100	2.78953600	-0.09515400
H	-1.11138500	-3.64970900	-0.98984600
H	-3.79364900	0.83324500	-1.34164800
H	-3.57066700	-2.66920500	-2.17511600
C	-2.21069500	-0.98713000	-2.03695100
C	-1.61087300	0.05547300	-2.77513000
C	-1.85073700	0.19932400	-4.14081700
C	-2.70064900	-0.69320700	-4.80938700
C	-3.30951700	-1.72196300	-4.08939500
C	-3.06897700	-1.86610000	-2.71584100
H	-0.95630300	0.76714400	-2.26732700
H	-1.37071100	1.01523400	-4.68845700
H	-2.88654800	-0.58206500	-5.88104200
H	-3.98261800	-2.42178500	-4.59231100
C	-2.48725200	-2.37475400	0.09124300
C	-3.55526800	-2.37395800	1.00415000
C	-4.04754200	-3.57069400	1.54112800
C	-3.48507600	-4.79427500	1.17376300
C	-2.42641900	-4.81191500	0.25586900
C	-1.93717700	-3.61963600	-0.27655900
H	-4.02138700	-1.43312200	1.29848400
H	-4.87910100	-3.53853600	2.25047500
H	-3.86621300	-5.72807300	1.59556700
H	-1.97675300	-5.76199500	-0.04537400
C	-2.44281900	0.16172900	0.21363200
C	-1.99397900	0.43509300	1.52097500
C	-2.46111100	1.54336500	2.22856200
C	-3.40220600	2.40605200	1.65215800
C	-3.87541400	2.13412300	0.36736600
C	-3.40119800	1.02468400	-0.34272400
H	-1.26940900	-0.22958100	1.99150200
H	-2.09193200	1.73167300	3.23935300

H	-3.76336800	3.27813000	2.20314200
C	7.65070200	-0.31539600	-1.25445900
N	3.63015100	0.36870600	-0.28915100
C	2.99048200	-0.75396800	-0.05225700
S	4.12286700	-2.16525300	-0.24753100
C	7.18520200	1.01353500	-1.22433800
C	5.85616000	1.29620800	-0.90859200
C	4.97546400	0.23899000	-0.61838600
C	5.45446900	-1.10006400	-0.65129800
C	6.79166800	-1.37957600	-0.96874300
Br	0.15584600	-1.13287800	-0.30775800
H	5.48872800	2.32581200	-0.88381500
H	8.69590500	-0.51819400	-1.50405700
H	7.87402800	1.83195500	-1.45127000
H	7.15678200	-2.40953800	-0.99322200
K	1.39933300	1.81857700	0.43239700
C	1.07198200	-1.16166800	3.42142200
H	0.36070400	-1.79413500	2.86638600
H	1.62673500	-1.79942100	4.13020200
C	-0.60923900	-0.54803600	5.12935900
H	-0.13271800	-1.18392400	5.89427500
H	-1.41017200	-1.13167700	4.64436800
H	-1.05944400	0.32381500	5.62418200
C	0.58840900	1.18955700	3.90490000
H	-0.00701800	1.85444000	4.56725600
N	0.36707800	-0.11572400	4.14491700
H	1.77422000	-0.71828900	2.70613700
O	1.35550500	1.64779300	3.05497400

TS2-(DMF)$_3$

C	-0.62375400	1.39367600	3.87796700
H	-0.73841200	1.75306100	2.84988400
H	0.44708400	1.22966700	4.08582300
H	-1.00083400	2.15523900	4.57683000
C	-1.49434100	-0.34772500	5.40562400
H	-2.03980500	0.37263700	6.03802400
H	-0.49731300	-0.51058500	5.84899500
H	-2.03834000	-1.30264000	5.40519900
O	-1.73441500	-0.19114800	1.81442900
C	-1.84622500	-0.52625100	2.99522800
H	-2.37695500	-1.45812100	3.28644100
N	-1.36838000	0.15781900	4.05037100
C	2.72024700	-0.25975500	0.07421100

H	0.55901300	3.61694100	1.69032600
H	4.03047200	-2.56298700	-0.59729500
H	1.69040300	1.45492700	2.01714200
H	4.87744200	-0.45047000	1.77165200
C	2.74627500	-0.75879400	1.51773900
C	1.57038000	-1.15065900	2.18698600
C	1.59787300	-1.54750100	3.52494000
C	2.80308100	-1.55494000	4.23717800
C	3.97641400	-1.15589200	3.59267900
C	3.94791400	-0.76362400	2.24904300
H	0.61780400	-1.13833800	1.65827600
H	0.66859300	-1.85015700	4.01339200
H	2.82487300	-1.86862400	5.28425600
H	4.92633900	-1.14887900	4.13424400
C	4.07994300	-0.39984100	-0.59734100
C	4.87595400	0.71807500	-0.89848600
C	6.15877700	0.56632800	-1.44044900
C	6.67586400	-0.70700300	-1.68643600
C	5.89961400	-1.83151000	-1.37628300
C	4.62274700	-1.67768800	-0.83700300
H	4.50467400	1.72372700	-0.69783000
H	6.75490100	1.45492900	-1.66583900
H	7.67558200	-0.82597500	-2.11261500
H	6.29203100	-2.83606400	-1.55668500
C	2.07756500	1.11130000	-0.08617800
C	1.92677700	1.67216200	-1.37446800
C	1.30539800	2.90635100	-1.55854600
C	0.80191100	3.61626200	-0.45854300
C	0.94621600	3.07888700	0.82142100
C	1.58555800	1.84469000	1.00611500
H	2.28744400	1.11897500	-2.24367500
H	1.19770000	3.31261200	-2.56662900
H	0.30097100	4.57556700	-0.60348100
C	-5.07131400	-4.88282300	-0.52546400
N	-1.99583300	-2.25413400	-1.61909600
C	-0.89163000	-2.88672100	-1.93426300
S	-1.15599300	-4.69921600	-1.75474600
C	-5.33478800	-3.49952300	-0.51460900
C	-4.34591000	-2.58342700	-0.87482200
C	-3.07325900	-3.05117800	-1.24984300
C	-2.81507800	-4.45080200	-1.25642400
C	-3.81384600	-5.36729900	-0.89561200
Br	1.35554400	-1.54061500	-1.00265900

H	-4.54826300	-1.50859600	-0.87122600
H	-5.85820300	-5.58696800	-0.24093000
H	-6.32539700	-3.14060700	-0.22182900
H	-3.61389300	-6.44196200	-0.90144500
K	-1.51193700	0.41360600	-0.74761100
C	-2.21396700	4.91957600	1.28860400
H	-2.02173800	4.61045400	0.25553000
H	-1.26882800	5.24132000	1.75456600
H	-2.90792600	5.77824700	1.28676500
C	-3.01018500	4.00874800	3.44871000
H	-3.73424600	4.82534100	3.61486700
H	-2.06834200	4.27630100	3.95464100
H	-3.40433700	3.08888100	3.90255100
O	-3.06175900	2.44815300	0.20138000
C	-3.16454600	2.67355900	1.40862400
H	-3.59247300	1.92359400	2.10693600
N	-2.78194600	3.80664600	2.02943500
C	-2.11056600	4.30105500	-2.66557200
H	-2.39083800	3.52513500	-1.94307200
H	-3.02646000	4.77741100	-3.05741700
H	-1.51795600	5.07487700	-2.15185900
C	-0.75309400	4.65386700	-4.70498500
H	-0.05881200	5.34494300	-4.19740500
H	-1.53983300	5.25425900	-5.19323400
H	-0.19884200	4.10432600	-5.47888600
O	-1.65400700	1.53990800	-3.13380300
C	-1.18453700	2.39475200	-3.88477700
H	-0.56933200	2.11799800	-4.76916100
N	-1.33479400	3.72634900	-3.75186400

Int2-(DMF)$_0$

C	-3.26877700	0.61166400	-0.00024000
N	0.90735000	0.27931500	0.00013400
C	1.30135800	-0.98331000	-0.00008000
S	-0.15627100	-2.06718800	0.00006900
C	-2.50284700	1.79397800	-0.00004600
C	-1.10926500	1.74032400	0.00025300
C	-0.46700900	0.48901000	0.00027300
C	-1.24816200	-0.69829000	0.00021600
C	-2.64967700	-0.64038500	-0.00020700
K	3.52316300	0.66872500	-0.00012800
H	-0.50927900	2.65439400	0.00043400
H	-4.36056200	0.67334300	-0.00057700

H	-3.00783100	2.76386600	-0.00007300
H	-3.24727600	-1.55552900	-0.00041700

Int2-(DMF)$_1$

C	-4.81497100	-0.94609800	-0.10359300
H	-5.14055500	-1.99061300	-0.29546600
N	-5.83894200	-0.09208000	0.04379600
C	-5.62191700	1.32139300	0.30304900
H	-4.54663600	1.52391800	0.37878600
H	-6.11343000	1.61363900	1.24596600
H	-6.04892800	1.92891600	-0.51255400
C	-7.21969100	-0.53464900	-0.05565300
H	-7.73539100	-0.00425500	-0.87379200
H	-7.75873800	-0.32727900	0.88400700
H	-7.25846900	-1.61474200	-0.25494600
O	-3.61689400	-0.64954400	-0.03943900
C	5.68355800	-0.94777300	0.09236300
N	1.57965600	-0.11017900	-0.03568000
C	1.34102300	1.18835400	-0.08117900
S	2.92502500	2.09004100	-0.06863400
C	4.77844200	-2.02711700	0.10191000
C	3.40235500	-1.80337900	0.06060200
C	2.91785000	-0.48376600	0.00903800
C	3.83934400	0.59908600	-0.00050600
C	5.22265300	0.36981100	0.04117700
K	-1.11379500	-0.14462800	-0.09405100
H	2.69488500	-2.63708800	0.06766500
H	6.75923400	-1.14204700	0.12508600
H	5.16009100	-3.05107900	0.14207400
H	5.92790600	1.20490600	0.03371800

Int2-(DMF)$_2$

C	-3.14750000	-2.63778800	0.62999100
H	-3.23108700	-3.74066500	0.73951100
N	-4.32772200	-2.00596200	0.74500400
C	-4.41984000	-0.56042300	0.61984400
H	-3.42573800	-0.11728300	0.74343900
H	-5.09225000	-0.16253500	1.39559200
H	-4.82029300	-0.27561000	-0.36826500
C	-5.57069600	-2.73871500	0.91302000
H	-6.24772800	-2.55106300	0.06216300
H	-6.08332500	-2.42428000	1.83747100
H	-5.36831000	-3.81745700	0.96996000

O	-2.05927500	-2.09111100	0.42978300
C	6.06642600	0.74737100	1.51714700
N	2.37377400	-0.24686200	-0.19581800
C	2.50842400	-0.83899800	-1.36750100
S	4.29330300	-0.97870600	-1.76006800
C	4.89100900	1.06740700	2.22426600
C	3.63685200	0.75839800	1.69766400
C	3.54976500	0.11983500	0.44731900
C	4.74197500	-0.19965400	-0.25940600
C	6.00129400	0.11343900	0.27396600
K	-0.26520400	-0.60417100	-0.71753100
H	2.72043000	1.00408900	2.24130600
H	7.04143700	0.99707300	1.94490400
H	4.96393300	1.56362100	3.19601000
H	6.91592400	-0.13326100	-0.27143400
C	-2.97541200	1.85305800	-1.35716800
H	-3.65638400	1.76761800	-2.23126800
N	-3.51870700	2.53426400	-0.33296700
C	-2.79171700	2.75418500	0.90548000
H	-1.78037000	2.33892100	0.81920300
H	-3.31236000	2.26873800	1.74777000
H	-2.72167000	3.83404300	1.11784600
C	-4.87469800	3.05022600	-0.39628700
H	-4.87970500	4.14336300	-0.24961900
H	-5.49764800	2.59352900	0.39140300
H	-5.32157600	2.82291600	-1.37428200
O	-1.84622100	1.35694400	-1.36991800

Int2-(DMF)$_3$

C	3.54430100	-2.29309400	-1.28469600
H	4.05981300	-3.20254600	-0.90370200
N	4.31527600	-1.19074800	-1.20359500
C	3.84075500	0.10935100	-1.64639900
H	2.80810700	0.03270000	-2.00548100
H	4.48247500	0.49251200	-2.45825300
H	3.87632300	0.82792400	-0.81163700
C	5.67531800	-1.25280400	-0.70008700
H	5.80390800	-0.55768300	0.14480400
H	6.39602000	-0.97358200	-1.48804500
H	5.90603700	-2.27082200	-0.35594800
O	2.39408800	-2.33885800	-1.72129100
C	-6.31536800	0.80876900	-0.32258000
N	-2.52599500	-0.97473900	-0.24064800

C	-2.43608500	-1.93408800	0.65732600
S	-4.06297200	-2.09474200	1.51070600
C	-5.33014000	1.28513400	-1.20942600
C	-4.05339700	0.72293400	-1.22343900
C	-3.75187500	-0.32958400	-0.34007800
C	-4.75205500	-0.80376500	0.55361400
C	-6.03509500	-0.23557400	0.56226000
K	0.17195300	-1.39051100	-0.55535200
H	-3.28342000	1.08722800	-1.90844000
H	-7.31137400	1.26042700	-0.32525900
H	-5.56985200	2.10376000	-1.89370900
H	-6.80306100	-0.60163600	1.24864900
C	3.04076900	-0.62886100	1.75768900
H	3.80804100	-1.39752100	1.99251000
N	3.48786500	0.62890500	1.93830200
C	2.63354400	1.77705900	1.68802700
H	1.64062100	1.43258600	1.37997200
H	3.06013000	2.40998300	0.89262800
H	2.53852800	2.38735400	2.60155300
C	4.83210500	0.90071500	2.41493600
H	4.79854000	1.46008900	3.36535500
H	5.38950100	1.50506100	1.67972400
H	5.37309700	-0.04131800	2.58063000
O	1.91093900	-0.93992200	1.37873600
C	0.82514600	2.13838600	-1.35528900
H	1.69828500	2.68791000	-1.77016900
N	0.16132000	2.84471300	-0.42036400
C	-1.00125600	2.30008700	0.26104700
H	-1.29370700	1.34766200	-0.19580500
H	-1.84599400	3.00439100	0.18876900
H	-0.78076100	2.13491500	1.32990600
C	0.61532500	4.15682100	0.00297900
H	0.87443900	4.14853400	1.07538700
H	-0.17554000	4.90953000	-0.15413200
H	1.50416300	4.45328600	-0.57160900
O	0.53558900	1.00490400	-1.74100600

TS3-(DMF)$_0$

K	2.25218300	2.19527600	0.00003600
C	-4.06725200	-0.07632700	-0.00001100
N	0.04500400	0.70991500	0.00000900
C	0.72903500	-0.41017800	-0.00001100
S	-0.36148100	-1.83877600	0.00006700

C	-3.64364300	1.26735900	-0.00005200
C	-2.28675700	1.58782000	-0.00008300
C	-1.33755800	0.55082100	-0.00003200
C	-1.77334100	-0.80133900	0.00003400
C	-3.13918200	-1.11948800	0.00004700
C	3.03829300	-1.10317600	-0.00002500
H	-1.95131200	2.62831300	-0.00011600
H	-5.13610600	-0.30671400	0.00000300
H	-4.38891100	2.06738000	-0.00007700
H	-3.47174000	-2.16045400	0.00009200
O	3.65722800	-0.08560900	0.00003500
O	2.90623400	-2.27198100	-0.00014800

TS3-(DMF)$_1$

N	-5.71925400	-0.77046600	0.00811300
C	-6.03976800	0.62917500	0.23282000
H	-5.11640400	1.22060600	0.25609800
H	-6.57209300	0.74895500	1.19130300
H	-6.69120900	1.00430200	-0.57414800
C	-6.83721600	-1.69634900	-0.05991100
H	-7.51389100	-1.42267400	-0.88685200
H	-7.41367000	-1.67285600	0.88011200
H	-6.47215900	-2.71969900	-0.22520500
O	-3.44890600	-0.46822300	-0.08555900
C	-4.45232600	-1.18864900	-0.13043500
H	-4.36896000	-2.28355500	-0.29652600
K	-1.18711300	0.70648200	-0.00947700
C	4.46679800	-2.95420300	0.03103300
N	1.38116800	-0.12385200	-0.00124700
C	1.86833400	1.09338700	-0.00635200
S	3.67075300	1.07187200	0.00238800
C	3.13571100	-3.41568300	0.02747400
C	2.06935800	-2.51733000	0.01678200
C	2.33493400	-1.13670700	0.00954100
C	3.68065100	-0.67975700	0.01316100
C	4.75022600	-1.58690000	0.02390100
C	1.01899500	3.34973800	-0.02689200
H	1.03395700	-2.86835200	0.01391900
H	5.28950700	-3.67442300	0.03945700
H	2.93792200	-4.49102300	0.03312800
H	5.78423500	-1.23322300	0.02670600
O	-0.16462200	3.22488900	-0.03233800
O	2.02831500	3.95567400	-0.02596700

TS3-(DMF)$_2$

N	-3.97349600	-0.81386200	0.27257900
C	-4.68789800	-0.68166400	-0.98484700
H	-4.25747200	-1.37327400	-1.71901100
H	-4.60902900	0.34977400	-1.36731400
H	-5.75607900	-0.91731700	-0.84354200
C	-4.41546000	0.02040900	1.37495200
H	-5.47538400	-0.17547200	1.60909500
H	-4.30875400	1.08834200	1.11887300
H	-3.81187400	-0.18783600	2.26966500
O	-2.44749700	-2.33811900	-0.50659000
C	-2.91539900	-1.63644200	0.39551400
H	-2.46815900	-1.62540400	1.41090800
K	0.21885000	-2.67345300	-0.69449000
C	-1.44242800	3.45145000	-0.61947500
N	0.74892300	-0.03508400	-1.37696500
C	2.03167900	0.17288100	-1.23195800
S	2.35758700	1.90938000	-0.81558700
C	-2.14502900	2.27859800	-0.95942500
C	-1.46201400	1.09511200	-1.23912500
C	-0.05836800	1.07938300	-1.17123700
C	0.64354100	2.26814500	-0.83358800
C	-0.04646800	3.45751100	-0.55897600
C	3.66928800	-1.53128300	-0.75553100
H	-1.99489600	0.18136300	-1.50626700
H	-1.99469300	4.36953800	-0.40104100
H	-3.23710700	2.29675300	-1.00527100
H	0.49405300	4.36999600	-0.29549400
O	3.00332400	-2.51456800	-0.82021900
O	4.65995700	-0.92211500	-0.56676300
N	1.17295200	0.40085200	2.31239400
C	2.48244100	-0.22595000	2.37823600
H	2.39595600	-1.28282800	2.09876600
H	2.88890000	-0.15439100	3.40151000
H	3.17698800	0.27383600	1.68576900
C	1.09451100	1.80291100	2.68193200
H	1.78298500	2.39803400	2.06222900
H	1.36878500	1.94107800	3.74218400
H	0.07310600	2.17699000	2.52688200
O	0.04774100	-1.49912100	1.68932000
C	0.07689200	-0.29744900	1.96963100
H	-0.84545900	0.32272500	1.95810200

TS3-(DMF)$_3$

N	0.37825900	-0.94679800	2.49946200
C	-0.57477300	-0.68477600	3.56256100
H	-1.40759400	-0.09139200	3.16523800
H	-0.09260900	-0.12786700	4.38431500
H	-0.96451800	-1.63275900	3.96988500
C	1.55396700	-1.72925500	2.83554900
H	1.26125200	-2.71474200	3.23462200
H	2.16071300	-1.21190800	3.59858500
H	2.17333300	-1.88040400	1.94064200
O	-0.73155100	0.26890900	0.90046100
C	0.21170600	-0.43600000	1.26485800
H	1.02635500	-0.72613200	0.57189100
K	-0.84813500	1.25020100	-1.66617300
C	4.46826100	-2.05407800	-0.67988800
N	1.97177200	1.25937700	-1.26847500
C	2.06275100	2.09939100	-0.27120200
S	3.26066500	1.50748600	0.96227900
C	3.67185600	-2.01142400	-1.84142000
C	2.83367700	-0.92542100	-2.09288400
C	2.78715300	0.13560300	-1.17138800
C	3.59019500	0.08179400	0.00168800
C	4.43415200	-1.01088500	0.24846900
C	0.55228300	3.89758000	0.27461700
H	2.19238400	-0.89728300	-2.97643900
H	5.11796300	-2.91486500	-0.49948300
H	3.70778800	-2.84287600	-2.55060400
H	5.04606300	-1.05133900	1.15300200
O	-0.25498400	3.79956900	-0.59261400
O	1.02094800	4.36157800	1.25175600
N	-1.10442100	-2.74960000	-0.50043400
C	-2.45856000	-2.24728800	-0.33774000
H	-2.76176600	-1.71967000	-1.24936100
H	-2.50946900	-1.54716600	0.51095500
H	-3.14892000	-3.08592700	-0.15579600
C	-0.63290700	-3.70894300	0.48197900
H	-1.21711500	-4.64335800	0.42948400
H	-0.73370000	-3.29417500	1.49868900
H	0.42665500	-3.94145200	0.30521400
O	-0.59871500	-1.40314900	-2.28790400
C	-0.29656700	-2.27118600	-1.46716500
H	0.71119100	-2.73991300	-1.46102000

N	-4.81447300	0.19662500	0.10585500
C	-4.09974400	0.69297600	1.27249300
H	-3.05785400	0.91561100	1.01436400
H	-4.10970900	-0.07757100	2.05788300
H	-4.58272000	1.60175200	1.67395600
C	-5.99996500	-0.60838400	0.34193000
H	-6.73632400	-0.04886500	0.94390200
H	-5.73861000	-1.53127100	0.88657100
H	-6.46663300	-0.88347900	-0.61453700
O	-3.47395700	1.25592800	-1.42221700
C	-4.43399700	0.53718700	-1.13840600
H	-5.08105400	0.09220300	-1.92615800

Int3-(DMF)$_0$

C	-4.41490700	-0.32685700	-0.00023700
N	-0.50759600	1.14237600	0.00015600
C	0.29203800	0.12419200	0.00021000
S	-0.48859300	-1.46915100	0.00008500
C	-4.22189500	1.07052000	-0.00016600
C	-2.94108900	1.61395100	-0.00002600
C	-1.83163100	0.74760800	0.00005000
C	-2.03875100	-0.66092000	-0.00001200
C	-3.33135300	-1.20444400	-0.00015800
C	1.81458700	0.15494500	0.00032800
H	-2.77728000	2.69408200	0.00001900
H	-5.43097300	-0.73003500	-0.00035700
H	-5.09136500	1.73285400	-0.00023300
H	-3.48787400	-2.28560200	-0.00020800
O	2.37142900	1.27572900	0.00030500
O	2.36682800	-0.97704900	0.00035900
K	4.75211100	0.13658300	-0.00036400

Int3-(DMF)$_1$

H	9.01084800	-0.22154900	0.16903100
O	4.76731600	-0.57100100	0.13295500
C	5.90437000	-0.66112500	0.60843500
H	6.16064400	-1.45077400	1.34630700
N	6.93881400	0.14096700	0.31416500
C	6.80921400	1.23917800	-0.62864400
H	5.79463100	1.25227000	-1.04502500
H	7.53672500	1.12258200	-1.44908400
H	7.00698600	2.20031200	-0.12471100
C	8.23969600	-0.03791300	0.93594000

H	8.21528500	-0.89324600	1.62536400
H	8.52366800	0.86575800	1.50124400
C	-6.78455200	0.49354700	0.37935000
N	-2.97475600	-1.14276800	-0.11204200
C	-2.12779200	-0.16367800	-0.12463100
S	-2.82323600	1.45895100	0.06008500
C	-6.66367400	-0.90728100	0.26672700
C	-5.41800700	-1.50503700	0.10147700
C	-4.27105600	-0.69086000	0.04751900
C	-4.40540800	0.72188300	0.16210900
C	-5.66263200	1.32010500	0.32867400
C	-0.61322300	-0.25787800	-0.27980300
H	-5.31034400	-2.58862900	0.01290300
H	-7.77389600	0.94020400	0.50864400
H	-7.56148700	-1.52918900	0.30976800
H	-5.76290500	2.40443900	0.41685500
O	-0.12006200	-1.39879900	-0.41603200
O	-0.01415800	0.84869500	-0.25348400
K	2.34088600	-0.35629400	-0.63883900

Int3-(DMF)$_2$

H	6.70582600	1.87073200	-0.98071700
O	3.39530800	0.17164100	-1.59949600
C	4.46906400	0.73299300	-1.37138600
H	5.44080600	0.23818700	-1.58406000
N	4.61047200	1.96767000	-0.85593700
C	3.45779800	2.78607600	-0.51952100
H	2.54173200	2.18966700	-0.60797100
H	3.38973700	3.65431000	-1.19756500
H	3.54963100	3.16062100	0.51272300
C	5.91816700	2.57423200	-0.67775900
H	6.07494400	2.84995800	0.37822300
H	6.00602600	3.48802100	-1.28988400
C	-7.44231700	0.96570100	0.75559600
N	-4.04495800	-0.85348000	-0.85249300
C	-3.01161300	-0.34363900	-0.26218300
S	-3.35834800	0.89251200	0.96462300
C	-7.61354700	-0.01178500	-0.24669600
C	-6.51560200	-0.64857600	-0.81747000
C	-5.22258300	-0.30505600	-0.38015300
C	-5.06204800	0.68304700	0.63223700
C	-6.17107200	1.32268300	1.20414200
C	-1.54343000	-0.68885900	-0.50442800

H	-6.63447900	-1.40744900	-1.59439900
H	-8.31997400	1.45226600	1.18919400
H	-8.62253200	-0.27091600	-0.57767400
H	-6.04455600	2.08039400	1.98096300
O	-1.29677200	-1.56156500	-1.36446300
O	-0.73247600	-0.04131100	0.20617900
K	1.36902600	-1.31896100	-0.92077400
H	6.79613300	-1.30903000	0.92793400
O	3.21333600	-2.44846500	0.61267500
C	4.38027900	-2.07483600	0.74644700
H	5.22690300	-2.63225000	0.28896800
N	4.78183600	-0.98882700	1.43368800
C	3.82939400	-0.12378600	2.10834000
H	2.83865500	-0.59460800	2.11121000
H	3.76706600	0.85443300	1.60365100
H	4.14758400	0.04420800	3.15005600
C	6.18271800	-0.61638500	1.52115600
H	6.52821600	-0.64385200	2.56861500
H	6.33247500	0.40457700	1.13557000

Int3-(DMF)$_3$

H	4.53452400	-4.20024000	-0.14811300
O	2.58428500	-1.09323400	-1.02673800
C	3.23204800	-2.10766200	-0.76006000
H	2.84345300	-2.88073900	-0.06240600
N	4.44717100	-2.41465800	-1.25215800
C	5.12678800	-1.53760800	-2.18974400
H	4.47301800	-0.69412800	-2.43904400
H	5.37745500	-2.08844400	-3.11173500
H	6.06332000	-1.15372200	-1.75097000
C	5.11628900	-3.65592800	-0.90530400
H	6.11963000	-3.45180900	-0.49721400
H	5.23002600	-4.29834800	-1.79542900
C	-8.68131200	-0.02802000	-0.11098500
N	-4.64621400	-0.72356100	-0.93360000
C	-3.95324900	-0.18127100	0.01623500
S	-4.89339400	0.63277800	1.28484300
C	-8.34702400	-0.74971600	-1.27569200
C	-7.01808700	-1.00982100	-1.59723300
C	-6.00318700	-0.54170600	-0.74173500
C	-6.35253500	0.18572000	0.43126700
C	-7.69279500	0.44536100	0.75140300
C	-2.43043200	-0.16256900	0.16315400

H	-6.74593200	-1.56754000	-2.49645000
H	-9.73220000	0.16419200	0.12084600
H	-9.14350700	-1.10835700	-1.93278900
H	-7.95737600	1.00404700	1.65226500
O	-1.76668700	-0.73668200	-0.72552700
O	-2.01813500	0.44625500	1.18254200
K	0.59783500	0.17081500	0.36019700
H	5.51488100	-2.41331000	2.24686400
O	2.18677300	-0.63384800	2.39876800
C	3.29045000	-1.18060300	2.39239000
H	3.44258000	-2.19459500	2.82436300
N	4.42298500	-0.65334800	1.88809700
C	4.42793900	0.66168200	1.26975700
H	3.53567100	1.21652900	1.58124500
H	4.42787100	0.57257600	0.17212400
H	5.32593200	1.21757500	1.58115700
C	5.67193900	-1.39307000	1.87001600
H	6.43179500	-0.89448700	2.49587300
H	6.06054600	-1.45540300	0.84109200
H	4.68456600	4.73432500	-0.03270900
O	1.61920700	2.54356100	-0.18837800
C	2.63647800	3.23673400	-0.13785900
H	2.75831500	4.03707600	0.62457500
N	3.69965500	3.12211400	-0.95439900
C	3.71783500	2.14079400	-2.02847100
H	3.04264400	1.30883100	-1.79447000
H	4.73820300	1.74637400	-2.14300000
H	3.41460500	2.59894800	-2.98668900
C	4.83556300	4.02177000	-0.85582800
H	4.96415700	4.58956300	-1.79320100
H	5.76225800	3.45441100	-0.66622100

9. Isotope Labeling

The experimental procedures and characterization data in this section relate to the isotope labeling studies that were performed in this study. Both the non-labeled and labeled compounds were prepared in all cases for completeness. The ^{13}C incorporation was determined by quantitative ^{13}C NMR or by the comparison of mass spectral patterns through the following method:

- Relative abundance of ^{13}C = number of carbons × 0.011

- Observed abundance of ^{12}C is obtained from the mass signal intensities at M (m/z).

- Observed abundance of ^{13}C is obtained from the mass signal intensities at M+1 (m/z).

- Corrected abundance of ^{13}C = Observed abundance of ^{13}C – (Observed abundance of ^{12}C × Relative abundance of ^{13}C)

- %^{13}C incorporation = Corrected abundance of ^{13}C / (Corrected abundance of ^{13}C + Observed abundance of ^{12}C)

Representative Example

For compound **3x***:

m/z	Intensity (int.)
278.0084	395
279.0127	51035

- Number of carbons = 10

- Relative abundance of ^{13}C = 10 × 0.011 = 0.11

- Observed abundance of ^{12}C = 395

- Observed abundance of ^{13}C = 51035

- Corrected abundance of ^{13}C = 51035 – (395 × 0.11) = 50992

- %^{13}C incorporation = 50992 / (50992 + 395) = 99%

9.1. Preparation of ^{13}C-labeled 2,2,2-triphenyl acetic acid (2'*)

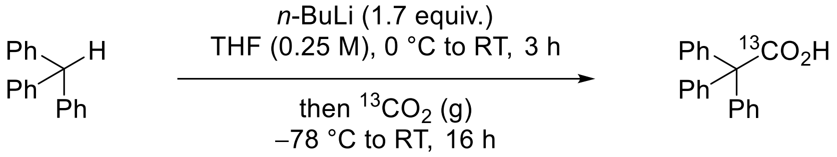

Adapted from the works of Perry et al.[2] A 500 mL 3-neck RBF was equipped with a stir bar and dried with a heat gun under *vacuo*. The flask was charged with triphenylmethane (5.38 g, 22.0 mmol, 1.0 equiv.) and placed under an inert atmosphere through cycles of N_2 gas and vacuum. THF (110 mL, 0.20 M) was added, and the solution was cooled to 0 °C. *n*-BuLi (13.8 mL, 26.4 mmol, 1.2 equiv., 1.91 M in hexane) was added dropwise to form a red solution. Upon complete addition of *n*-BuLi, the solution was warmed to room temperature and stirred for 3 h. The mixture was solidified in a liquid N_2 bath, then placed under vacuum for 5 min. The tap to the vacuum was closed and the mixture was warmed to –78 °C in a dry ice/acetone bath to give a red solution. The flask was filled with $^{13}CO_2$ gas *via* connection to a $^{13}CO_2$ cannister (364592-1L purchased from Merck. One litre of CO_2 gas is approximately 45 mmol, 2.05 equiv. Packaged in a 450 mL carbon steel lecture bottle with brass CGA 110/180 valve and equipped with a stainless-steel control valve. Merck state that nominal gas pressures at 21 ºC are 20 psig. Note: open the control valve carefully to avoid over pressure). The mixture was warmed to room temperature and stirred overnight. The valves between the $^{13}CO_2$ cannister and the reaction vessel were kept open overnight. The product mixture was quenched with 55 mL H_2O and acidified to pH 1 with 2 M HCl. The organic phase was then extracted with EtOAc (3 × 80 mL), dried over Na_2SO_4 and concentrated under *vacuo* until ~10% solvent remained. The contents were then filtered under *vacuo* and washed with ice-cold EtOAc. One portion was purified by filtration and another by column chromatography. Column eluent: 0% to 50% EtOAc in Hexane.

Yield: 3.82 g (60%, 13.2 mmol)

R_f: 0.26 (1:1 Hex:EtOAc)

^{1}H NMR (400 MHz, DMSO-d_6): δ 13.23 (br.s, 1H, CO_2H), 7.33 – 7.29 (m, 6H, Ar*H*), 7.27 – 7.23 (m, 3H, Ar*H*), 7.14 – 7.12 (m, 6H, Ar*H*).

^{13}C NMR (101 MHz, DMSO-d_6): δ 174.8 ($^{13}CO_2$H), 143.7 (d, J = 1.5 Hz. C_{Ar}), 130.4 (d, J = 2.2 Hz, CH_{Ar}), 128.1 (CH_{Ar}), 127.1 (CH_{Ar}), 67.3 (d, J = 53.3 Hz, Ar_3C).

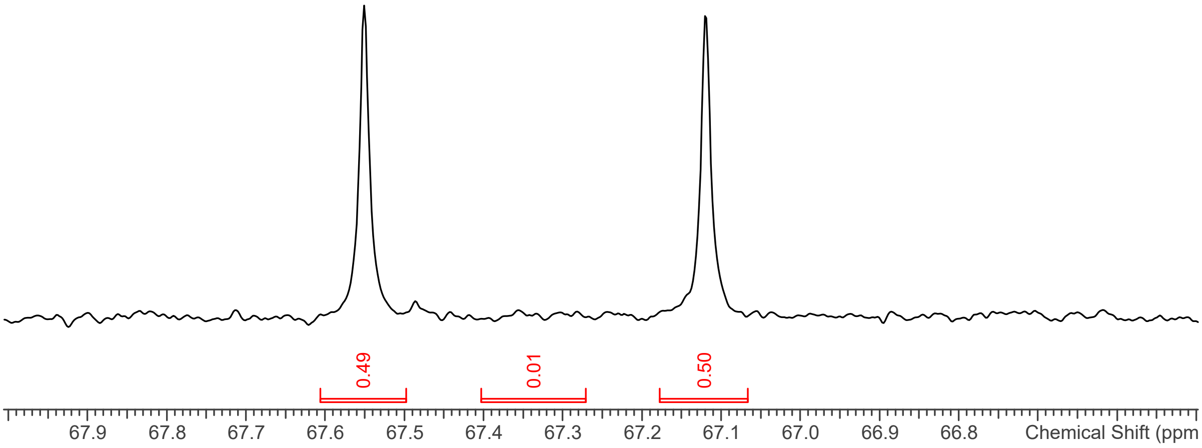

Figure 2: ^{13}C incorporation for $Ph_3C^{13}CO_2H$.

The ^{13}C incorporation was found to be >99% by quantitative ^{13}C NMR.

The ^{1}H and ^{13}C NMR resonances are consistent with those previously reported.[2]

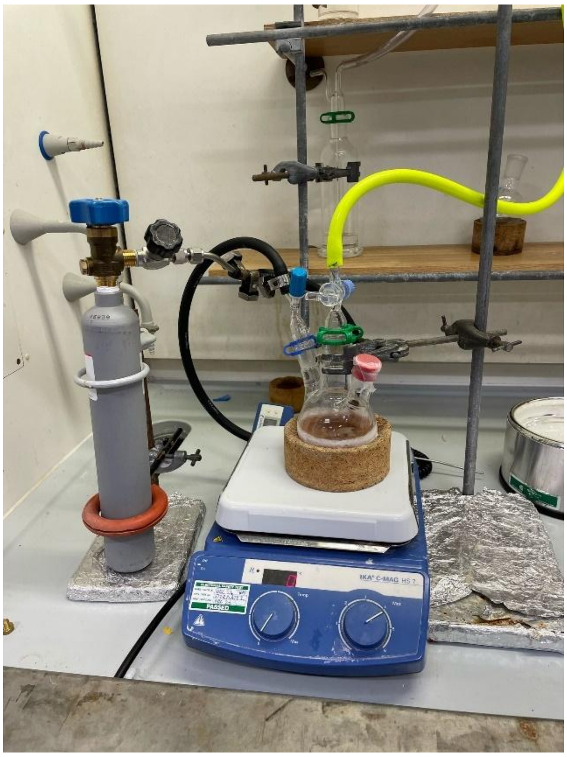

Figure 3: General setup for the preparation of $Ph_3C^{13}CO_2H$.

9.2. Preparation of ^{13}C-labeled potassium 2,2,2-triphenyl acetic acid (2-K*)

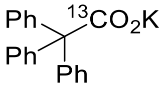

Following general procedure 1 (page S6) with $Ph_3C^{13}CO_2H$ (1.45 g, 5.00 mmol, 1.00 equiv.), KO^{t}Bu (561 mg, 5.00 mmol, 1.00 equiv.) and EtOH (25 mL, 0.2 M). The product was obtained as a white solid.

Yield: 1.38 g (83%, 4.2 mmol)

^{1}H NMR (400 MHz, D_2O): δ 7.35 – 7.27 (m, 15H, Ar*H*).

^{13}C NMR (125 MHz, D_2O): δ 180.3 (^{13}*C*O_2K), 145.5 (d, *J* = 1.5 Hz, *C*$_{Ar}$), 130.3 (d, *J* = 1.5 Hz, *C*H$_{Ar}$), 127.7 (*C*H$_{Ar}$), 126.2 (*C*H$_{Ar}$), 70.3 (d, *J* = 49.2 Hz, Ph_3*C*).

The ^{1}H and ^{13}C NMR resonances are consistent with those previously reported.[2]

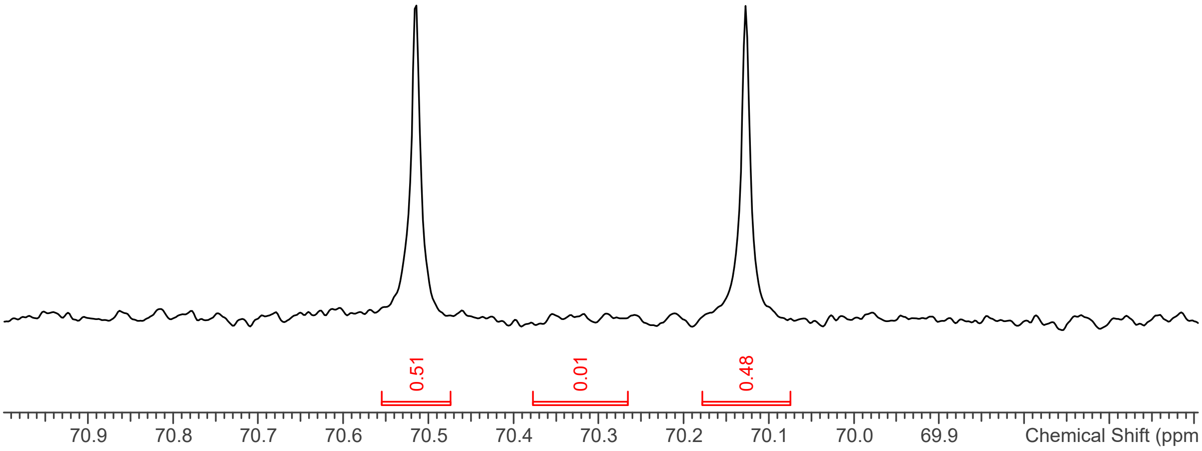

Figure 4: ^{13}C incorporation for $Ph_3C^{13}CO_2K$.

The ^{13}C incorporation was found to be >99% by quantitative ^{13}C NMR.

9.3. Synthesis of isotope labeled compounds

Riluzole derivative (3x)

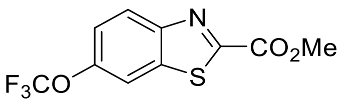

Following general procedure 2 (page S15) with 2-bromo-6-trifluoromethoxybenzothiazole (298 mg, 1.00 mmol, 1.00 equiv.). Product was obtained as a white crystalline solid. Column eluent 0% to 25% Et_2O in hexane.

Yield: 216 mg (78%, 0.78 mmol)

R$_f$: 0.22 (4:1 Hex:Et_2O).

^{1}H NMR (400 MHz, $CDCl_3$): δ 8.24 (dd, 1H, *J* = 9.0, 0.5 Hz, Ar*H*), 7.83 – 7.82 (m, 1H, Ar*H*), 7.46 – 7.43 (m, 1H, Ar*H*), 4.08 (s, 3H, CO_2CH_3).

^{13}C NMR (101 MHz, $CDCl_3$): δ 160.6 (CO_2CH_3), 159.2 (C_{Ar}), 151.5 (C_{Ar}), 148.3 (q, *J* = 2.2 Hz, C_{Ar}), 137.7 (C_{Ar}), 126.6 (CH_{Ar}), 121.2 (CH_{Ar}), 120.4 (q, *J* = 258.2 Hz, OCF_3), 114.2 (CH_{Ar}), 53.8 (CO_2CH_3).

^{19}F{^{1}H} NMR (376 MHz, $CDCl_3$): δ –58.02 (s, 3F, OCF_3).

HR-MS (ESI$^+$): Found *m/z* 278.0087, [M + H]$^+$; calculated for $[C_{10}H_7F_3NO_3S]^+$: 278.0093.

^{13}C-Labeled riluzole derivative (3x*)

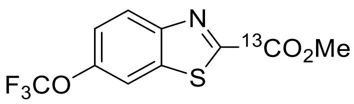

Following general procedure 2 (page S15) with 2-bromo-6-trifluoromethoxybenzothiazole (75.1 mg, 0.25 mmol, 1.00 equiv.), $Ph_3C^{13}CO_2K$ (167 mg, 0.51 mmol, 2.05 equiv.) and DMF (1.25 mL, 0.2 M). The work-up was conducted using $NaHCO_3$ (63.0 mg, 0.75 mmol, 3.00 equiv.), methyl iodide (250 µL, 4.00 mmol, 4.00 equiv) and DMF (1.25 mL, 0.2 M). Product was obtained as a white crystalline solid. Column eluent 0% to 25% Et_2O in hexane.

Yield: 50 mg (72%, 0.18 mmol)

%^{13}C incorporation: Found to be >99% by HR-MS.

R$_f$: 0.19 (4:1 Hex:Et_2O)

^{1}H NMR (400 MHz, $CDCl_3$): δ 8.25 (d, 1H, *J* = 9.1 Hz, Ar*H*), 7.84 (d, 1H, *J* = 1.1 Hz, Ar*H*), 7.47 – 7.44 (m, 1H, Ar*H*), 4.09 (d, 3H, *J* = 4.0 Hz, $^{13}CO_2CH_3$).

^{13}C NMR (101 MHz, $CDCl_3$): δ 160.6 ($^{13}CO_2CH_3$), 159.3 (d, *J* = 95.4 Hz, C_{Ar}), 151.5 (d, *J* = 8.8 Hz, C_{Ar}), 148.3 (q, *J* = 1.5 Hz, C_{Ar}), 137.7 (C_{Ar}), 126.7 (CH_{Ar}), 121.3 (CH_{Ar}), 120.4 (q, *J* = 259.0 Hz, OCF_3), 114.2 (CH_{Ar}), 53.8 (d, *J* = 2.2 Hz, $^{13}CO_2CH_3$).

^{19}F{^{1}H} NMR (376 MHz, $CDCl_3$): δ –58.00 (s, 3F, OCF_3).

HR-MS (ESI$^+$): Found *m/z* 279.0127, $[M + H]^+$; calculated for $[C_9{}^{13}CH_7F_3NO_3S]^+$: 279.0127.

Edoxaban precursor (3y)

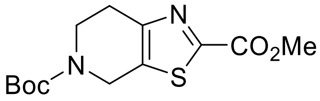

Following general procedure 2 (page S15) with *tert*-butyl 2-bromo-6,7-dihydrothiazolo[5,4-*c*]pyridine-5(4*H*)-carboxylate (319 mg, 1.00 mmol, 1.00 equiv.). The carboxylation step was run for 1 h. The product was isolated as a pale-yellow solid. Column eluent 0% to 60% Et_2O in hexane.

Yield: 224 mg (75%, 0.75 mmol)

R$_f$: 0.13 (3:2 Hex:Et_2O)

^{1}H NMR (500 MHz, DMSO-d$_6$): δ 4.70 (t, 2H, *J* = 1.6 Hz, CH_2), 3.89 (s, 3H, CO_2CH_3), 3.68 (t, 2H, *J* = 5.8 Hz, CH_2), 2.85 (tt, 2H, *J* = 6.0, 1.6 Hz, CH_2), 1.43 (s, 9H, $C(CH_3)_3$).

^{13}C NMR (125 MHz, DMSO-d$_6$): δ 160.5 (CO_2Me), 155.1 (C_{Ar}), 154.5 ($CO_2{}^tBu$), 152.0 (C_{Ar}), 133.5 (C_{Ar}), 80.2 ($C(CH_3)_3$), 53.2 (CO_2CH_3), 42.6 (CH_2), 41.6 (CH_2), 28.6 $C(CH_3)_3$), 27.1 (CH_2).

HR-MS (ESI$^+$): Found *m/z* 299.1061, $[M + H]^+$; calculated for $[C_{13}H_{19}N_2O_4S]^+$: 299.1060.

X-Ray Crystallography (3y, CCDC: 2371571):

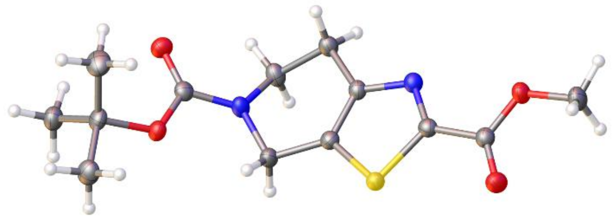

Experimental. Single clear colourless block-shaped crystals of **3y** recrystallised from Et2O by slow evaporation. A suitable crystal with dimensions 0.14 × 0.06 × 0.04 mm^3 was selected and mounted on a MITIGEN holder with silicon oil on a ROD, Synergy Custom system, HyPix-Arc 100 diffractometer. The crystal was kept at a steady T = 100(2) K during data collection. The structure was solved with the ShelXT 2014/5 (Sheldrick, 2014) solution program using dual methods and by using Olex2 1.5-alpha (Dolomanov et al., 2009) as the graphical interface. The model was refined with ShelXL 2016/6 (Sheldrick, 2015) using full matrix least squares minimisation on F^2.

Crystal Data. $C_{13}H_{18}N_2O_4S$, M_r = 298.35, monoclinic, $P2_1$ (No. 4), a = 8.9511(2) Å, b = 6.22610(10) Å, c = 13.2381(2) Å, b = 101.295(2)°, a = g = 90°, V = 723.48(2) $Å^3$, T = 100(2) K, Z = 2, Z' = 1, m(Cu K_a) = 2.132, 10599 reflections measured, 2582 unique (R_{int} = 0.0161) which were used in all calculations. The final wR_2 was 0.0518 (all data) and R_1 was 0.0197 (I≥2 s(I)).

^{13}C-Labeled edoxaban precursor (3y*)

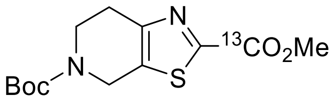

Following general procedure 2 (page S15) with *tert*-butyl 2-bromo-6,7-dihydrothiazolo[5,4-*c*]pyridine-5(4*H*)-carboxylate (80 mg, 0.25 mmol, 1.00 equiv.), $Ph_3C^{13}CO_2K$ (167 mg, 0.51 mmol, 2.05 equiv.) and DMF (1.25 mL, 0.2 M). The work-up was conducted using $NaHCO_3$ (63.0 mg, 0.75 mmol, 3.00 equiv.), methyl iodide (250 µL, 4.00 mmol, 4.00 equiv) and DMF (1.25 mL, 0.2 M). The carboxylation step was run for 1 h. The product was isolated as a pale orange solid. Column eluent 0% to 60% Et_2O in hexane.

Yield: 48 mg (65%, 0.16 mmol)

%^{13}C incorporation: Found to be >99% by HR-MS.

R_f: 0.12 (Hex:Et_2O 3:2)

^{1}H NMR (500 MHz, DMSO-d_6): δ 4.70 (t, 2H, J = 1.6 Hz, CH_2), 3.91 (d, 3H, J = 4.0 Hz, $^{13}CO_2CH_3$), 3.72 (t, 2H, J = 5.9 Hz, CH_2), 2.87 (tt, 2H, J = 5.9, 1.7 Hz, CH_2), 1.45 (s, 9H, C(CH_3)$_3$).

^{13}C NMR (125 MHz, DMSO-d_6): δ 160.5 ($^{13}CO_2$Me), 155.1 (d, J = 98.2 Hz, C_{Ar}), 154.5 ($CO_2{}^t$Bu), 152.0 (d, J = 8.2 Hz, C_{Ar}), 133.5 (C_{Ar}), 80.2 (C(CH_3)$_3$), 53.2 ($^{13}CO_2CH_3$), 42.6 (CH_2), 41.6 (CH_2), 28.6 (C(CH_3)$_3$), 27.1 (CH_2).

HR-MS (ESI$^+$): Found m/z 300.1090, [M + H]$^+$; calculated for $[C_{12}{}^{13}CH_{18}N_2O_4S]^+$: 300.1094.

General procedure 3 for the synthesis of befuraline derivative (3z)

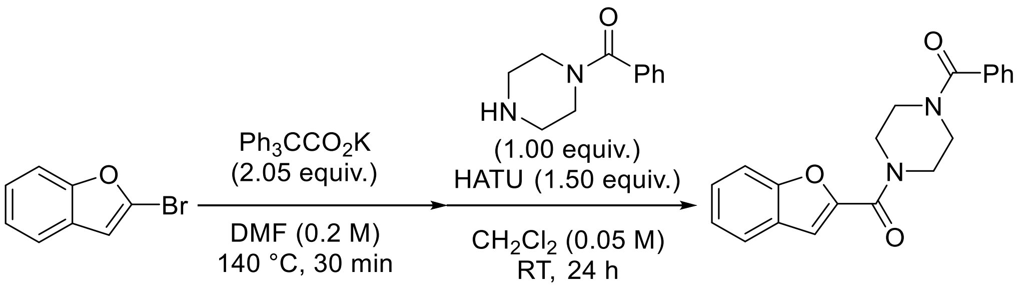

A Biotage® microwave vial was equipped with a magnetic stirrer bar and septum and dried with a heat-gun under *vacuo*. The vial was then charged with Ph_3CCO_2K (166 mg, 0.51 mmol, 2.05 equiv.) and placed under a nitrogen atmosphere through cycles of vacuum/nitrogen gas. DMF (1.25 mL, 0.2 M) and 2-bromobenzofuran (32 µL, 0.25 mmol, 1.00 equiv.) were then added to the vial. Under an N_2 gas flow, the septum was replaced with a Biotage® crimp cap. The reaction mixture was stirred at 140 °C for 30 mins. After this time, $NaHCO_3$ (63 mg, 0.75 mmol, 3.00 equiv.) was added and stirred for a further 2 min. The reaction was cooled to room temperature and quenched with 10 mL H_2O and extracted with Et_2O (3 × 10 mL). The aqueous phase was then concentrated under *vacuo* and dried overnight. The solid obtained was then dispersed in anhydrous CH_2Cl_2 (5.0 mL, 0.05 M), followed by the addition of 1-[Bis(dimethylamino)methylene]-1*H*-1,2,3-triazolo[4,5-*b*]pyridinium 3-oxid hexafluorophosphate (HATU, 144 mg, 0.38 mmol, 1.50 equiv.). After the reaction was

stirred at room temperature for 30 min, 1-Benzoylpiperazine (48 mg, 0.25 mmol, 1.00 equiv.) was added and stirred at room temperature for 24 h. The mixture was then diluted with brine (20 mL) and extracted with CH_2Cl_2 (3 × 20 mL). The combined organic phases were then dried over Na_2SO_4, concentrated under vacuum and purified using column chromatography (gradient: 0% to 80% EtOAc in Hex, 5% NEt_3) to give the product as a yellow solid.

Yield: 66 mg (80%, 0.20 mmol)

R_f: 0.28 (2:0.95:0.5 EtOAc:Hex:NEt_3)

^{1}H NMR (500 MHz, DMSO-d_6): δ 7.74 (ddd, 1H, *J* = 7.8, 1.3, 0.7 Hz, Ar*H*), 7.61 (app dq, 1H, *J* = 8.3, 1.3 Hz, Ar*H*), 7.48 – 7.42 (m, 6H, Ar*H*), 7.37 (d, 1H, *J* = 0.9 Hz, Ar*H*), 7.33 (ddd, 1H, *J* = 7.8, 7.2, 0.9 Hz, Ar*H*), 3.82 – 3.80 (m, 4H, C*H*$_2$), 3.64 – 3.62 (m, 4H, C*H*$_2$).

^{13}C NMR (DMSO-d_6): δ 170.1 (*C*O), 159.9 (*C*O), 154.7 (*C*$_{Ar}$), 149.0 (*C*$_{Ar}$), 136.4 (*C*$_{Ar}$), 130.0 (*C*H$_{Ar}$), 128.8 (*C*H$_{Ar}$), 127.4 (*C*H$_{Ar}$), 127.3 (*C*$_{Ar}$), 126.9 (*C*H$_{Ar}$), 124.1 (*C*H$_{Ar}$), 122.8 (*C*H$_{Ar}$), 112.1 (*C*H$_{Ar}$), 111.3 (*C*H$_{Ar}$), 44.9 (*C*H$_2$), 44.8 (*C*H$_2$).

HR-MS (ESI$^+$): Found *m/z* 335.1395, [M + H]$^+$; calculated for $[C_{20}H_{19}N_2O_3]^+$: 335.1390.

^{13}C-labeled befuraline derivative (3z*)

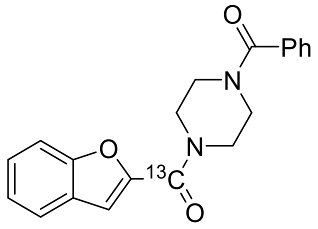

Following general procedure 3 with 2-bromobenzofuran (32 µL, 0.25 mmol, 1.00 equiv.). The compound was obtained as a yellow solid.

Yield: 67 mg (78%, 0.20 mmol).

%^{13}C incorporation: Found to be >99% by HR-MS.

^{1}H NMR (500 MHz, DMSO-d_6): δ 7.74 (ddd, 1H, *J* = 7.6, 1.7, 0.8 Hz, Ar*H*), 7.62 (app dq, 1H, *J* = 8.4, 0.9 Hz, Ar*H*), 7.49 – 7.43 (m, 6H, Ar*H*), 7.39 (t, 1H, *J* = 0.9 Hz, Ar*H*), 7.35 – 7.32 (m, 1H, Ar*H*), 3.82 – 3.79 (m, 4H, 2C*H*$_2$), 3.65 – 3.62 (m, 4H, 2C*H*$_2$).

^{13}C NMR (DMSO-d_6): δ 169.8 (*C*O), 159.8 (^{13}*C*O), 154.6 (d, *J* = 3.3 Hz, C_{Ar}), 148.9 (d, *J* = 83.0 Hz, C_{Ar}), 136.4 (C_{Ar}), 130.0 (CH_{Ar}), 128.8 (CH_{Ar}), 127.4 (CH_{Ar}), 127.3 (d, *J* = 3.6 Hz, C_{Ar}), 126.9 (CH_{Ar}), 124.1 (CH_{Ar}), 122.8 (CH_{Ar}), 112.2 (CH_{Ar}), 111.4 (d, *J* = 6.2 Hz, CH_{Ar}), 44.8 (broad peak, 2 × CH_2).

HR-MS (ESI$^+$): Found *m/z* 336.1431, [M + H]$^+$; calculated for $[C_{19}{}^{13}CH_{19}N_2O_3]^+$: 336.1424.

9.4. Carbon Isotope Exchange

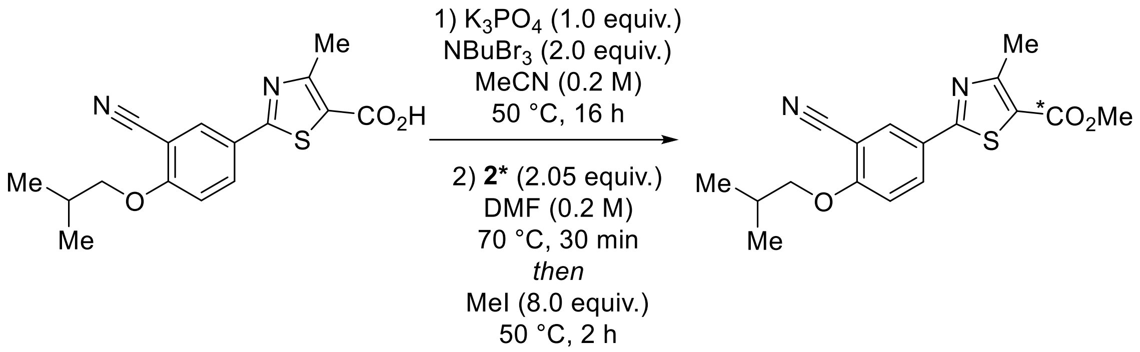

Preparation of 5-(5-Bromo-4-methylthiazol-2-yl)-2-isobutoxybenzonitrile (1aa-Br)

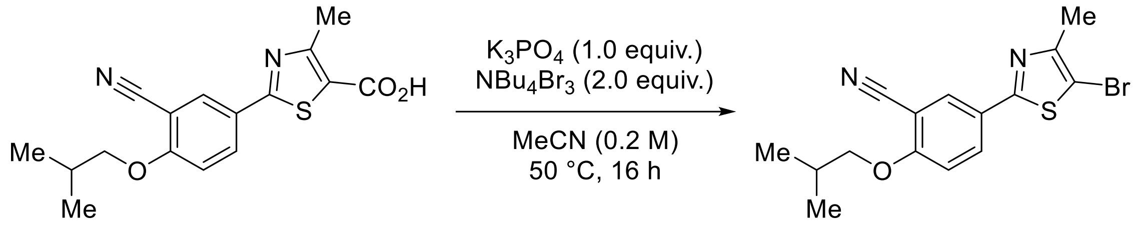

Adapted from the works of Larrosa et al.[15] A Biotage® microwave vial was equipped with a magnetic stirrer bar and septum and dried with a heat-gun under vacuum. The vial was then charged with febuxostat (316 mg, 1.00 mmol, 1.0 equiv.), NBu_4Br_3 (964 mg, 2.00 mmol, 2.0 equiv.) and anhydrous K_3PO_4 (212 mg, 1.00 mmol, 1.0 equiv.) and placed under a nitrogen atmosphere through cycles of vacuum/nitrogen gas. Anhydrous MeCN (5.0 mL, 0.2 M) was added and, under a flow of nitrogen gas, the septum was replaced with a crimp cap and firmly sealed using a Biotage® crimper. The mixture was then allowed to stir at 50 °C for 16 hours. On completion of the reaction, the mixture was cooled to room temperature, then 15% $Na_2S_2O_3$ (aq., 20.0 mL) and sat. Na_2CO_3 (aq., 20 mL) were added. The organic phase was collected by washing with CH_2Cl_2 (3 × 15 mL), dried over Na_2SO_4 and concentrated under *vacuo*. The mixture was dissolved in pentane/EtOAc (20.0 mL, 98:2) and filtered through a short plug of celite with further washings of pentane/EtOAc (4 × 40 mL, 98:2). Removal of the solvent under vacuum gave the product as a white solid.

Yield: 263 mg (75%, 0.75 mmol).

^{1}H NMR (400 MHz, $CDCl_3$): δ 8.04 (d, 1H, *J* = 2.3 Hz, Ar*H*), 7.98 – 7.95 (m, 1H, Ar*H*), 6.99 (d, 1H, *J* = 8.9 Hz, Ar*H*), 3.88 (d, 2H, *J* = 6.5 Hz, OC*H*$_2$), 2.44 (s, 3H, C*H*$_3$), 2.21 (app. non., 1H, *J* = 6.6 Hz, C*H*(CH_3)$_2$), 1.09 (d, 6H, *J* = 6.6 Hz, CH(C*H*$_3$)$_2$).

^{13}C NMR (101 MHz, $CDCl_3$): δ 164.3 (*C*$_{Ar}$), 162.0 (*C*$_{Ar}$), 152.9 (*C*$_{Ar}$), 131.8 (*C*H$_{Ar}$), 131.3 (*C*H$_{Ar}$), 126.4 (*C*$_{Ar}$), 115.5 (*C*N), 112.6 (*C*H$_{Ar}$), 104.1 (*C*$_{Ar}$), 102.0 (*C*$_{Ar}$), 75.7 (O*C*H$_2$), 28.2 (*C*H(CH_3)$_2$), 19.1 (CH(*C*H$_3$)$_2$), 15.7 (Ar*C*H$_3$).

HR-MS (ESI$^+$): Found *m/z* 251.0155, [M + H]$^+$; calculated for $[C_{15}H_{16}BrN_2OS]^+$: 351.0161.

Febuxostat ester (3aa)

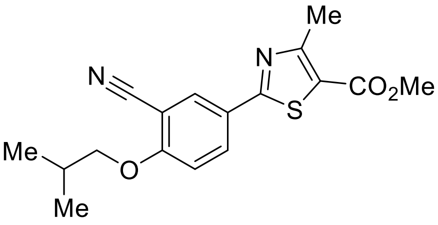

Following general procedure 2 (page S15) with 5-(5-bromo-4-methylthiazol-2-yl)-2-isobutoxybenzonitrile (89 mg, 0.25 mmol, 1.00 equiv.), Ph_3CCO_2K (166 mg, 0.51 mmol, 2.05 equiv.) and DMF (1.25 mL, 0.2 M). The work-up was conducted using $NaHCO_3$ (63.0 mg, 0.75 mmol, 3.00 equiv.), methyl iodide (250 μL, 4.00 mmol, 4.00 equiv) and DMF (1.25 mL, 0.2 M). The product was isolated as a white solid. Column eluent: 0% to 50% Et_2O in hexane.

Yield: 56 mg (68%, 0.17 mmol)

R$_f$: 0.20 (3:1 Hex:Et_2O)

^{1}H NMR (400 MHz, $CDCl_3$): δ 8.17 (d, 1H, *J* = 2.2 Hz, Ar*H*), 8.09 (dd, 1H, *J* = 8.9, 2.3 Hz, Ar*H*), 7.01 (d, 1H, *J* = 8.9 Hz, Ar*H*), 3.91 – 3.89 (m, 5H, CO_2C*H*$_3$/OC*H*$_2$), 2.77 (s, 3H, ArC*H*$_3$), 2.21 (sept., 1H, *J* = 6.7 Hz, C*H*(CH_3)$_2$), 1.09 (d, 6H, *J* = 6.6 Hz, CH(C*H*$_3$)$_2$).

^{13}C NMR (101 MHz, $CDCl_3$): δ 167.3 (*C*$_{Ar}$), 162.5 (*C*$_{Ar}$), 162.5 (*C*O$_2$Me), 161.4 (*C*$_{Ar}$), 132.6 (*C*H$_{Ar}$), 132.1 (*C*H$_{Ar}$), 126.0 (*C*$_{Ar}$), 121.4 (*C*$_{Ar}$), 115.4 (*C*N), 112.6 (*C*H$_{Ar}$), 103.0 (*C*$_{Ar}$), 75.7 (O*C*H$_2$), 52.3 (CO_2*C*H$_3$), 28.2 (*C*H(CH_3)$_2$), 19.1 (CH(*C*H$_3$)$_2$), 17.5 (*C*H$_3$).

The ^{1}H and ^{13}C NMR resonances are consistent with those previously reported.[2]

^{13}C-Labeled febuxostat ester (3aa*)

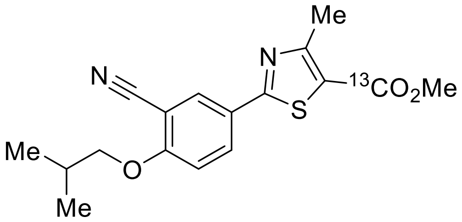

Following general procedure 2 (page S15) with 5-(5-bromo-4-methylthiazol-2-yl)-2-isobutoxybenzonitrile (89 mg, 0.25 mmol, 1.00 equiv.), $Ph_3C^{13}CO_2K$ (167 mg, 0.51 mmol, 2.05 equiv.) and DMF (1.25 mL, 0.2 M). The work-up was conducted using $NaHCO_3$ (63.0 mg, 0.75 mmol, 3.00 equiv.), methyl iodide (250 µL, 4.00 mmol, 4.00 equiv) and DMF (1.25 mL, 0.2 M). The product was isolated as a white solid. Column eluent: 0% to 50% Et_2O in hexane.

Yield: 53.0 mg (64%, 0.16 mmol)

%^{13}C incorporation: Found to be >99% by HR-MS.

R$_f$: 0.32 (4:1 Hex:EtOAc)

^{1}H NMR (500 MHz, $CDCl_3$): δ 8.18 (d, 1H, *J* = 2.1 Hz, Ar*H*), 8.10 (dd, 1H, *J* = 8.9, 2.3 Hz, Ar*H*), 7.01 (d, 1H, *J* = 9.0 Hz, Ar*H*), 3.91 – 3.89 (m, 5H, $^{13}CO_2CH_3$/OCH_2), 2.77 (d, 3H, *J* = 0.8 Hz, ArCH_3), 2.21 (app .septet, 1H, *J* = 6.6 Hz, C*H*(CH_3)$_2$), 1.10 (d, 6H, *J* = 6.8 Hz, CH(CH_3)$_2$).

^{13}C NMR (125 MHz, $CDCl_3$): δ 167.3 (d, *J* = 3.3 Hz, *C*$_{Ar}$), 162.5 (*C*$_{Ar}$), 162.5 ($^{13}CO_2CH_3$), 161.4 (d, *J* = 4.8 Hz, *C*$_{Ar}$), 132.6 (*C*H$_{Ar}$), 132.1 (*C*H$_{Ar}$), 126.0 (*C*$_{Ar}$), 121.4 (d, *J* = 88.0 Hz, *C*$_{Ar}$), 115.4 (C$_{Ar}$), 112.6 (*C*H$_{Ar}$), 103.0 (*C*$_{Ar}$), 75.7 (O*C*H_2), 52.3 (d, *J* = 2.4 Hz, $^{13}CO_2CH_3$), 28.2 (*C*H(CH_3)$_2$), 19.1 (CH(*C*H_3)$_2$), 17.5 (Ar*C*H_3).

The ^{1}H and ^{13}C NMR resonances are consistent with those previously reported.[2]

9.5. Recovery of 2,2,2-Triphenylacetic Acid

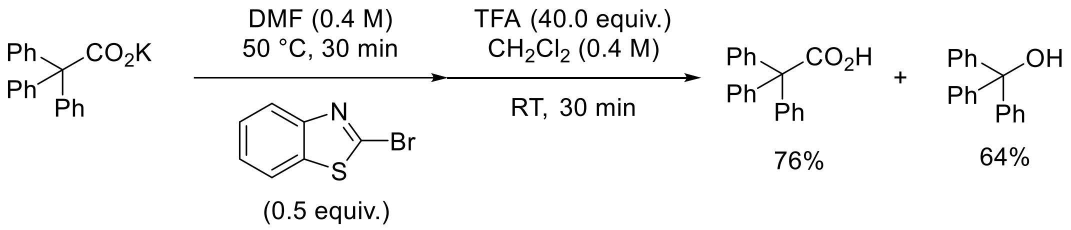

Adapted from the works of Perry et al.[2] and Ishihara et al.[16] A Biotage® microwave vial was equipped with a magnetic stirrer bar and septum and dried with a heat-gun under vacuum. The vial was then charged with 2-bromobenzothiazole (107 mg, 0.50 mmol, 1.00 equiv.) and Ph_3CCO_2K (326 mg, 1.00 mmol, 2.00 equiv.) and placed under a nitrogen atmosphere through cycles of vacuum/nitrogen gas. DMF (2.5 mL, 0.4 M) was then added to the vial. Under an N_2 gas flow, the septum was replaced with a Biotage® crimp cap. The reaction mixture was stirred at 50 °C for 30 min. After this time, $NaHCO_3$ (126 mg, 1.50 mmol, 1.50 equiv.) was added and the reaction was stirred for a further 2 min and then cooled to room temperature. The mixture was then diluted with 10 mL H_2O and extracted with Et_2O (3 × 10 mL), dried over Na_2SO_4 and concentrated under vacuum. The crude mixture was then dissolved in CH_2Cl_2 (2.5 mL, 0.4 M) and TFA (1.5 mL, 20.0 mmol, 20.0 equiv.) was added. The mixture was stirred at room temperature for 30 min, concentrated under vacuum and purified using silica gel column chromatography. The desired acid was isolated using a 0% to 60% EtOAc in hexane eluent, the alcohol was isolated in a secondary column using an eluent of 0% to 15% Et_2O in hexane.

Yield Ph_3CCO_2H: 110 mg (76%, 0.38 mmol).

The spectral data matched that reported in section 2.1.

Yield Ph_3COH: 83 mg (64%, 0.32 mmol).

^{1}H NMR (400 MHz, $CDCl_3$):δ 7.35 – 7.27 (m, 15H, Ar*H*), 2.79 (s, 1H, OH).

^{13}C NMR (101 MHz, $CDCl_3$):δ 146.9 (*C*$_{Ar}$), 127.9 (*C*H$_{Ar}$), 127.9 (*C*H$_{Ar}$), 127.3 (*C*H$_{Ar}$), 82.0 (*C*OH).

The ^{1}H and ^{13}C NMR resonances are consistent with those previously reported.[17]

10. References

1. Burchat, A. F.; Chong, J. M.; Nielsen, N. Titration of Alkyllithiums with a Simple Reagent to a Blue Endpoint. *J. Organomet. Chem.* **1997**, *542*, 281–283.
2. Wang, S.; Larrosa, I.; Yorimitsu, H.; Perry, G. J. P. Carboxylic Acid Salts as Dual-Function Reagents for Carboxylation and Carbon Isotope Labeling. *Angew. Chem. Int. Ed.* **2023**, *62*, e202218371.
3. Chen, J.-H.; Deng, C.-H.; Fang, S.; Ma, J.-G.; Cheng, P. Binuclear Molybdenum Alkoxide as the Versatile Catalyst for the Conversion of Carbon Dioxide. *Green Chem.* **2018**, *20*, 989–996.
4. Inomata, H.; Ogata, K.; Fukuzawa, S.; Hou, Z. Direct C–H Carboxylation with Carbon Dioxide Using 1,2,3-Triazol-5-Ylidene Copper(I) Complexes. *Org. Lett.* **2012**, *14*, 3986–3989.
5. Panda, N.; Mothkuri, R. Synthesis of Substituted Oxazoles from Enamides. *New J. Chem.* **2014**, *38*, 5727–5735.
6. Valdez-Padilla, D.; Rodríguez-Morales, S.; Hernández-Campos, A.; Hernández-Luis, F.; Yépez-Mulia, L.; Tapia-Contreras, A.; Castillo, R. Synthesis and Antiprotozoal Activity of Novel 1-Methylbenzimidazole Derivatives. *Bioorg. Med. Chem.* **2009**, *17*, 1724–1730.
7. Sheela, K.; Santhosh, C.; Singh, K. R.; Sharath, K.; Sadashiva, M. P. An Efficient Synthesis of Mono-, Di-, and Tri-Substituted 1,3-Thiazoles Employing Functionalized Thioamides as Thiocarbonyl Precursors. *Org. Biomol. Chem.* **2024**, *22*, 3490–3501.
8. Coe, P. L.; Rees, A. J. Preparation and Reactions of 2,3,4,6-Tetrafluoropyridine and Its Derivatives. *J. Fluor. Chem.* **2000**, *101*, 45–60.
9. Collins, K. D.; Glorius, F. A Robustness Screen for the Rapid Assessment of Chemical Reactions. *Nat. Chem.* **2013**, *5*, 597–601. (b) Collins, K. D.; Rühling, A.; Glorius, F. Application of a Robustness Screen for the Evaluation of Synthetic Organic Methodology. *Nat. Protoc.* **2014**, *9*, 1348–1353.
10. (a) A. D. Becke, *J. Chem. Phys.* **1993**, *98*, 5648–5652. (b) C. Lee, W. Yang and R. G. Parr, *Phys. Rev. B* **1988**, *37*, 785–789.
11. Gaussian 16, Revision C.02, M. J. Frisch, G. W. Trucks, H. B. Schlegel, G. E. Scuseria, M. A. Robb, J. R. Cheeseman, G. Scalmani, V. Barone, G. A. Petersson, H. Nakatsuji, X. Li, M. Caricato, A. V. Marenich, J. Bloino, B. G. Janesko, R. Gomperts, B. Mennucci, H. P. Hratchian, J. V. Ortiz, A. F. Izmaylov, J. L. Sonnenberg, D. Williams-Young, F. Ding, F. Lipparini, F. Egidi, J. Goings, B. Peng, A. Petrone, T. Henderson, D. Ranasinghe, V. G. Zakrzewski, J. Gao, N. Rega, G. Zheng, W. Liang, M. Hada, M. Ehara, K. Toyota, R. Fukuda, J. Hasegawa, M. Ishida, T. Nakajima, Y. Honda, O. Kitao, H. Nakai, T. Vreven, K. Throssell, J. A. Montgomery, Jr., J. E. Peralta, F. Ogliaro, M. J. Bearpark, J. J. Heyd, E. N. Brothers, K. N. Kudin, V. N. Staroverov, T. A. Keith, R. Kobayashi, J. Normand, K. Raghavachari, A. P. Rendell, J. C. Burant, S. S. Iyengar, J. Tomasi, M. Cossi, J. M. Millam, M. Klene, C. Adamo, R. Cammi, J. W. Ochterski,

R. L. Martin, K. Morokuma, O. Farkas, J. B. Foresman, and D. J. Fox, Gaussian, Inc., Wallin

12. S. Grimme, J. Antony, S. Ehrlich and H. Krieg, *J. Chem. Phys.* **2010**, *132*, 154104.
13. A. V. Marenich, C. J. Cramer, D. G. Truhlar, *J. Phys. Chem. B* **2009**, *113*, 6378–6396.
14. C. Y. Legault, CYLview20; Unversité de Sherbrooke: Sherbrooke, Canada, **2020** (http://www.cylview.org).
15. Quibell, J. M.; Perry, G. J. P.; Cannas, D. M.; Larrosa, I. Transition-Metal-Free Decarboxylative Bromination of Aromatic Carboxylic Acids. *Chem. Sci.* **2018**, *9*, 3860–3865.
16. Uyanik, M.; Sahara, N.; Tsukahara, M.; Hattori, Y.; Ishihara, K. Chemo- and Enantioselective Oxidative α-Azidation of Carbonyl Compounds. *Angew. Chem. Int. Ed.* **2020**, *59*, 17110–17117.
17. Sarkar, W.; Szymczak, N. K. Expanding Perchlorate Use for C–H Oxidative Transformations: A Tandem Photo- and Iron-Catalytic Strategy. *Organometallics* **2025**, *44*, 777–782.

11. NMR Data

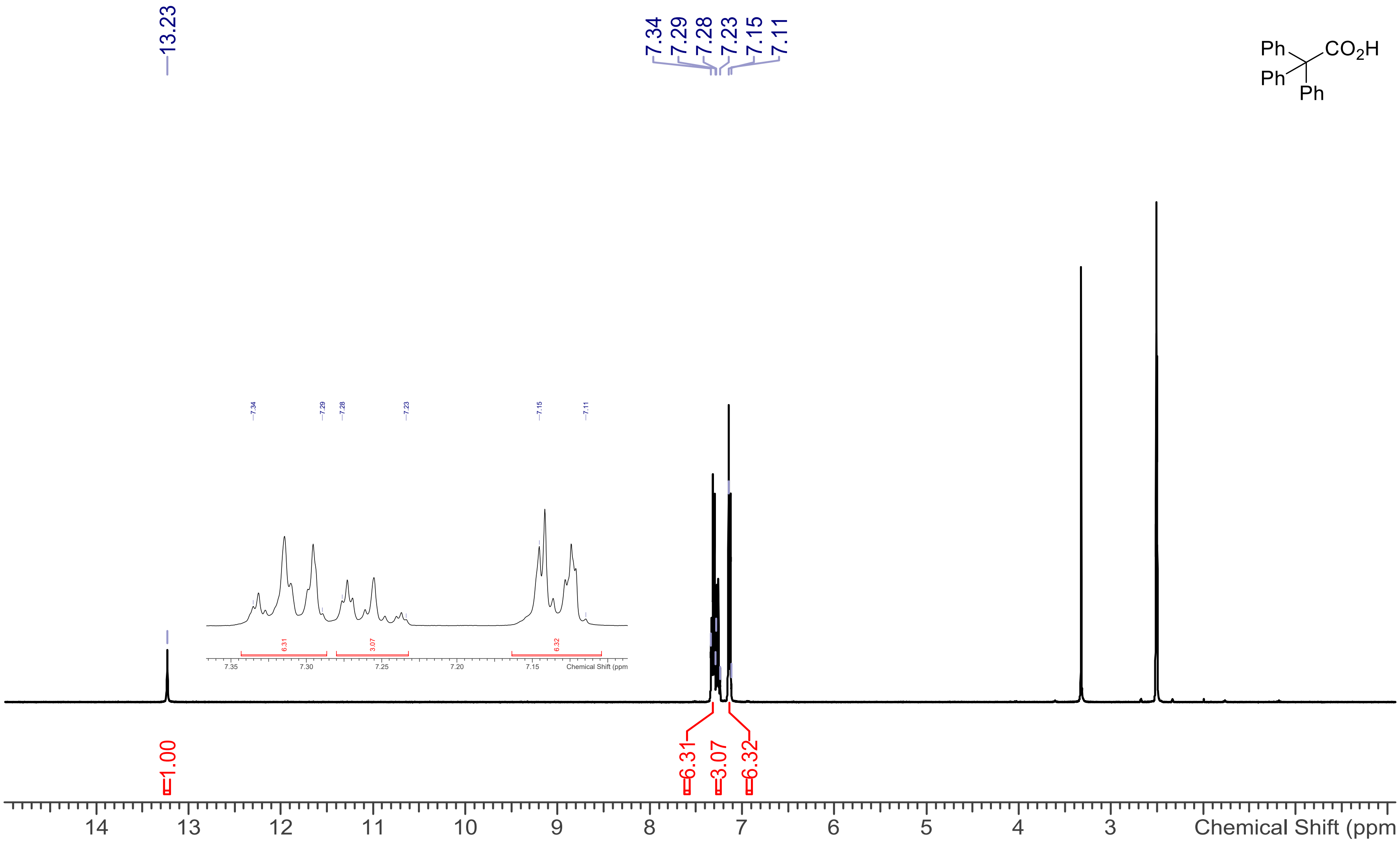

^{1}H NMR (400 MHz, DMSO-d6) Triphenylacetic acid (**2'**)

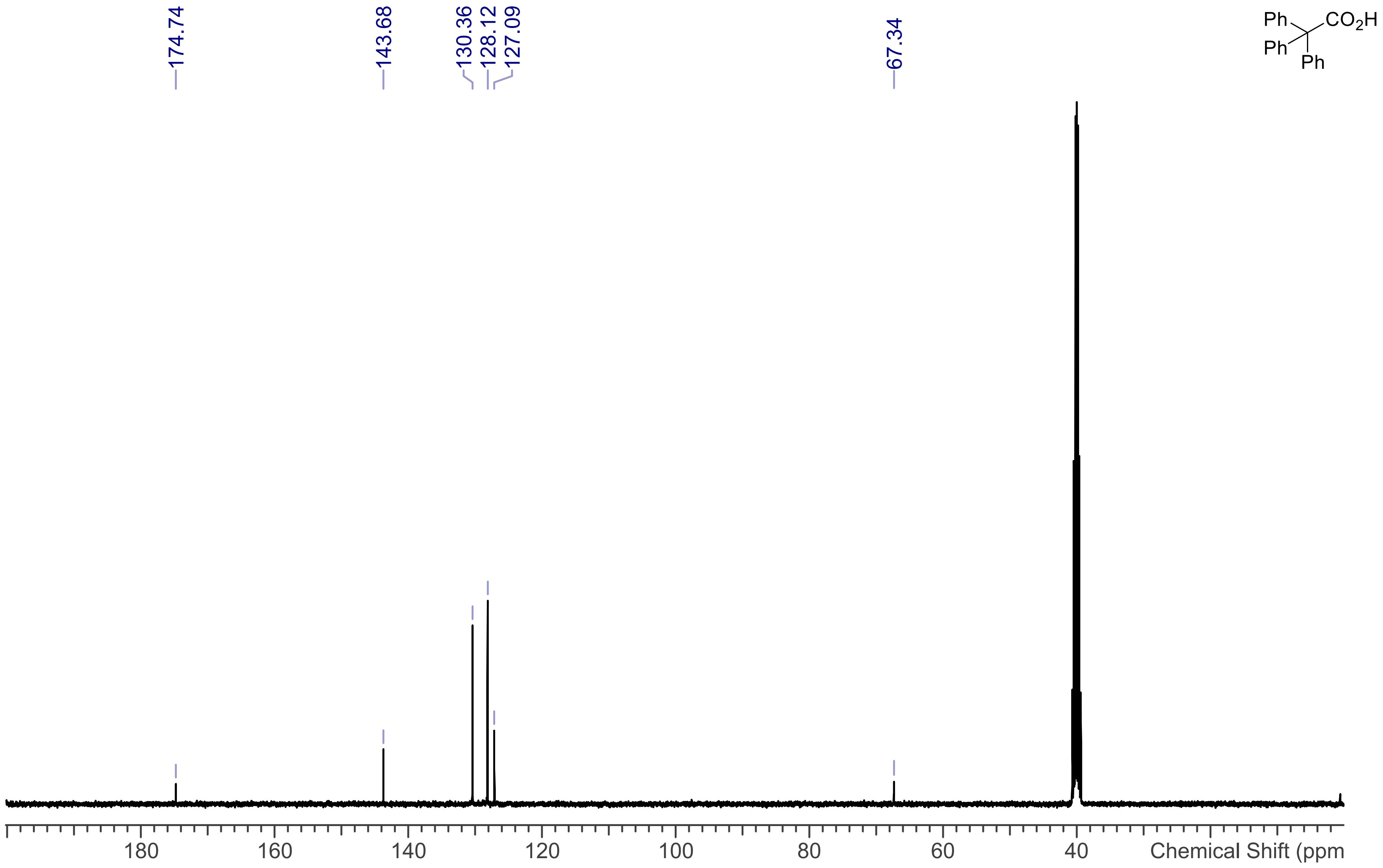

^{13}C NMR (101 MHz, DMSO-d_6) Triphenylacetic acid. (**2’**)

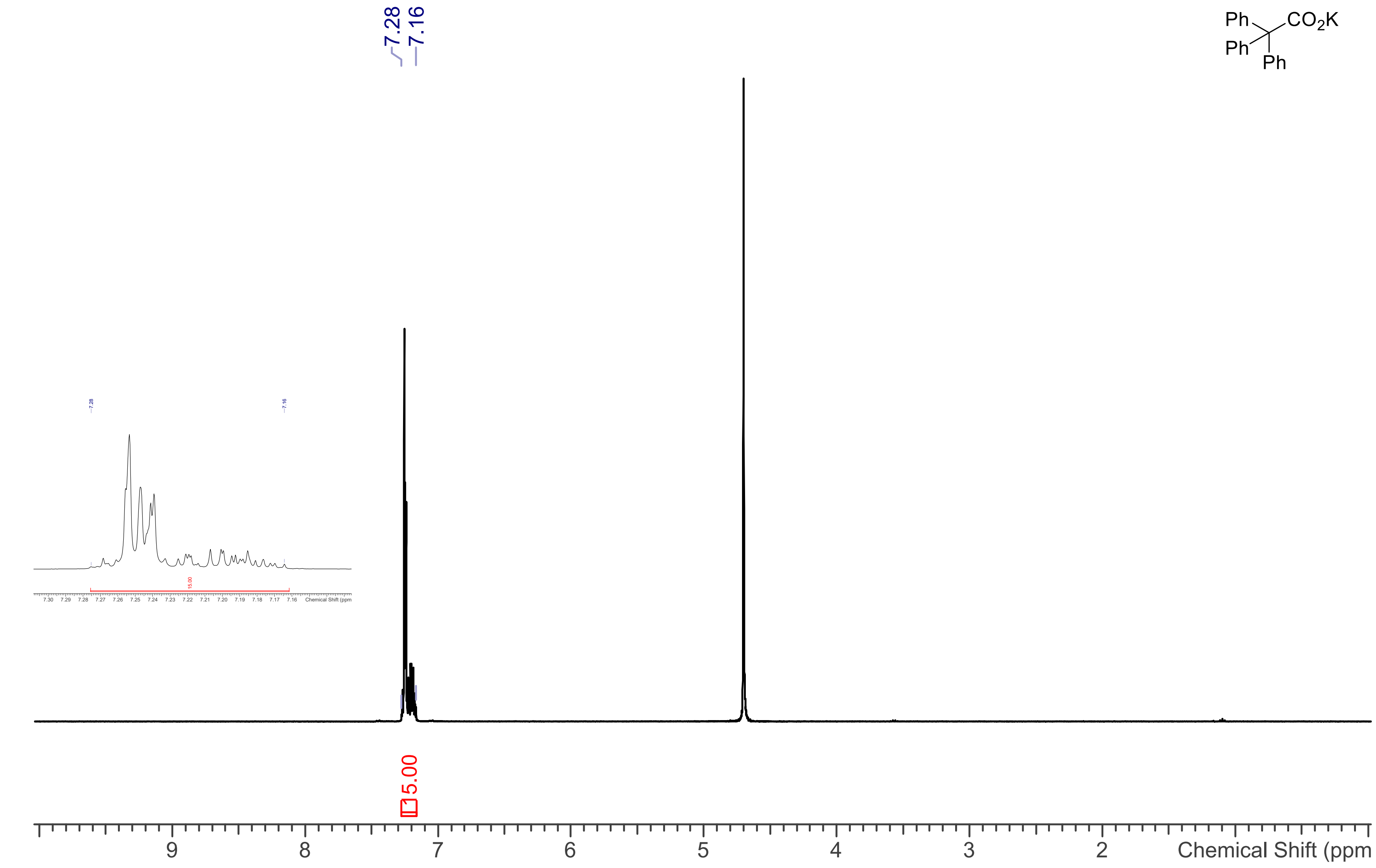

^{1}H NMR (400 MHz, D_2O) Potassium 2,2,2-triphenyl acetate (**2-K**)

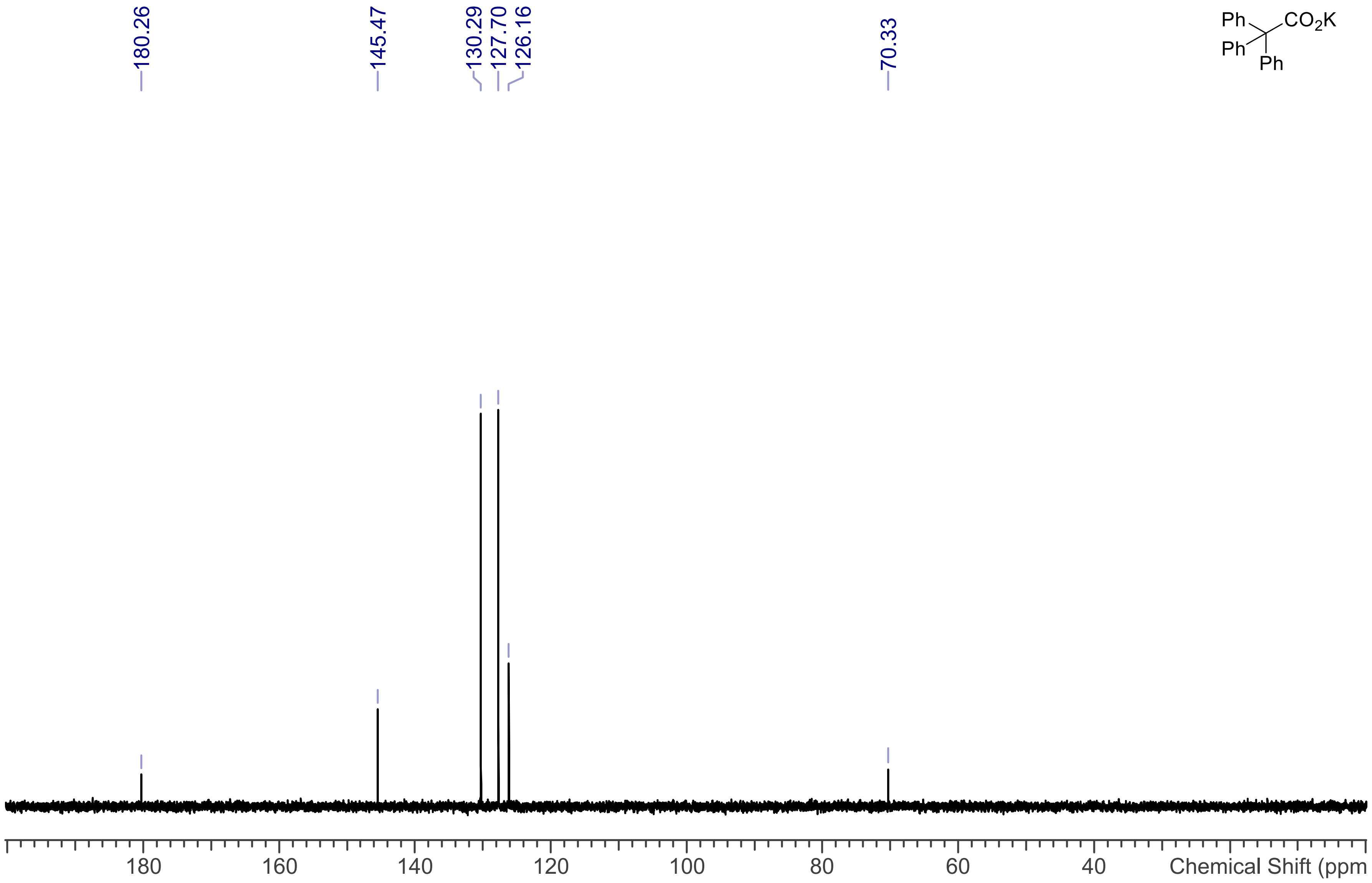

^{13}C NMR (101 MHz, D_2O) Potassium 2,2,2-triphenyl acetate (**2-K**).

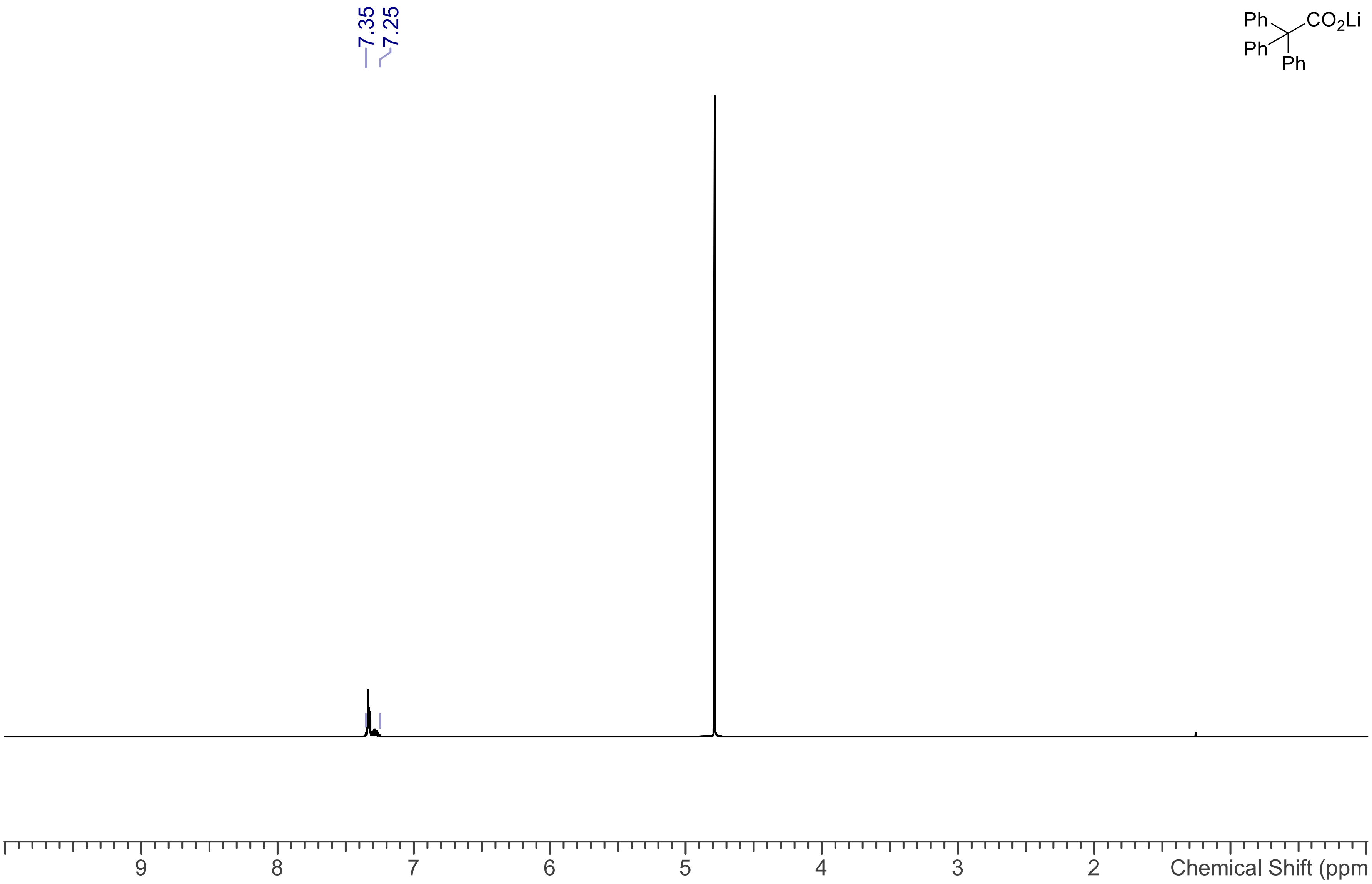

^{1}H NMR (400 MHz, D_2O) Lithium 2,2,2-triphenyl acetate (**2-Li**).

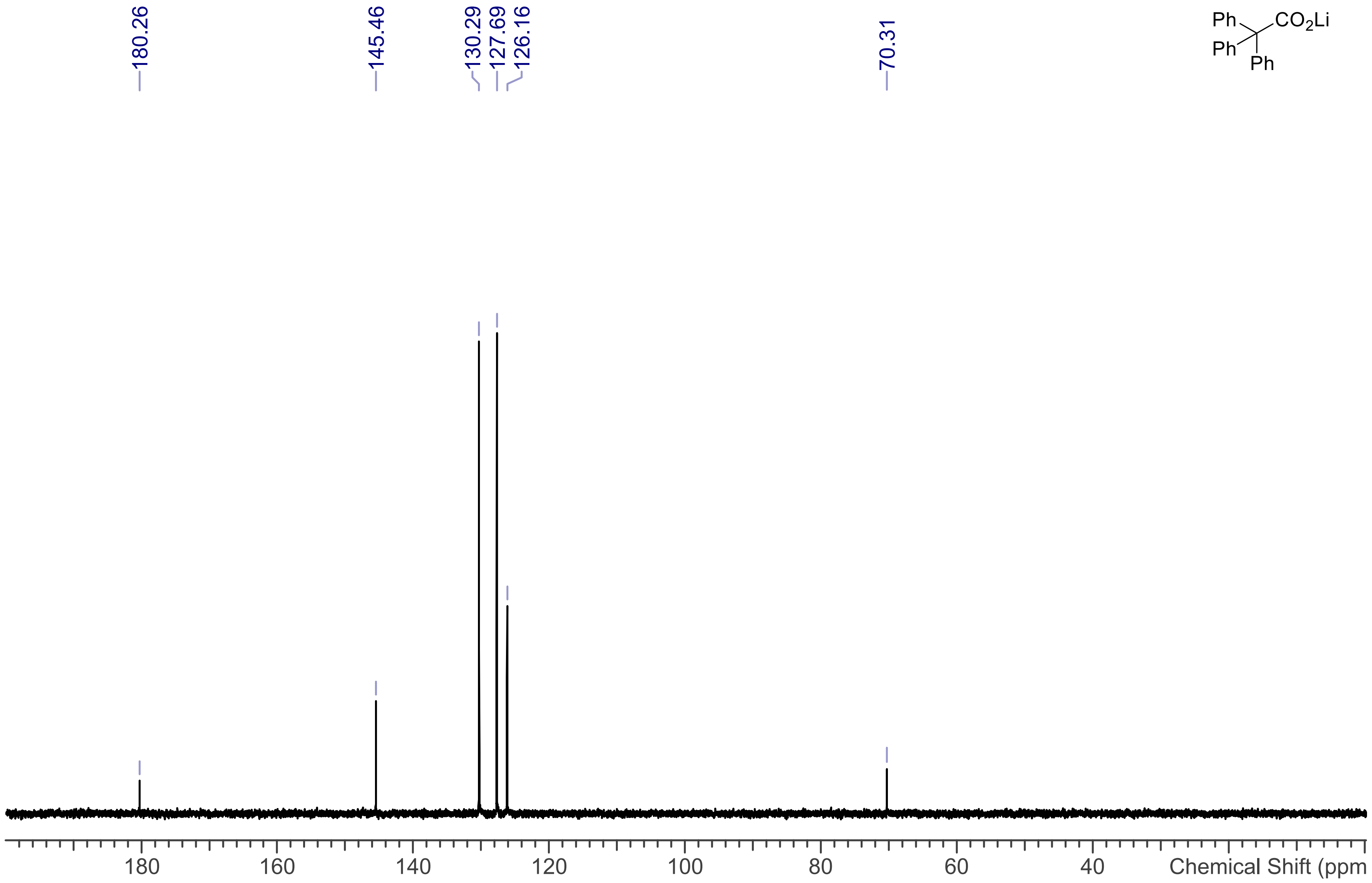

^{13}C NMR (101 MHz, D_2O) Lithium 2,2,2-triphenyl acetate (**2-Li**).

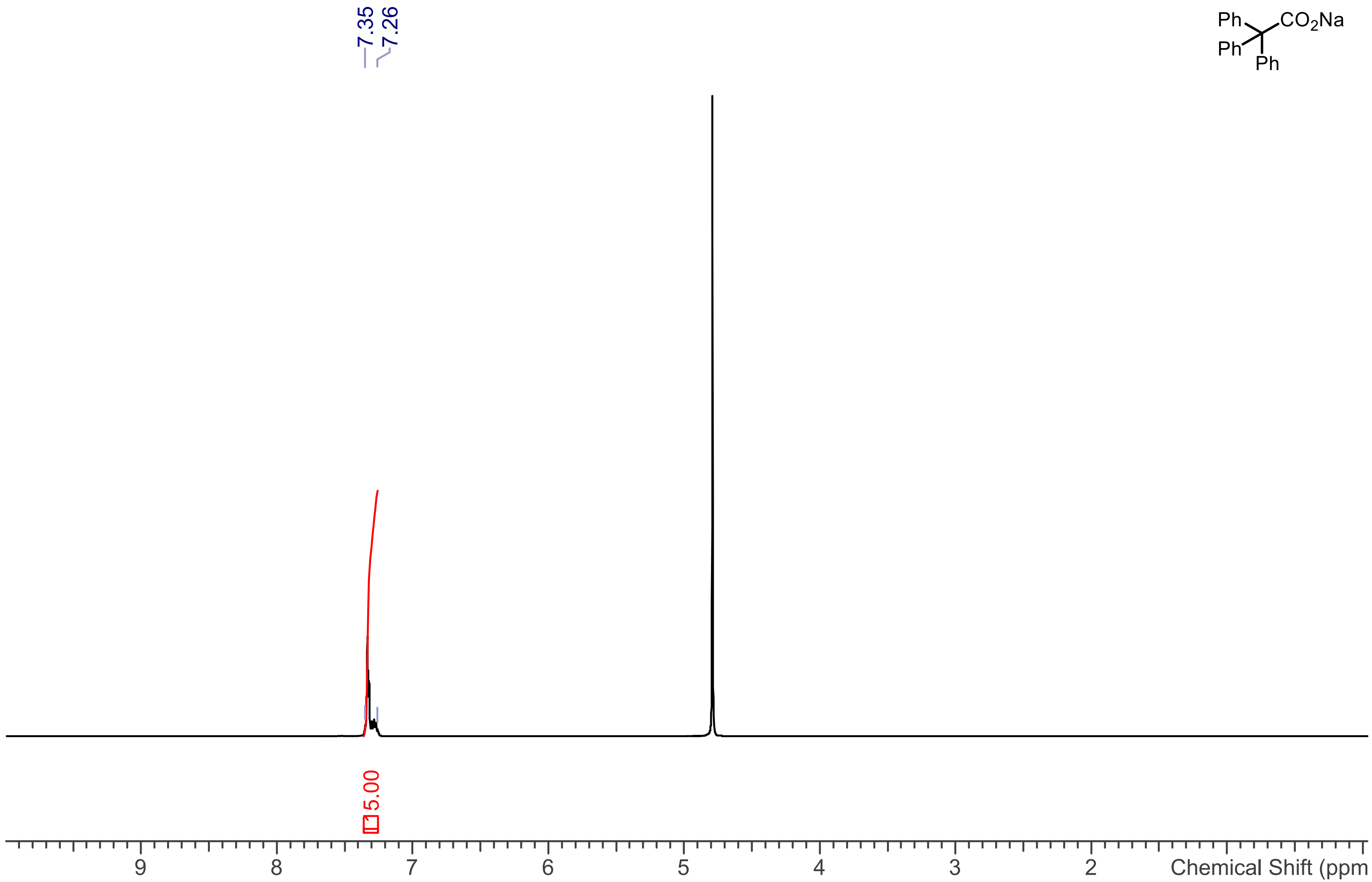

^{1}H NMR (400 MHz, D_2O) Sodium 2,2,2-triphenyl acetate (**2-Na**).

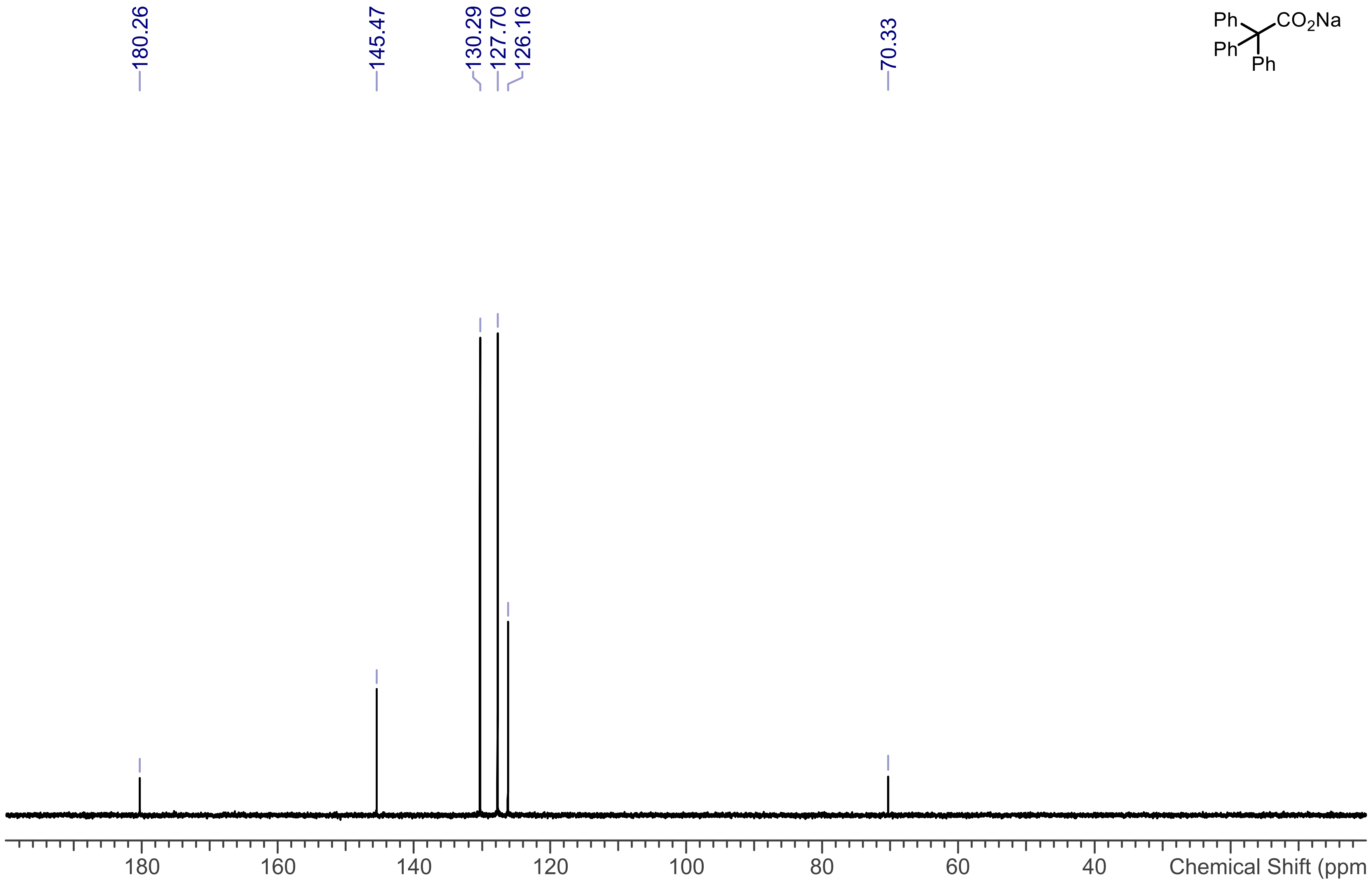

^{13}C NMR (101 MHz, D_2O) Sodium 2,2,2-triphenyl acetate (**2-Na**).

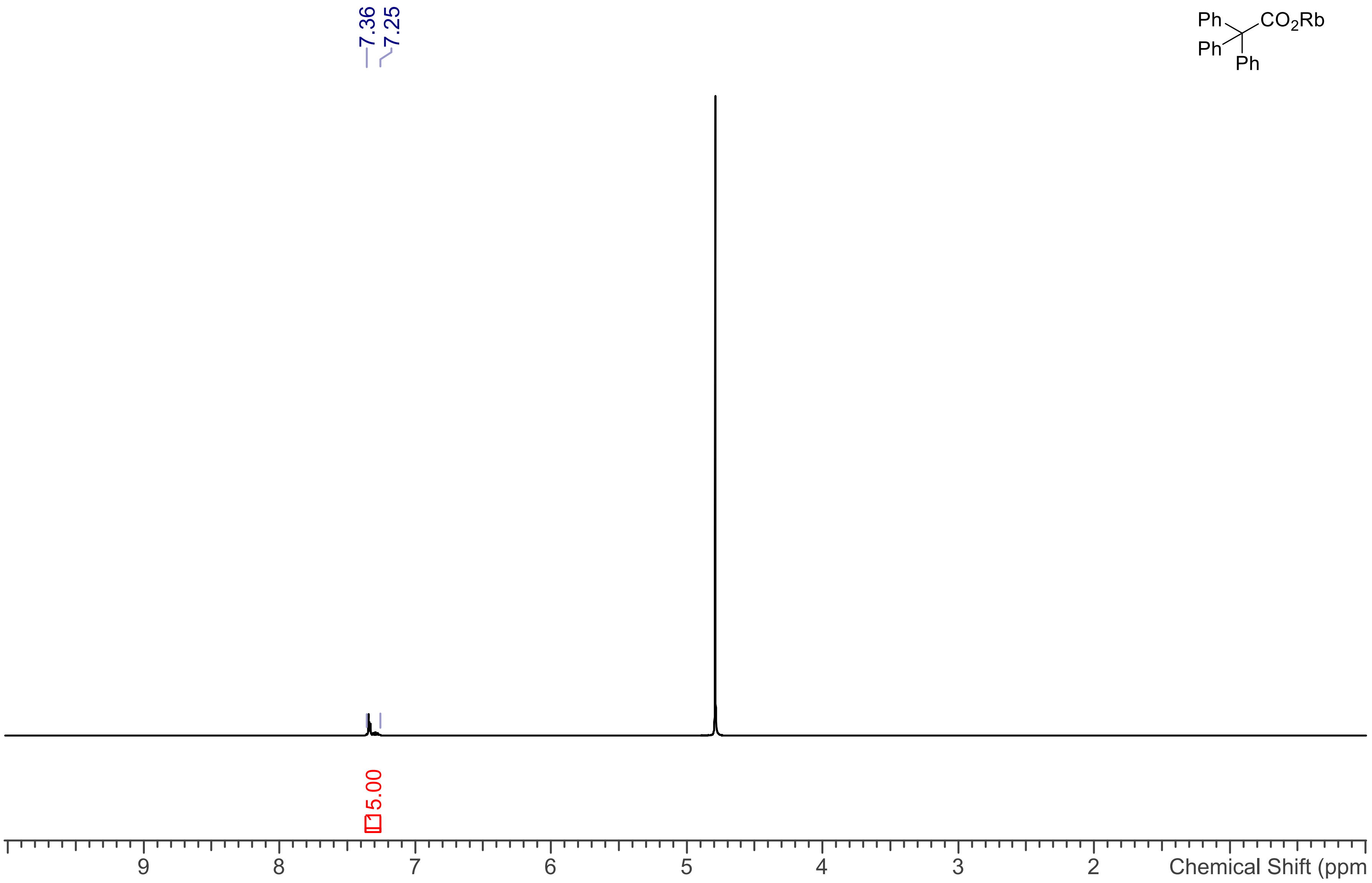

^{1}H NMR (400 MHz, D_2O) Rubidium 2,2,2-triphenylacetate (**2-Rb**).

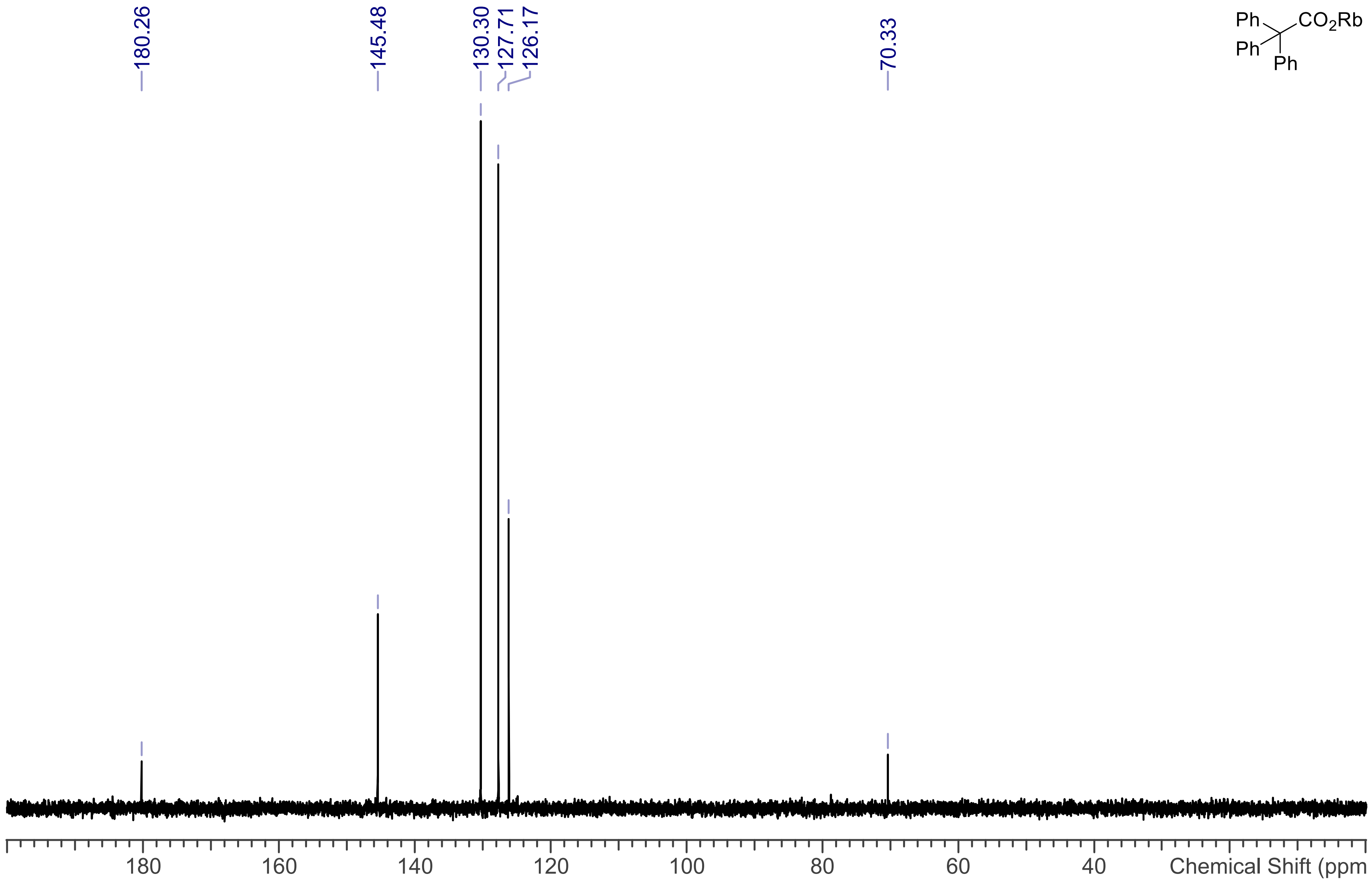

^{13}C NMR (101 MHz, D_2O) Rubidium 2,2,2-triphenylacetate (**2-Rb**).

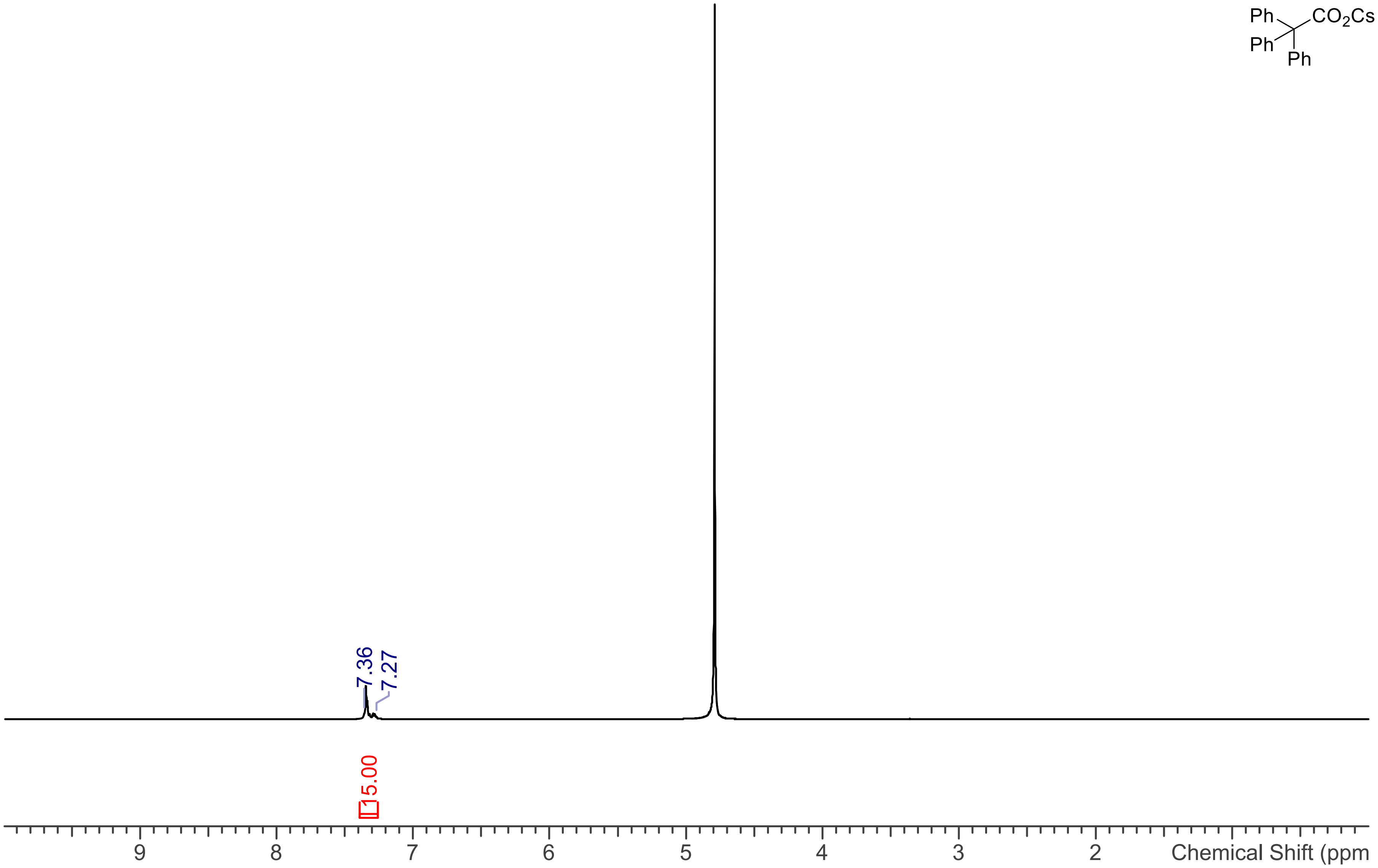

^{1}H NMR (500 MHz, D_2O) Caesium 2,2,2-triphenylacetate (**2-Cs**).

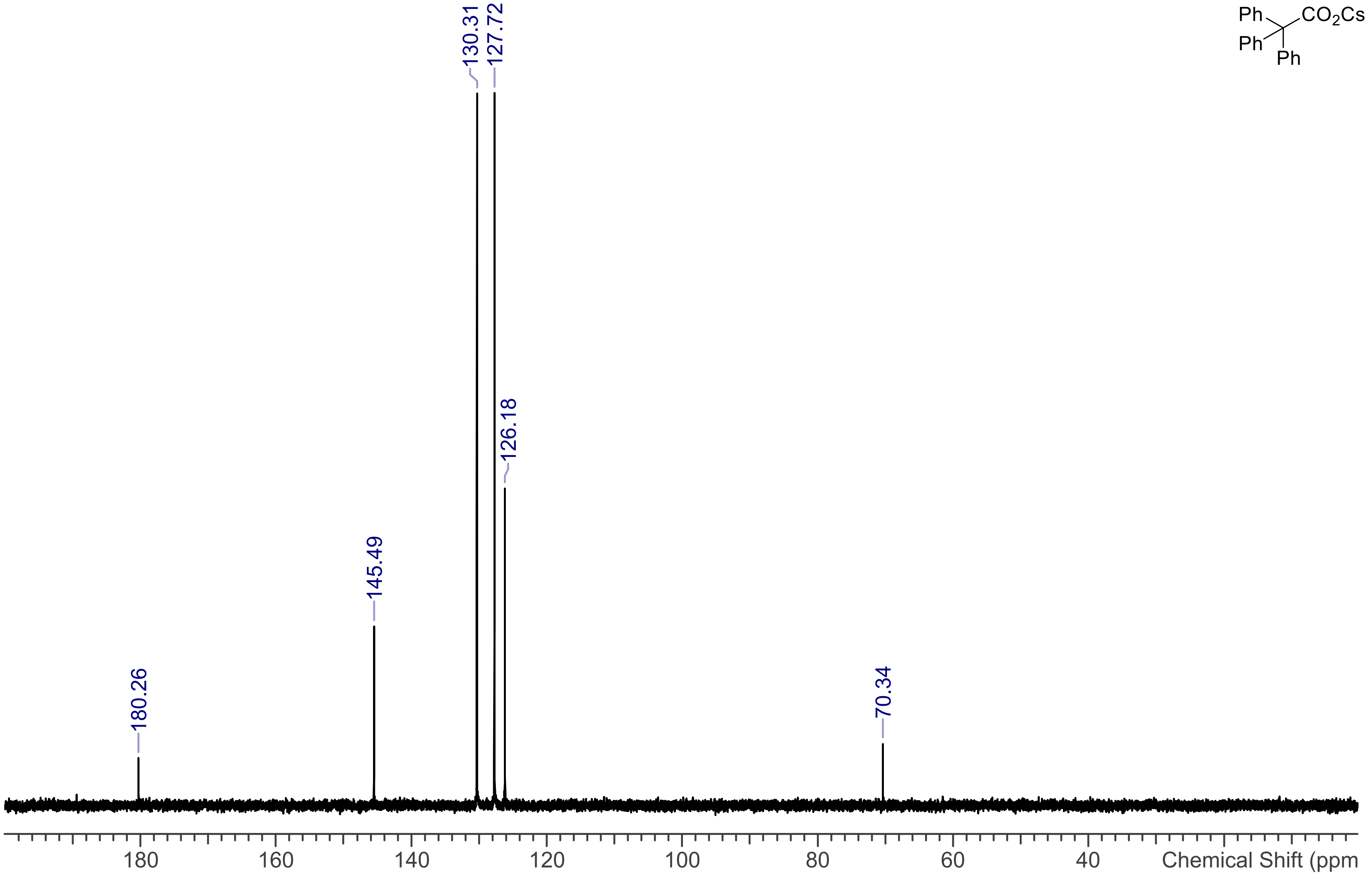

^{13}C NMR (126 MHz, D_2O) Caesium 2,2,2-triphenylacetate (**2-Cs**).

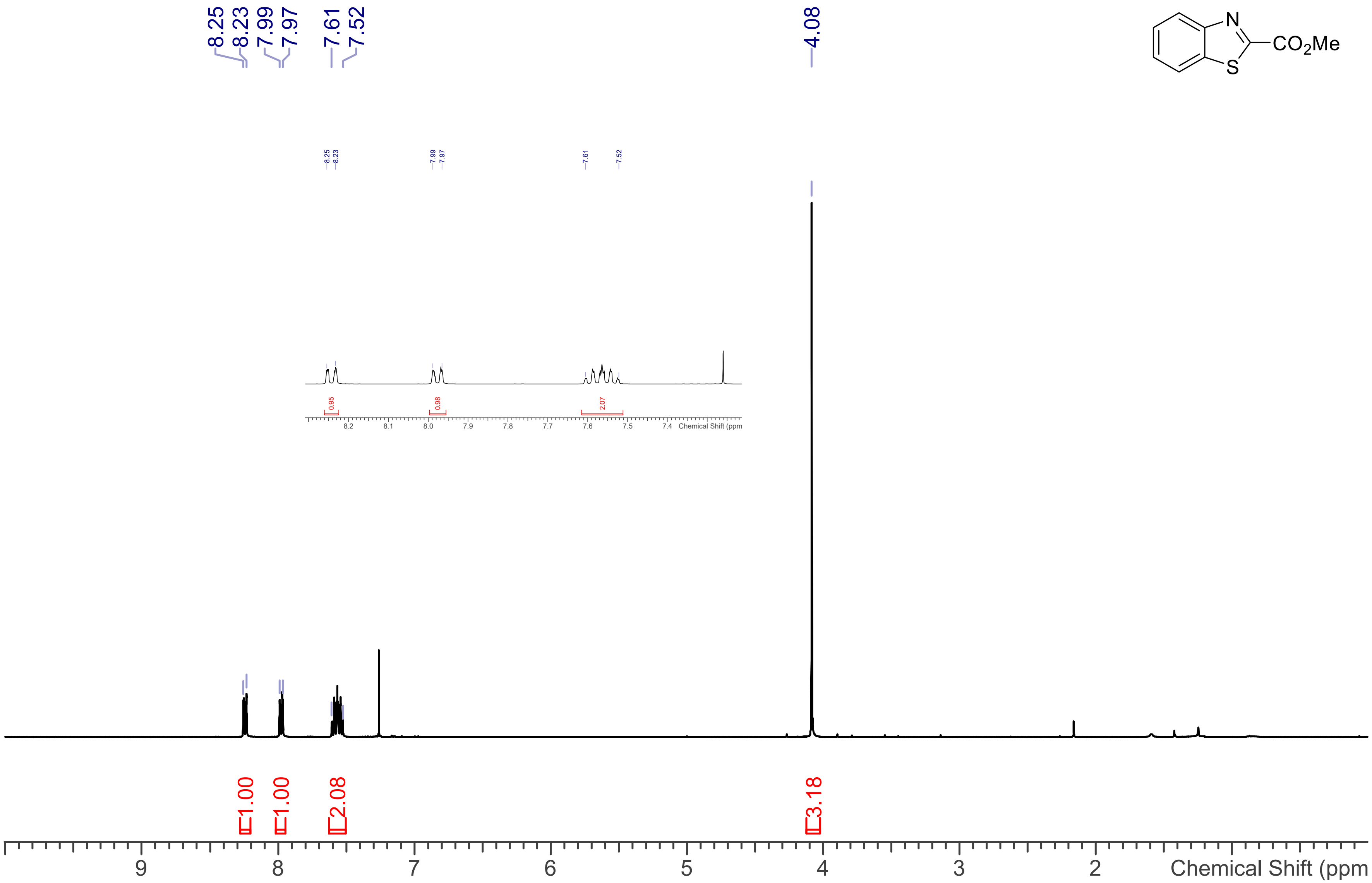

^{1}H NMR (400 MHz, $CDCl_3$) Methyl Benzo[d]thiazole-2-carboxylate (**3a**).

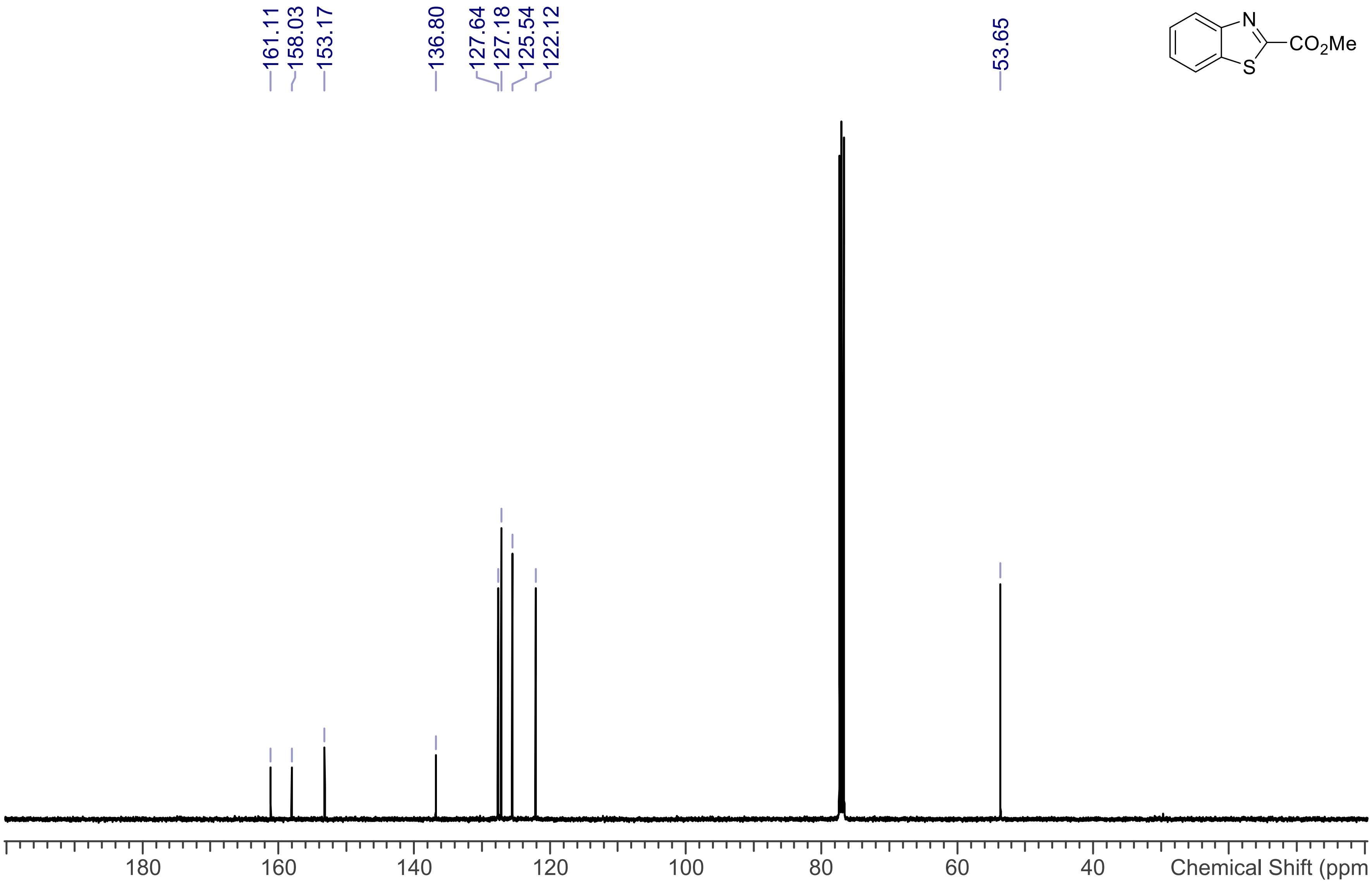

^{13}C NMR (101 MHz, $CDCl_3$) Methyl Benzo[d]thiazole-2-carboxylate (**3a**).

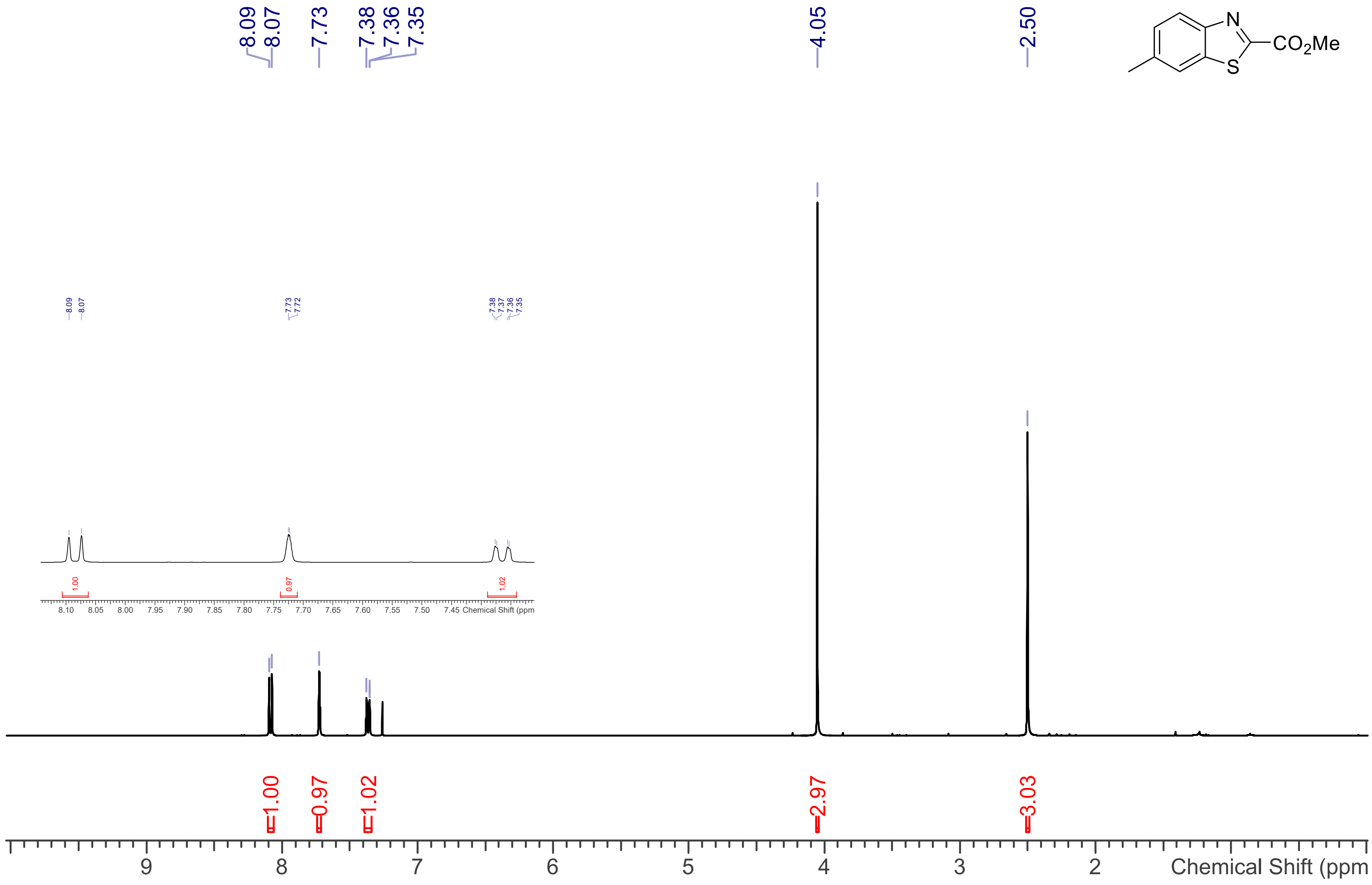

^{1}H NMR (400 MHz, CDCl3) Methyl 6-Methylbenzo[d]thiazole-2-carboxylate (**3b**).

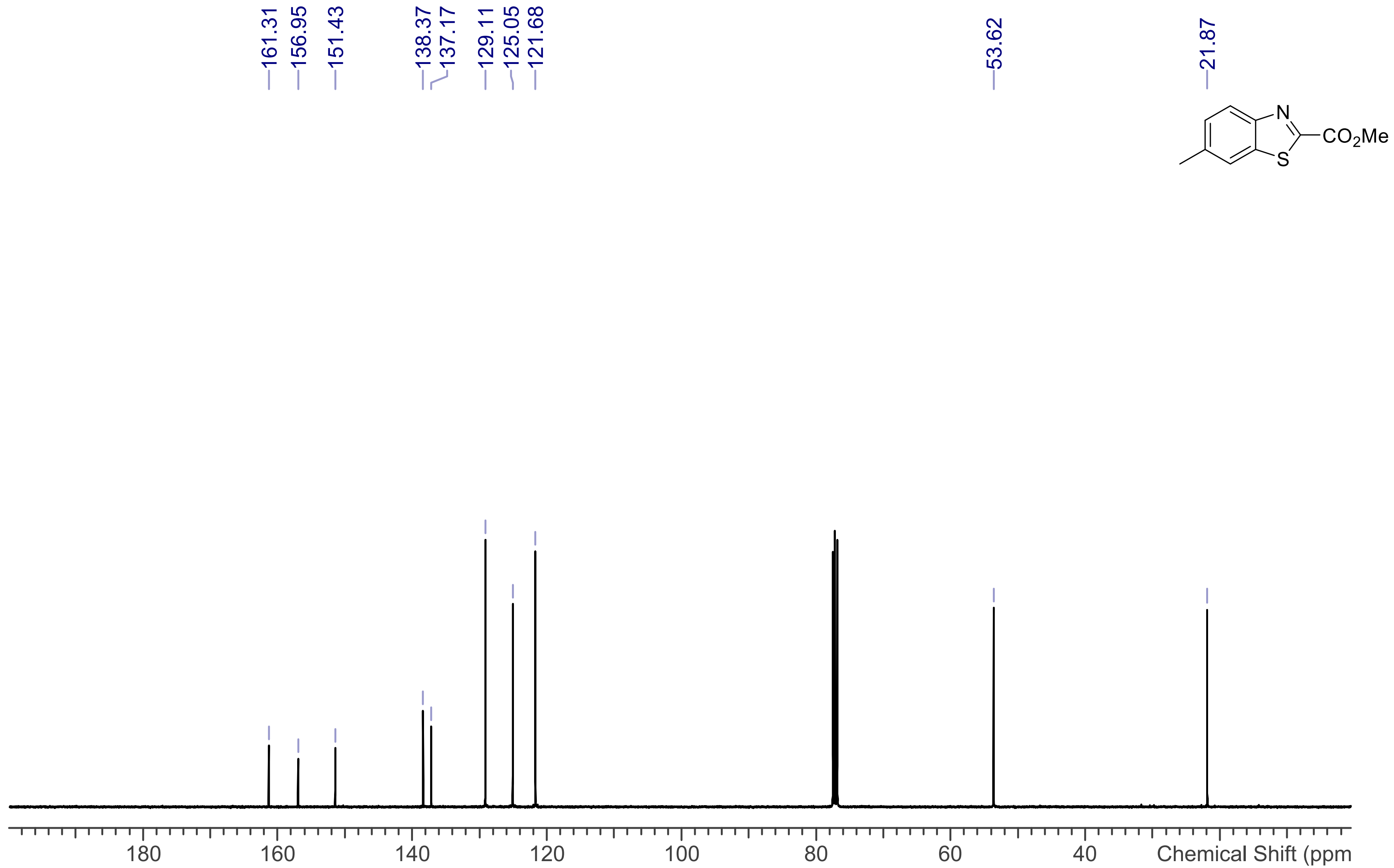

^{13}C NMR (101 MHz, $CDCl_3$) Methyl 6-Methylbenzo[d]thiazole-2-carboxylate (**3b**).

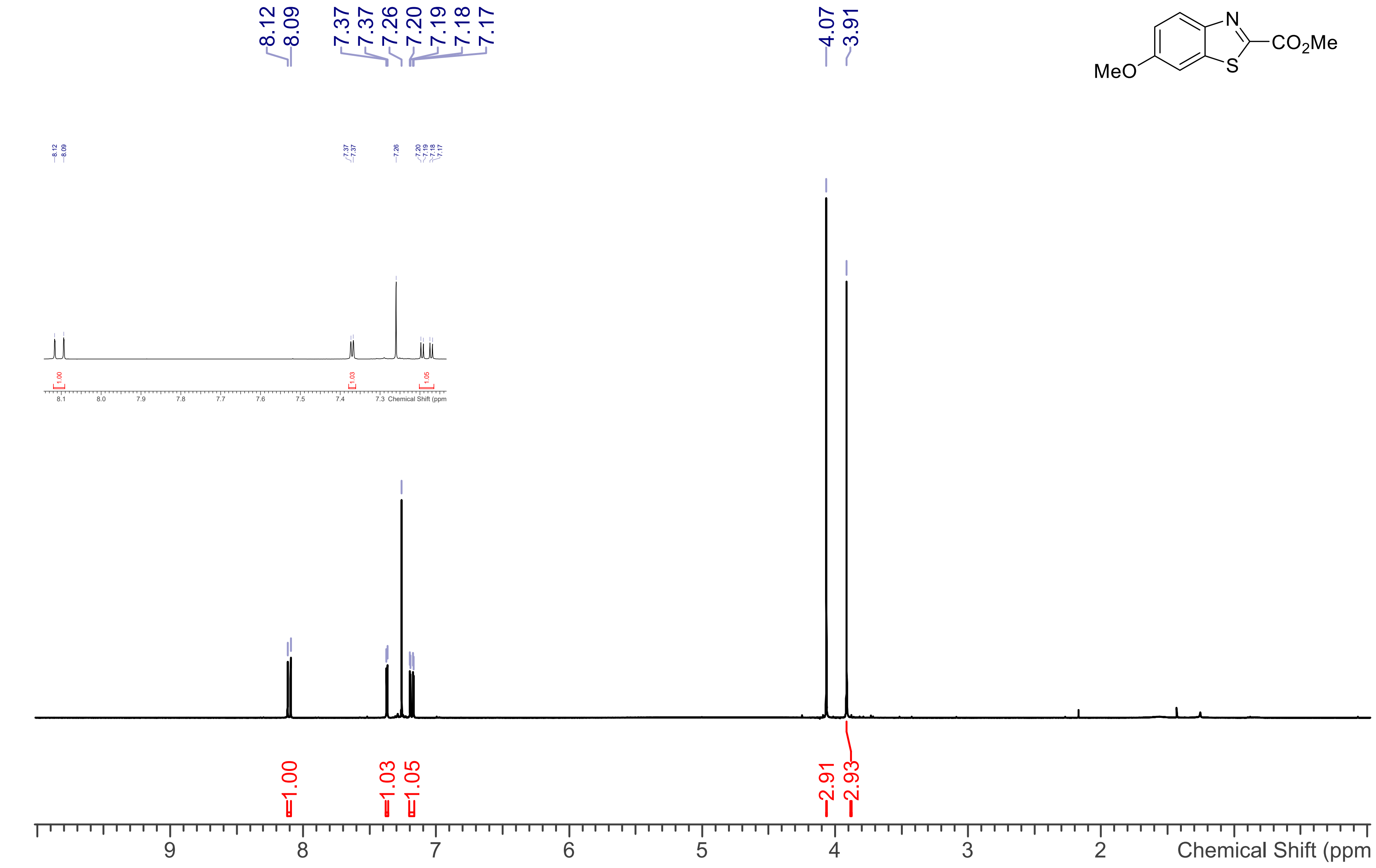

^{1}H NMR (400 MHz, $CDCl_3$) Methyl 6-Methoxybenzo[*d*]thiazole-2-carboxylate (**3c**).

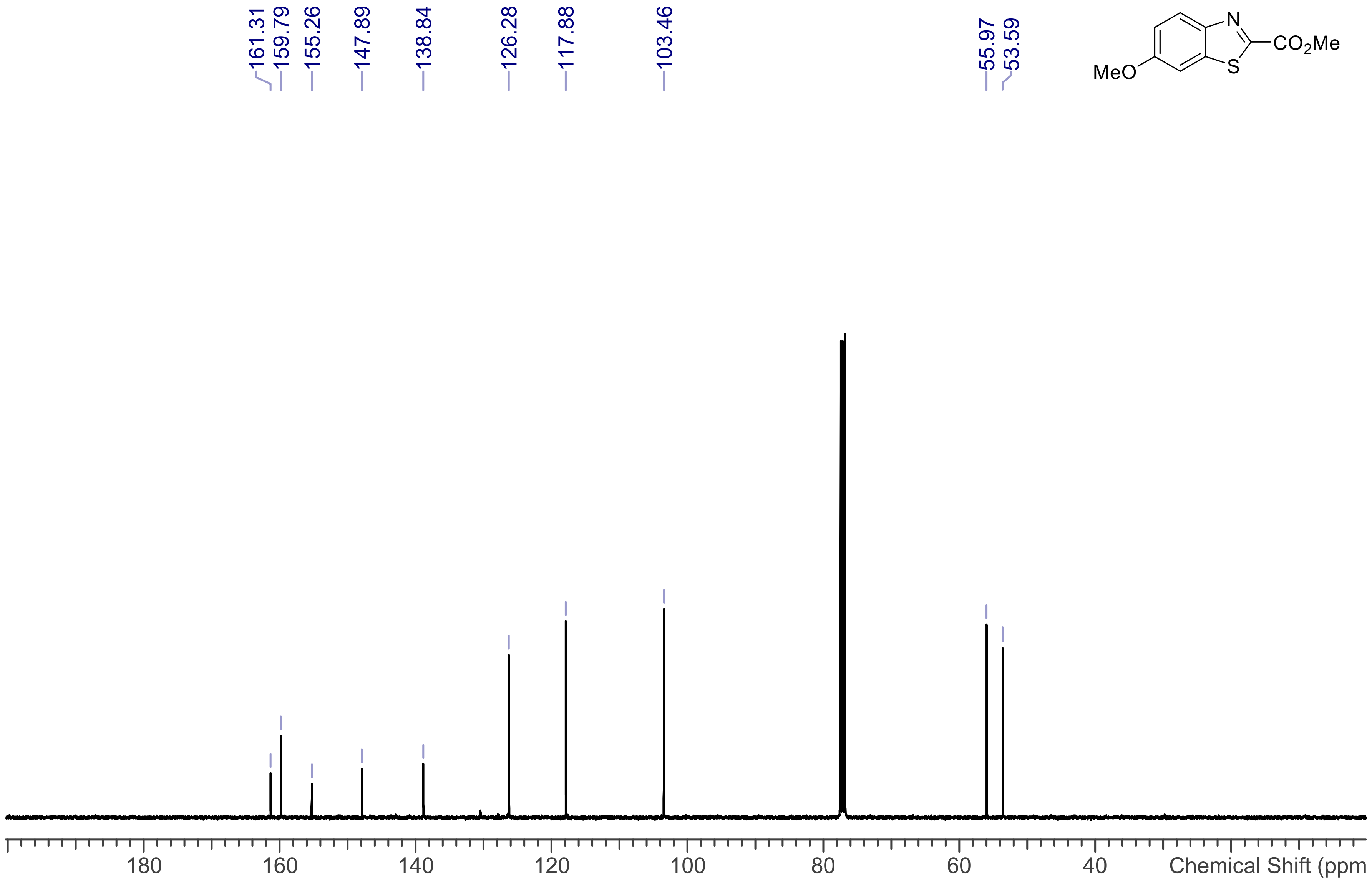

^{13}C NMR (101 MHz, CDCl3) Methyl 6-Methoxybenzo[*d*]thiazole-2-carboxylate (**3c**).

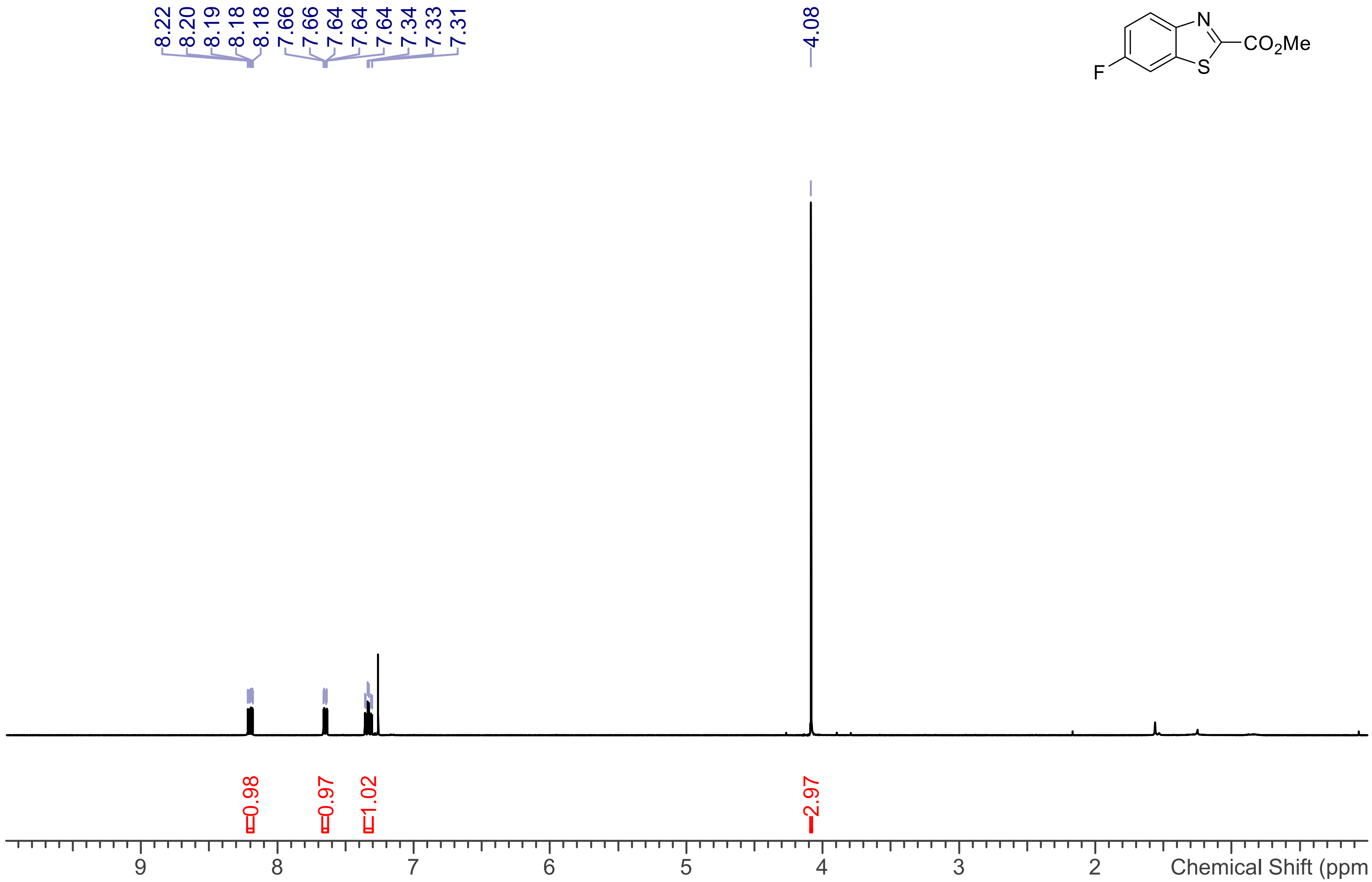

^{1}H NMR (400 MHz, $CDCl_3$) Methyl 6-Fluorobenzo[*d*]thiazole-2-carboxylate (**3d**).

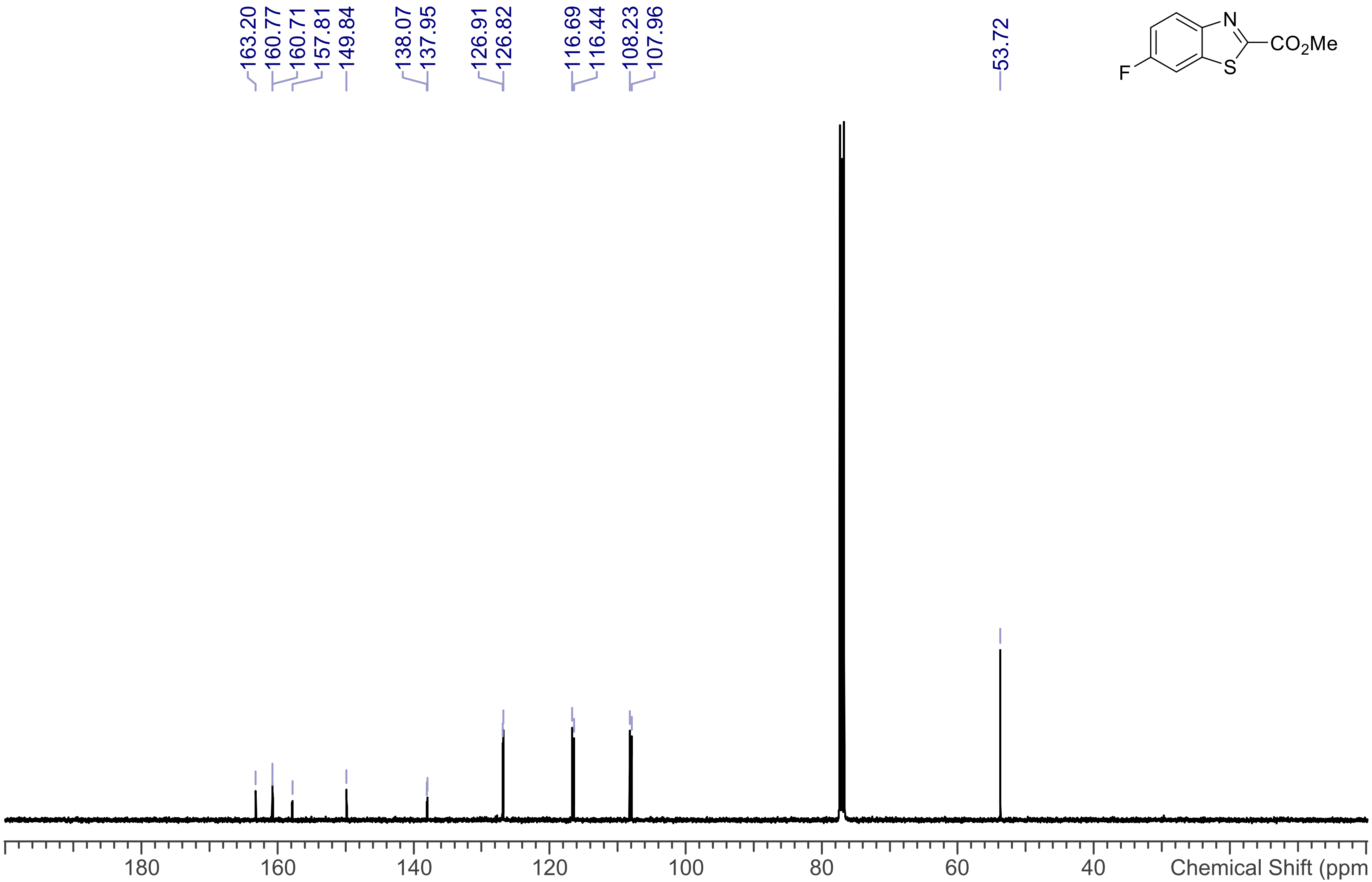

^{13}C NMR (101 MHz, $CDCl_3$) Methyl 6-Fluorobenzo[d]thiazole-2-carboxylate (**3d**).

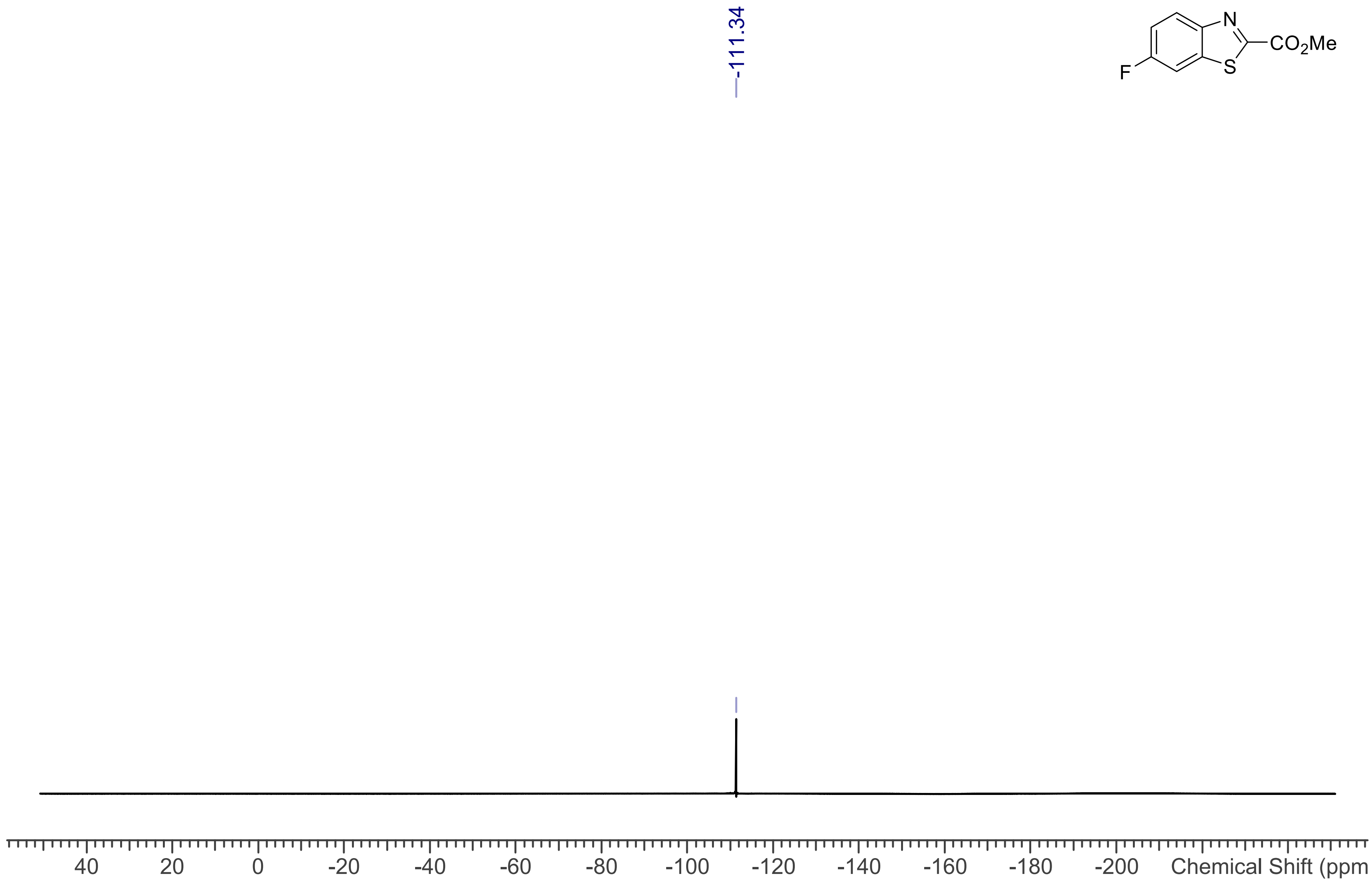

$^{19}F\{^1H\}$ NMR (376 MHz, $CDCl_3$) Methyl 6-Fluorobenzo[d]thiazole-2-carboxylate (**3d**).

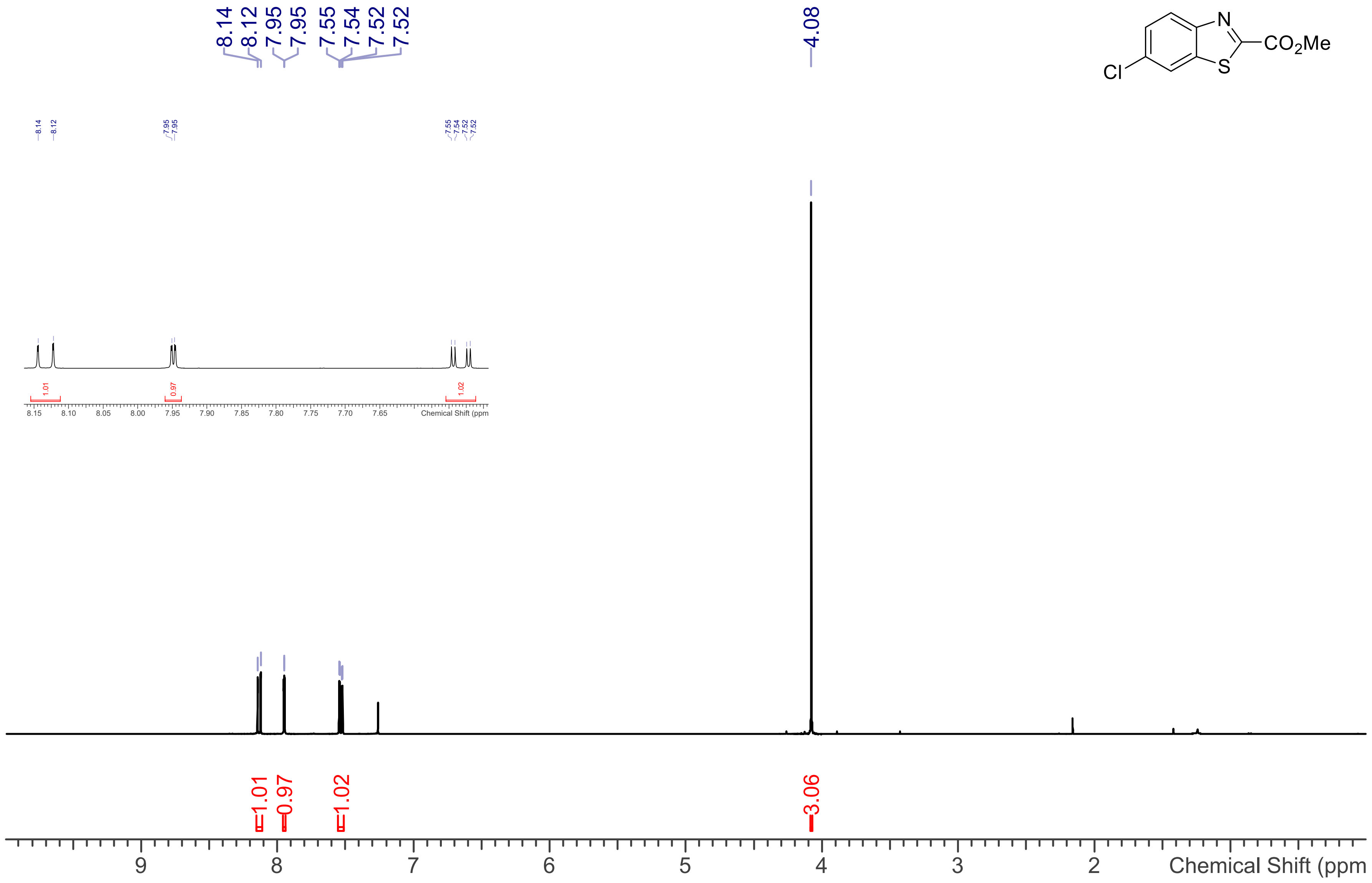

^{1}H NMR (400 MHz, $CDCl_3$) Methyl 6-Chlorobenzo[d]thiazole-2-carboxylate (**3e**).

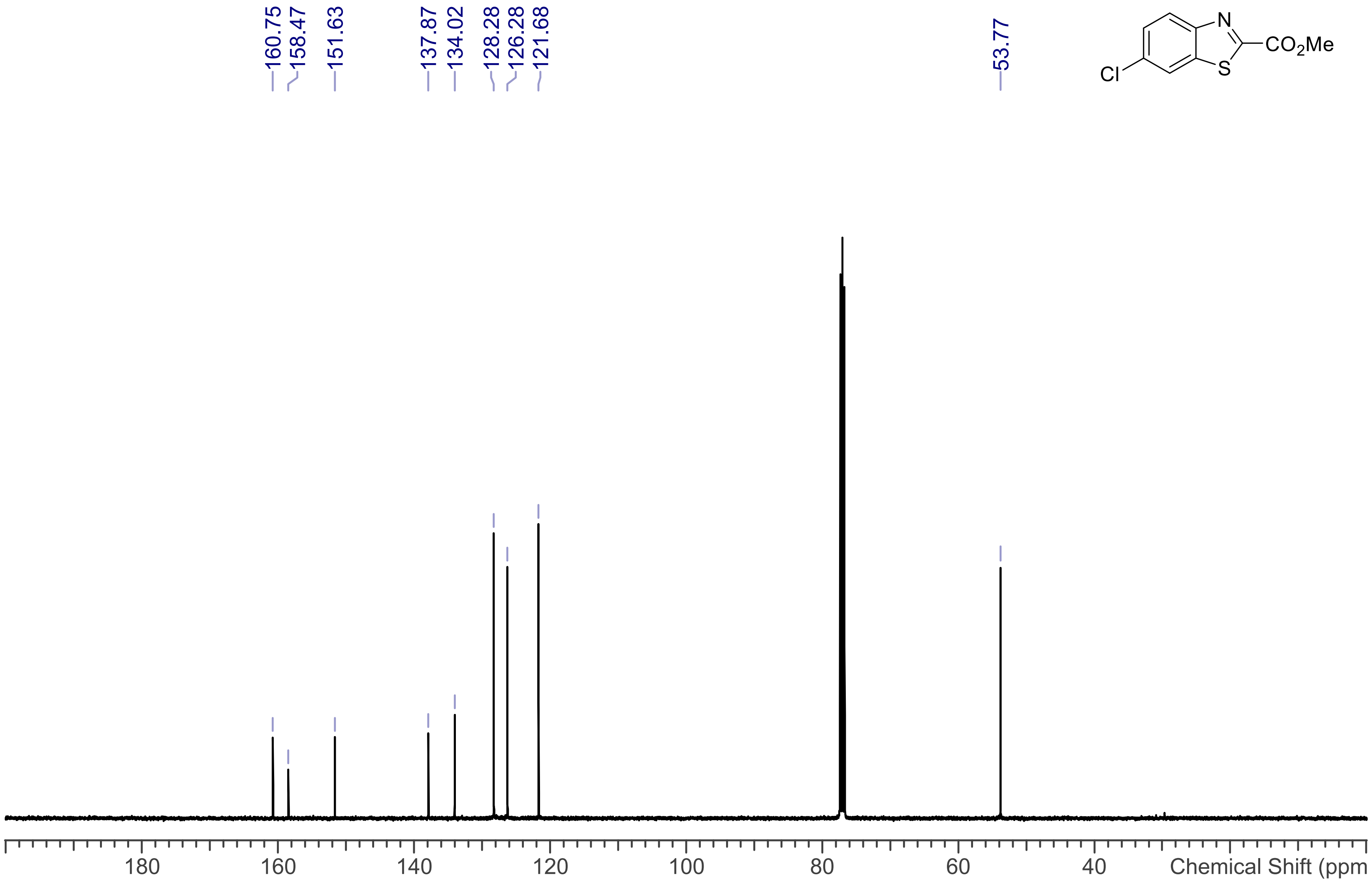

^{13}C NMR (101 MHz, $CDCl_3$) Methyl 6-Chlorobenzo[d]thiazole-2-carboxylate (**3e**).

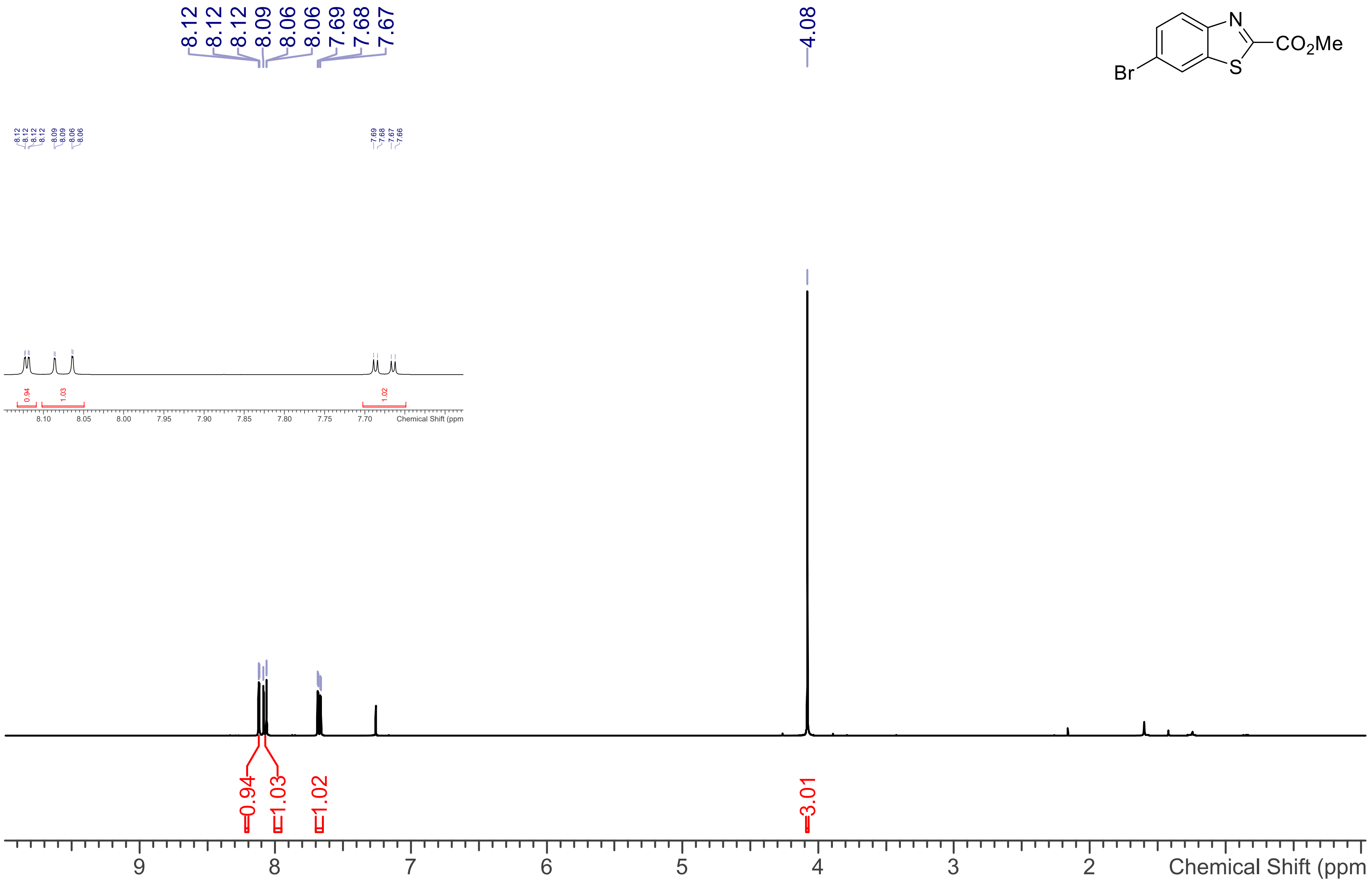

^1H NMR (400 MHz, $CDCl_3$) Methyl 6-Bromobenzo[*d*]thiazole-2-carboxylate (**3f**).

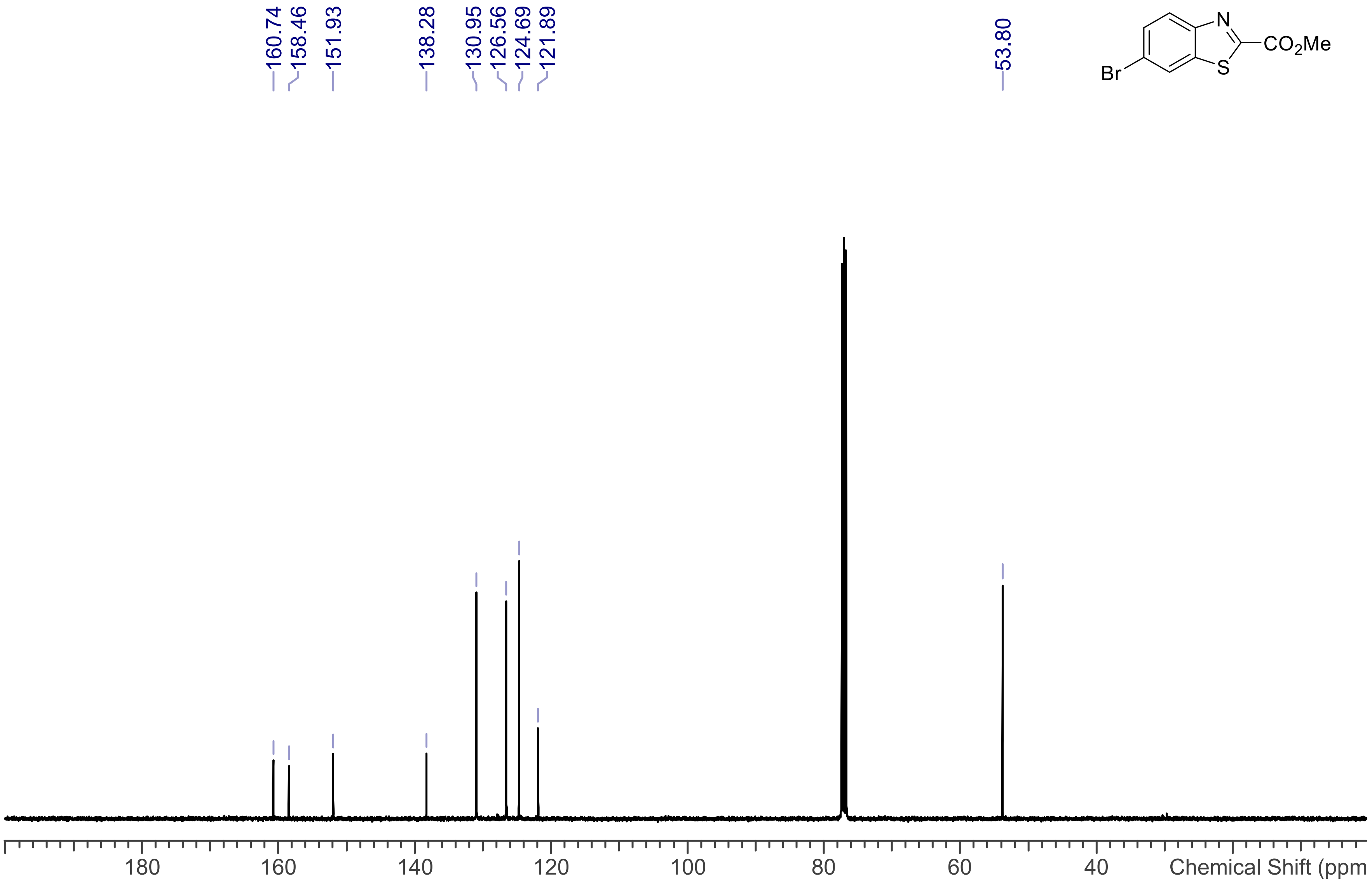

^{13}C NMR (101 MHz, $CDCl_3$) 6-Bromobenzo[*d*]thiazole-2-carboxylate (**3f**).

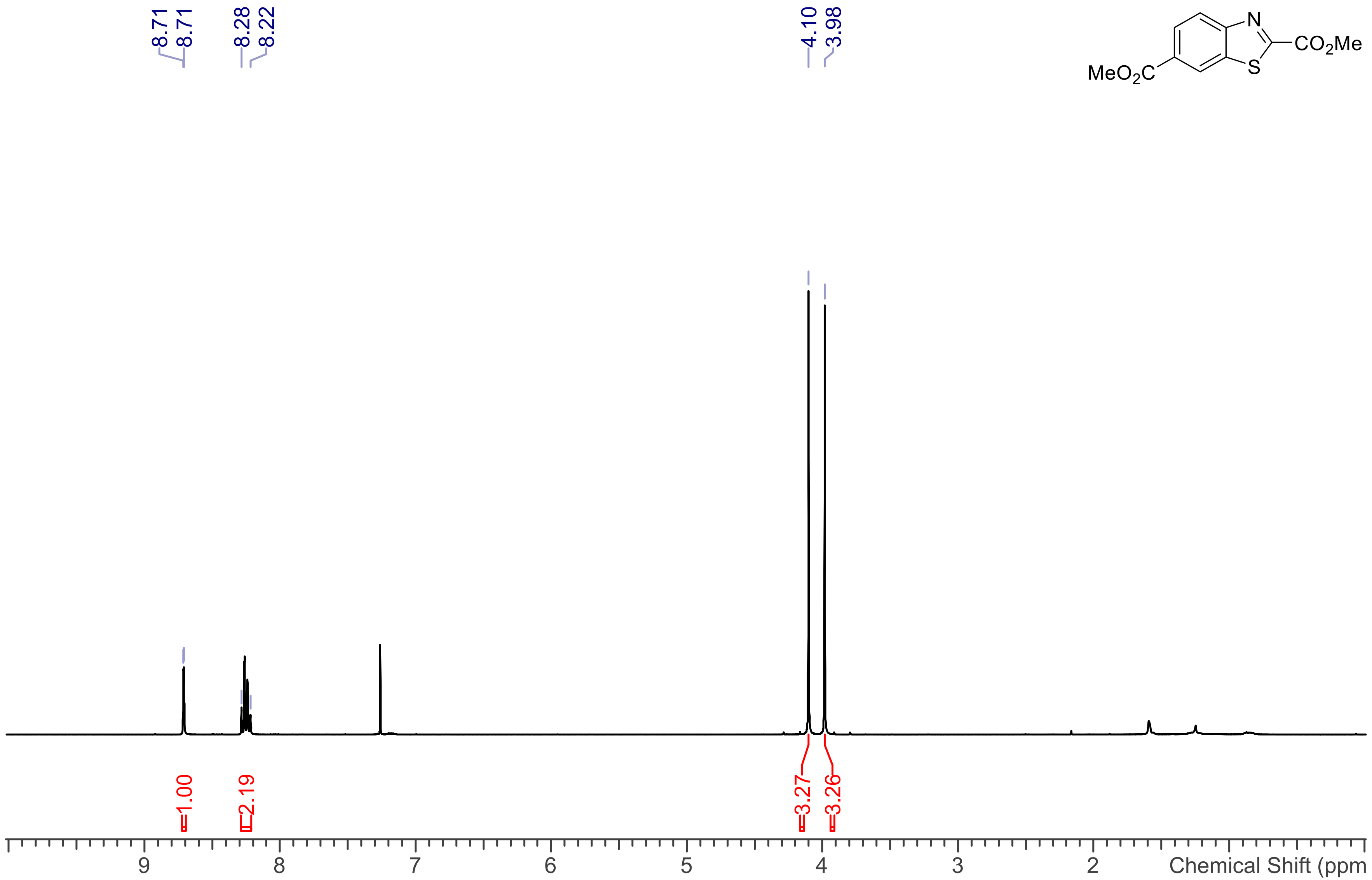

^{1}H NMR (400 MHz, $CDCl_3$) Dimethyl benzothiazole-2,6-dicarboxylate (**3g**).

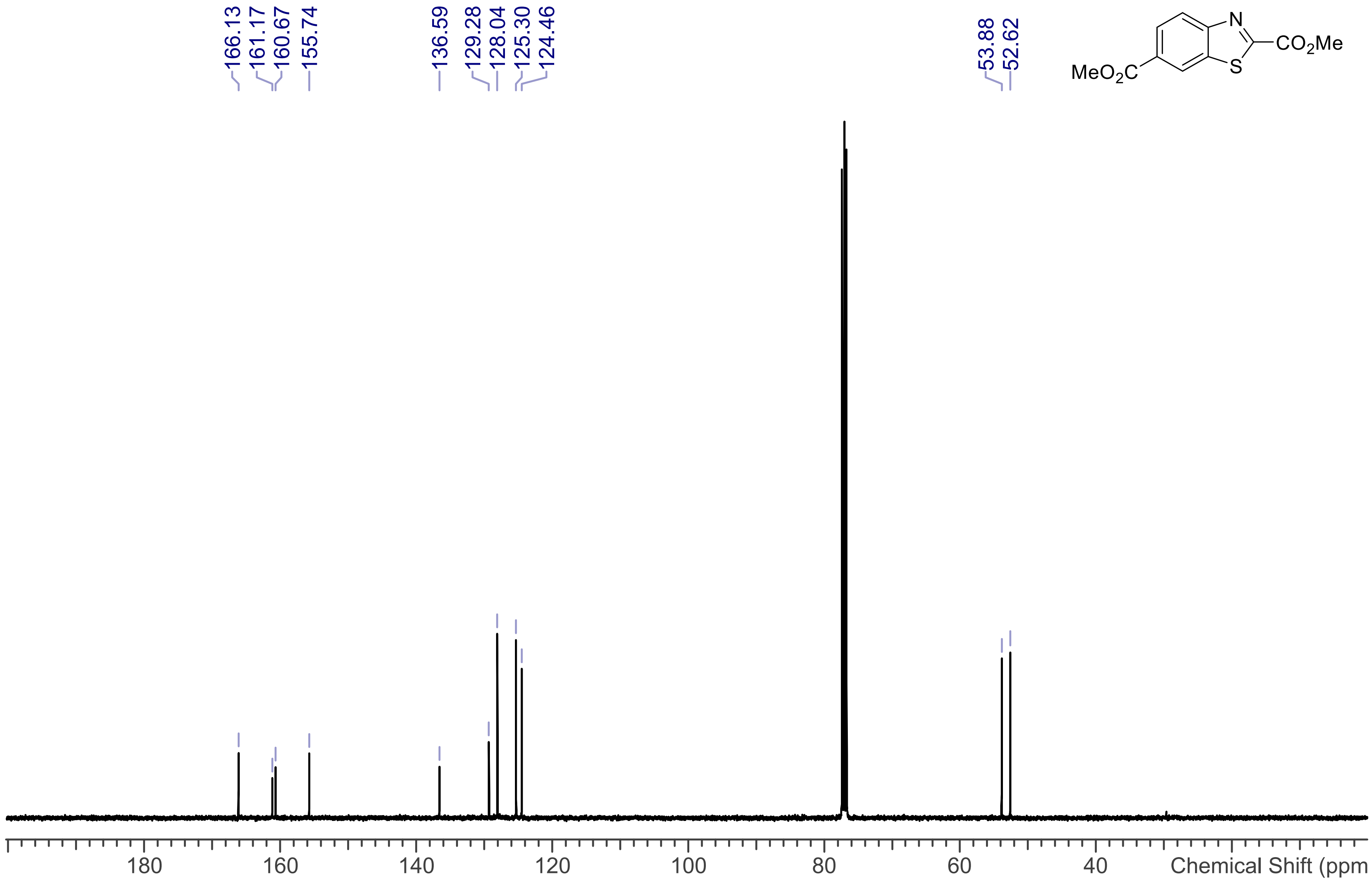

^{13}C NMR (101 MHz, $CDCl_3$) Dimethyl benzothiazole-2,6-dicarboxylate (**3g**).

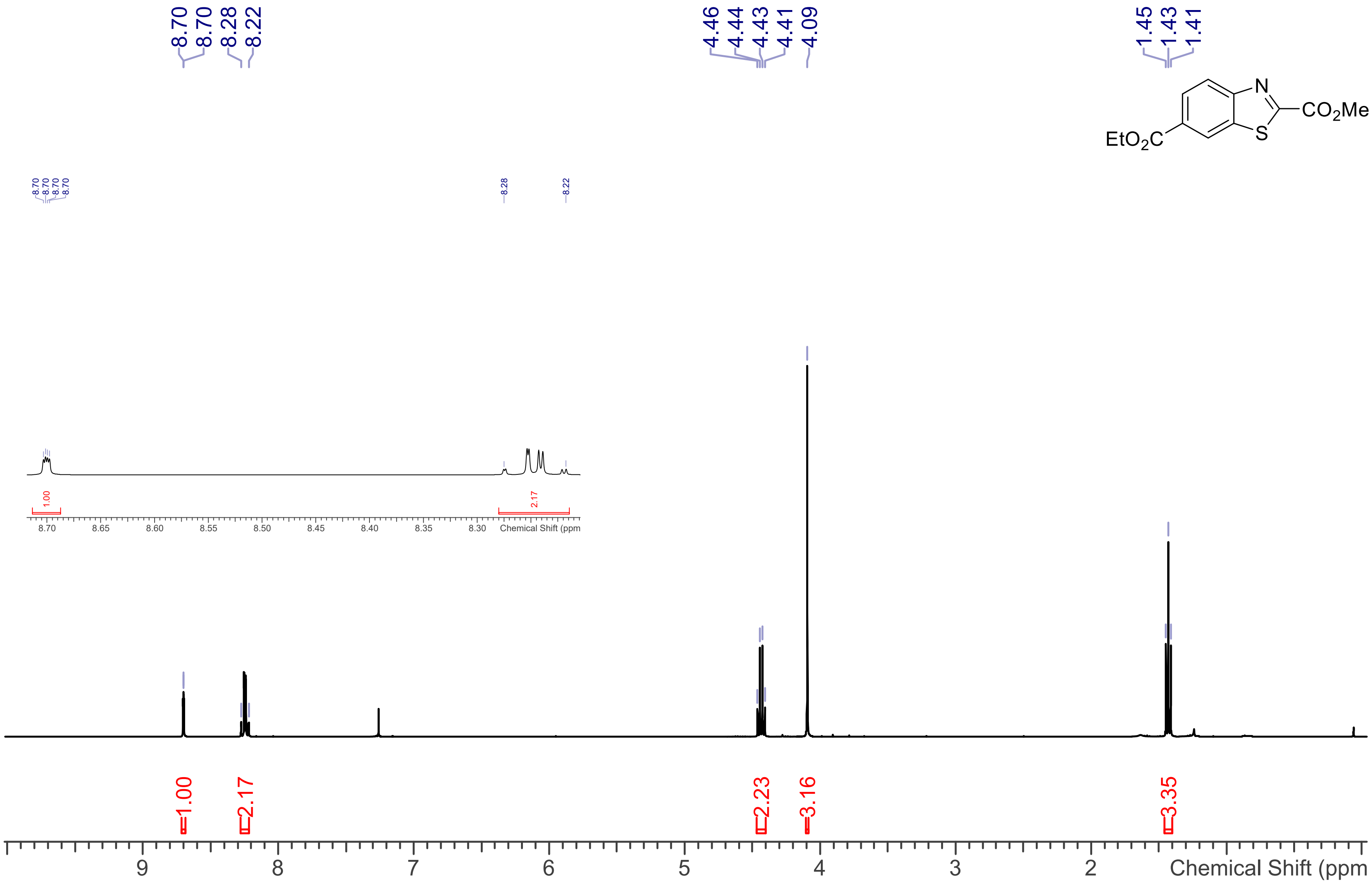

^{1}H NMR (400 MHz, $CDCl_3$) Ethyl Methylbenzo[*d*]thiazole-2,6-dicarboxylate (**3h**).

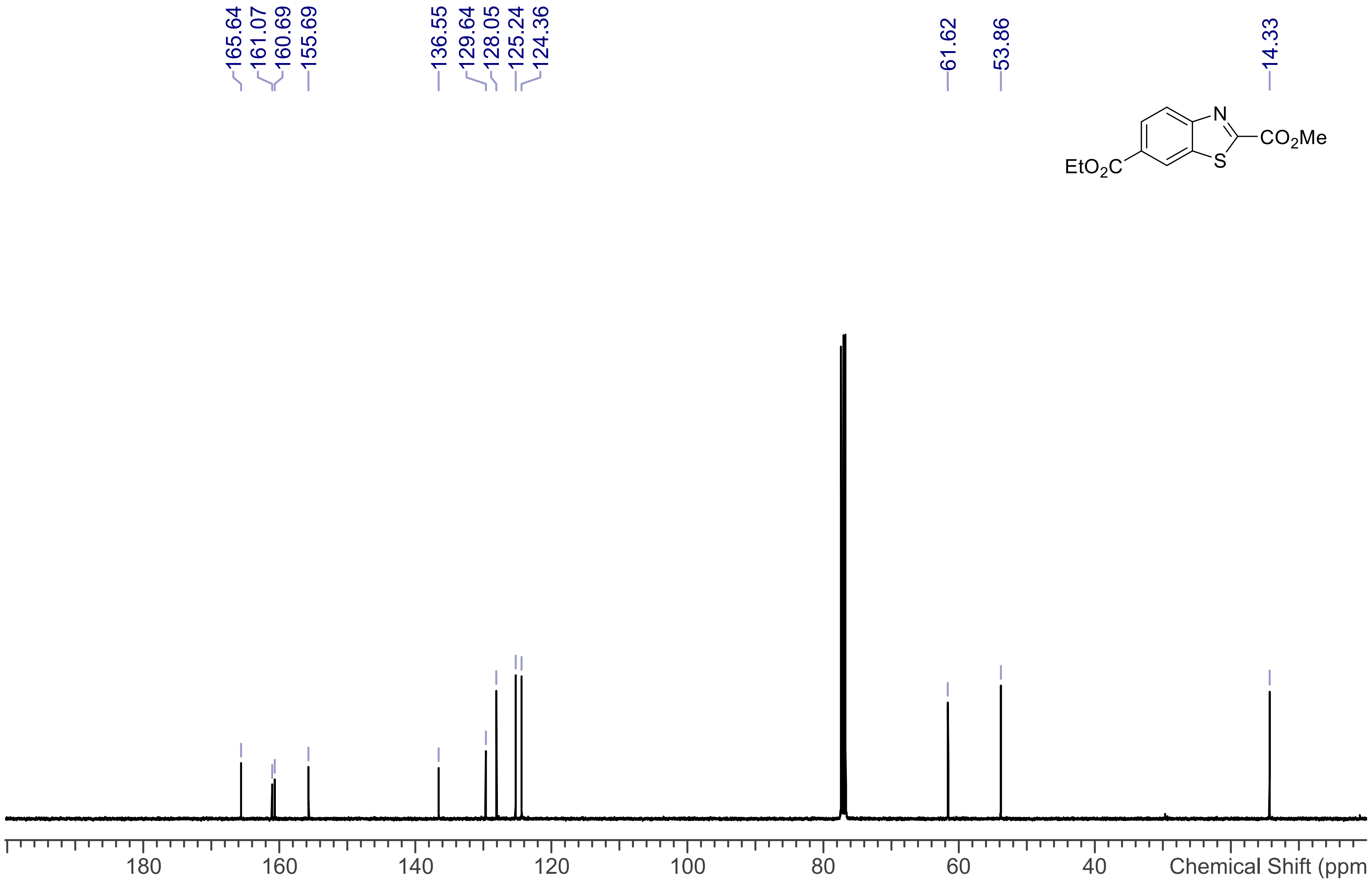

^{13}C NMR (101 MHz, $CDCl_3$) Ethyl Methylbenzo[*d*]thiazole-2,6-dicarboxylate (**3h**).

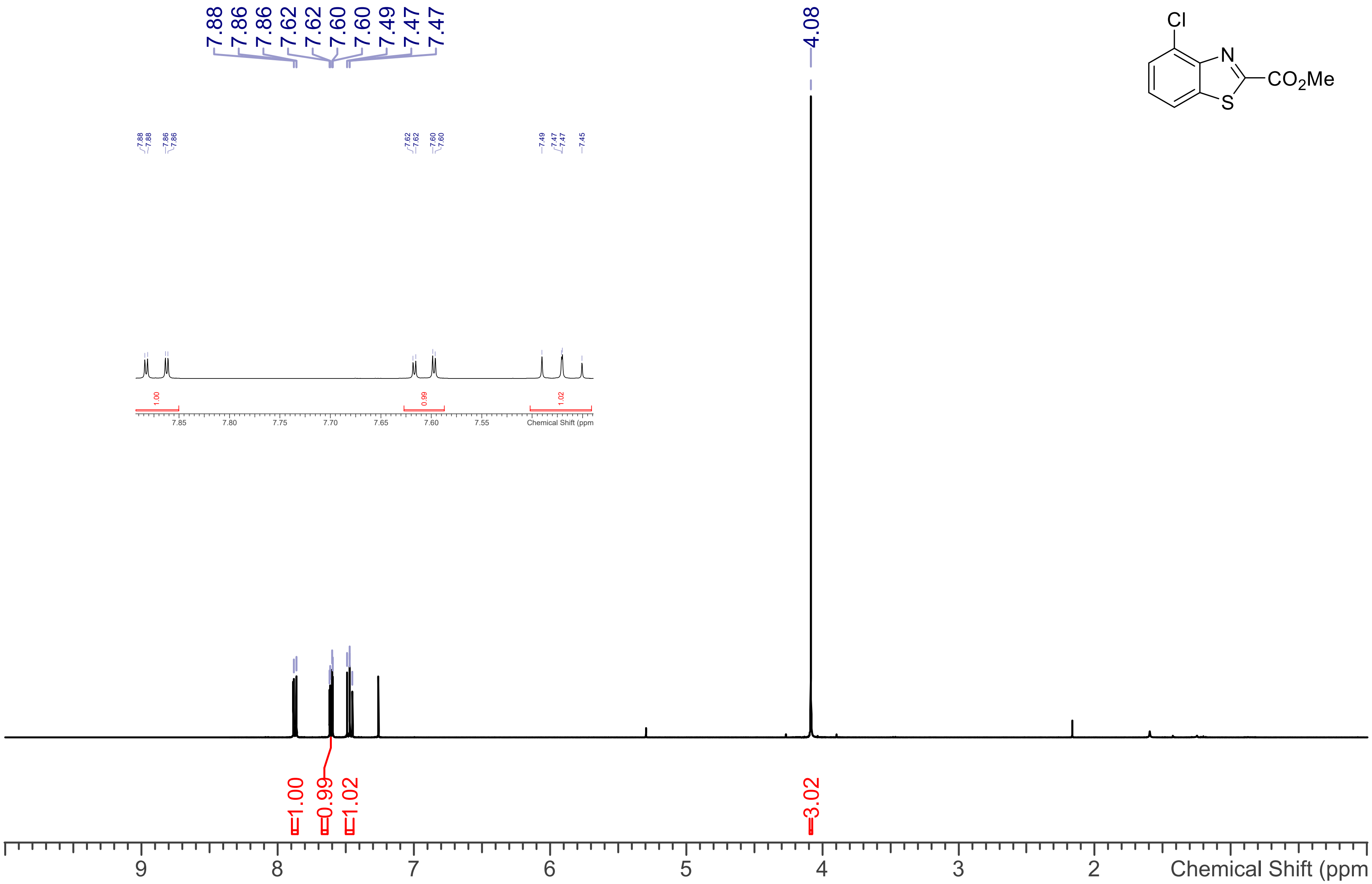

^{1}H NMR (400 MHz, $CDCl_3$) Methyl 4-Chlorobenzo[*d*]thiazole-2-carboxylate (**3i**).

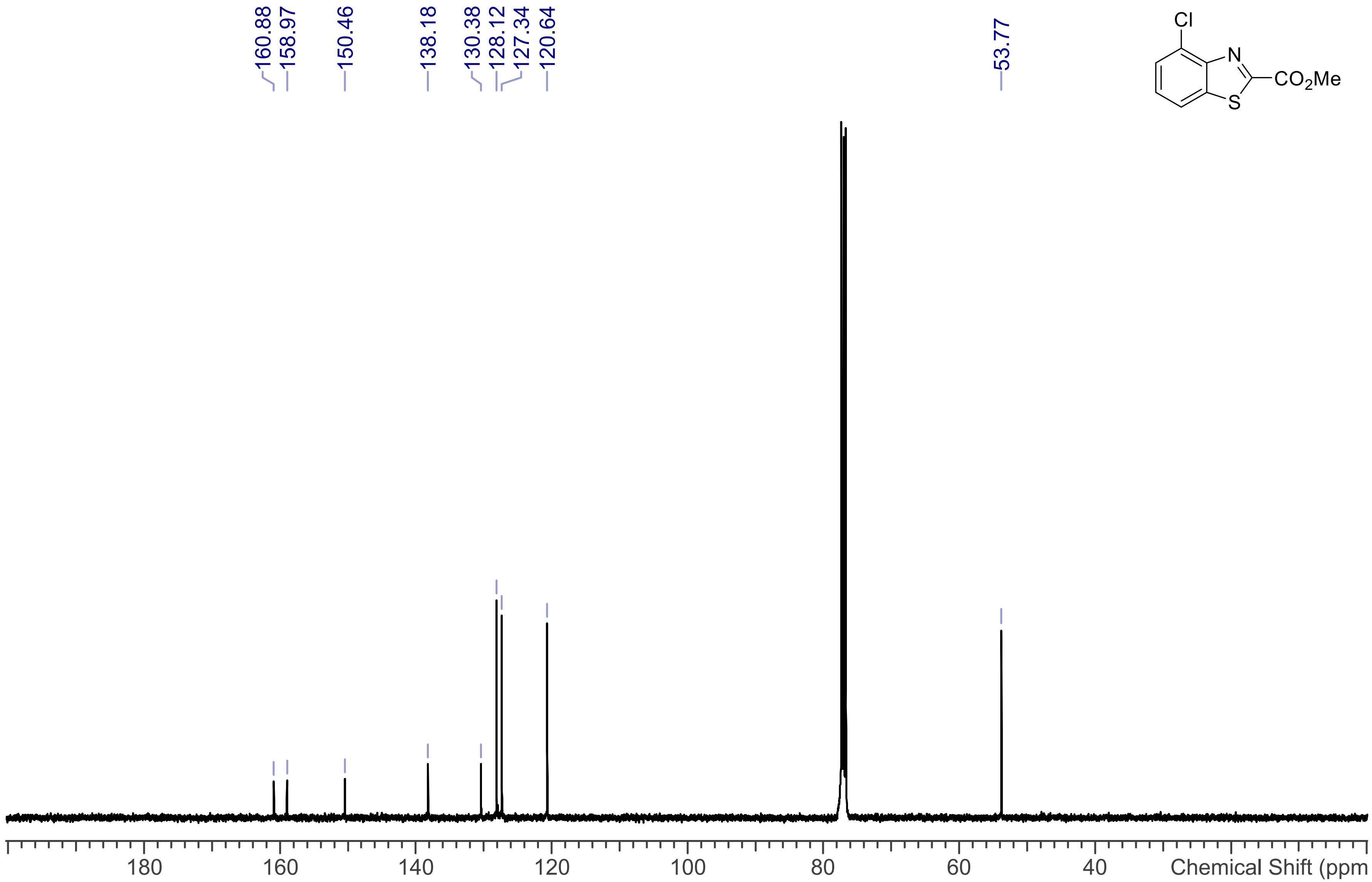

^{13}C NMR (400 MHz, $CDCl_3$) Methyl 4-Chlorobenzo[*d*]thiazole-2-carboxylate (**3i**).

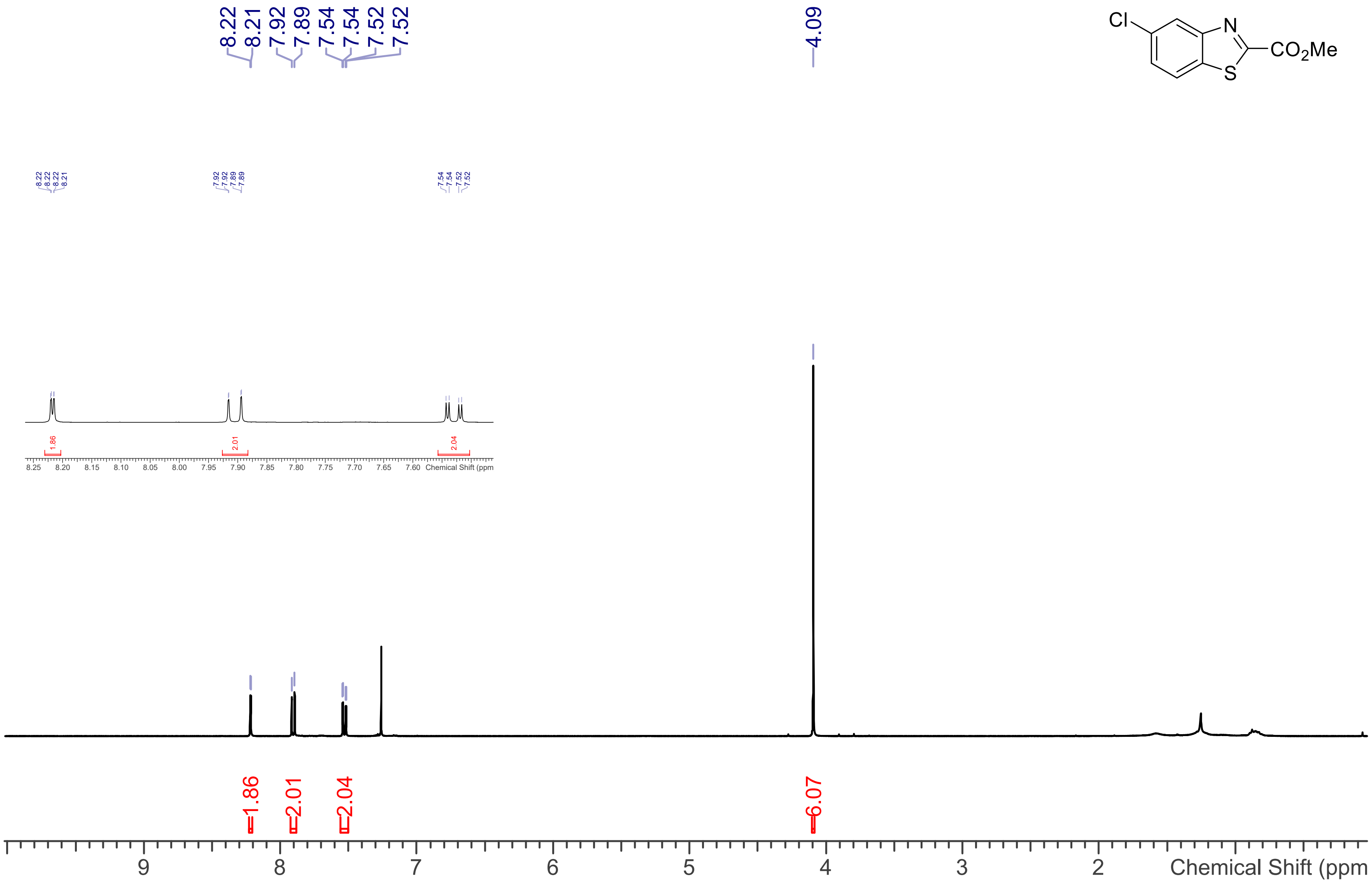

^{1}H NMR (400 MHz, $CDCl_3$) Methyl 5-Chlorobenzo[d]thiazole-2-carboxylate (**3j**).

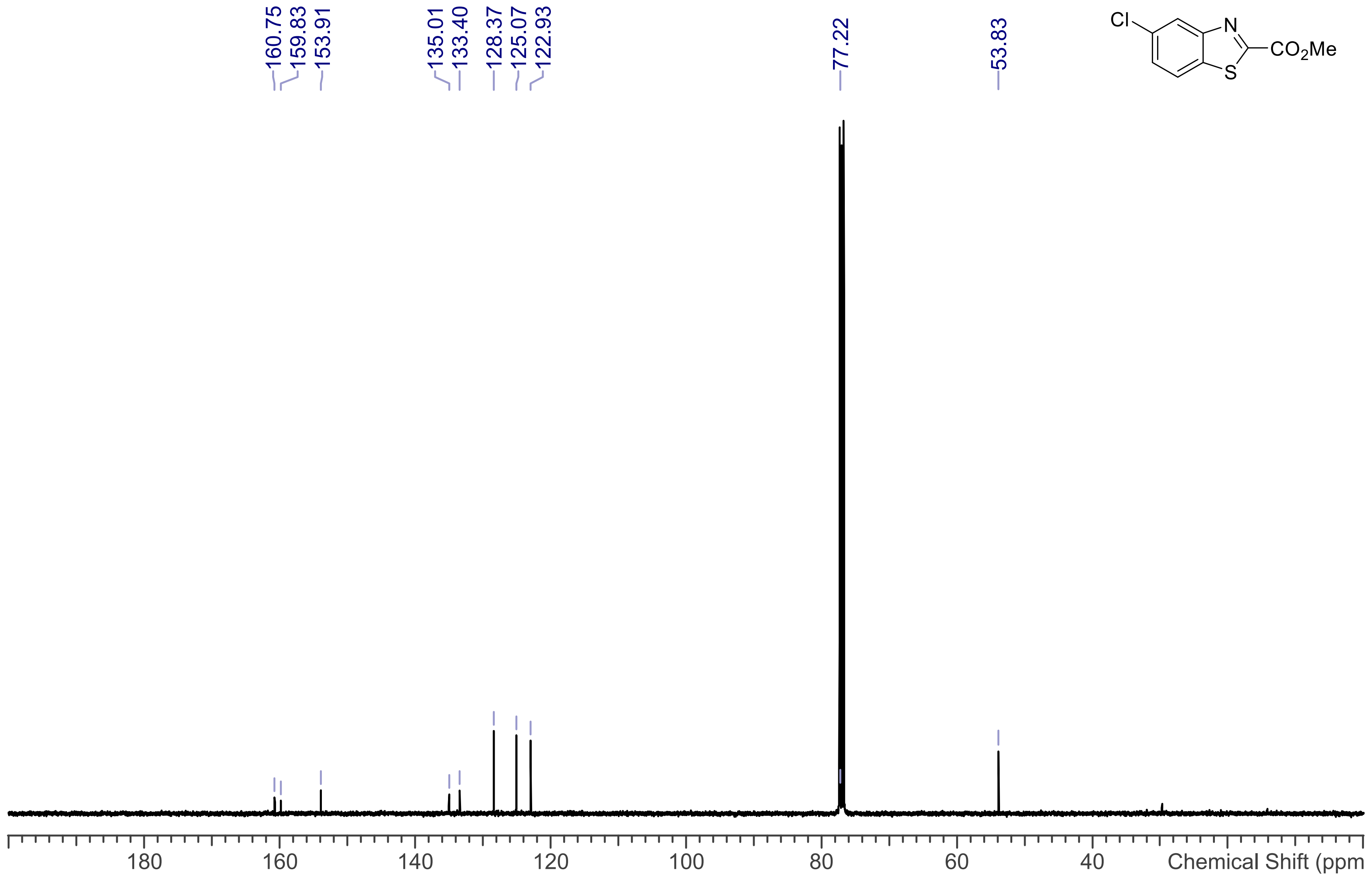

^{13}C NMR (101 MHz, $CDCl_3$) Methyl 5-Chlorobenzo[d]thiazole-2-carboxylate (**3j**).

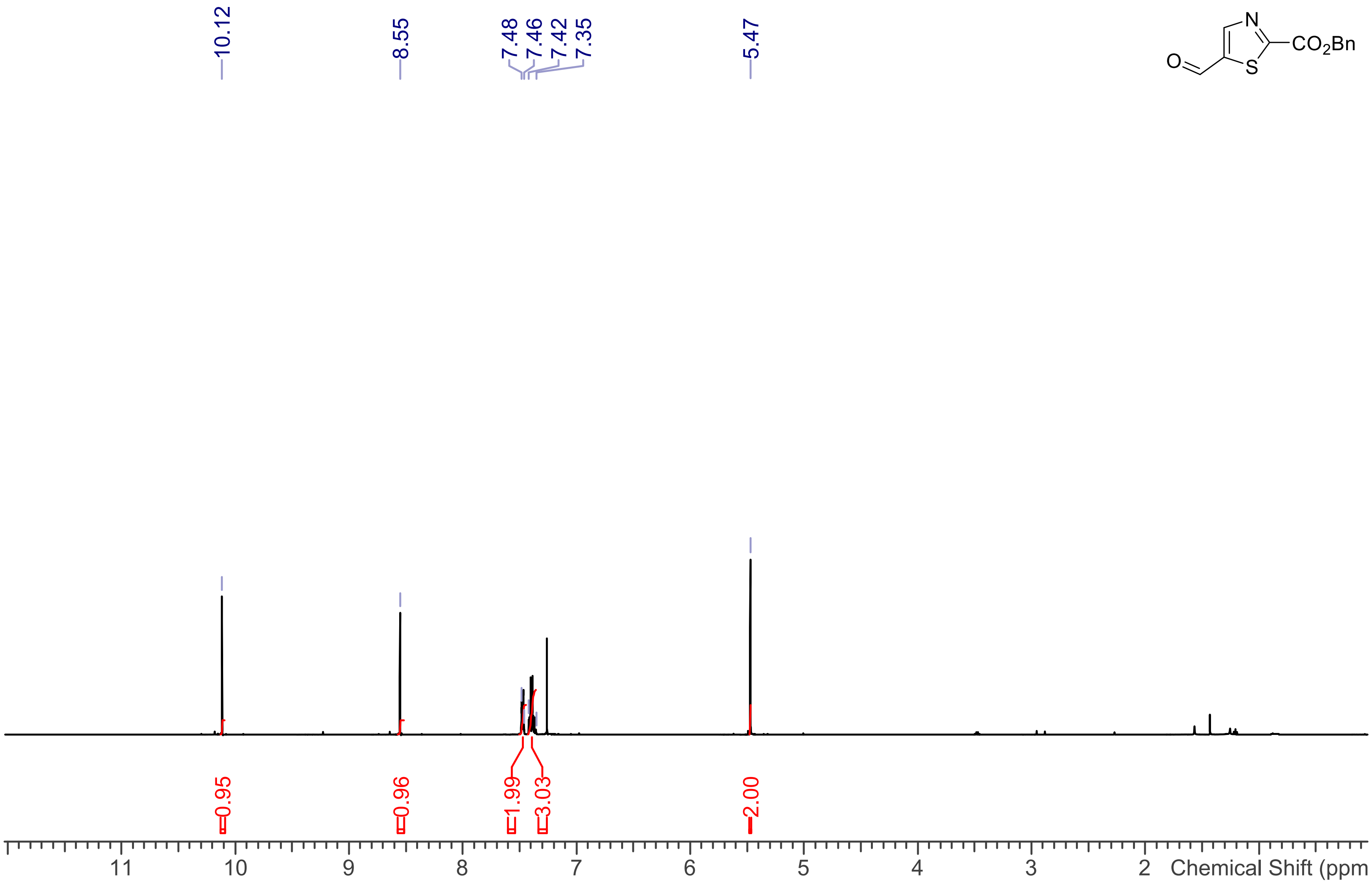

^{1}H NMR (500 MHz, $CDCl_3$) Benzyl 5-formylthiazole-5-carboxylate (**3k**).

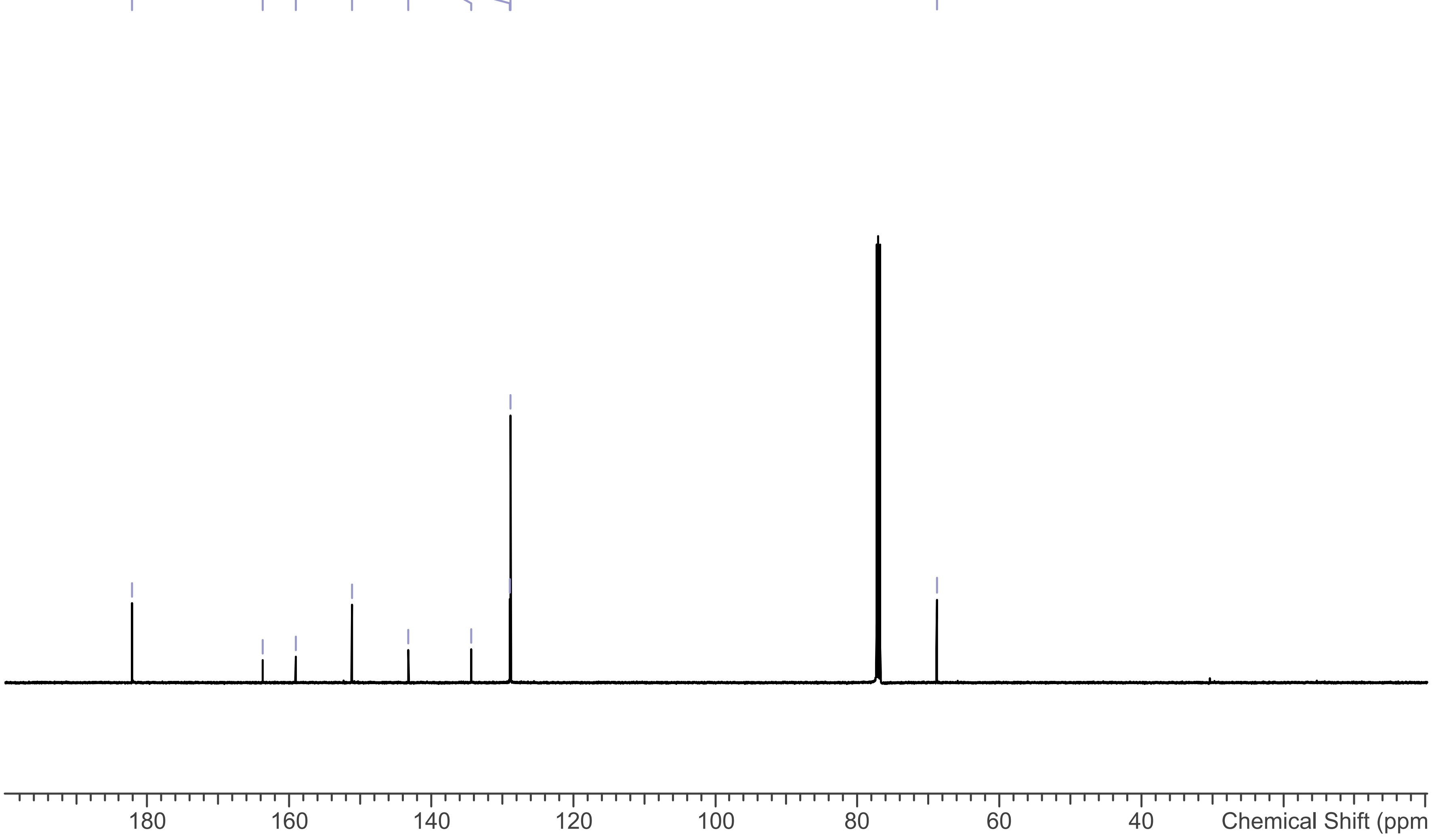

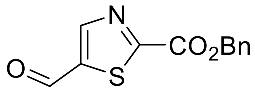

^{13}C NMR (125 MHz, $CDCl_3$) Benzyl 5-formylthiazole-5-carboxylate (**3k**).

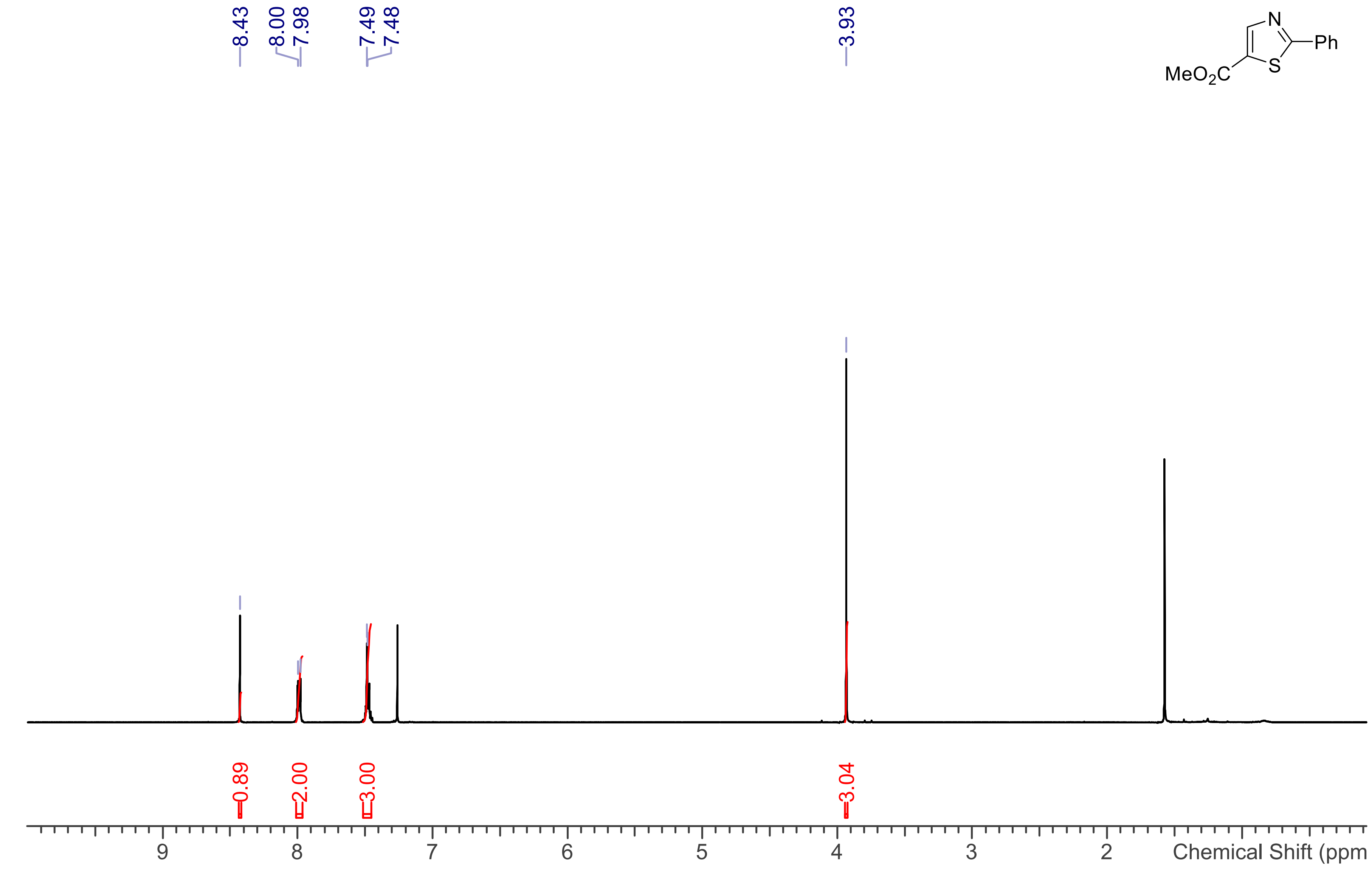

^{1}H NMR (400 MHz, $CDCl_3$) Methyl 2-phenylthiazole-5-carboxylate (**3l**).

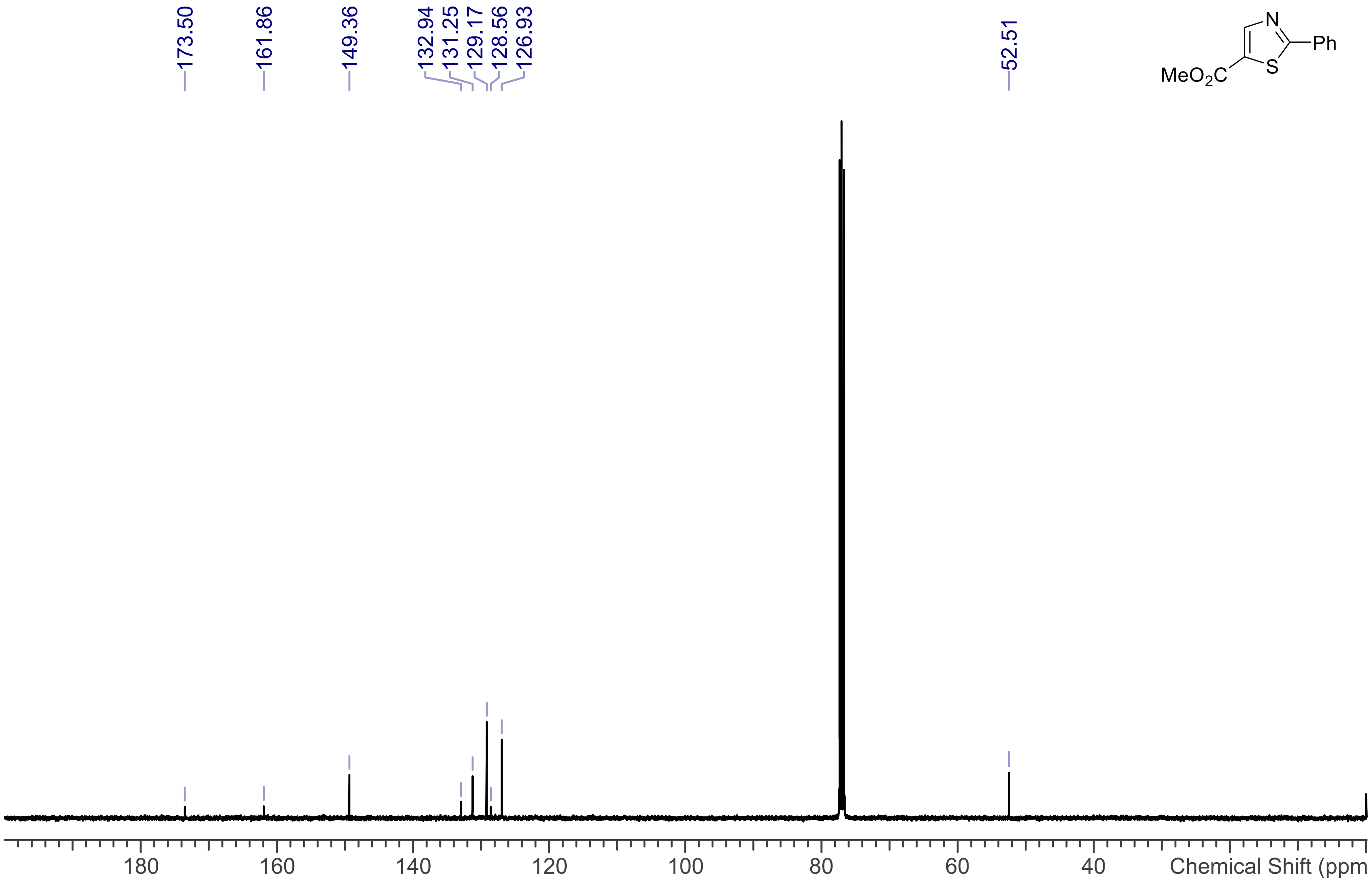

^{13}C NMR (400 MHz, $CDCl_3$) Methyl 2-phenylthiazole-5-carboxylate (**3l**).

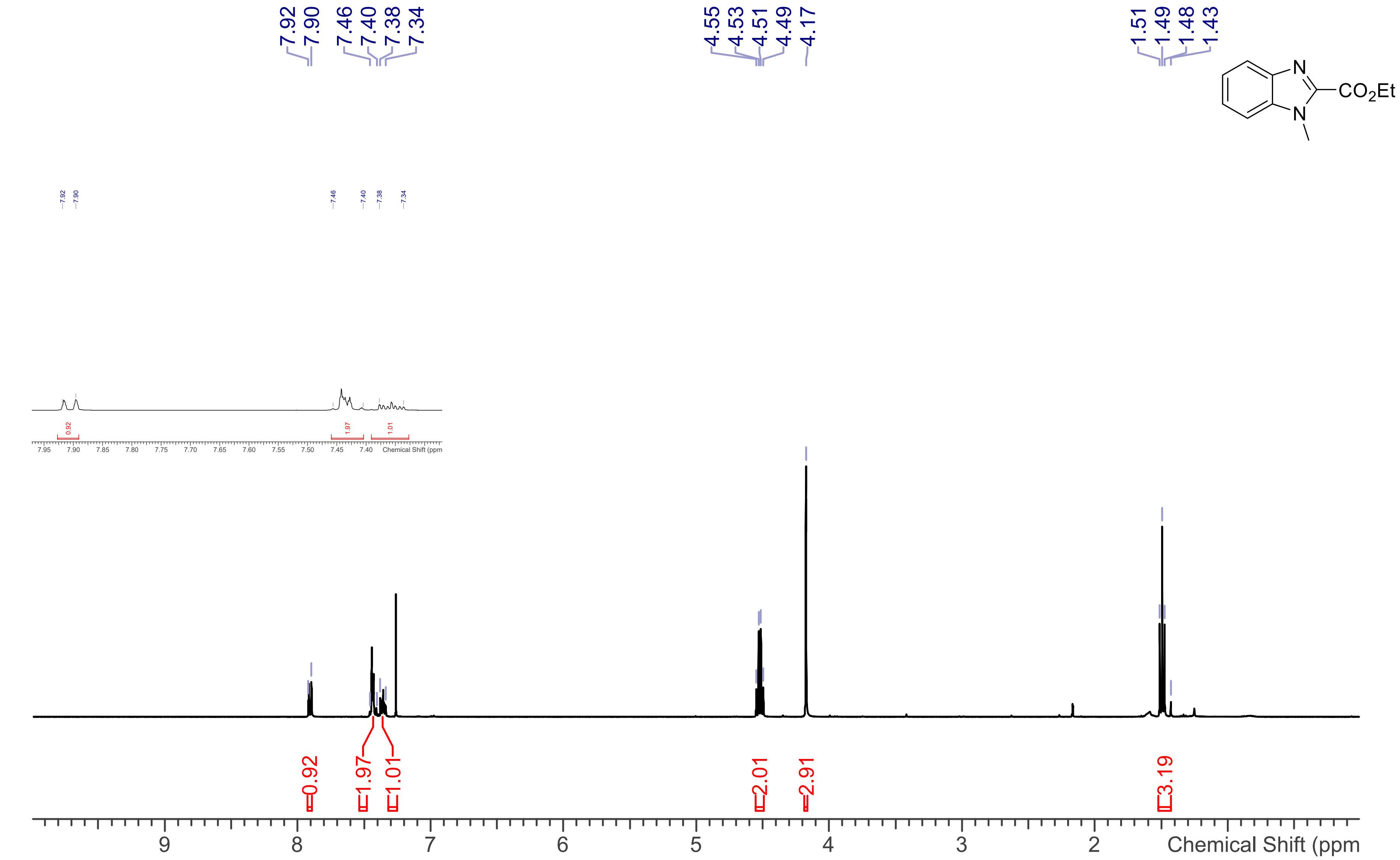

^{1}H NMR (400 MHz, $CDCl_3$) Ethyl 1-Methyl-1*H*-Benzo[*d*]imidazole-2-carboxylate (**3m**).

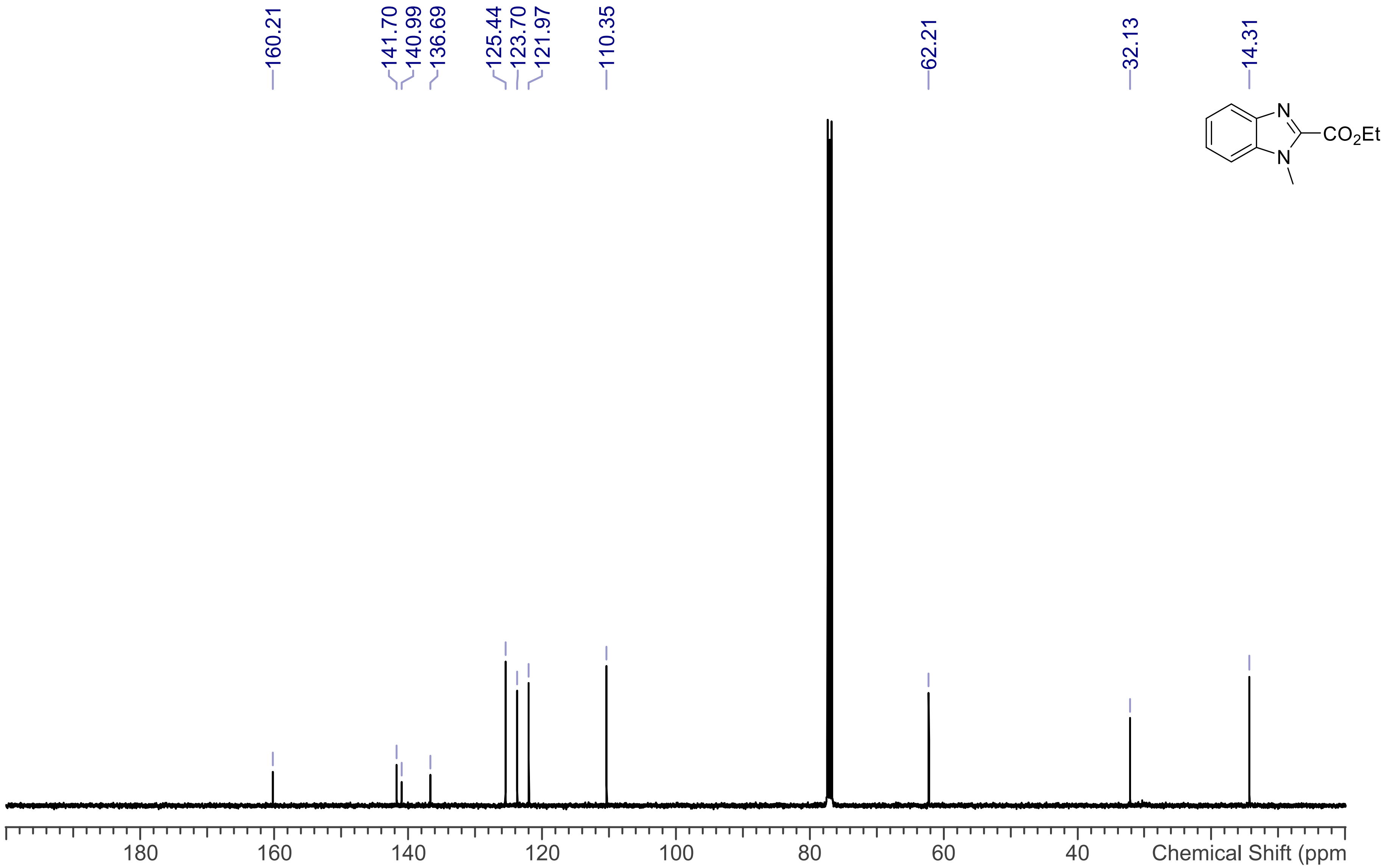

^{13}C NMR (101 MHz, $CDCl_3$) Ethyl 1-Methyl-1*H*-Benzo[*d*]imidazole-2-carboxylate (**3m**).

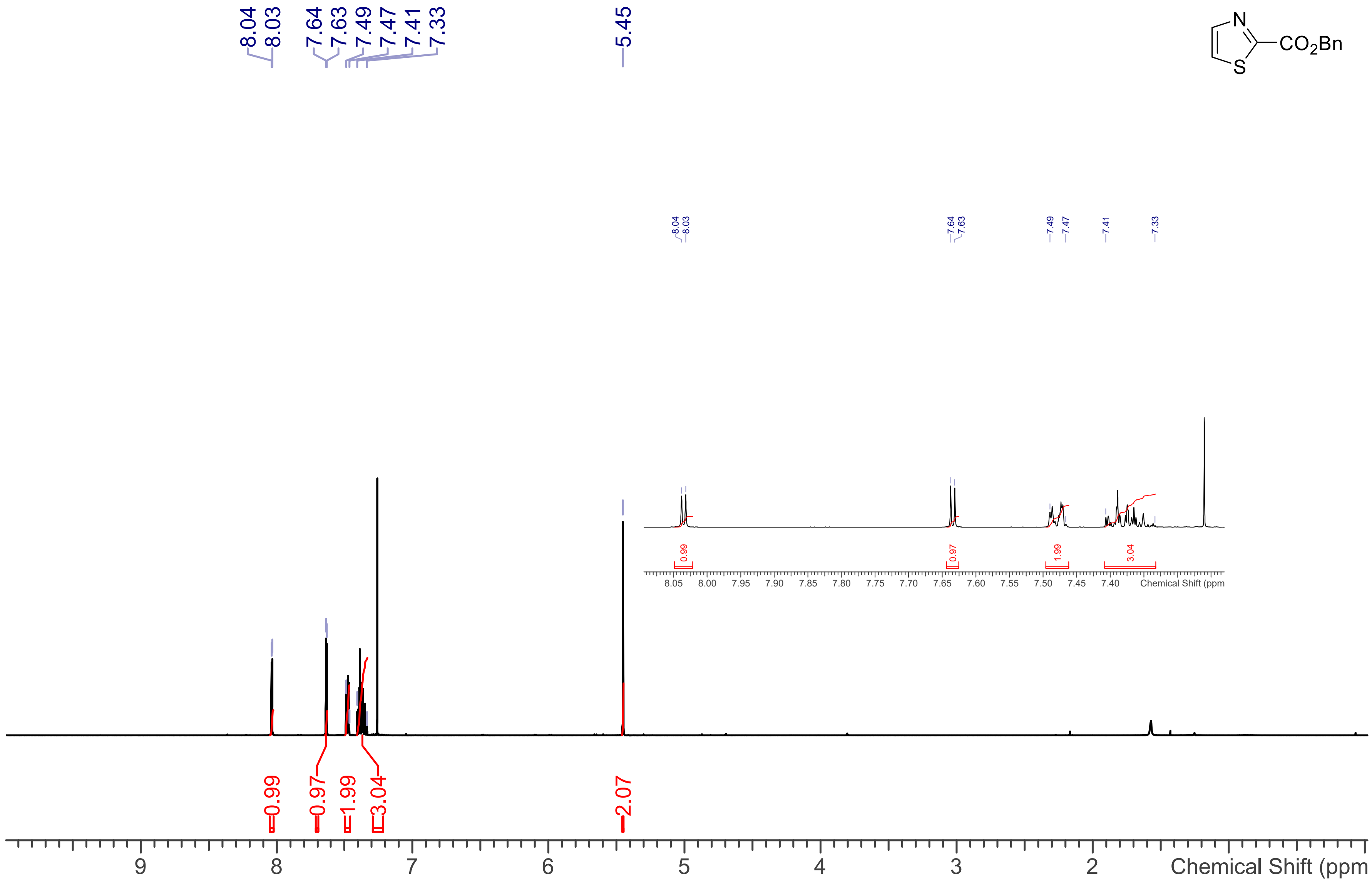

^{1}H NMR (500 MHz, $CDCl_3$) Benzyl thiazole-2-carboxylate (**3n**).

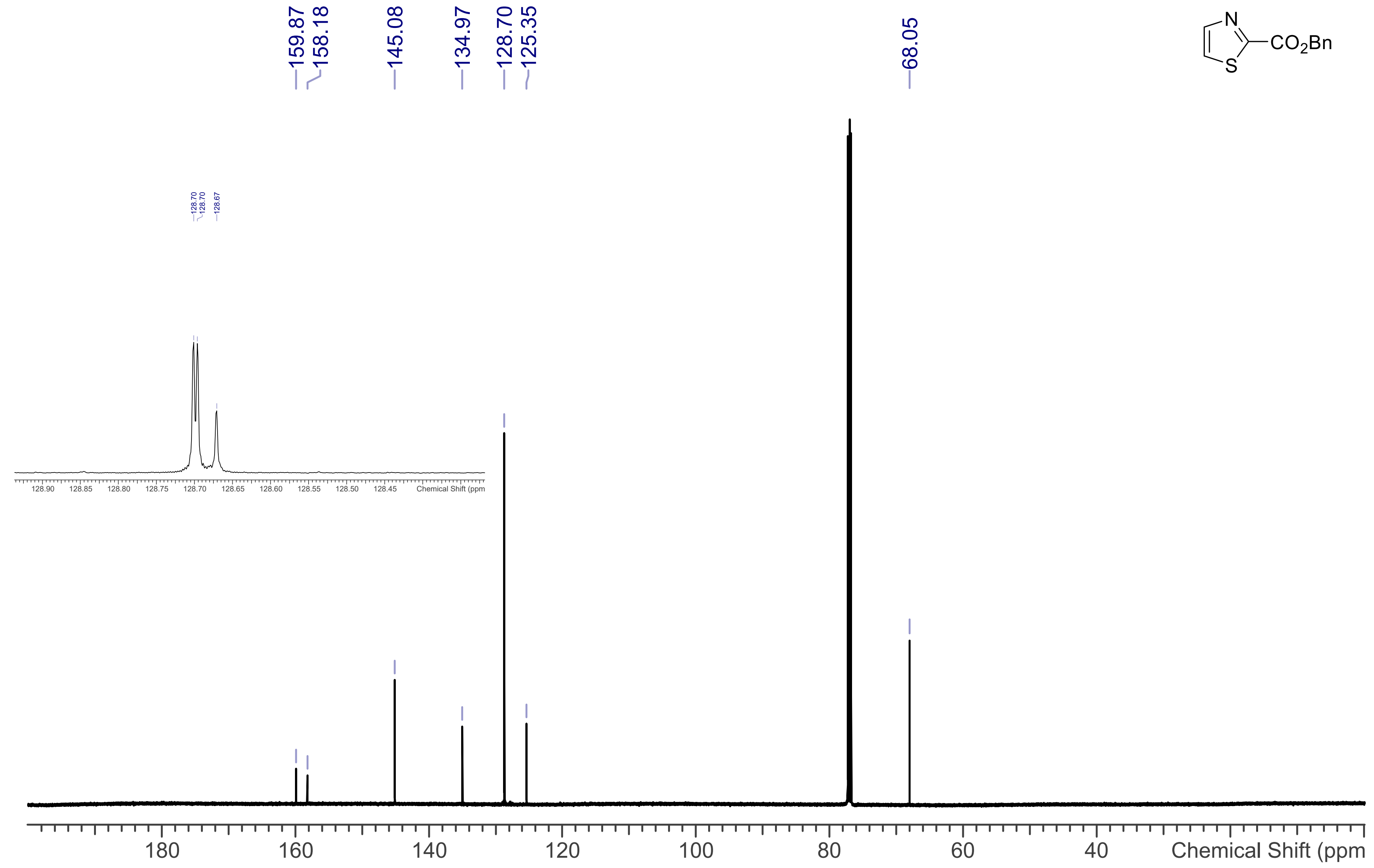

^{13}C NMR (125 MHz, $CDCl_3$) Benzyl thiazole-2-carboxylate (**3n**).

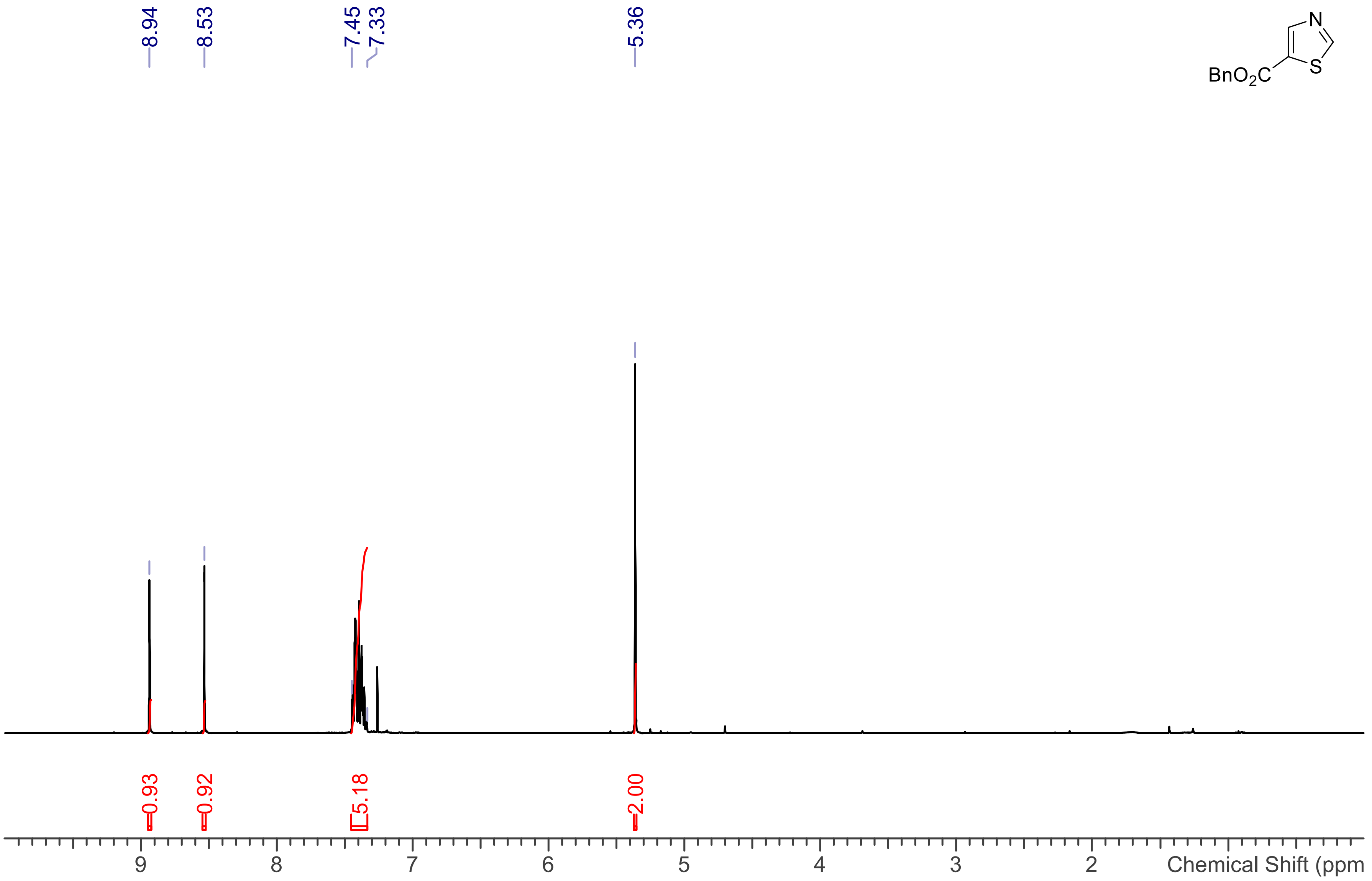

^{1}H NMR (400 MHz, $CDCl_3$) Benzyl thiazole-5-carboxylate (**3o**).

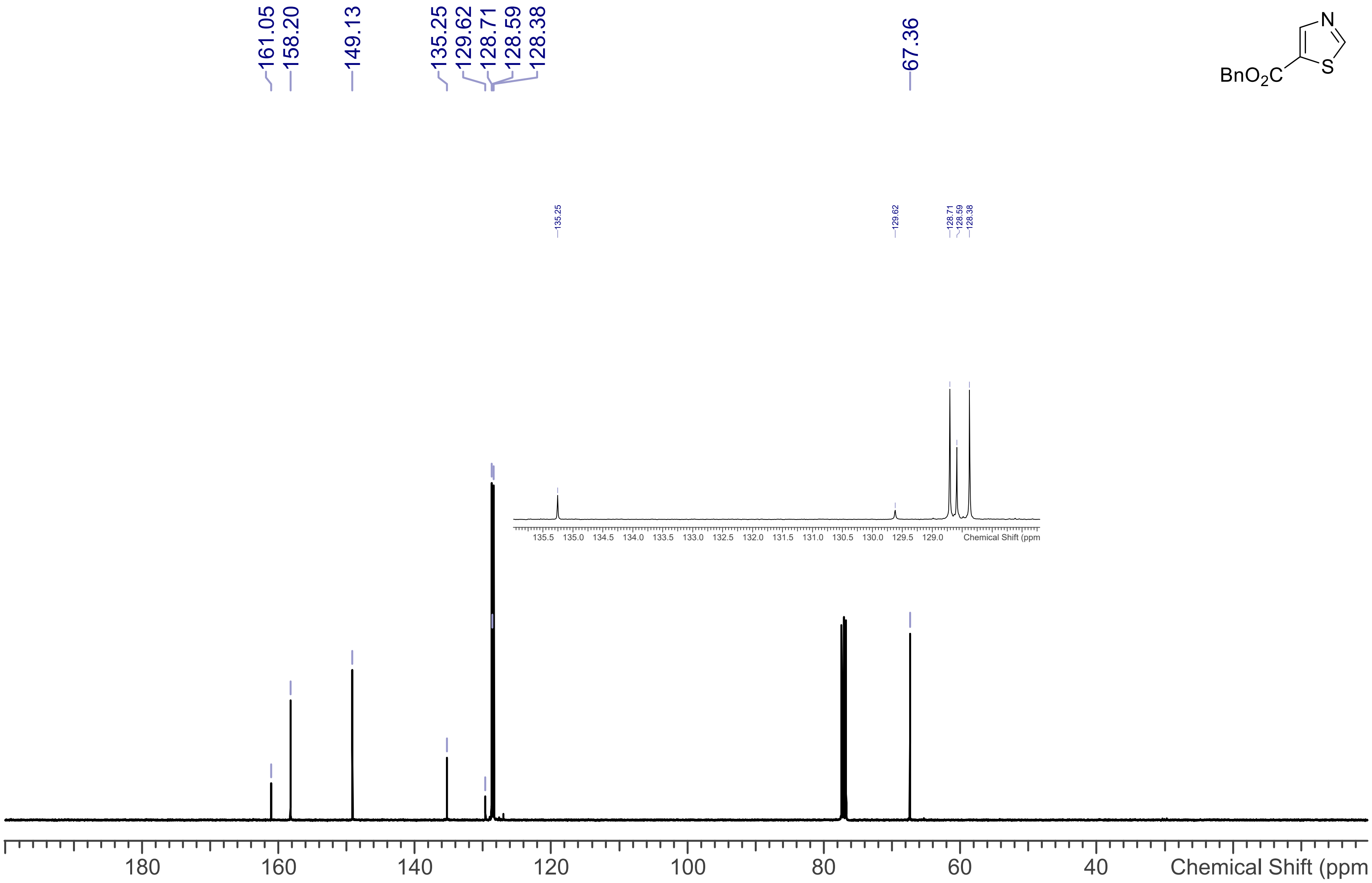

^{13}C NMR (101 MHz, $CDCl_3$) Benzyl thiazole-5-carboxylate (**3o**).

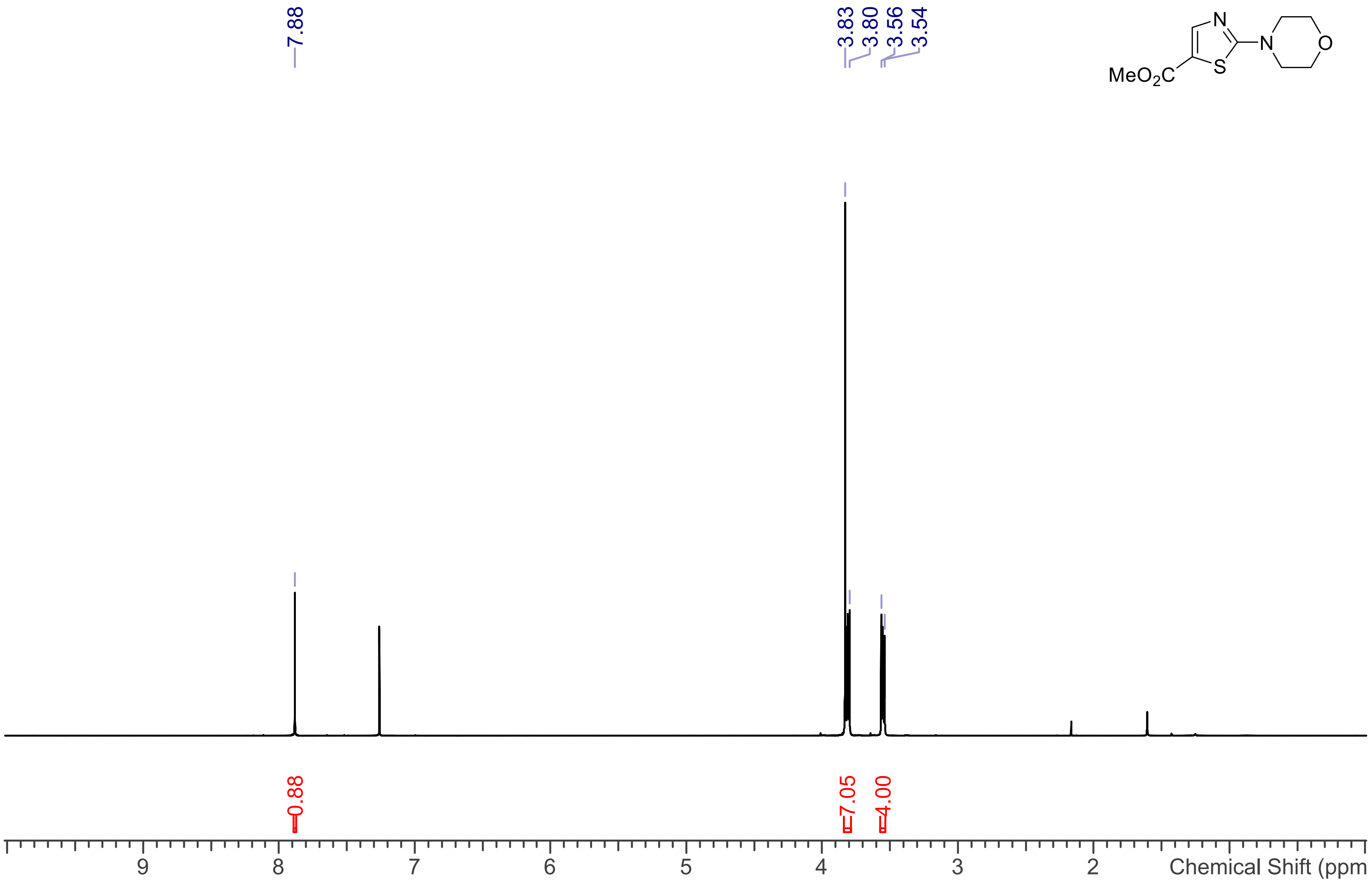

^{1}H NMR (400 MHz, $CDCl_3$) Methyl 2-morpholinothiazole-5-carboxylate (**3p**).

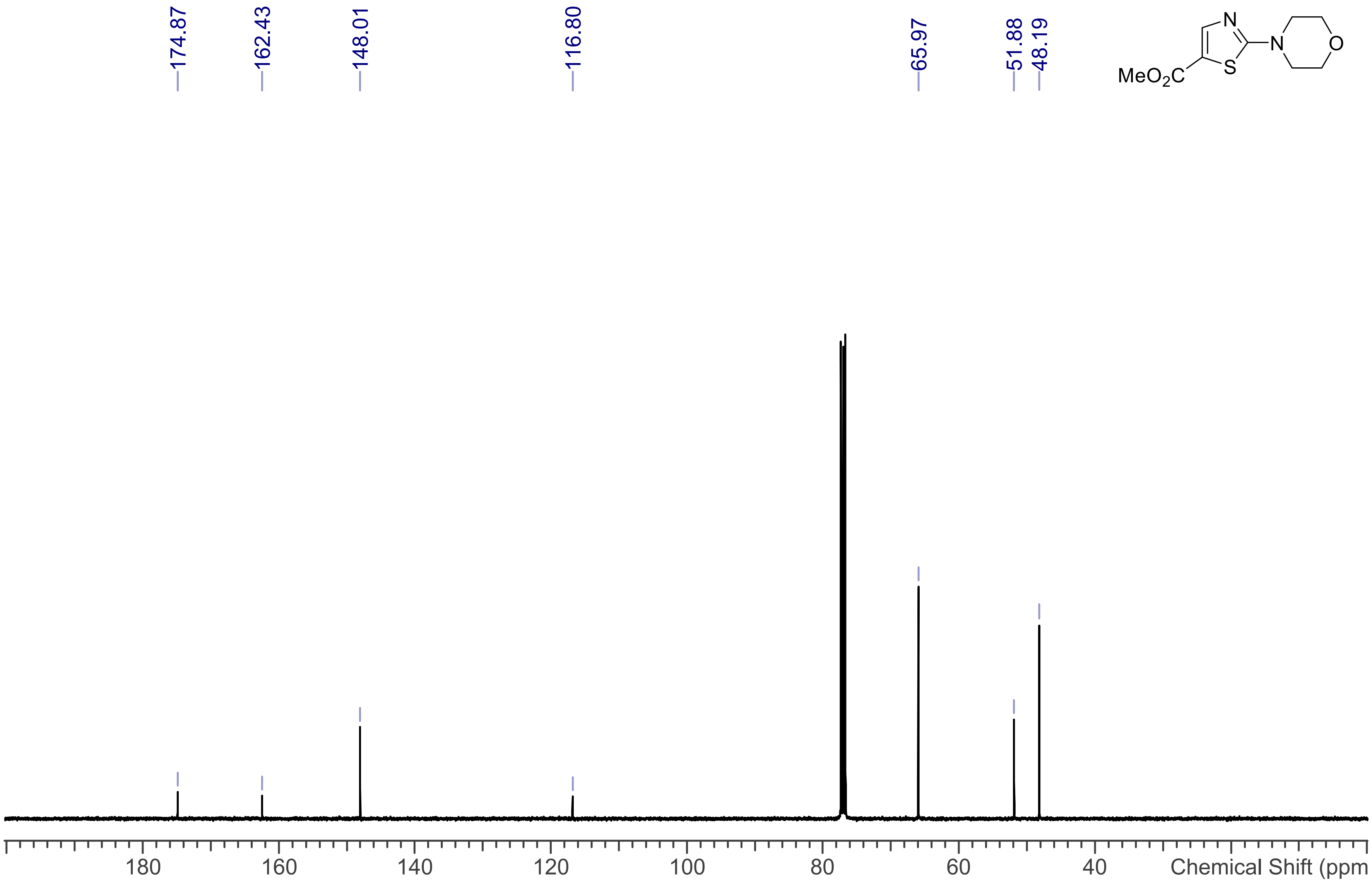

^{13}C NMR (101 MHz, $CDCl_3$) Methyl 2-morpholinothiazole-5-carboxylate (**3p**).

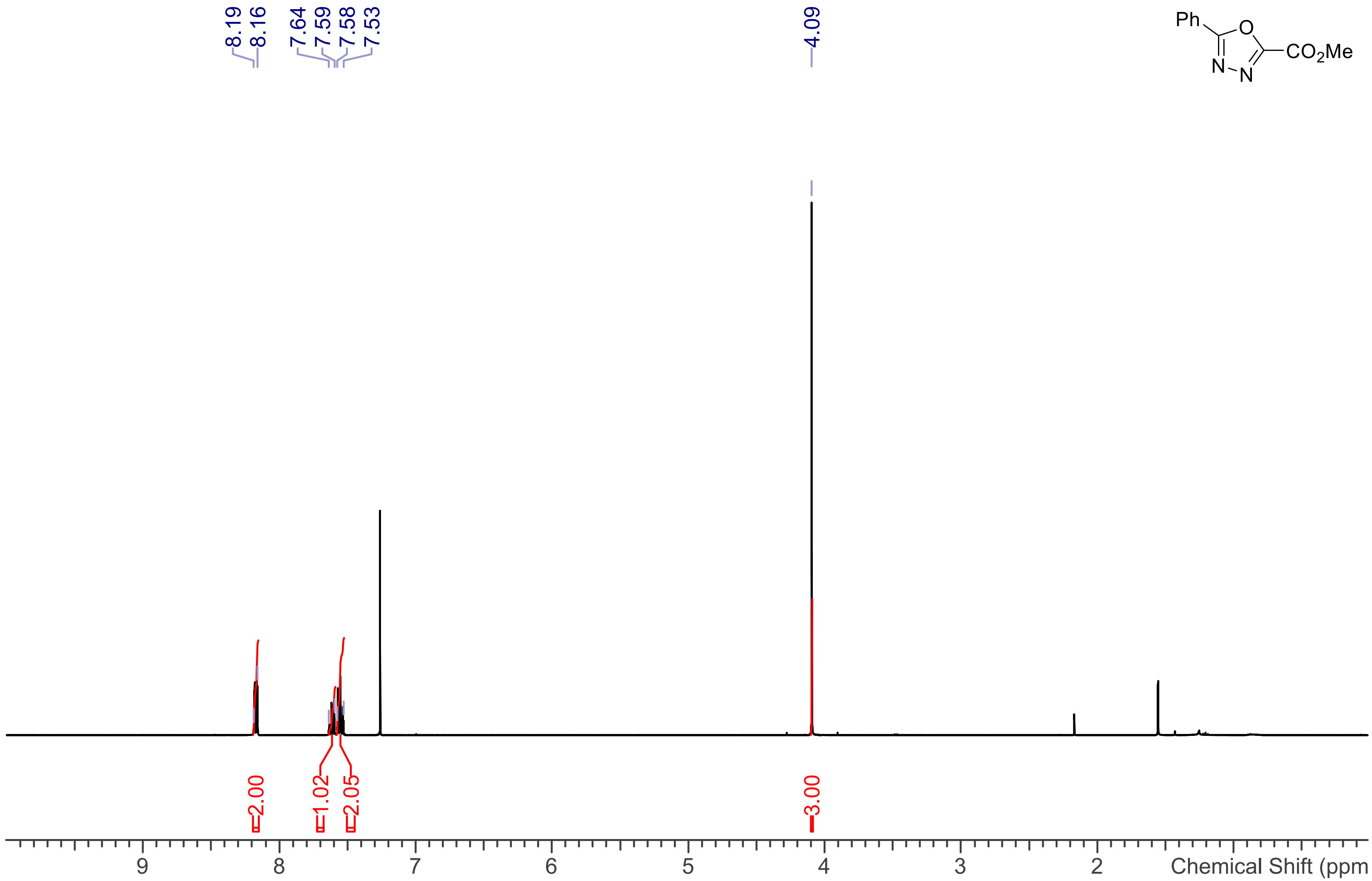

^{1}H NMR (400 MHz, $CDCl_3$) Methyl 5-phenyl-1,3,4-oxadiazole-2-carboxylate (**3q**).

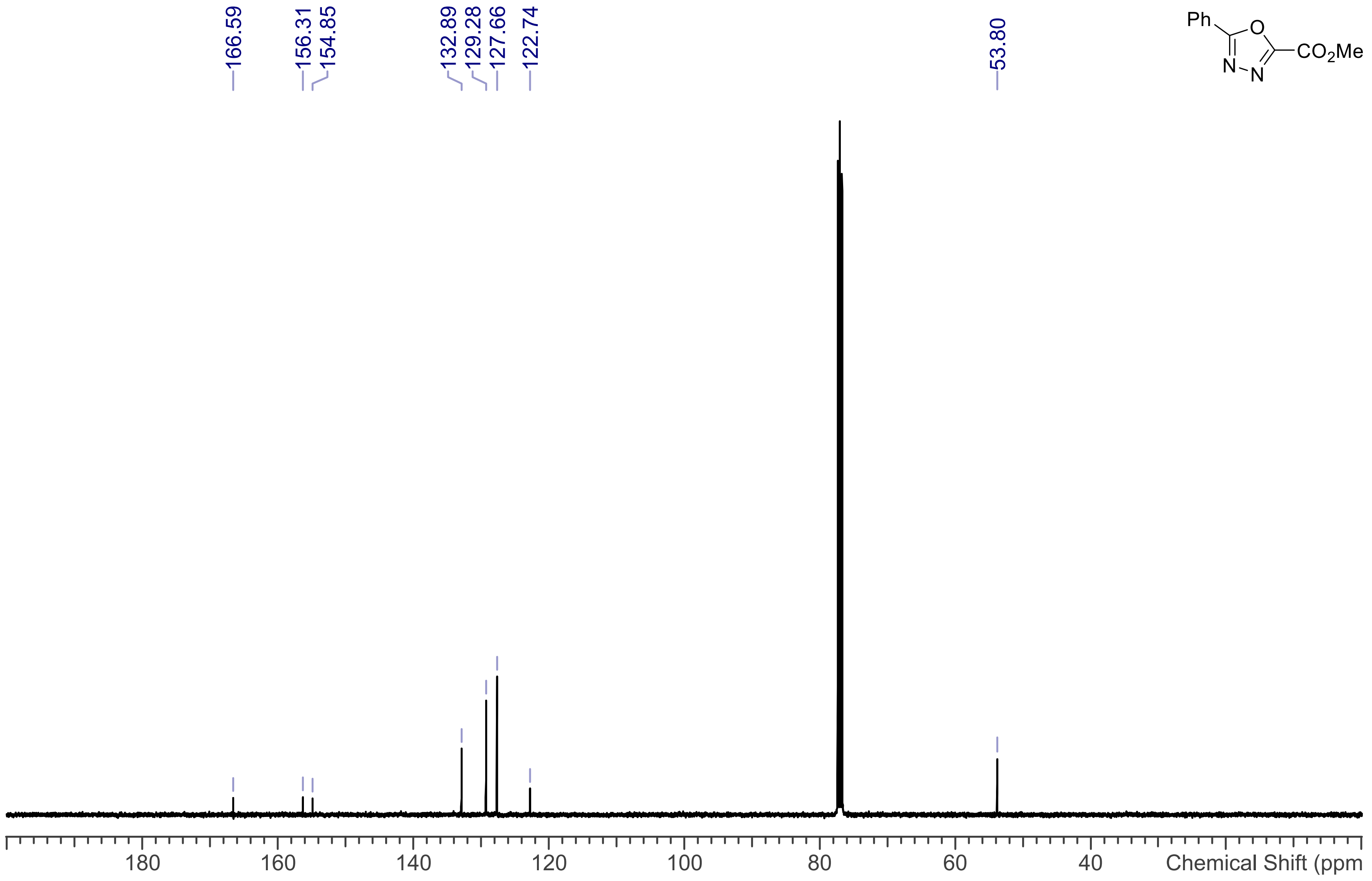

^{13}C NMR (101 MHz, $CDCl_3$) Methyl 5-phenyl-1,3,4-oxadiazole-2-carboxylate (**3q**).

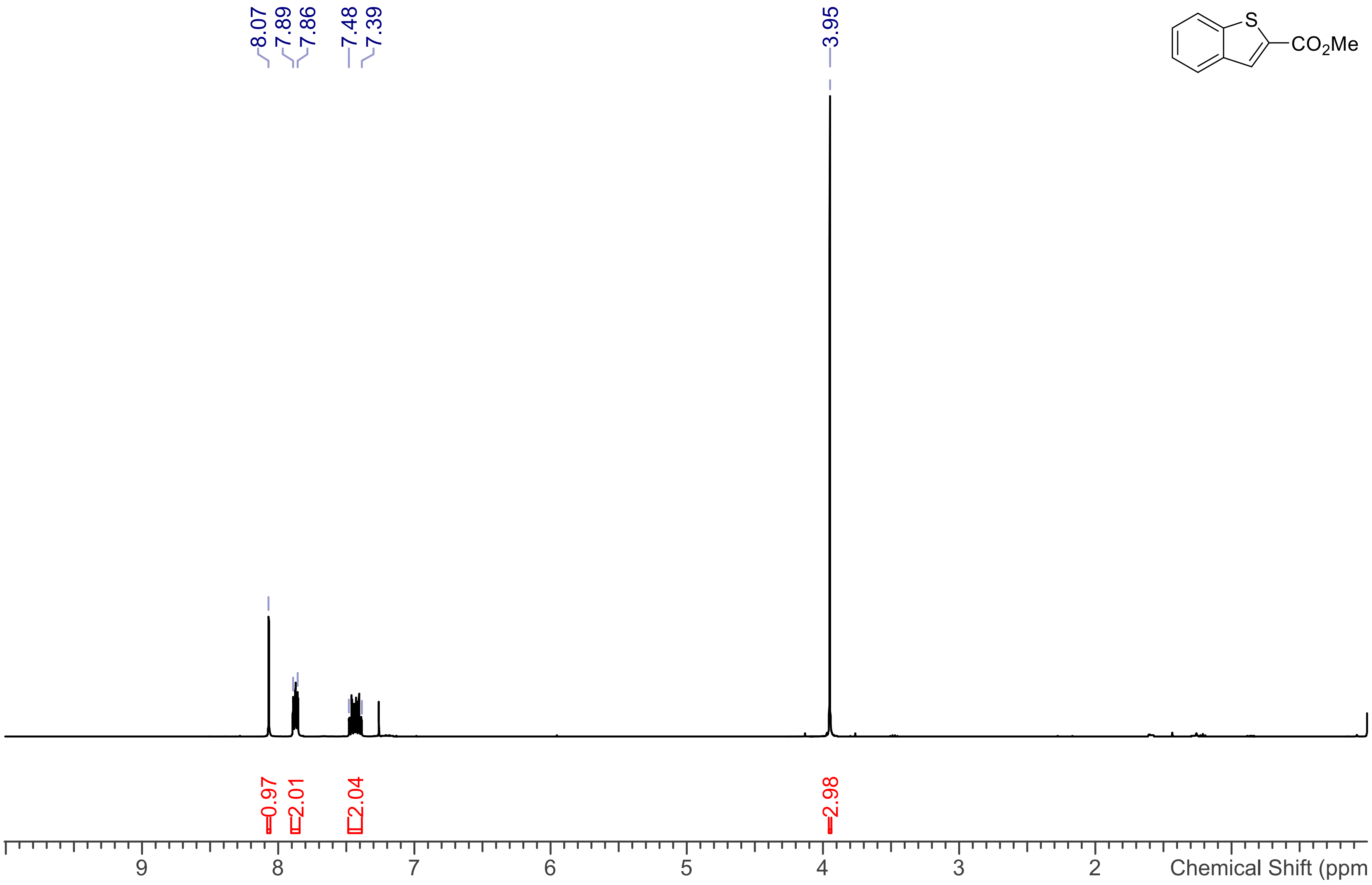

^{1}H NMR (400 MHz, $CDCl_3$) Methyl Benzo[*b*]thiophene-2-carboxylate (**3t**)

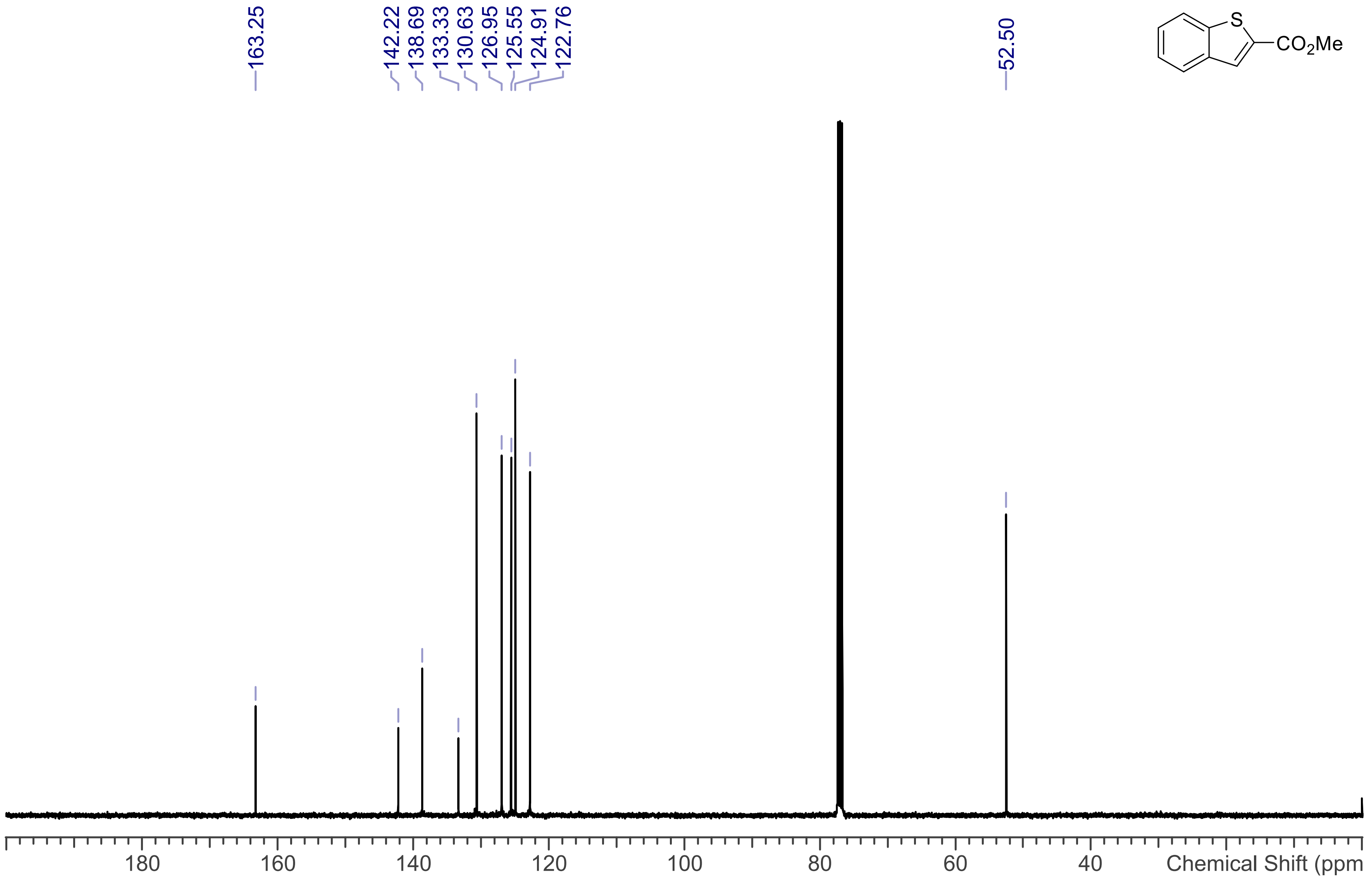

^{13}C NMR (101 MHz, $CDCl_3$) Methyl Benzo[*b*]thiophene-2-carboxylate (**3t**).

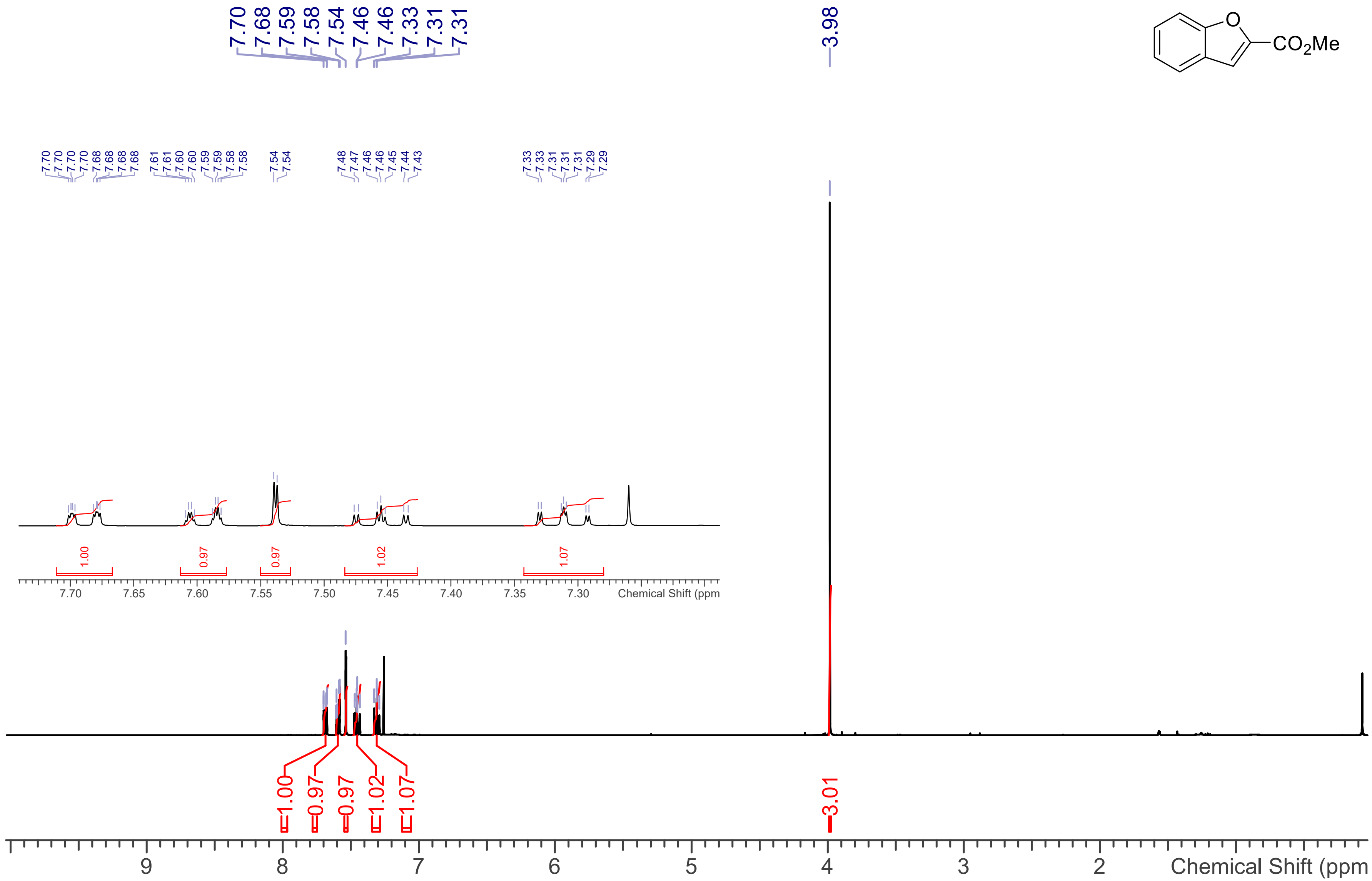

^{1}H NMR (400 MHz, $CDCl_3$) Methyl Benzofuran-2-carboxylate (**3u**).

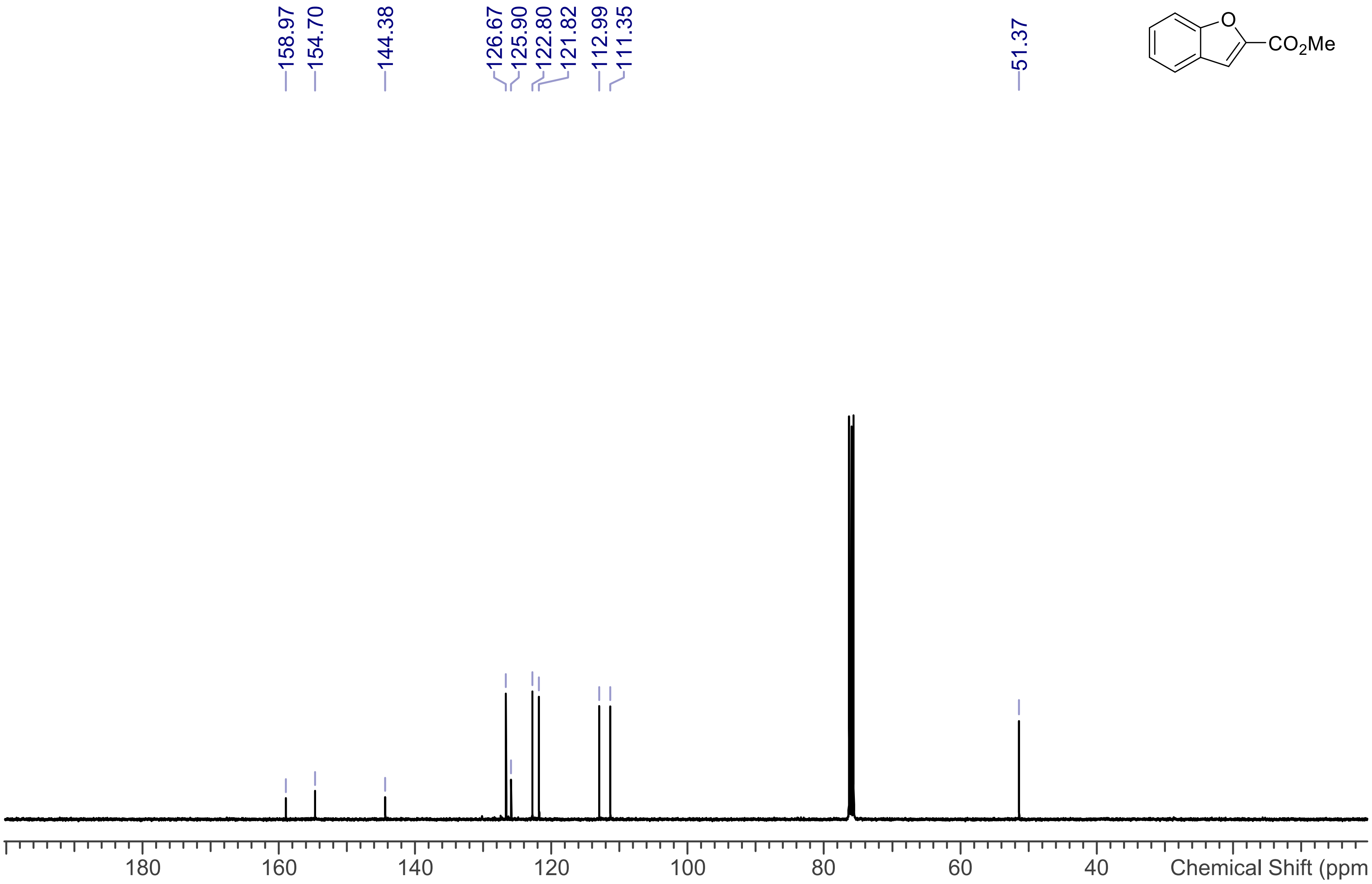

^{13}C NMR (101 MHz, $CDCl_3$) Methyl Benzofuran-2-carboxylate (**3u**).

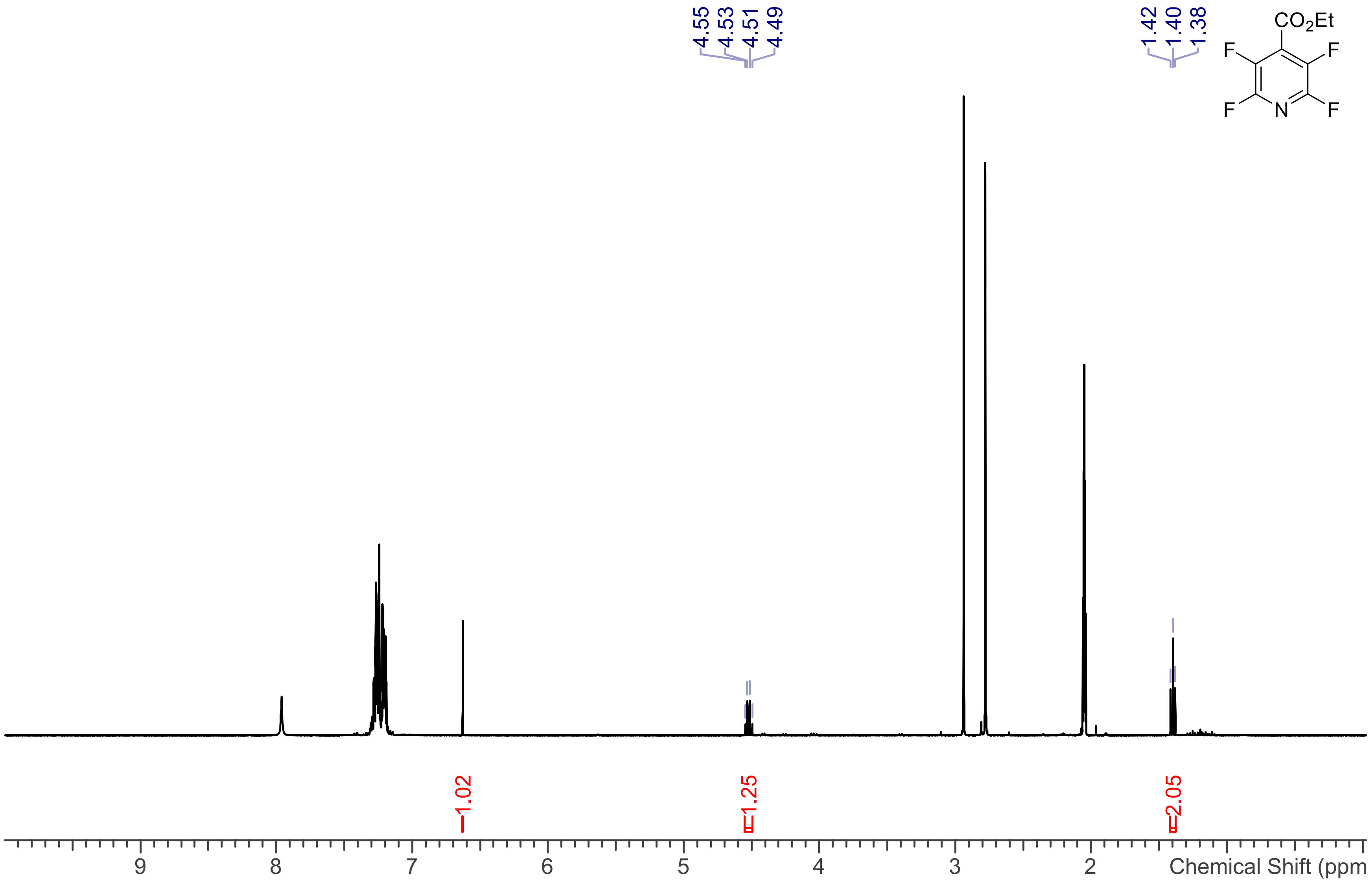

Crude ^{1}H NMR (400 MHz, $(CD_3)_2CO$) Ethyl tetrafluoroisonicotinate (**3v**). 1,1,2,2-tetrachloroethane as an internal standard.

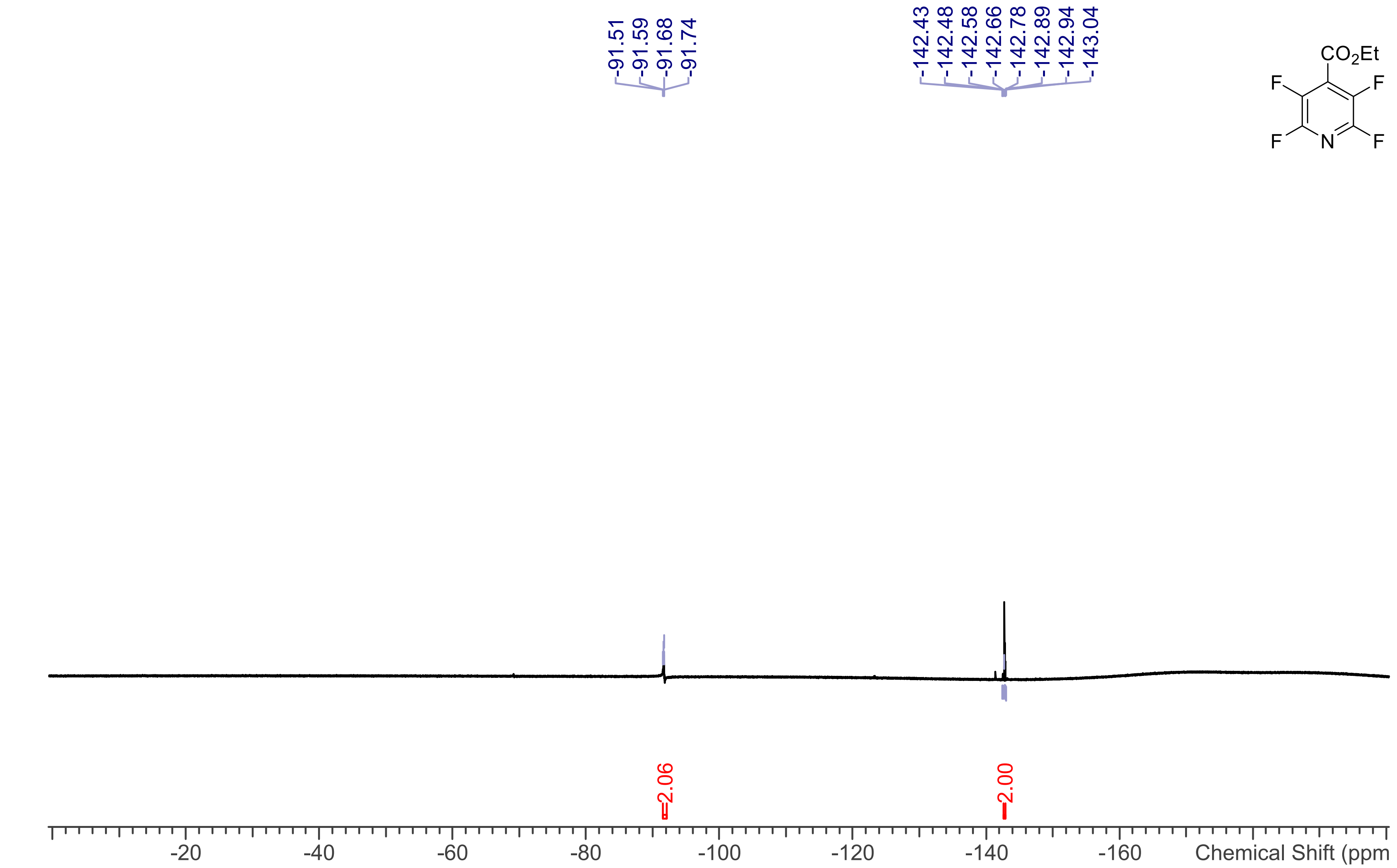

Crude $^{19}F\{^1H\}$ NMR (376 MHz, $(CD_3)_2CO$) Ethyl tetrafluoroisonicotinate (**3v**).

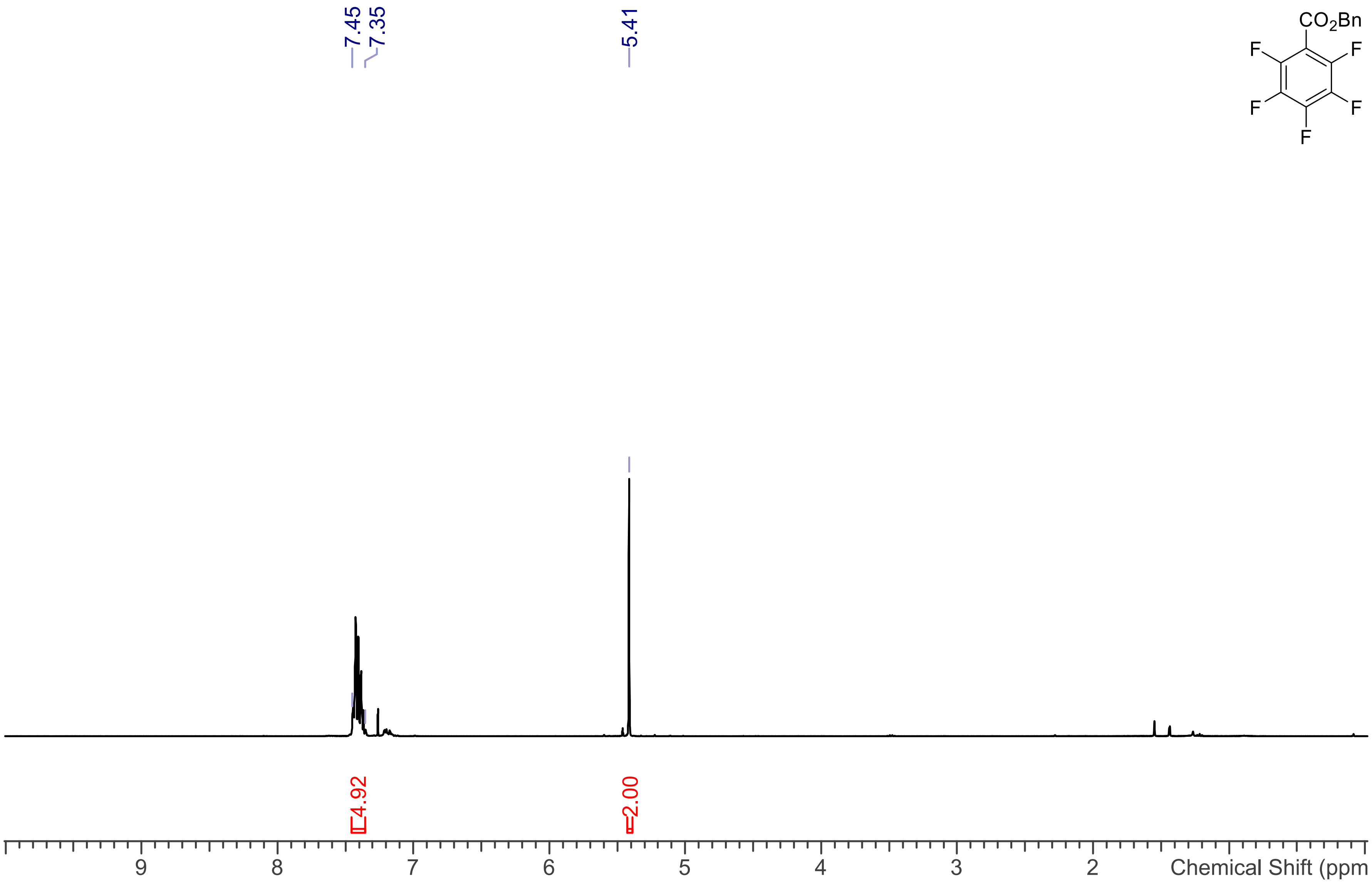

^{1}H NMR (400 MHz, $CDCl_3$) Benzyl 2,3,4,5,6-pentafluorobenzoate (**3w**).

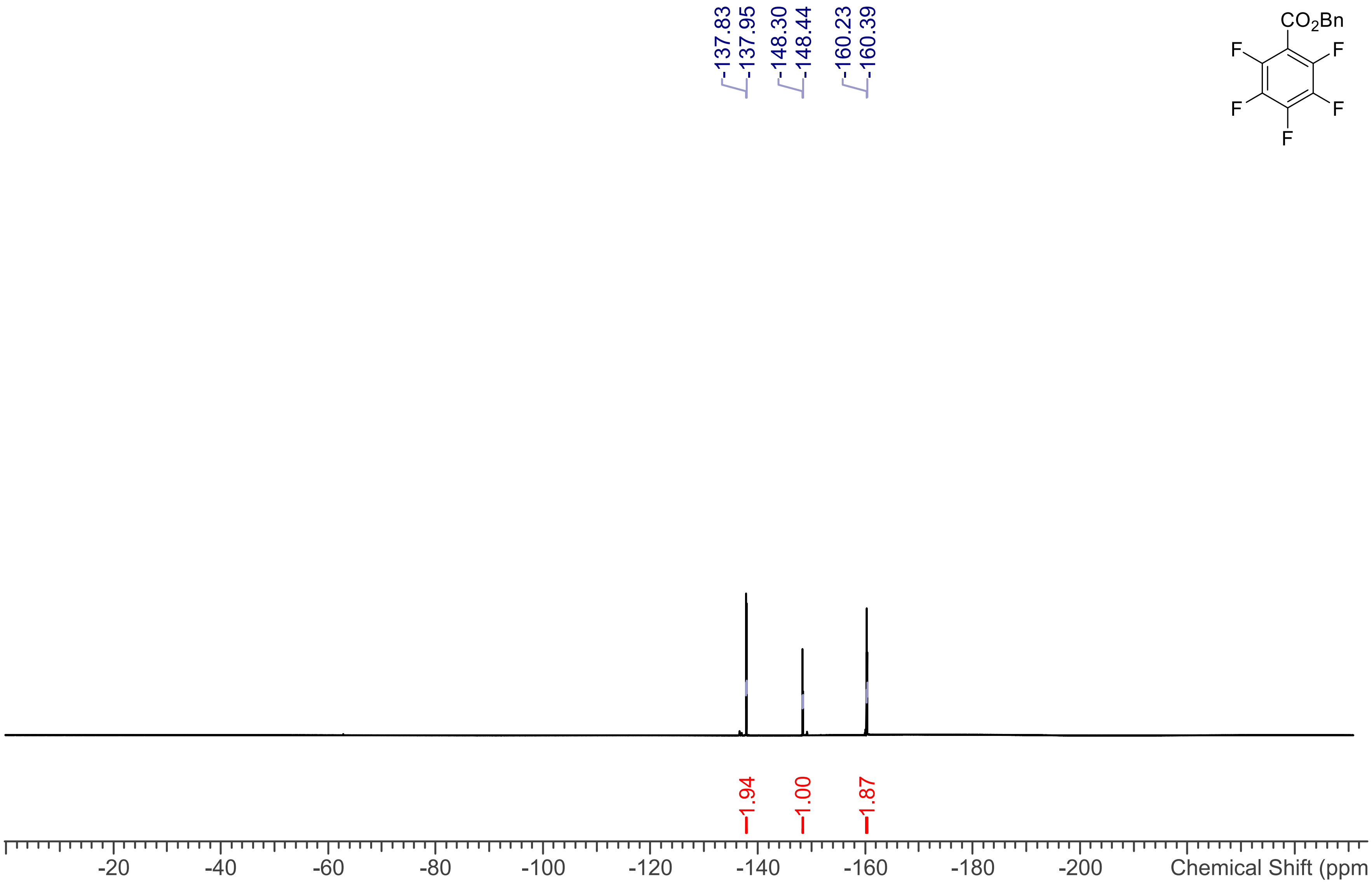

$^{19}F\{^1H\}$ NMR (376 MHz, $CDCl_3$) Benzyl 2,3,4,5,6-pentafluorobenzoate (**3w**).

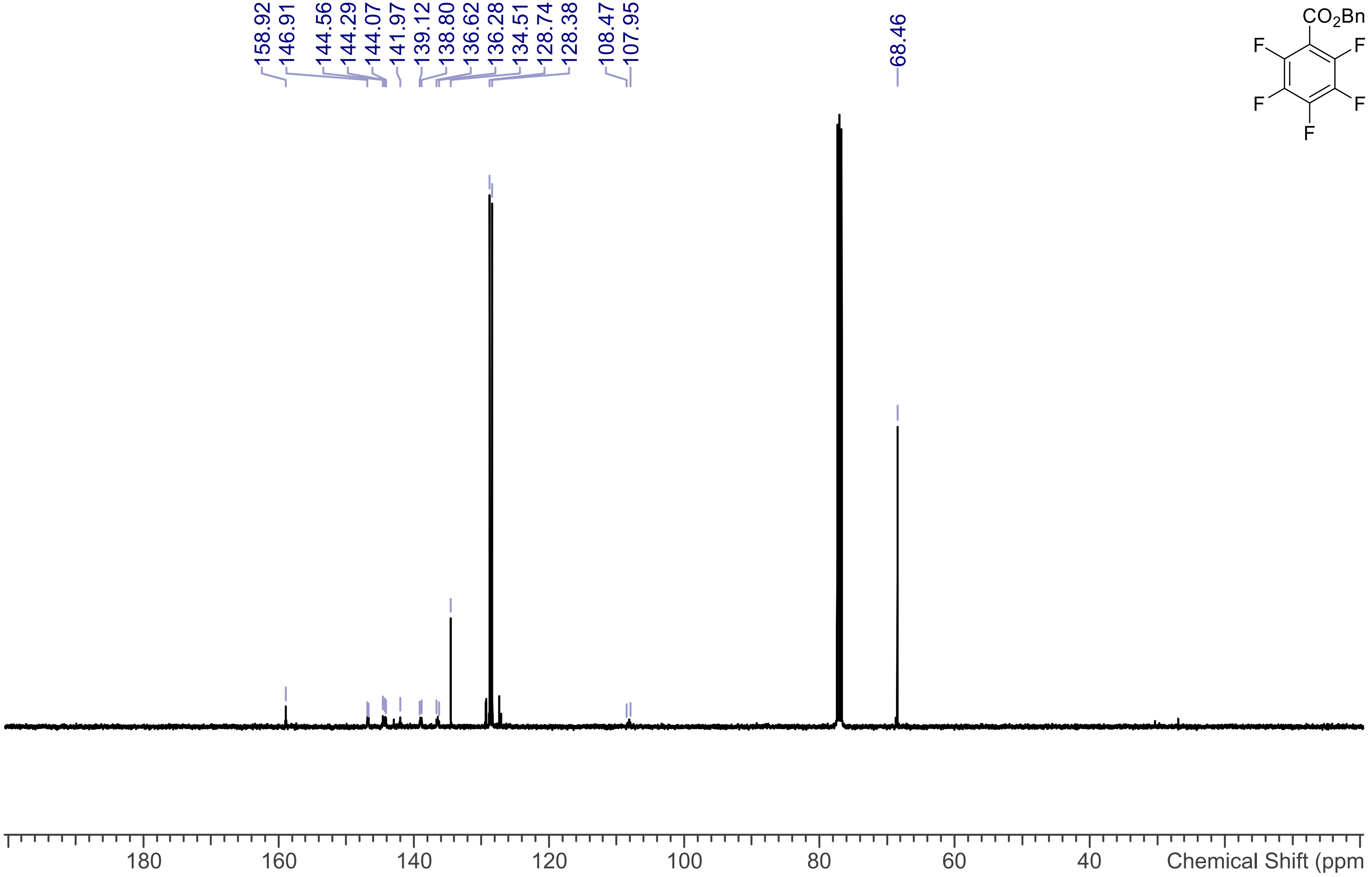

^{13}C NMR (101 MHz, CDCl$_3$) Benzyl 2,3,4,5,6-pentafluorobenzoate (**3w**).

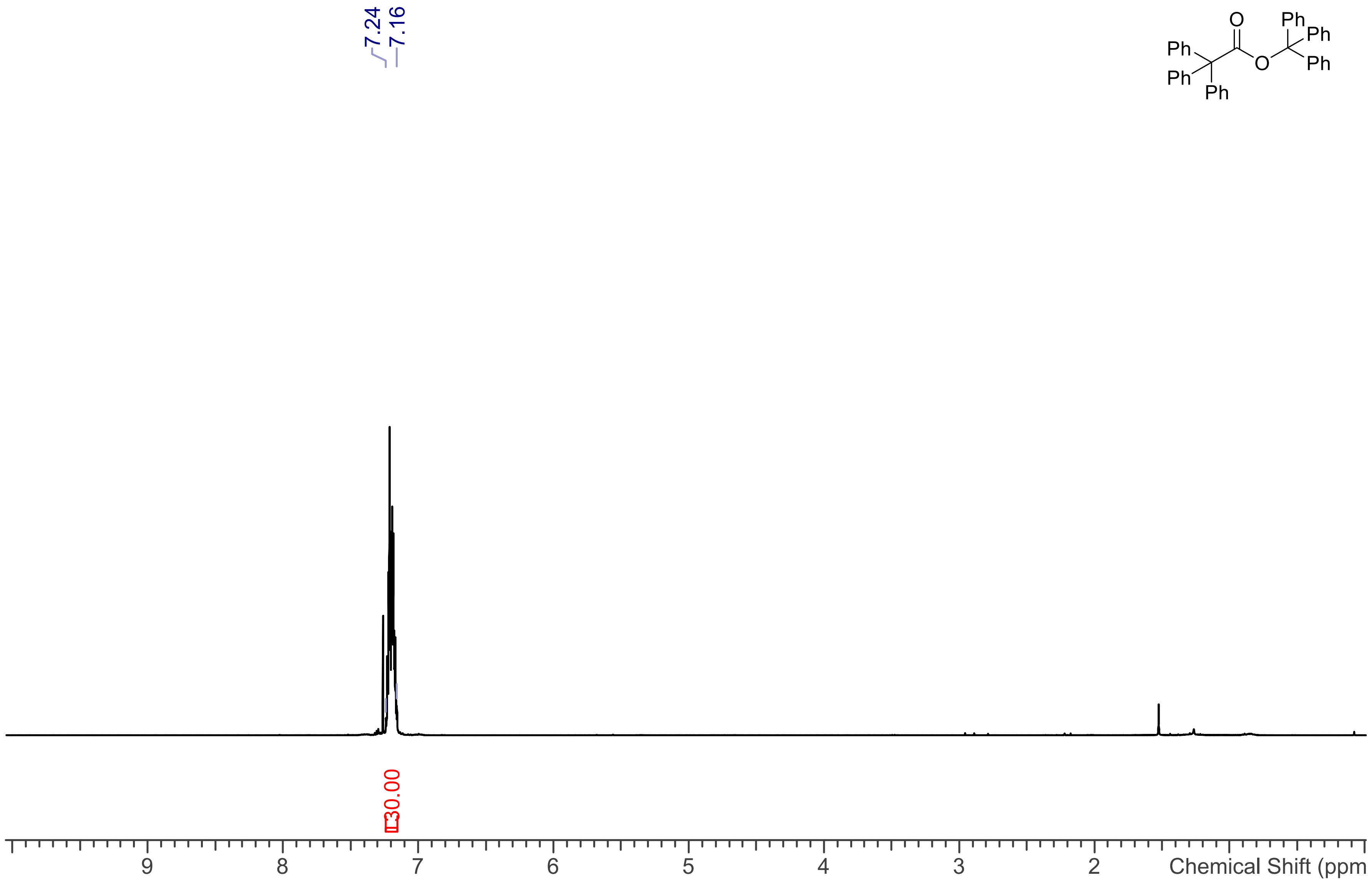

^{1}H NMR (400 MHz, $CDCl_3$) Trityl 2,2,2-triphenyl acetate (**6**).

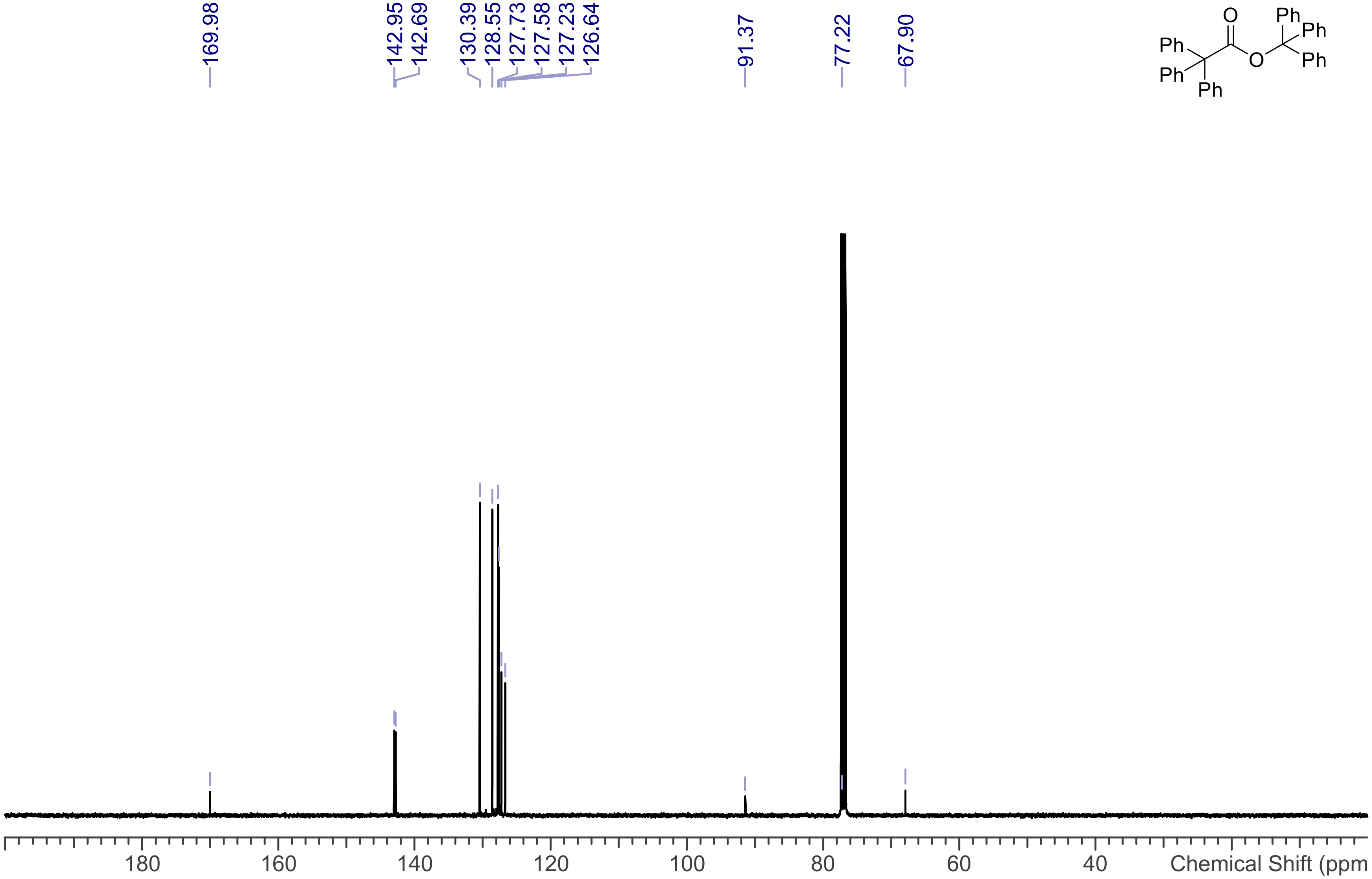

^{13}C NMR (101 MHz, $CDCl_3$) Trityl 2,2,2-triphenylacetate (**6**).

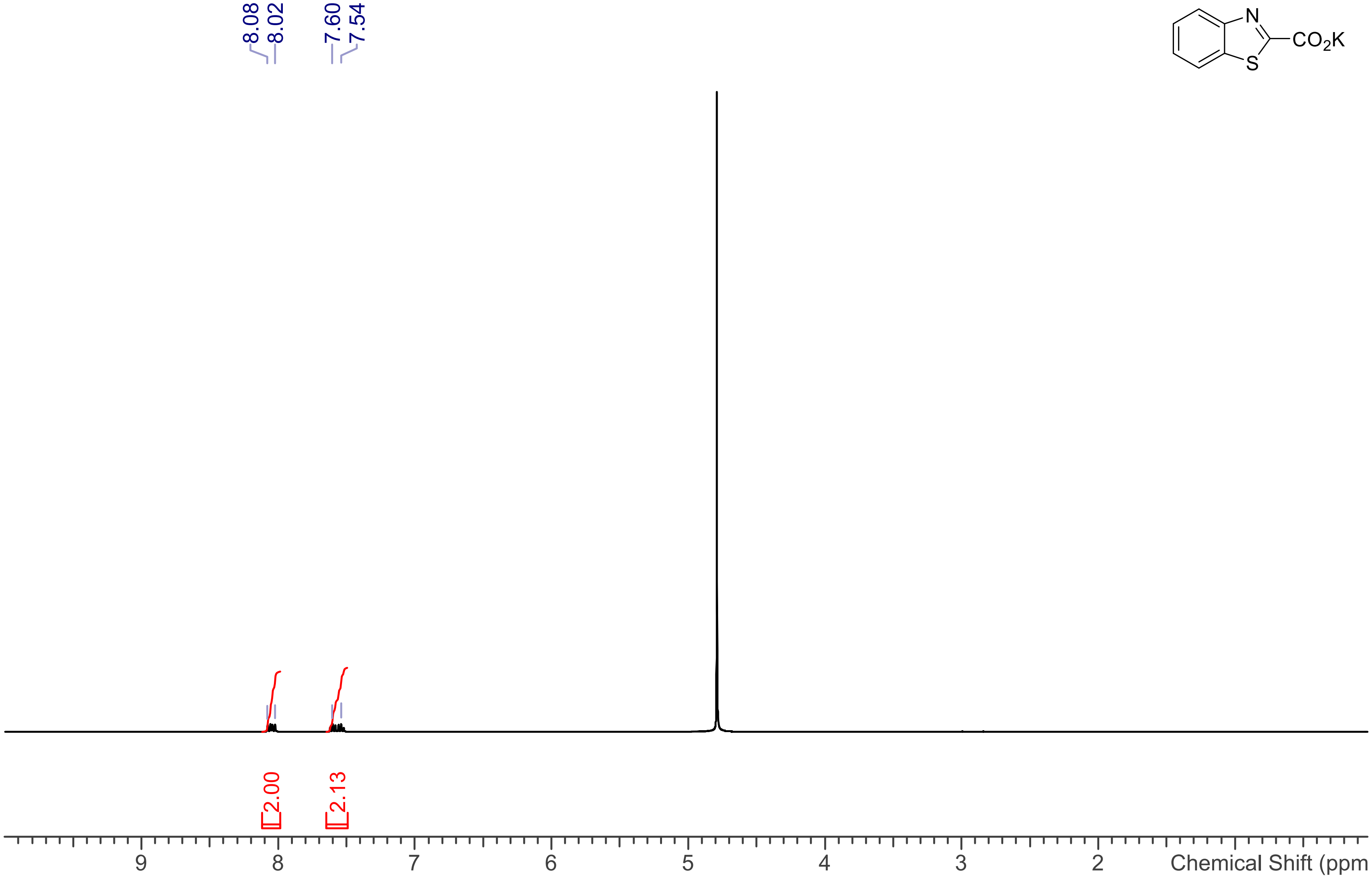

^{1}H NMR (400 MHz, D_2O) Potassium benzothiazole-2-carboxylate.

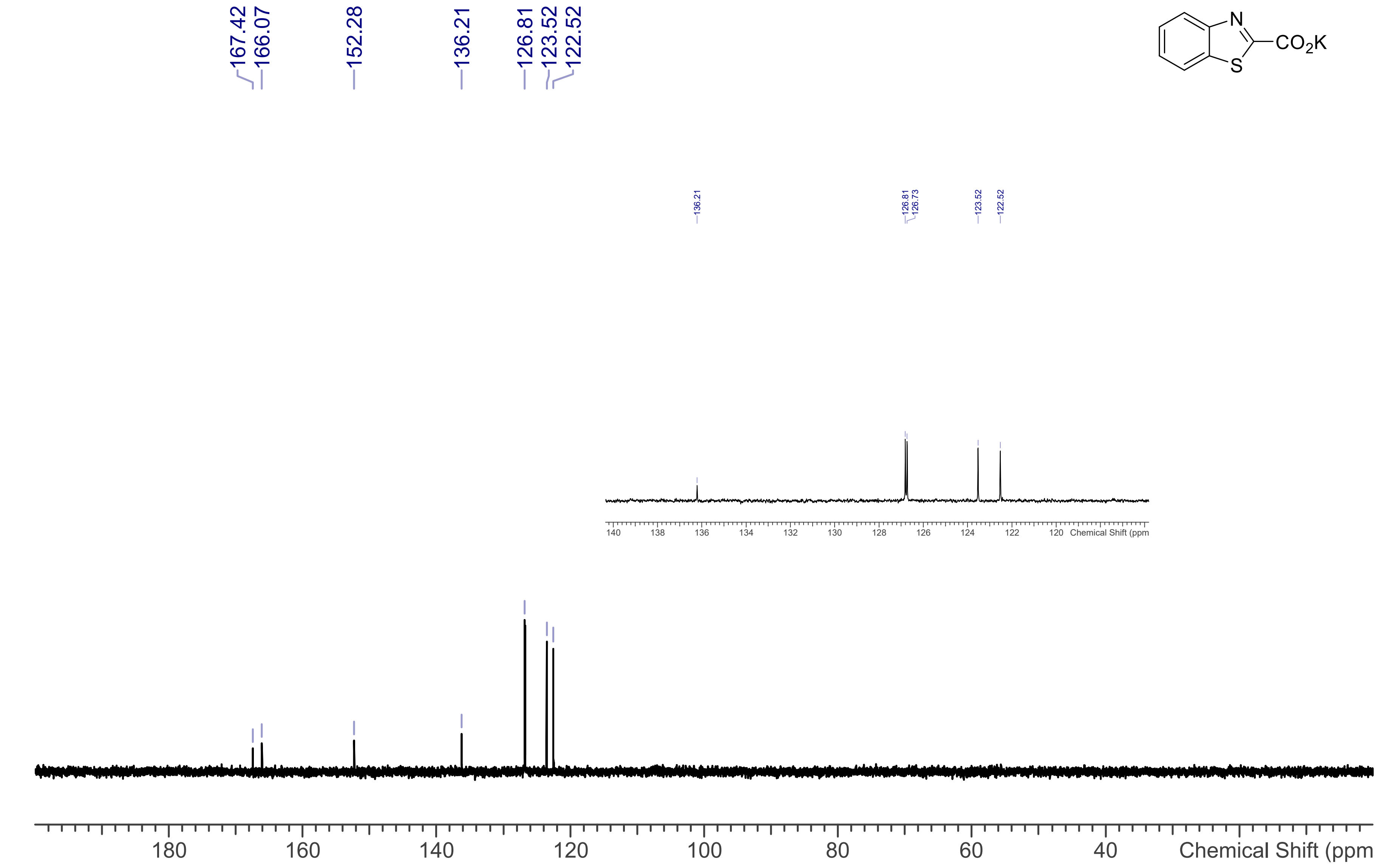

^{13}C NMR (101 MHz, D_2O) Potassium benzothiazole-2-carboxylate.

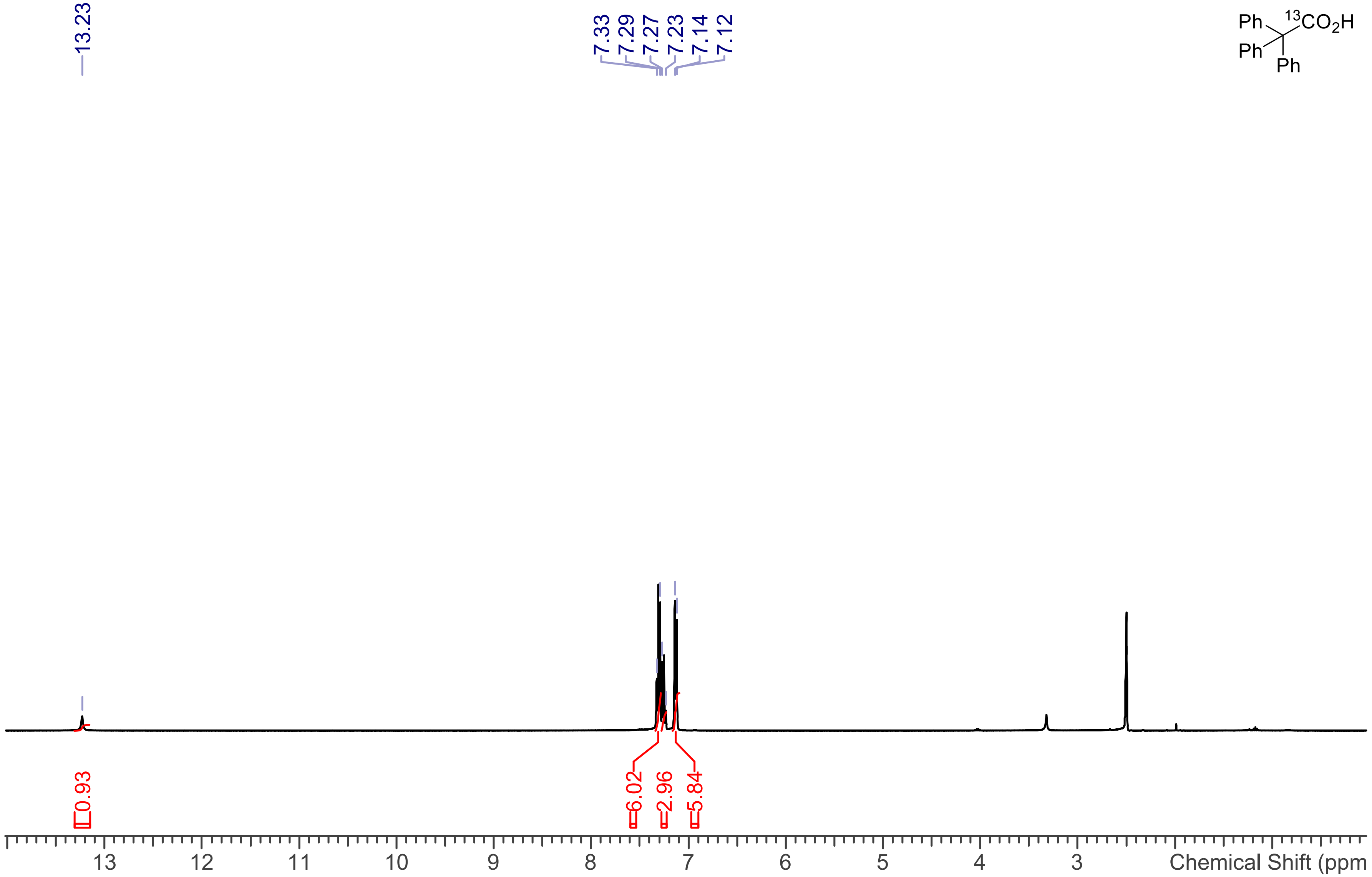

^{1}H NMR (101 MHz, DMSO-d_6) [^{13}C]Triphenylacetic acid. (**2’***)

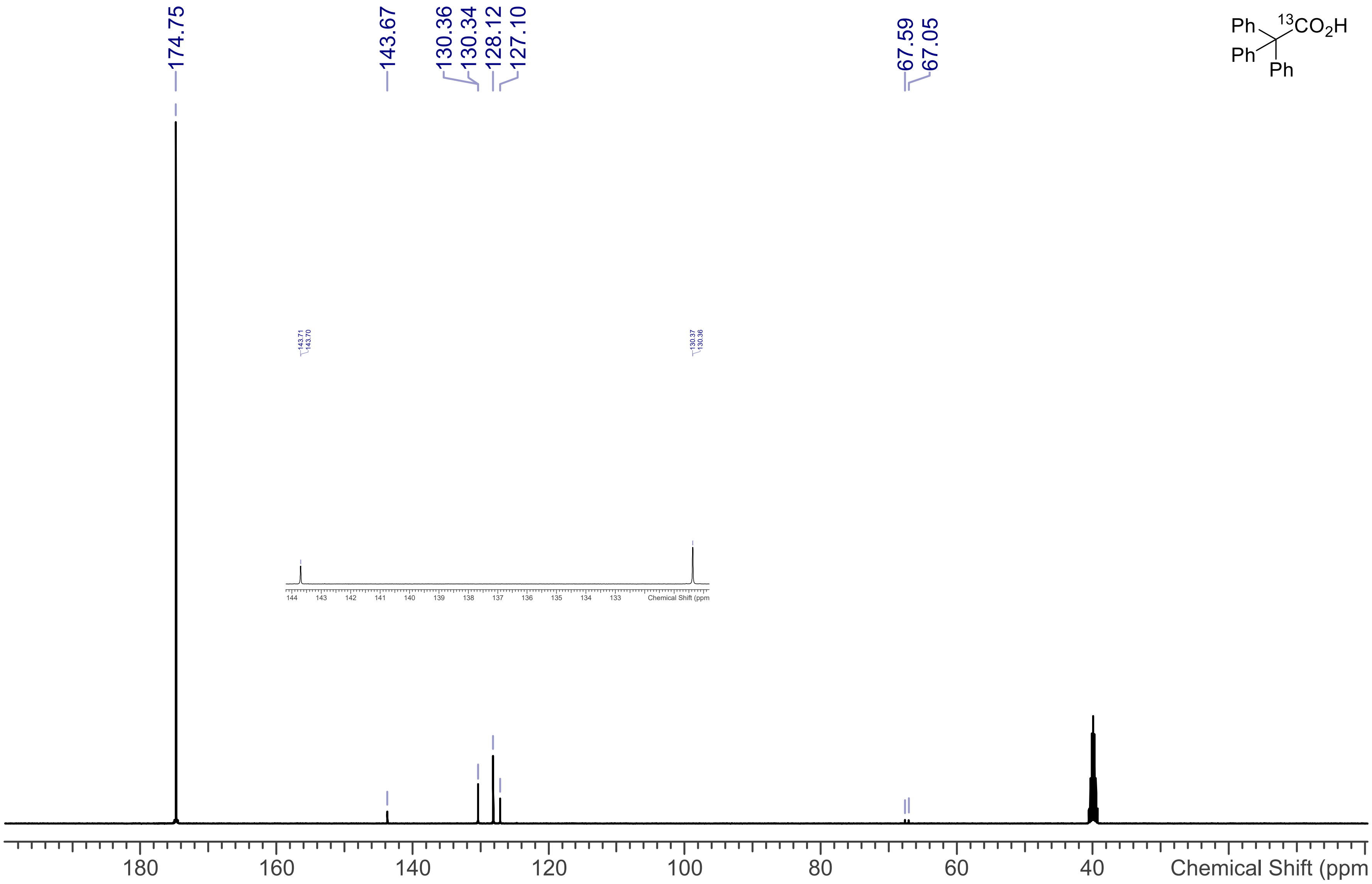

^{13}C NMR (125 MHz, DMSO-d6) [^{13}C]Triphenylacetic acid. (**2’***)

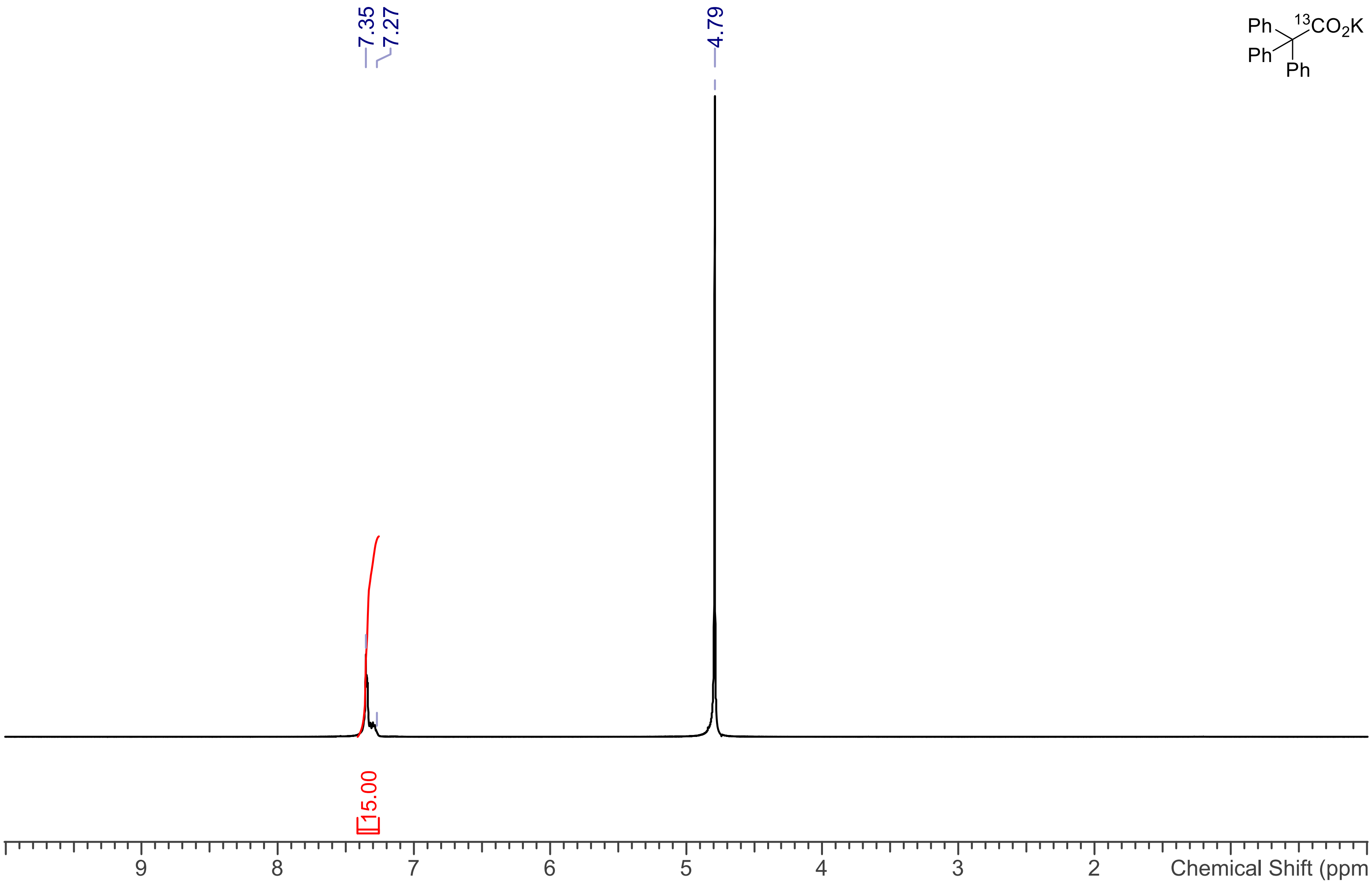

^{1}H NMR (400 MHz, D_2O) [^{13}C]Potassium 2,2,2-triphenyl acetate (**2-K***).

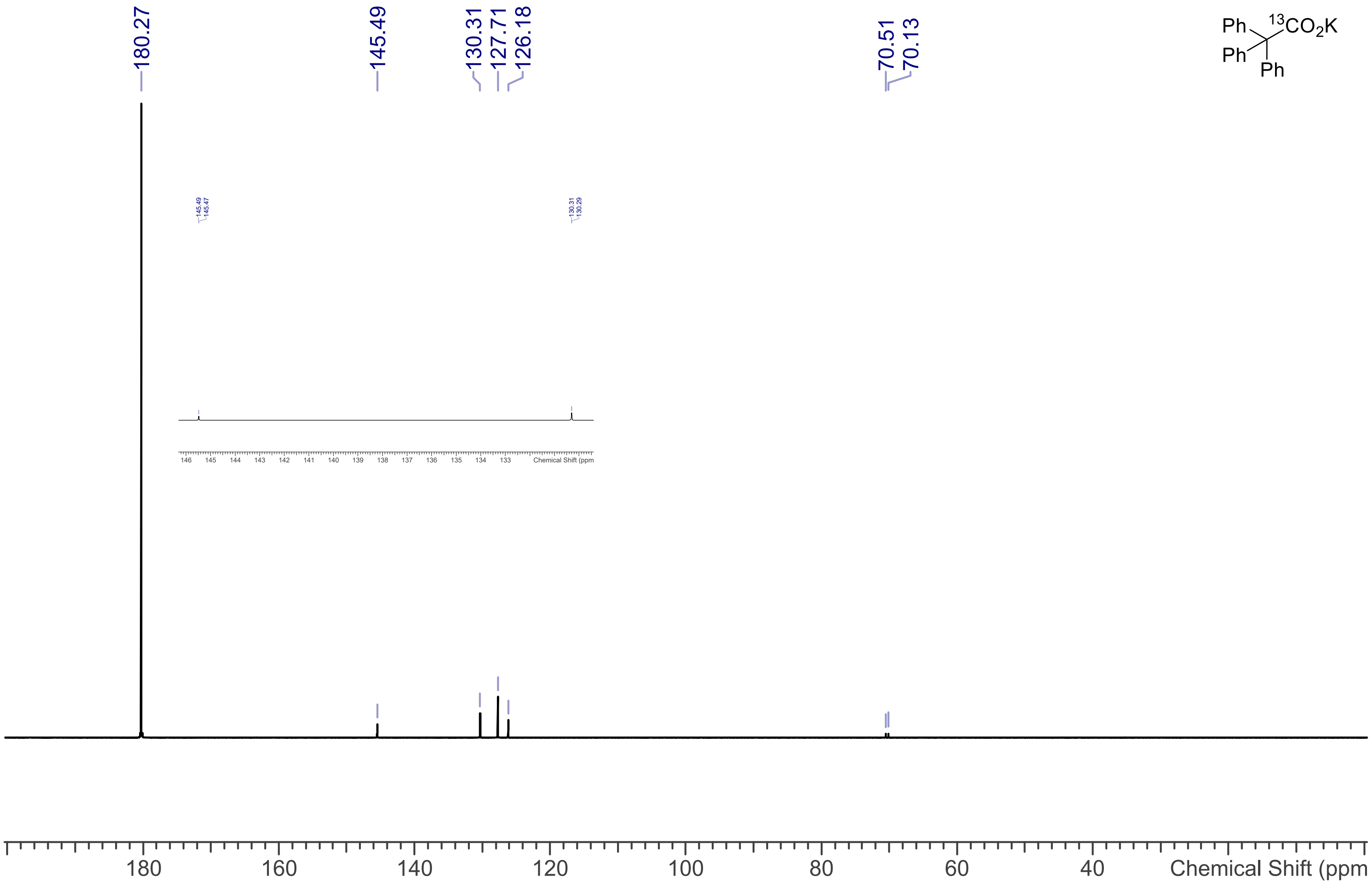

^{13}C NMR (125 MHz, D_2O) [^{13}C]Potassium 2,2,2-triphenyl acetate (**2-K***).

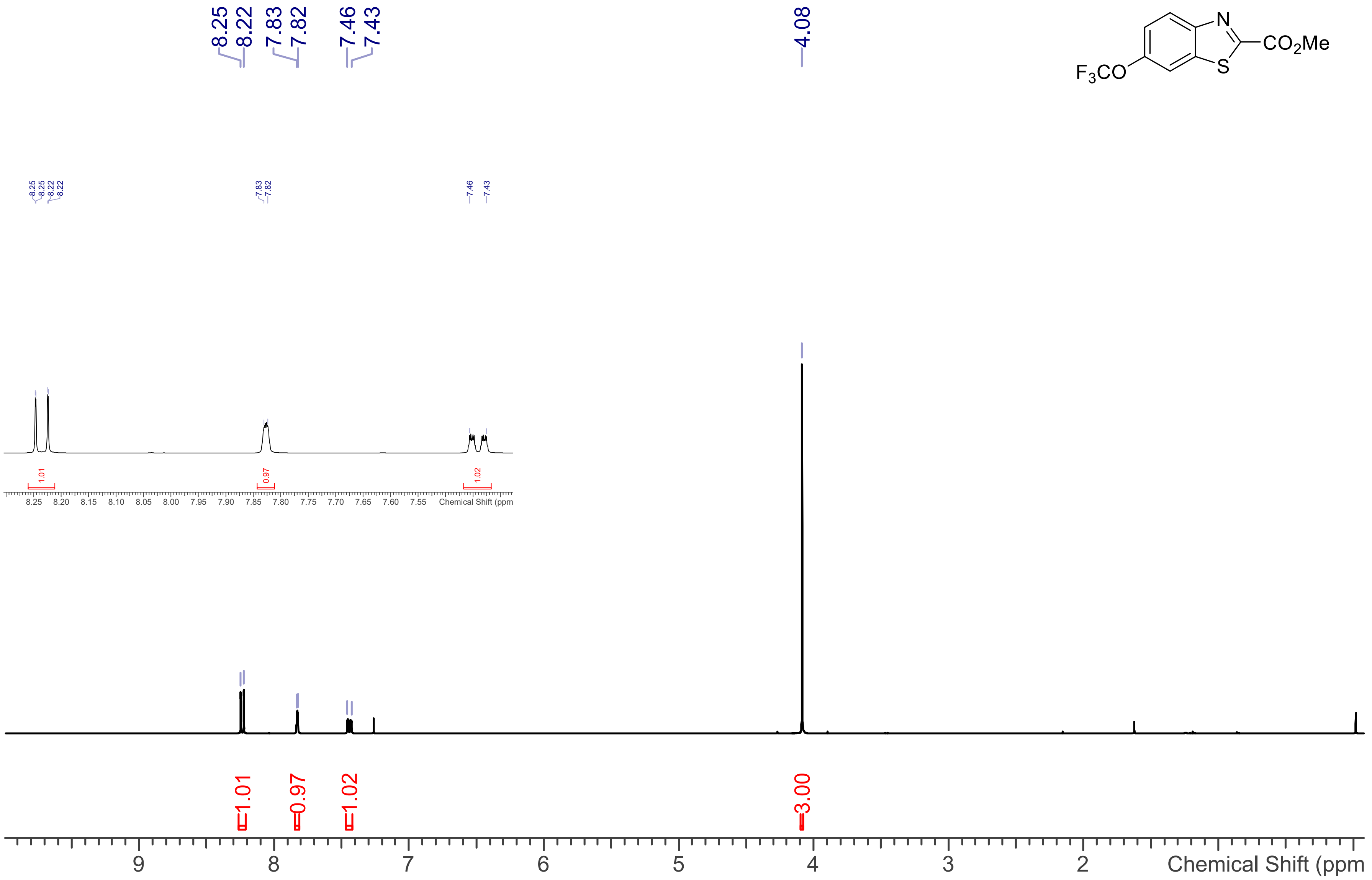

^{1}H NMR (400 MHz, $CDCl_3$) Methyl 6-Trifluoromethoxybenzo[*d*]thiazole-2-carboxylate (**3x**).

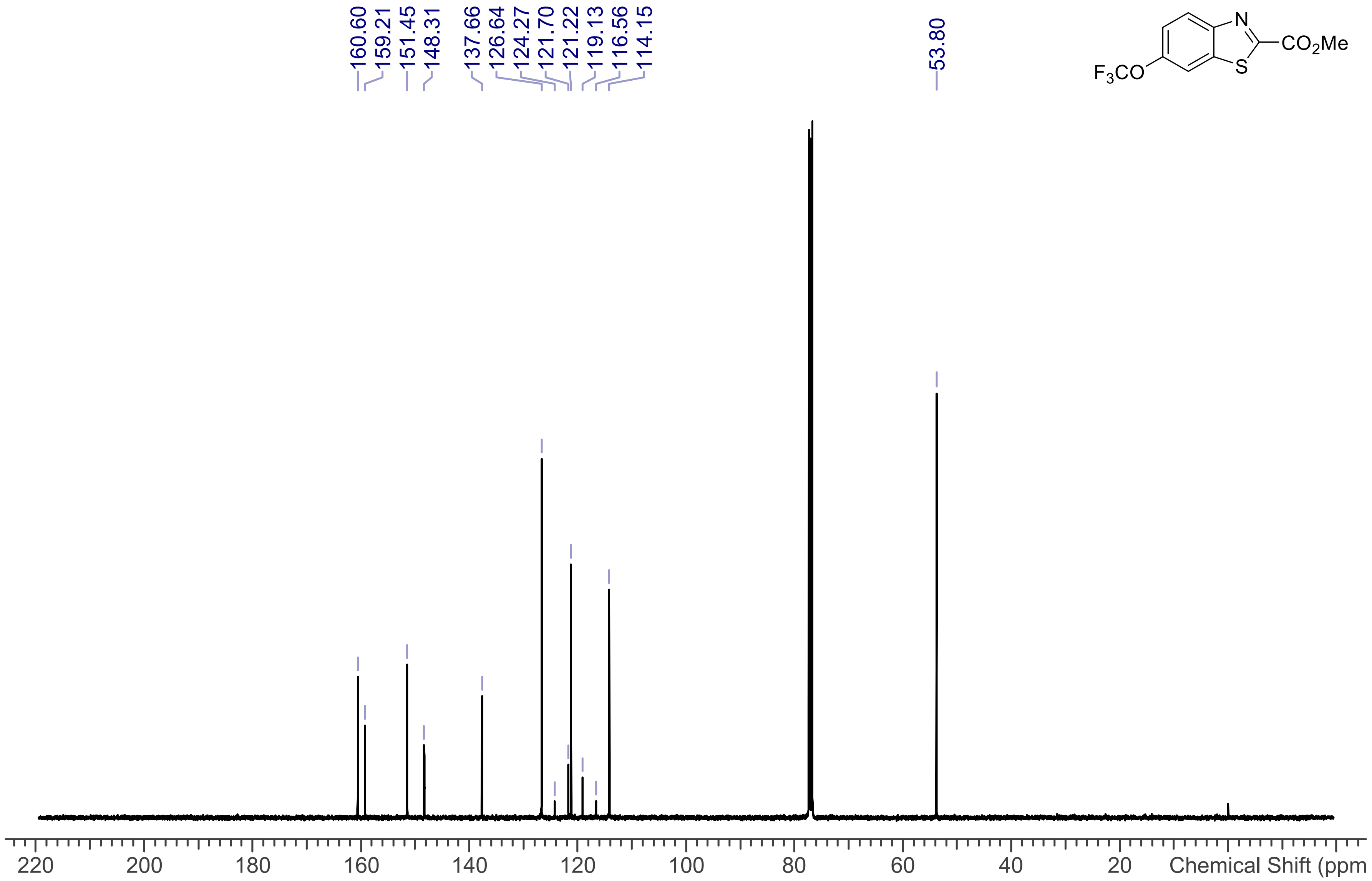

^{13}C NMR (101 MHz, $CDCl_3$) Methyl 6-Trifluoromethoxybenzo[*d*]thiazole-2-carboxylate (**3x**).

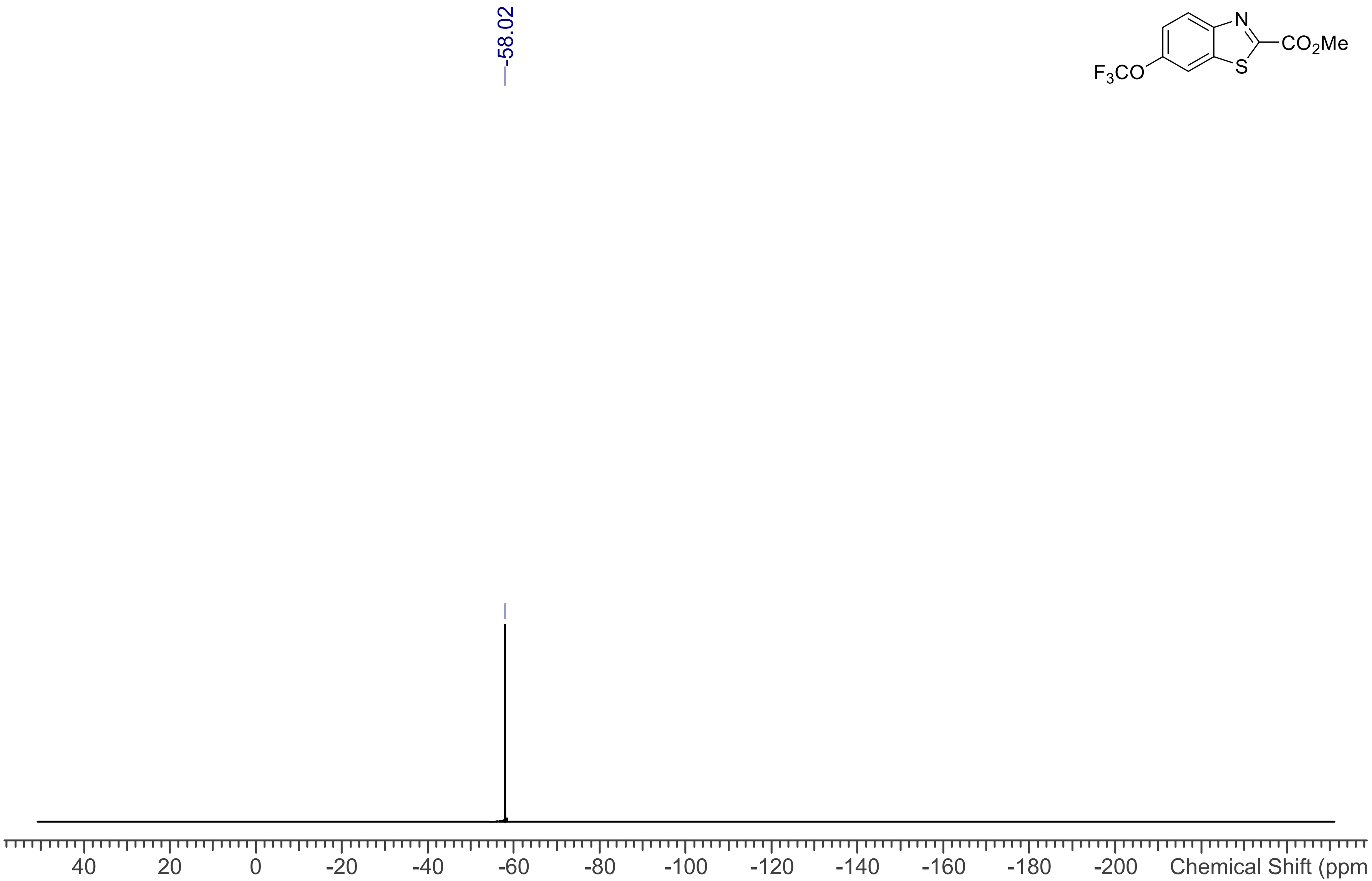

^{19}F NMR (376 MHz, $CDCl_3$) Methyl 6-Trifluoromethoxybenzo[*d*]thiazole-2-carboxylate (**3x**).

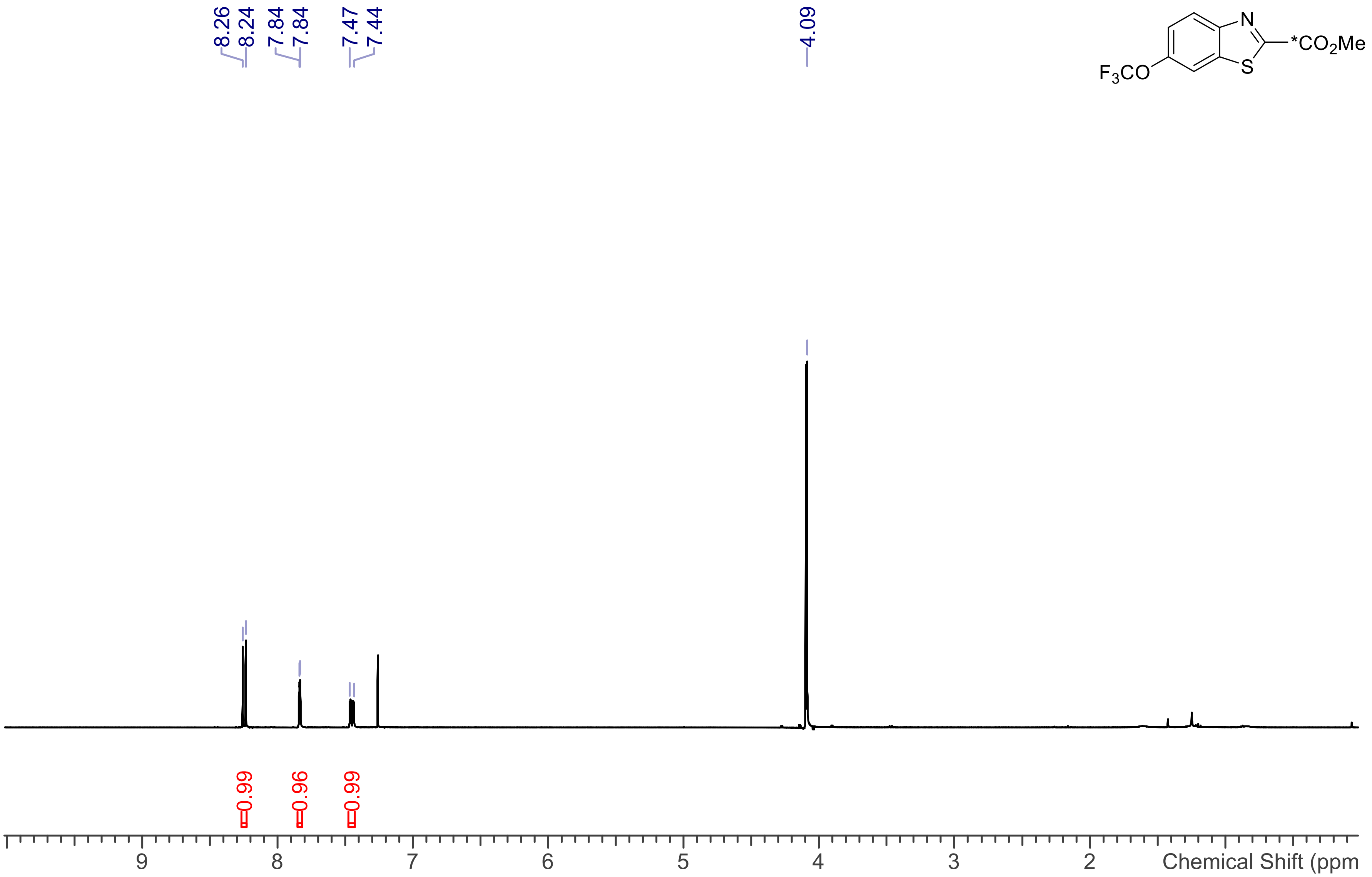

^{1}H NMR (400 MHz, $CDCl_3$) [^{13}C]Methyl 6-Trifluoromethoxybenzo[*d*]thiazole-2-carboxylate (**3x***).

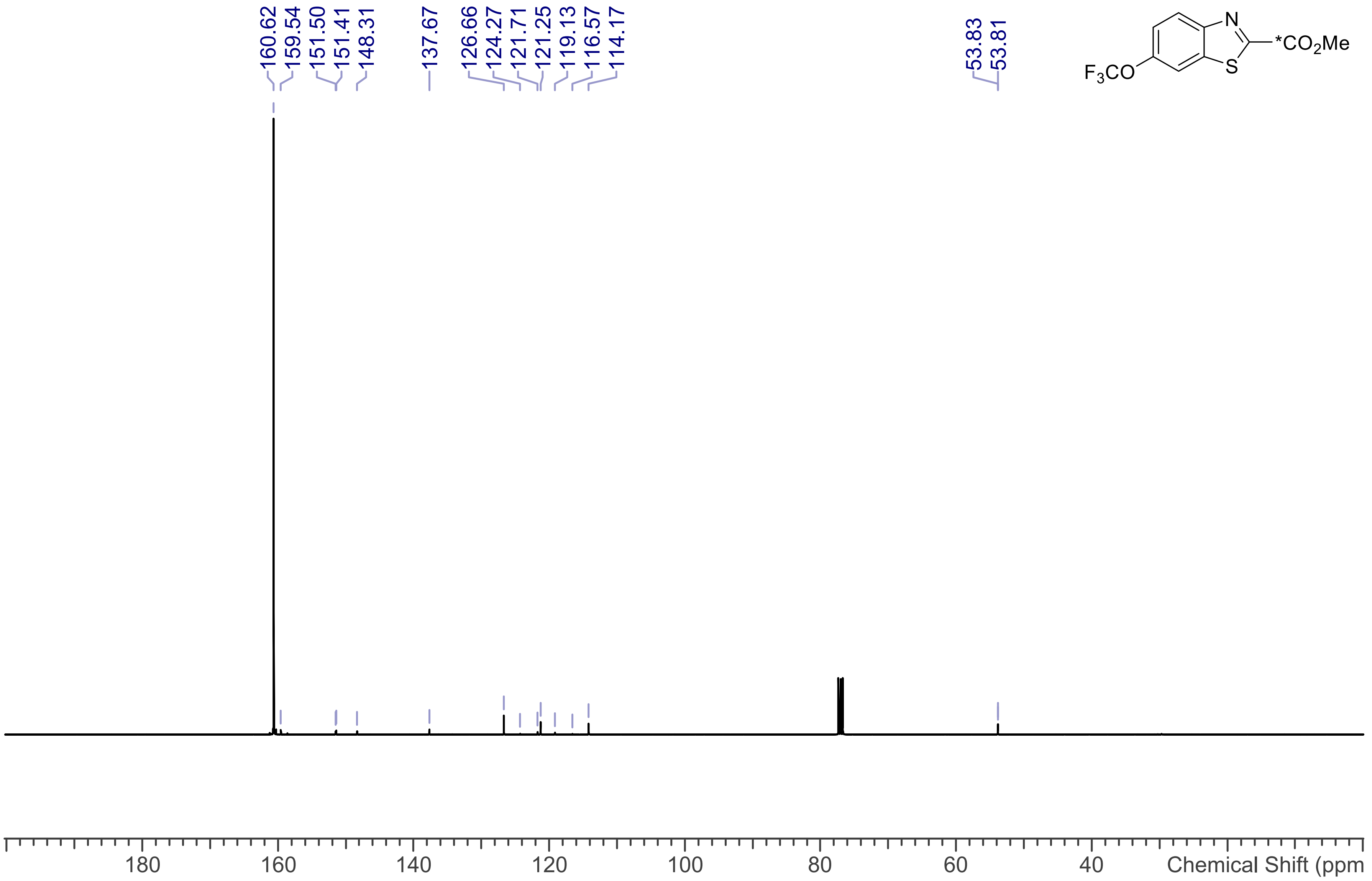

^{13}C NMR (101 MHz, $CDCl_3$) [^{13}C]Methyl 6-Trifluoromethoxybenzo[*d*]thiazole-2-carboxylate (**3x***).

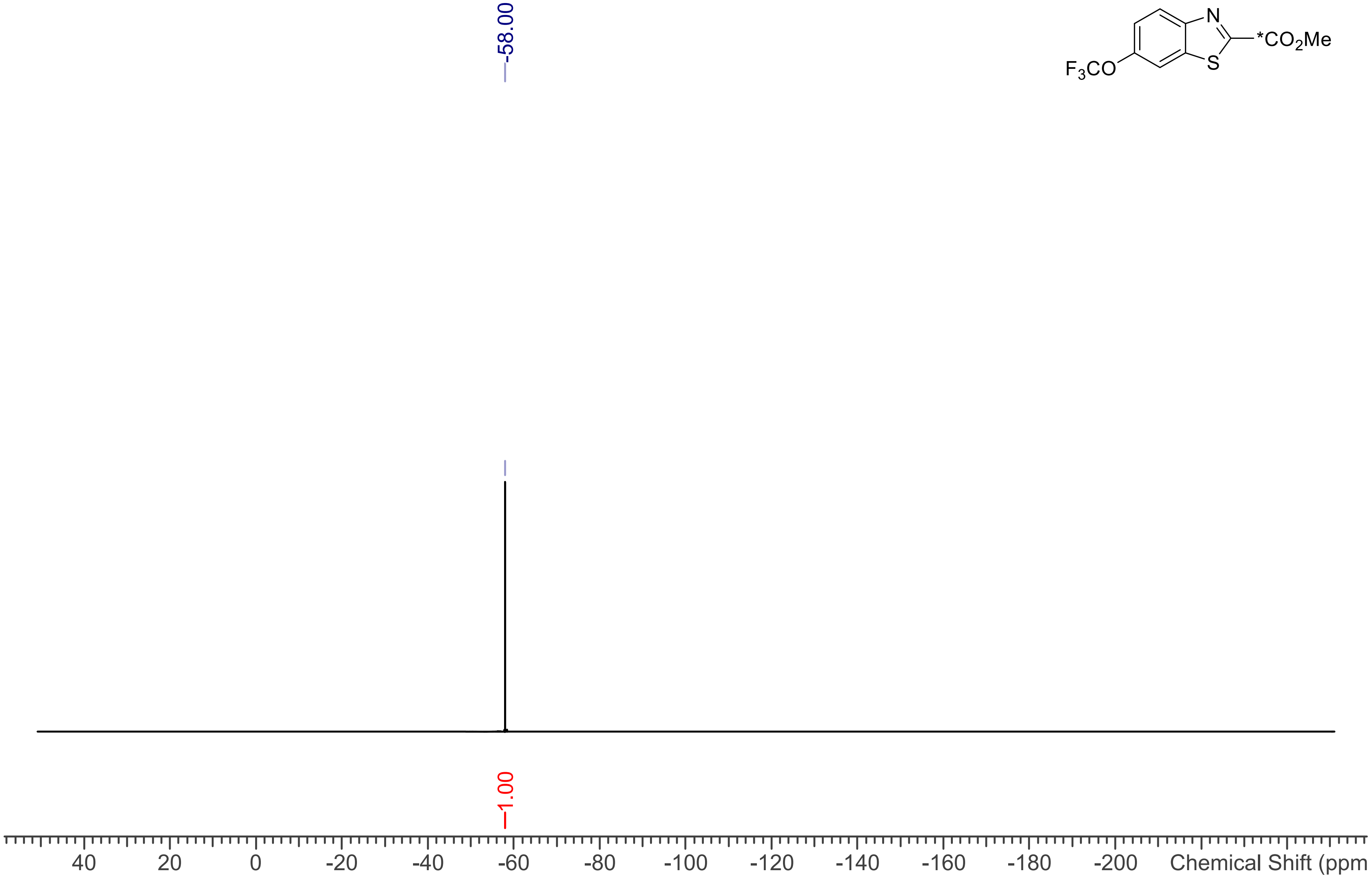

$^{19}F\{^1H\}$ NMR (376 MHz, $CDCl_3$) [^{13}C]Methyl 6-Trifluoromethoxybenzo[*d*]thiazole-2-carboxylate (**3x***).

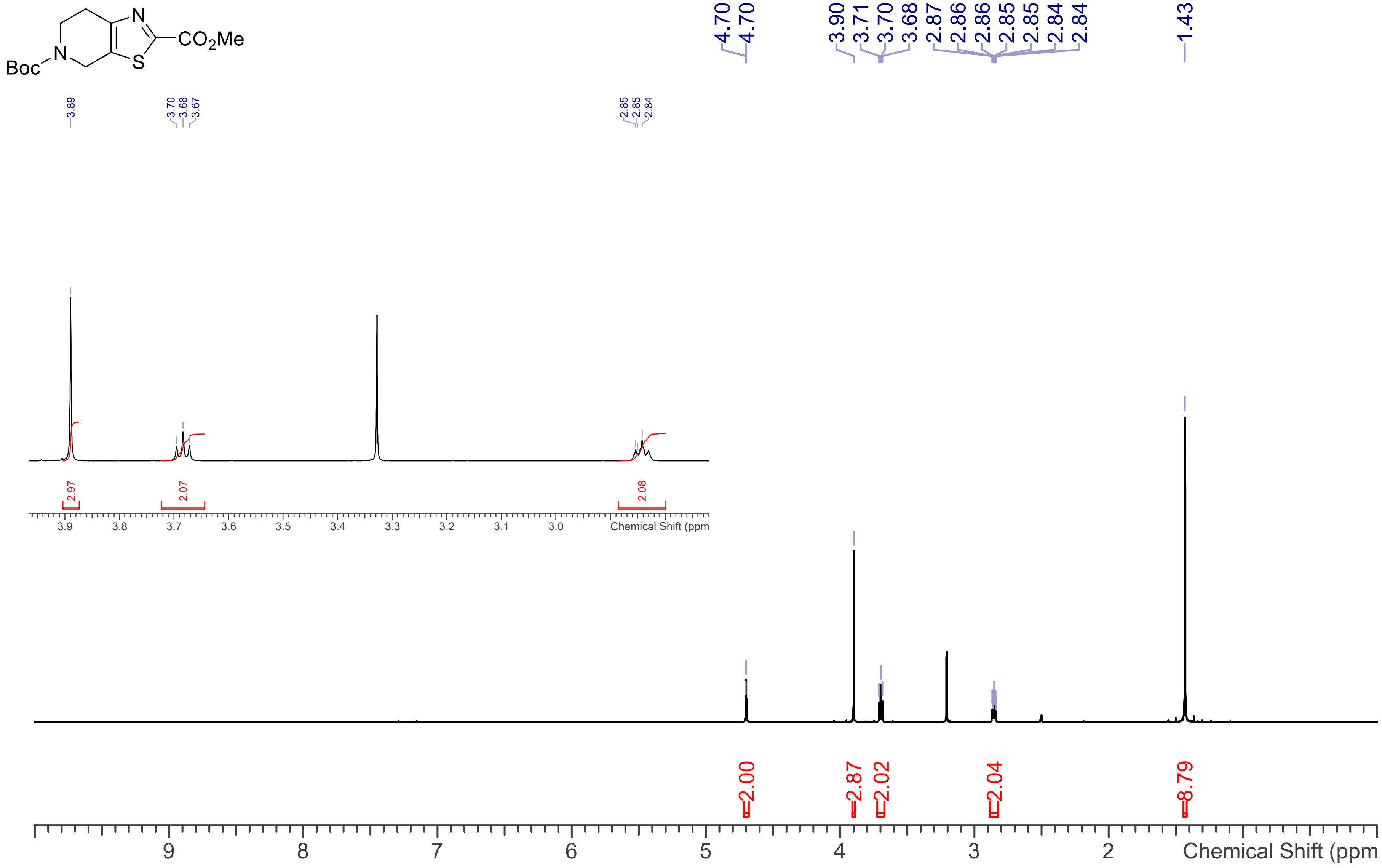

^{1}H NMR (500 MHz, DMSO-d6) 5-(tert-butyl)-2-methyl-6,7-dihydrothiazolo[5,4-c]pyridine-2,5(4*H*)-dicarboxylate (**3y**).

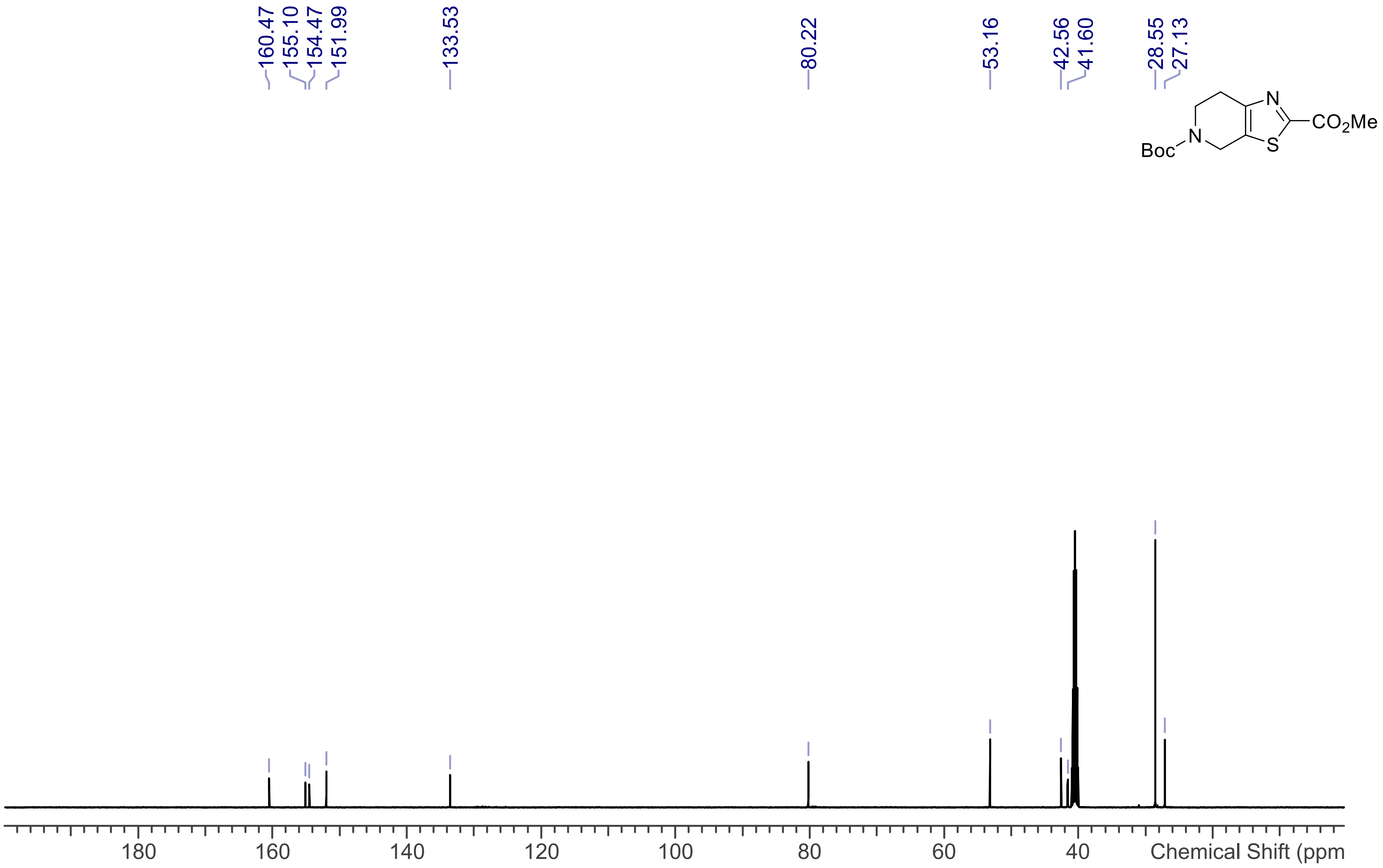

^{13}C NMR (125 MHz, DMSO-d$_6$) 5-(tert-butyl)-2-methyl-6,7-dihydrothiazolo[5,4-c]pyridine-2,5(4*H*)-dicarboxylate (**3y**).

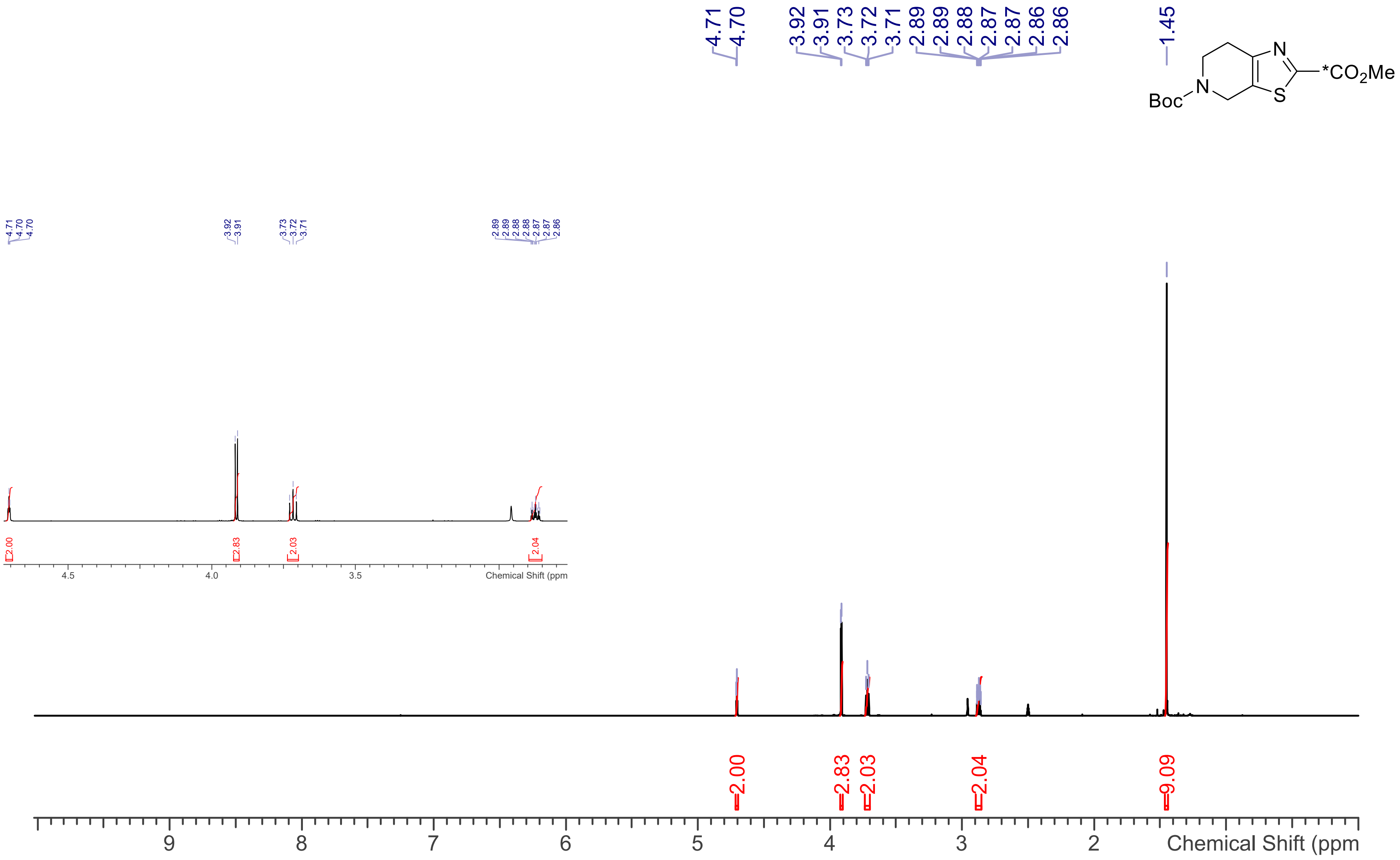

^{1}H NMR (500 MHz, DMSO-d$_6$) [^{13}C]5-(tert-butyl)-2-methyl-6,7-dihydrothiazolo[5,4-c]pyridine-2,5(4*H*)-dicarboxylate (**3y***).

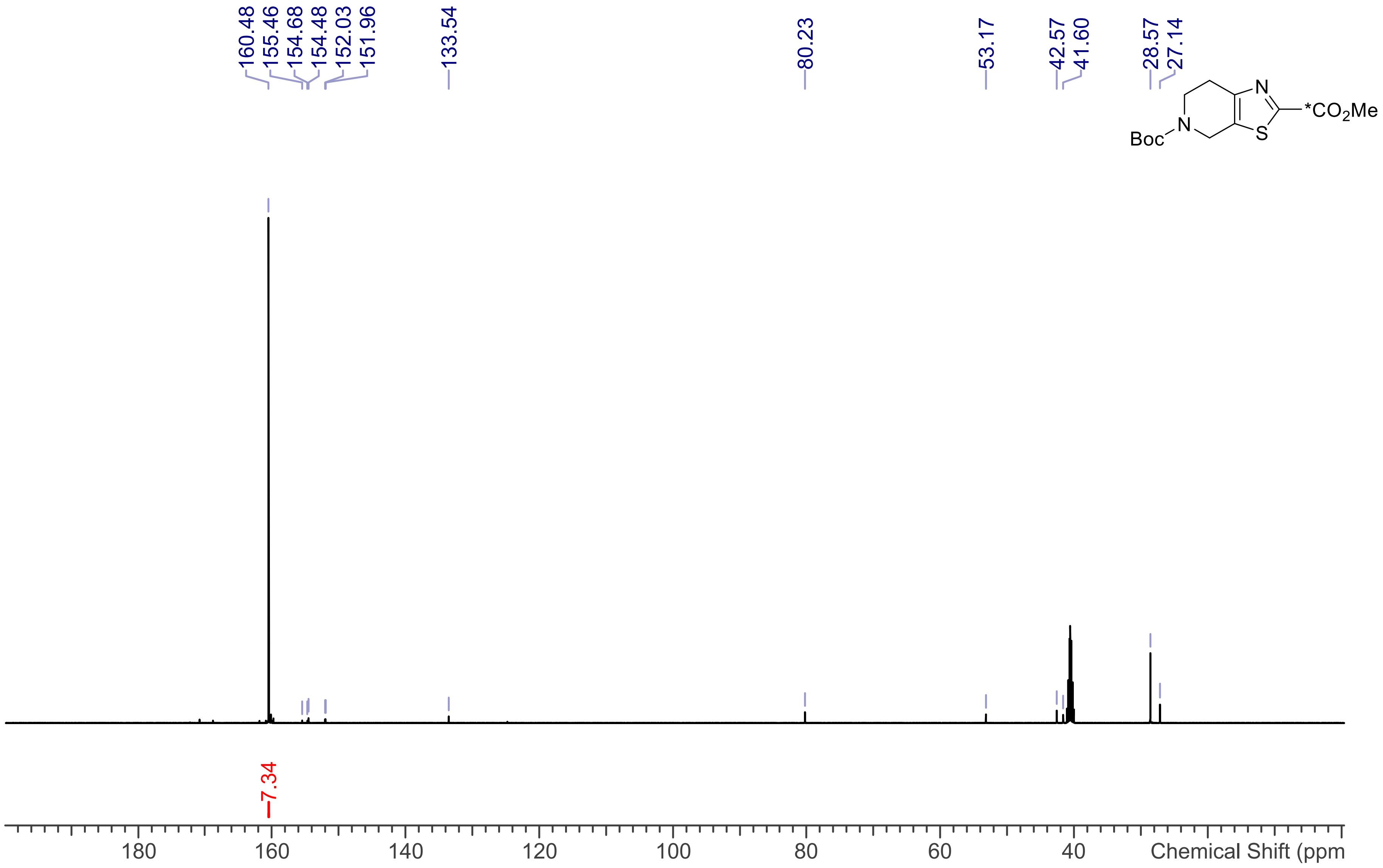

^{13}C NMR (125 MHz, DMSO-d_6) [^{13}C]5-(tert-butyl)-2-methyl-6,7-dihydrothiazolo[5,4-c]pyridine-2,5(4*H*)-dicarboxylate (**3y***).

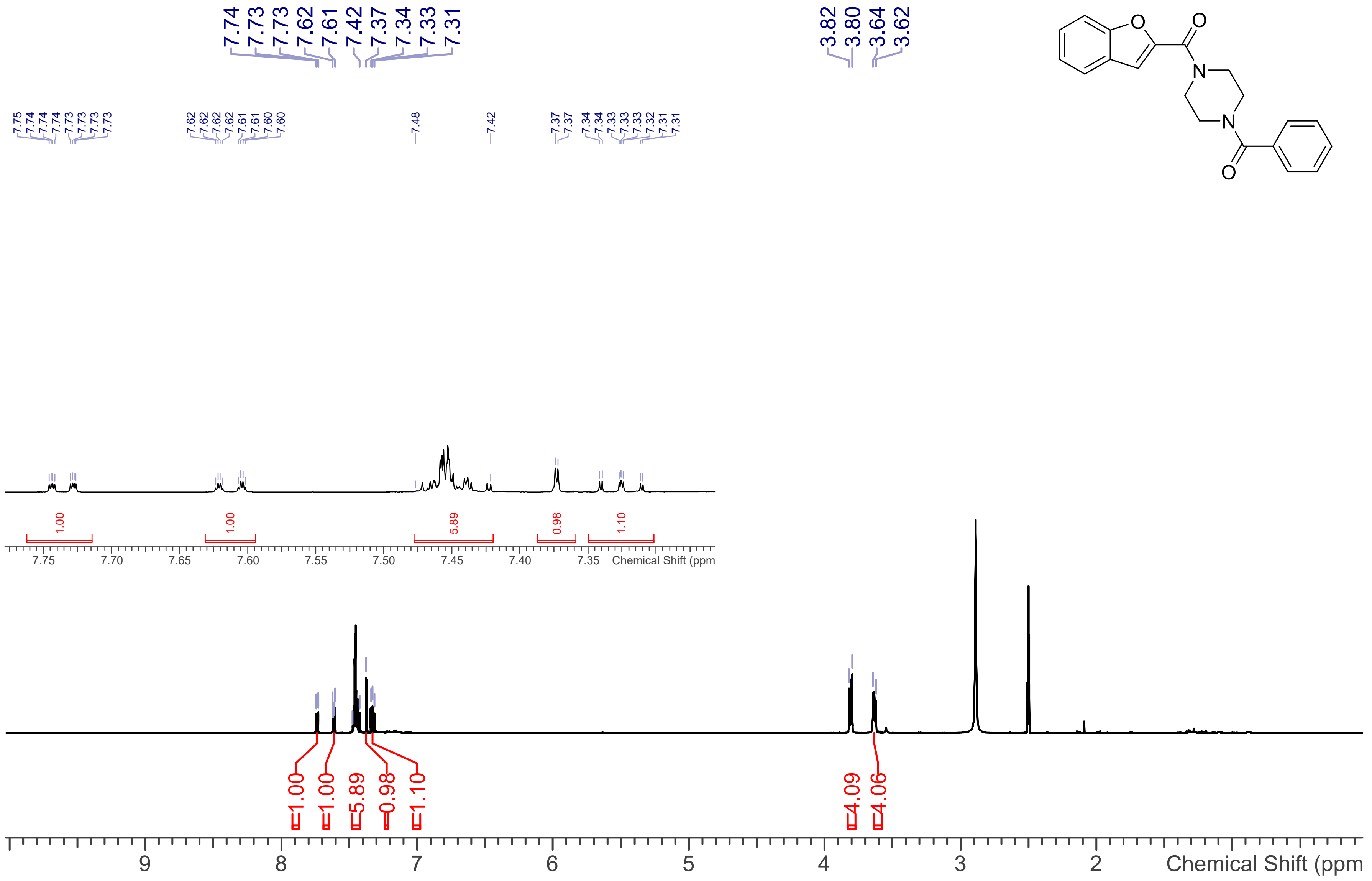

^{1}H NMR (500 MHz, DMSO-d6) (4-(benzofuran-2-carbonyl)piperazin-1-yl)(phenyl)methanone (**3z**).

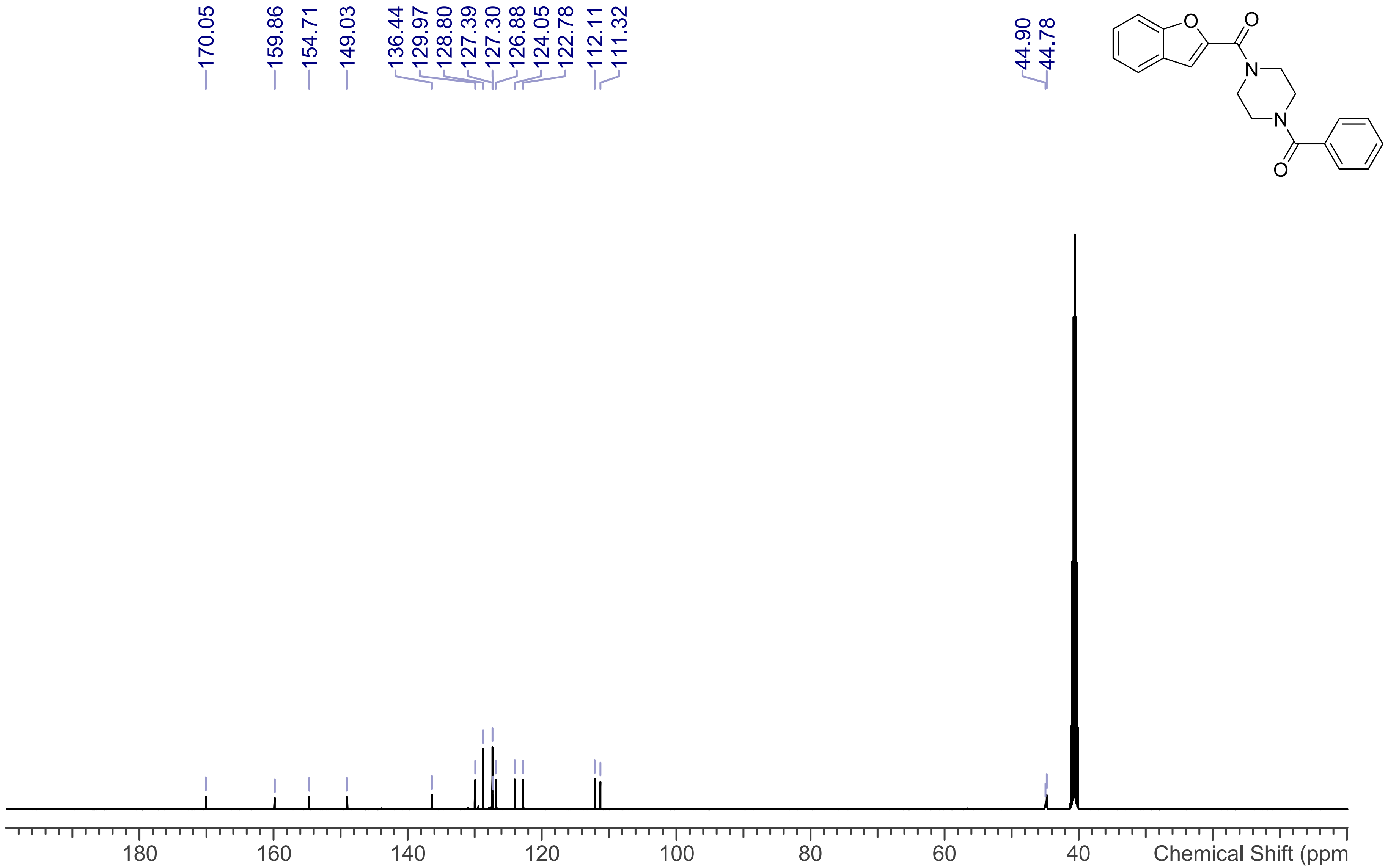

^{13}C NMR (126 MHz, DMSO-d_6) (4-(benzofuran-2-carbonyl)piperazin-1-yl)(phenyl)methanone (**3z**).

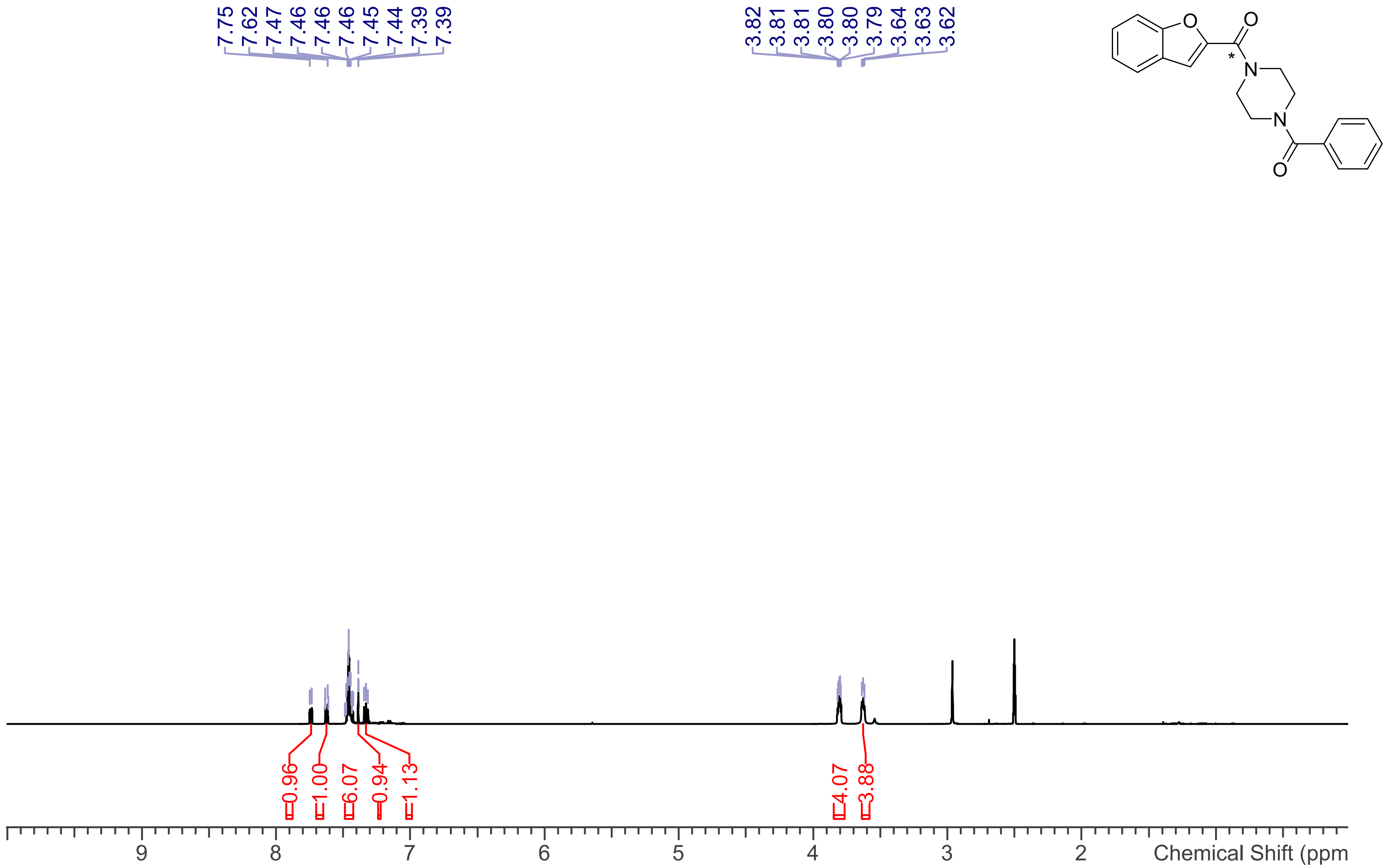

^{1}H NMR (126 MHz, DMSO-d$_6$) [^{13}C](4-(benzofuran-2-carbonyl)piperazin-1-yl)(phenyl)methanone (**3z***).

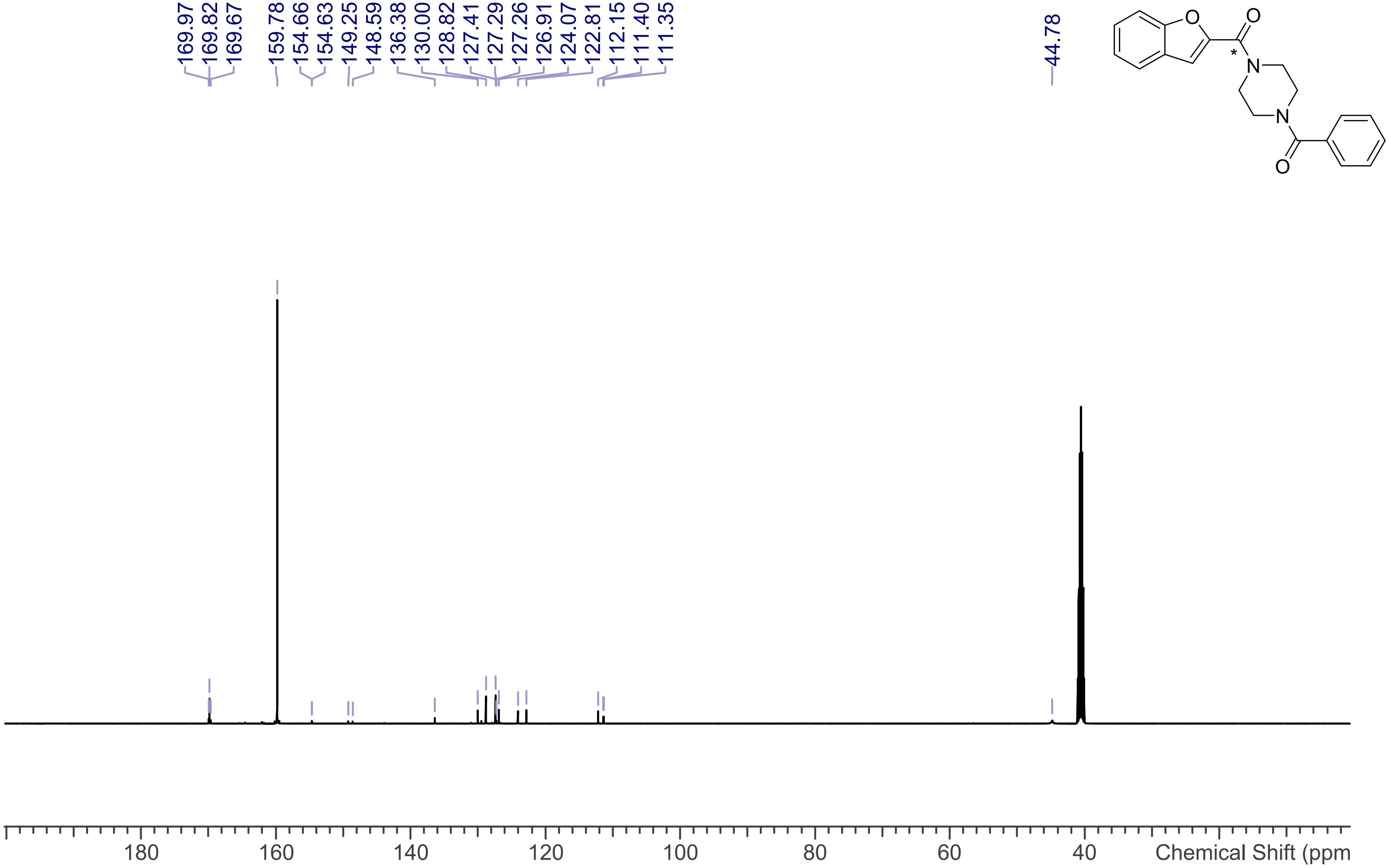

^{13}C NMR (126 MHz, DMSO-d$_6$) [^{13}C](4-(benzofuran-2-carbonyl)piperazin-1-yl)(phenyl)methanone (**3z***).

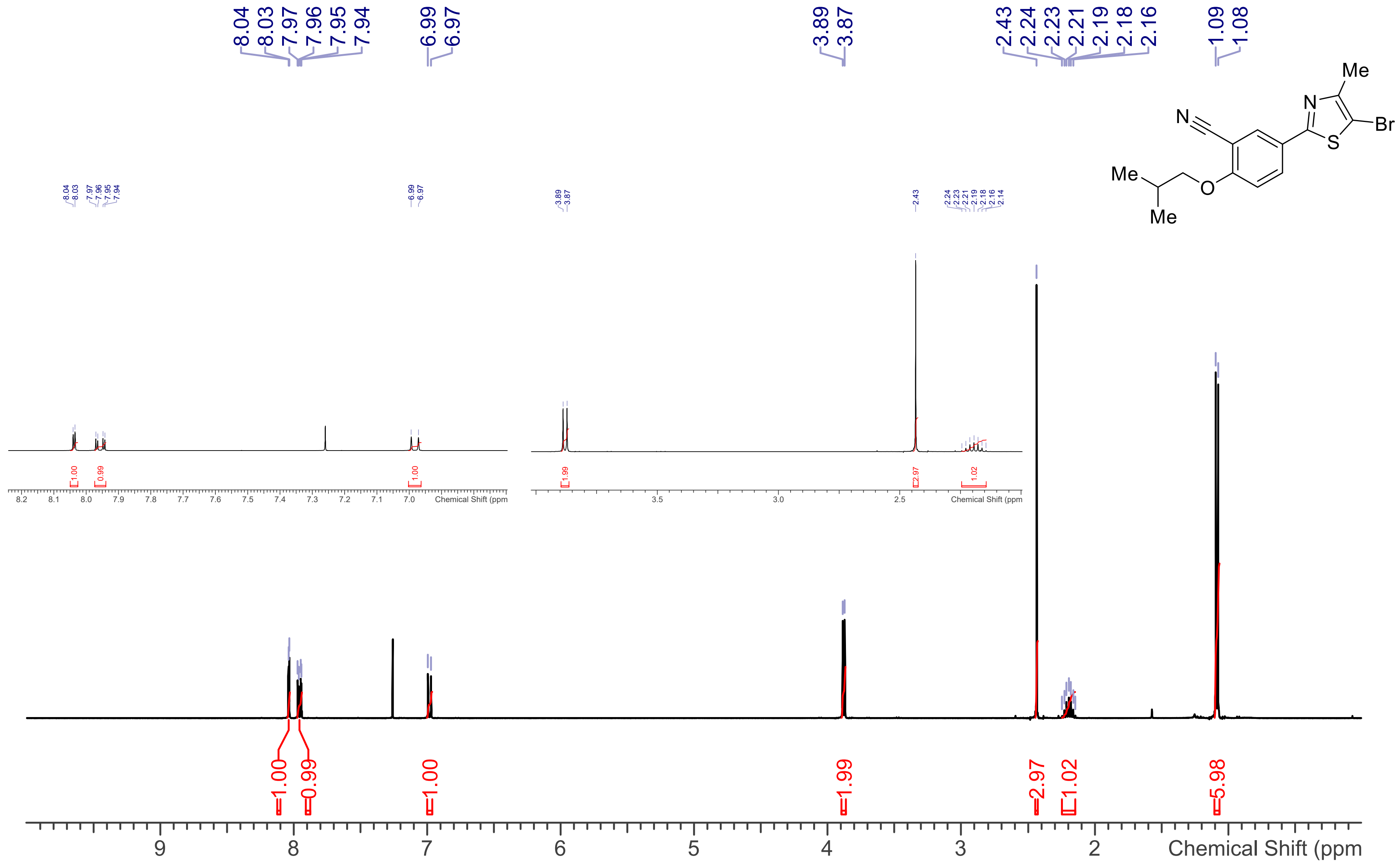

^{1}H NMR (400 MHz, $CDCl_3$) 5-(5-Bromo-4-methylthiazol-2-yl)-2-isobutoxybenzonitrile (**1aa-Br**).

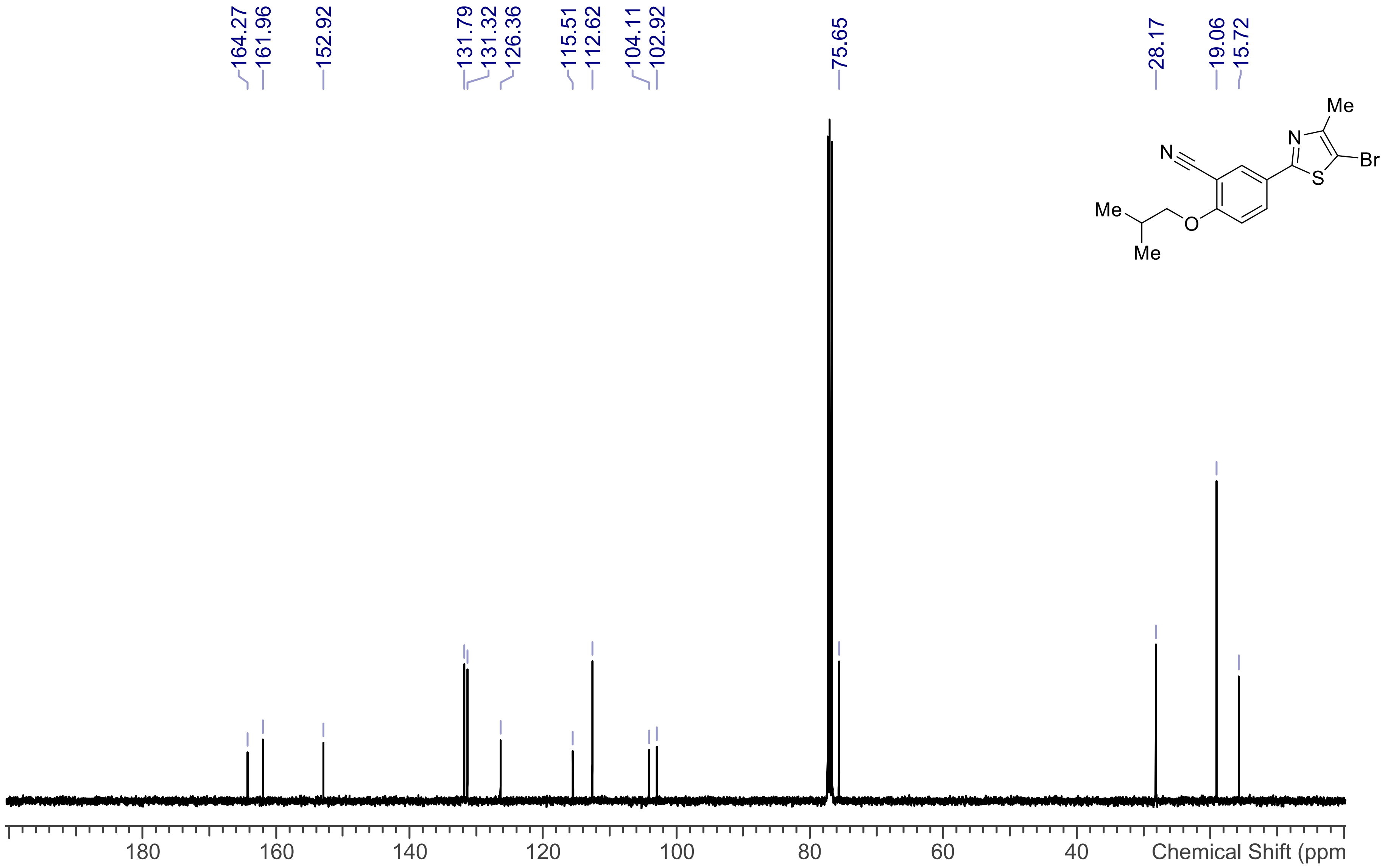

^{13}C NMR (101 MHz, $CDCl_3$) 5-(5-Bromo-4-methylthiazol-2-yl)-2-isobutoxybenzonitrile (**1aa-Br**).

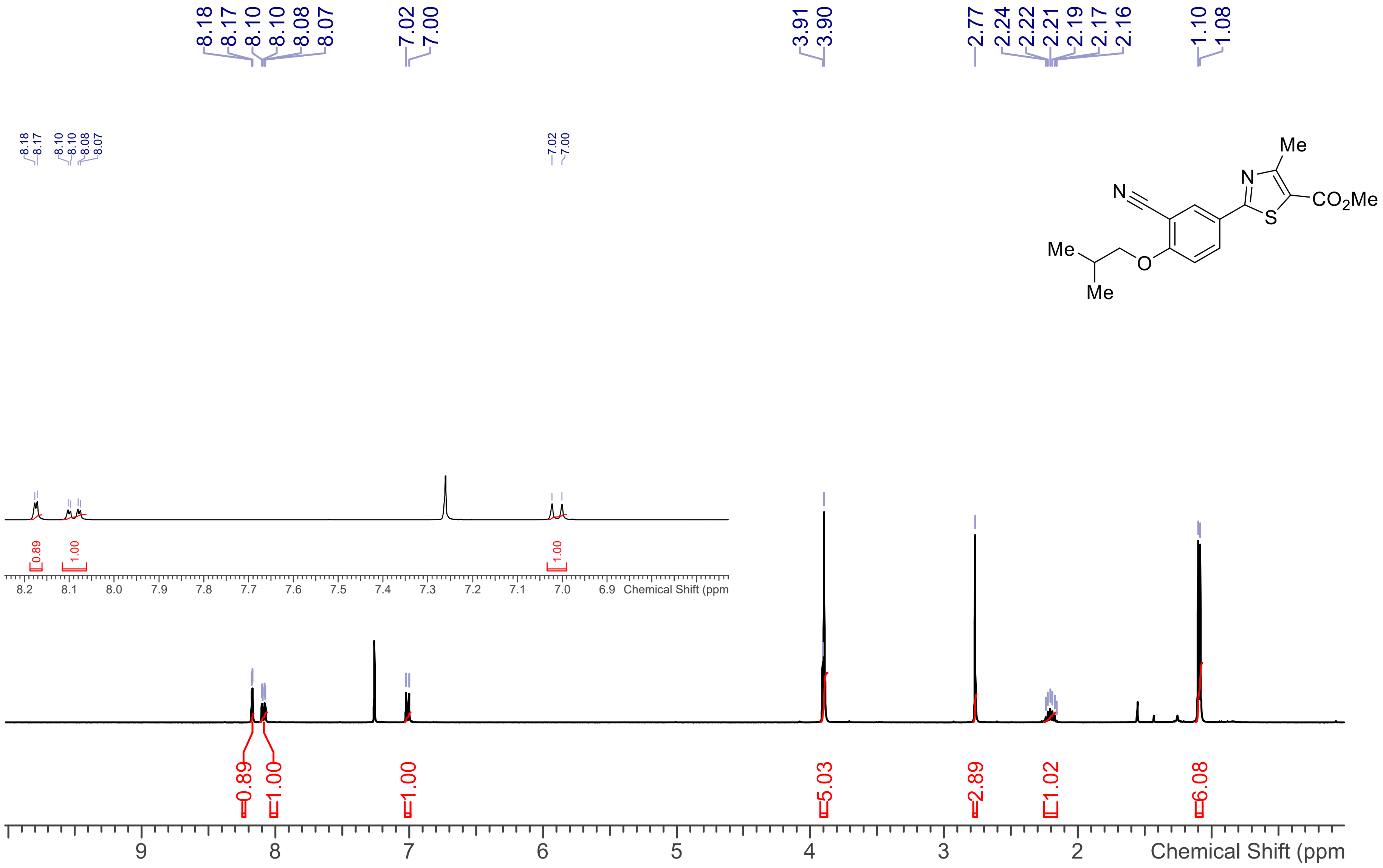

^{1}H NMR (400 MHz, $CDCl_3$) Methyl-2-(3-cyano-4-isobutoxyphenyl)-4-methylthiazole-5-carboxylate (**3aa**).

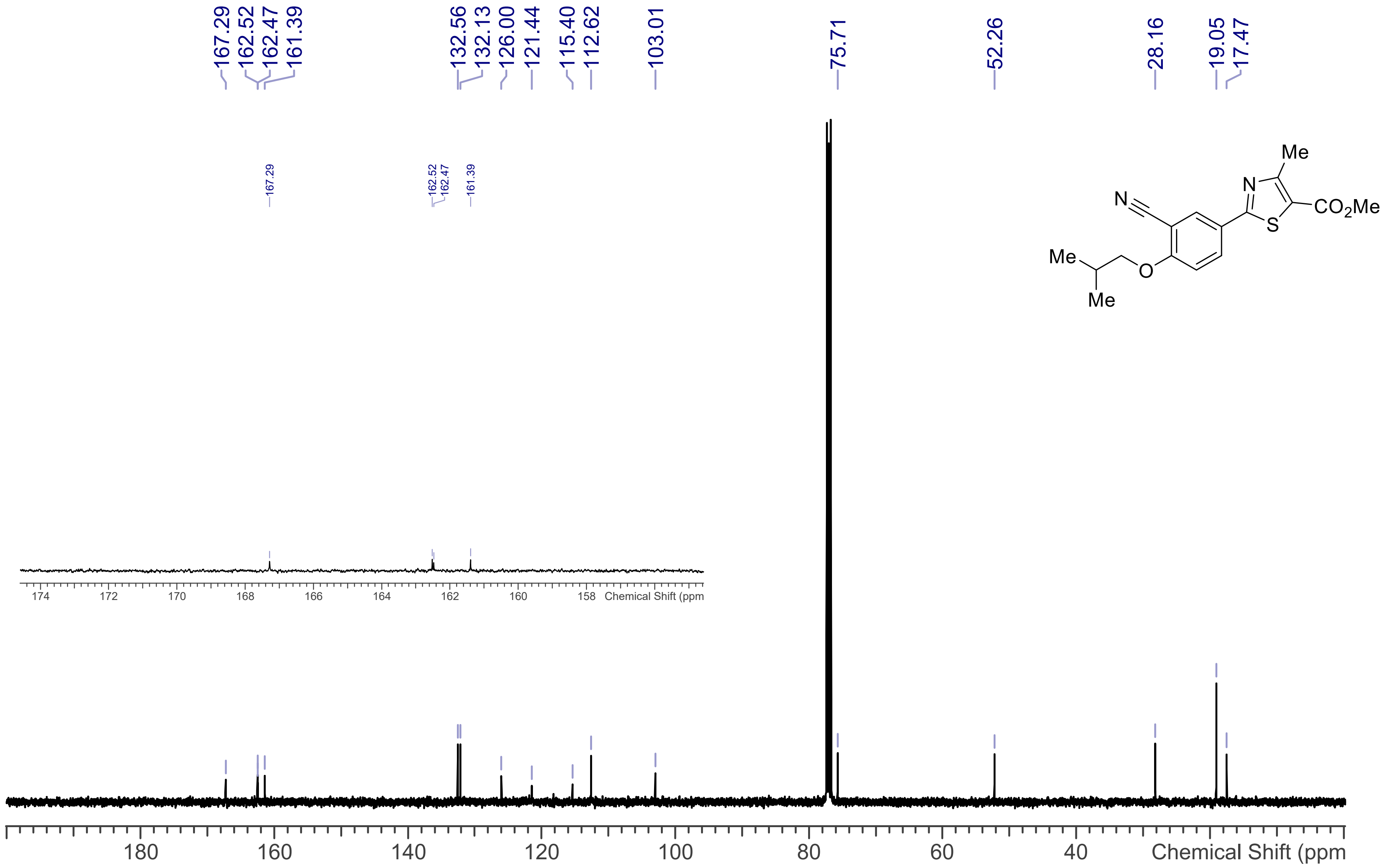

^{13}C NMR (101 MHz, $CDCl_3$) Methyl-2-(3-cyano-4-isobutoxyphenyl)-4-methylthiazole-5-carboxylate (**3aa**).

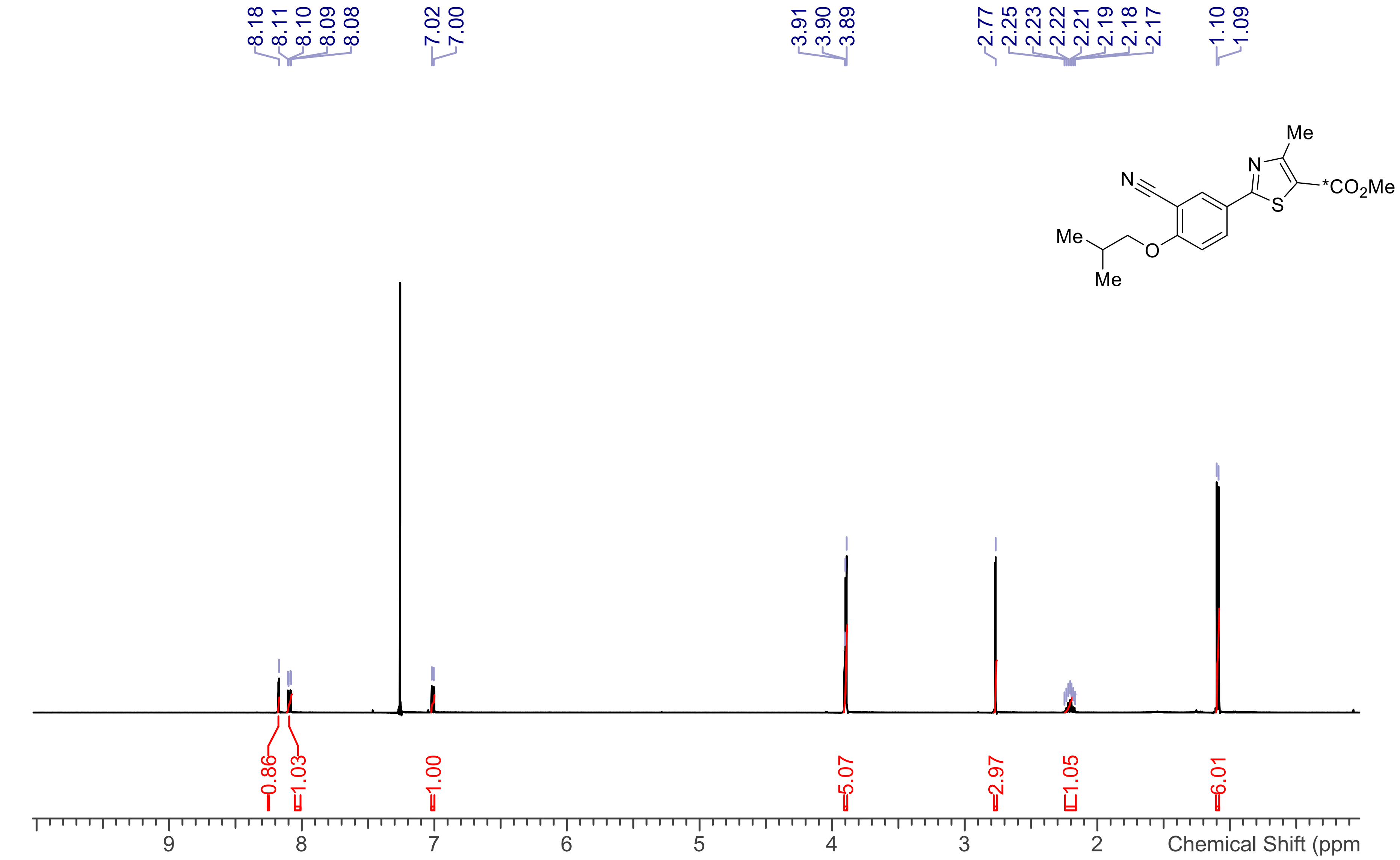

^{1}H NMR (400 MHz, $CDCl_3$) [^{13}C]Methyl-2-(3-cyano-4-isobutoxyphenyl)-4-methylthiazole-5-carboxylate (**3aa***).

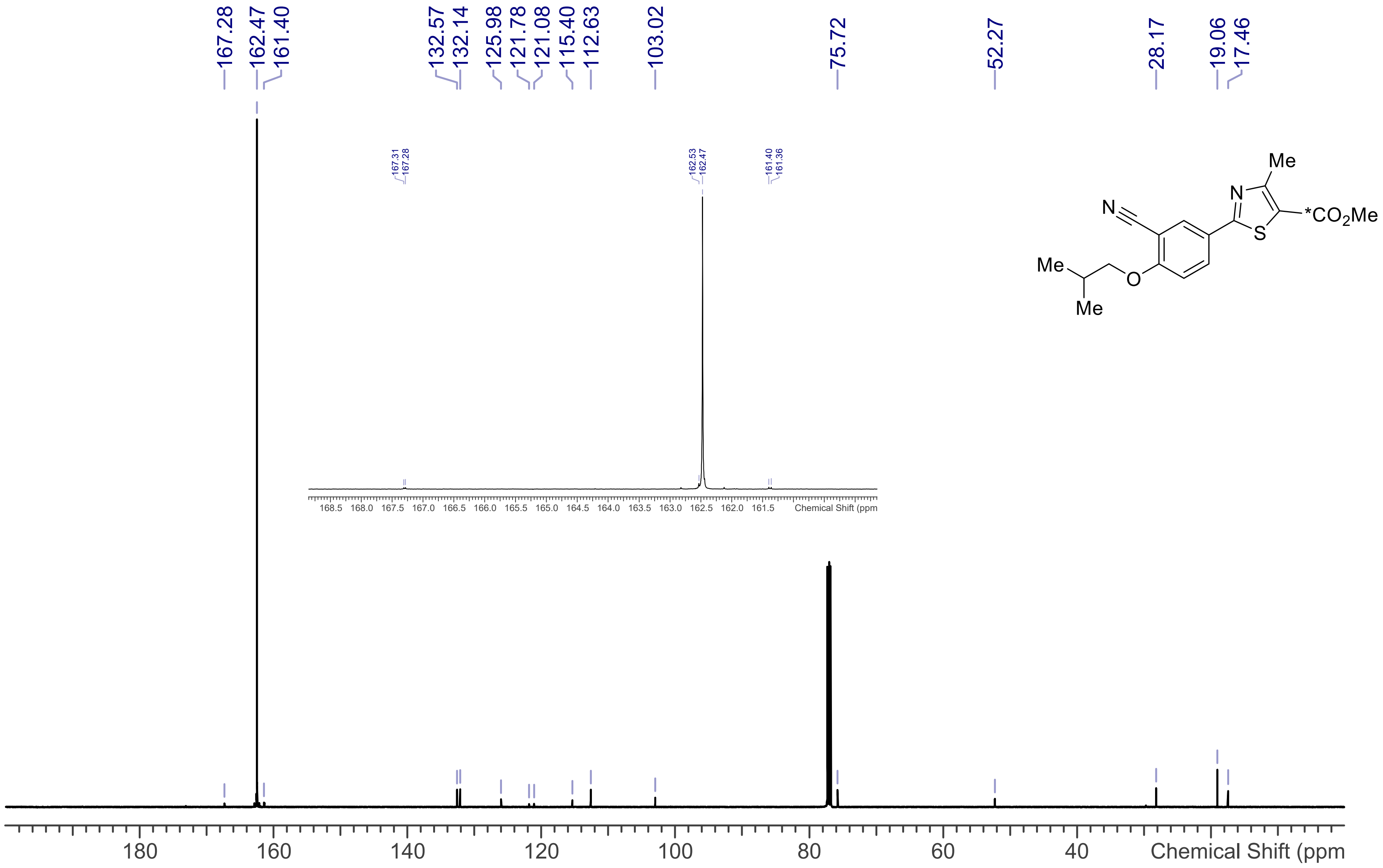

^{13}C NMR (101 MHz, $CDCl_3$) [^{13}C]Methyl-2-(3-cyano-4-isobutoxyphenyl)-4-methylthiazole-5-carboxylate (**3aa***).

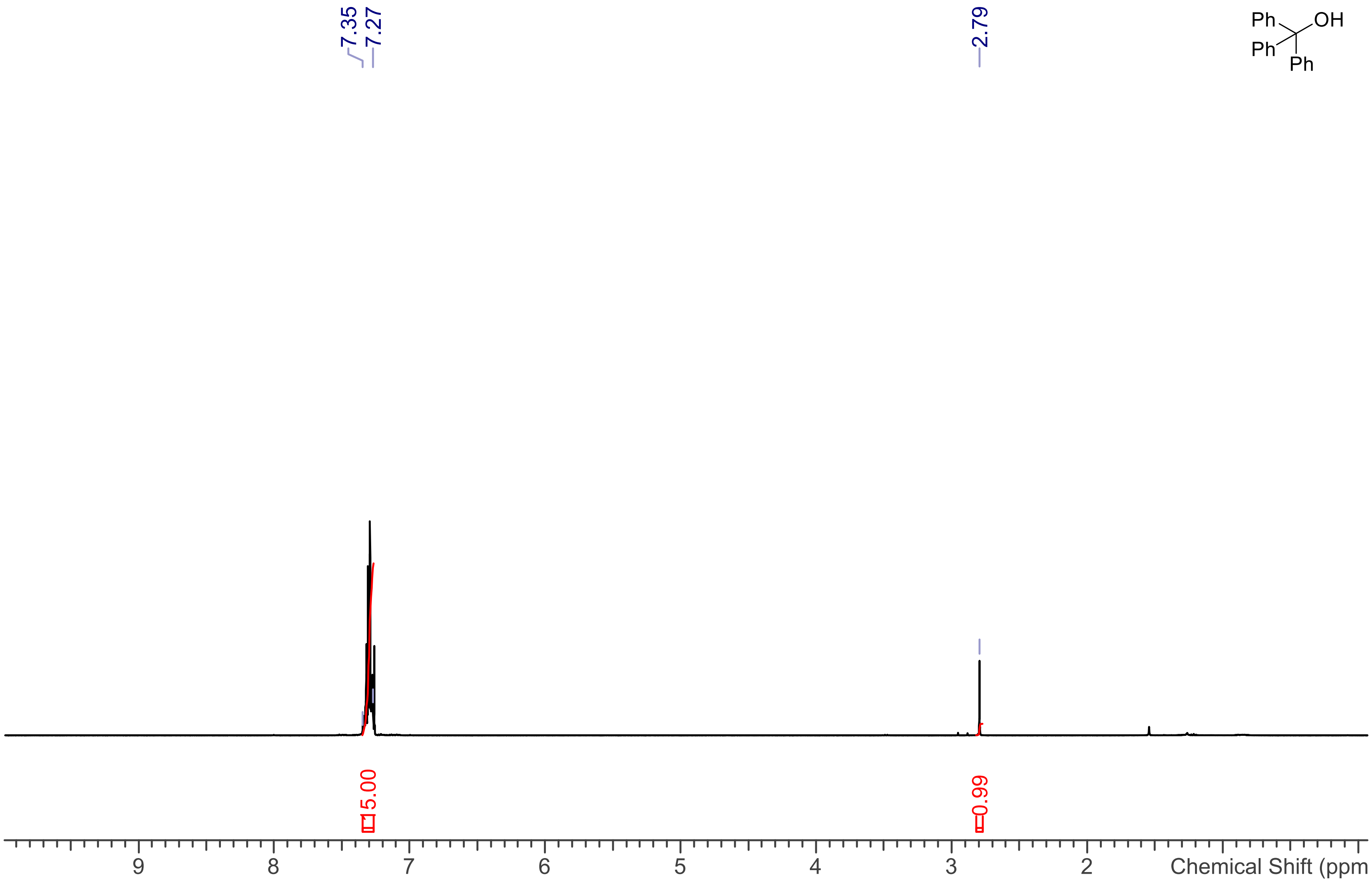

^{1}H NMR (400 MHz, $CDCl_3$) Triphenylmethanol.

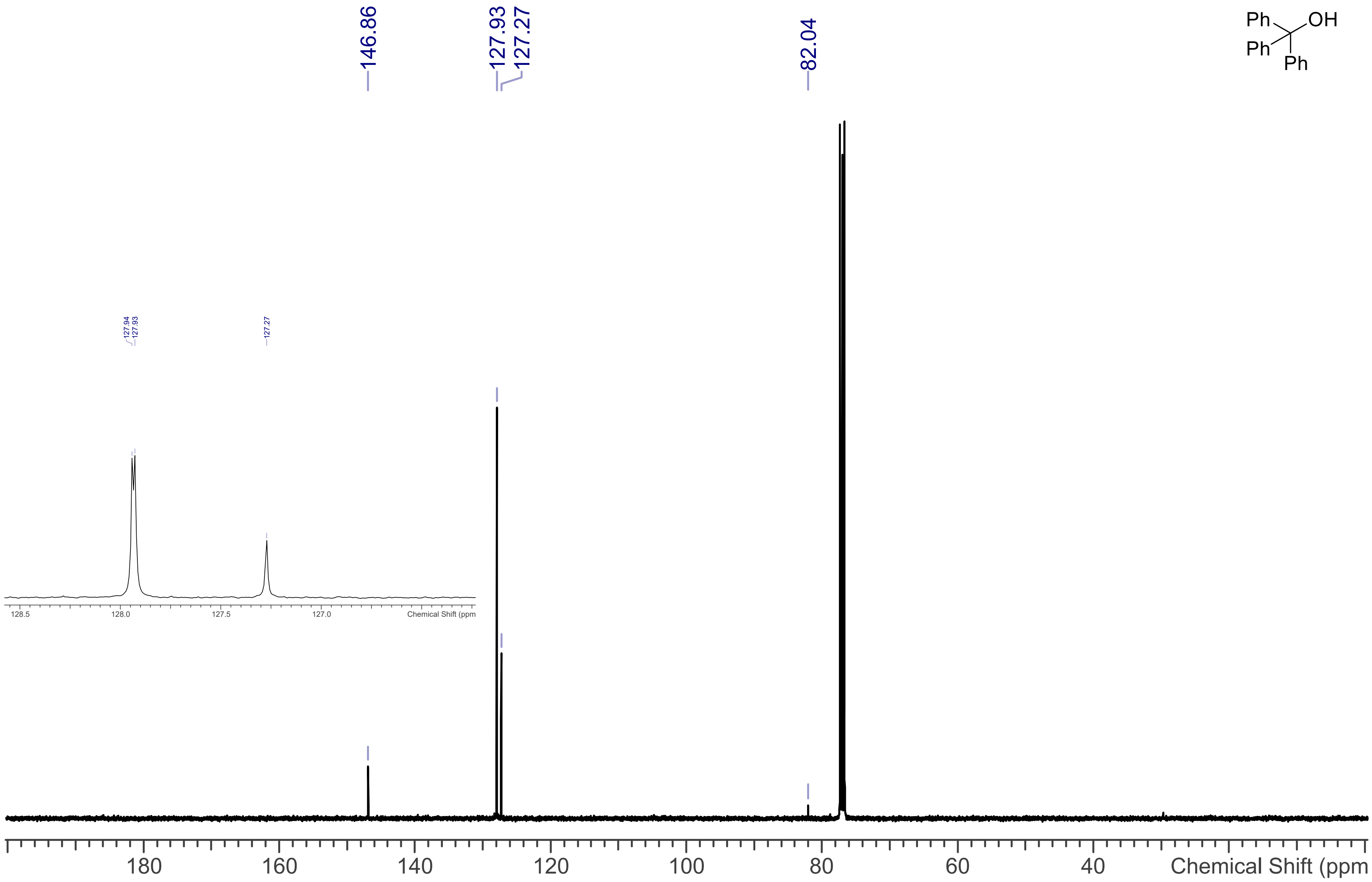

^{1}H NMR (400 MHz, $CDCl_3$) Triphenylmethanol.
